# Supplementary material for: Trends in outcomes used to measure the effectiveness of UK-based support interventions and services targeted at adults with experience of domestic and sexual violence and abuse: a scoping review
Source: BMJ Open. 2024 Apr 30;14(4):e074452. doi: 10.1136/bmjopen-2023-074452 (PMC11086554; doi:10.1136/bmjopen-2023-074452)
Supplement: Supplementary data [file bmjopen-2023-074452supp001.pdf]

Appendix 1: Glossary

Advocacy: Adapted from the National Institute for Health and Clinical Excellence (NICE) Domestic Violence and Abuse: Multi-agency Working public health guideline (PH50)[1]. In general, advocacy for people who have experienced DSVA includes: legal, housing and financial advice: access to and use of community resources such as refuges, emergency housing and psychological interventions; and safety planning advice. The activities may differ according to the level of risk facing the person. Crisis advocacy involves working with the person for a limited period of time (they may then be referred on to more specialist agencies). Practitioners providing advocacy can also provide ongoing support and informal counselling. The intensity of the advocacy provided may vary. It may last for a year – or longer, if the person is particularly vulnerable.

By-and-for: By-and-for services are lived experience organisation, where all of the staff, senior managers, Board of Trustees, and women and girls using services are Black and minoritised women. Such services have a Black feminist ethos, locating themselves as anti-racist, social justice and intersectional organisations.

Domestic and sexual violence and abuse (DSVA): This review works to the UK government definition of domestic violence and abuse[2], and The Istanbul Convention[3], The World Health Organisation[4], and The Rome Statute of the International Criminal Court's (ICC) Elements of Crimes definitions of sexual violence and abuse[5]. In brief, domestic violence and abuse is defined as behaviour within an intimate relationship or family which includes acts of physical violence, sexual violence, emotional or psychological abuse, and controlling behaviours. This also includes 'honour'-based violence and FGM. Sexual violence and abuse is defined as any non-consensual act of sexual nature, extending to attempts to obtain a sexual act, unwanted sexual comments or advances, or acts to traffic, using force, threat of force or coercion.

Independent domestic violence advisers (IDVAs): Adapted from the NICE PH50 guideline. Also known as independent domestic violence advocates, IDVAs work primarily with people at high risk of domestic violence and abuse, independently of any one agency, to secure their safety and the safety of their children. Serving as the primary point of contact, IDVAs normally work with their clients from the point of crisis to assess the level of risk, discuss the options and develop plans that address their immediate safety, as well as longer-term solutions. In many areas they are funded by the local community safety partnership, in some areas they are funded by the police or local authorities.

Intervention: Adapted from the NICE Glossary. A treatment, service, project, or programme.

Intimate partner violence (IPV): Adapted from the World Health Organisation's definition. Intimate partner violence refers to behaviour within an intimate relationship that causes physical, sexual or psychological harm, including acts of physical aggression, sexual coercion, psychological abuse and controlling behaviours. This definition covers violence by both current and former spouses and partners.

Organisation: In the context of this review, organisations refer exclusively to those that carry out activities relating to DSVA. Organisations may include both specialist service providers, and organisations that do not directly provider services (such as women's rights organisations that may focus on activities such as campaigning, research, supporting specialist services, training, and education, for issues relating to DSVA, for example).

People who have experienced DSVA: Throughout this review, people who experience DSVA refers to those who are victims or survivors of DSVA.

Perpetrator programmes: Any group or individual intervention for people who have perpetrated DSVA, with the aim of reducing the incidence of violence by changing attitudes, behaviours and/or beliefs of perpetrators.

Primary, secondary, and tertiary prevention: Primary prevention refers to any action, strategy or policy that works to stop domestic violence from occurring in the first place. Primary prevention seeks to reduce the overall likelihood that anyone will become a victim or perpetrator of domestic violence by creating conditions that make violence less likely to occur. Secondary prevention refers to intervening and responding to violence that has already occurred in order to stop violence from happening again. Secondary prevention activities can include refuge, counselling, and safety planning. Tertiary prevention refers to ongoing support to victims and ongoing accountability for abusers. Tertiary prevention activities address the long-term consequences of domestic violence. They can include support groups and other resources for survivors to help them heal so that they do not experience abuse again. They can also include perpetrator programs for abusers to prevent them from continuing to perpetrate violence in the future.

Refuge or shelter: Adapted from the NICE PH50 guideline. Residential service – a safe house – provided for adults (usually women) and children who are experiencing domestic violence and abuse.

Service provider: Primary, community, including third sector, secondary and tertiary care providers of health and social care services, including prison health services and criminal justice agencies. In the context of DSVA, the service providers are predominantly third sector providers. Third sector service providers may be specialist DSVA providers (i.e., they only provide services relating to DSVA) or non-specialist-DSVA providers (i.e., they provide specialist support services for DSVA as well as support services for other issues). Outside of the third sector, service providers such as the NHS, police and courts may provide some DSVA specific services.

Third sector organisations: In this review the definition of third sector services provided by the National Audit Office has been adopted. 'Third sector organisations' is a term used to describe the range of organisations that are neither public sector nor private sector. It includes voluntary and community organisations (both registered charities and other organisations such as associations, self-help groups and community groups), social enterprises, mutuals and co-operatives. Third sector organisations generally: are independent of government. This is also an important part of the history and culture of the sector; are 'value-driven'. This means they are motivated by the desire to achieve social goals (for example, improving public welfare, the environment or economic well-being) rather than the desire to distribute profit; and reinvest any surpluses generated in the pursuit of their goals. For this reason they are sometimes called 'not-for-profit organisations'. A better term is 'not-for-personal-profit'. In many cases, third sector organisations need to make surpluses (or 'profits') to be financially sustainable.

References

1. National Institute for Health and Care Excellence. *Domestic violence and abuse: multi-agency working: Public health guideline [PH50]. Recommendations for research*. 2014; Available from: https://www.nice.org.uk/guidance/ph50/chapter/5-Recommendations-for-research.
2. Home Office. *Circular 003/2013: new government domestic violence and abuse definition*. 2013; Available from: https://www.gov.uk/government/publications/new-government-domestic-violence-and-abuse-definition/circular-0032013-new-government-domestic-violence-and-abuse-definition.
3. Council of Europe, *Council of Europe Convention on preventing and combating violence against women and domestic violence (CETS No. 210)*. 2011.
4. World Health Organization, *World report on violence and health*. 2002.
5. International Criminal Court, *Elements of Crime*. 2013.
