# Supplementary material for: Trends in outcomes used to measure the effectiveness of UK-based support interventions and services targeted at adults with experience of domestic and sexual violence and abuse: a scoping review
Source: BMJ Open. 2024 Apr 30;14(4):e074452. doi: 10.1136/bmjopen-2023-074452 (PMC11086554; doi:10.1136/bmjopen-2023-074452)
Supplement: Supplementary data [file bmjopen-2023-074452supp004.pdf]

Appendix 4: Details of included studies

Study	Peer review or grey lit	Study design	Setting/ sector	Type of intervention	Type of abuse*	No. of outcomes
ADVA (2009)[1]	Grey lit.	Service evaluation	Third	*"Repair"*<br>Perpetrator programme<br>• Comprising individual and group work sessions. Primarily cognitive behavioural, focusing on motivation, responsibility, safety and acknowledgment<br>Women's service<br>• Supportive befriending, information about legal and support services, expectation management, group work, telephone contact, feedback on partners' attendance, safety planning and monitoring and awareness raising	DVA	7
Advance (2020)[2]	Grey lit.	Service evaluation	Third	*"Minerva"*<br>Multi-service<br>• Advocacy for access to other community services<br>• One-to-one and group work support on a range of issues including emotional wellbeing, physical and mental health, violence and abuse, problematic substance use<br>• Benefits and debt advice, housing	DVA	1
Barber et al (2000)[3]	Peer review	Pre-post	Public: Learning disability hospital	-<br>Psychological support<br>• Group therapy using a person-centred therapeutic model, which aimed to run interactive and structured educative sessions, supplemented with supportive and non-confrontational discussions	SVA	4

Study	Peer review or grey lit	Study design	Setting/ sector	Type of intervention	Type of abuse*	No. of outcomes
Bloomfield & Dixon (2015)[4]	Grey lit.	Non-randomised comparative	Public: National Probation Service	*"Integrated Domestic Abuse Programme (IDAP) and Community Domestic Violence Programme (CDVP)"*<br>Perpetrator programme<br>• Cognitive behavioural programme, which challenged offenders' attitudes and beliefs to change their behaviour	DVA	2
Bowen (2010)[5-9]	Peer review	Quasi experimental	Public: local probation service	*"Domestic Violence Perpetrator Programme"*<br>Perpetrator programme<br>• Psychoeducational treatment programme in a group setting, focusing on definitions of DV, socialisation, impact of DV on partners and children, empathy, sexual respect and accountability and communication	DVA	9
Brady et al (2021)[10]	Peer review	RCT	Mixed: Third (human rights charity); unclear ('clinical setting')	-<br>Psychological support<br>• Narrative exposure therapy, involving co-construction of traumatic experiences and a therapy plan, to process traumatic memories. Psychoeducation was also delivered to all participants	SVA	5
Burton et al (1998)[11]	Grey lit.	Service evaluation	Third	*"Violence Prevention Programme (VPP)"*<br>Perpetrator programme<br>• Structured group sessions designed to assist men in understanding why they abuse, how they can change it, and constructing respectful relationships with women<br>Multi-service<br>• Telephone advice and support; making contact with, and regular follow-up of, partners of men on VPP; one-to-one support sessions; two weekly support groups;	DVA	2

Study	Peer review or grey lit	Study design	Setting/ sector	Type of intervention	Type of abuse*	No. of outcomes
				advocacy for women users; weekly liaison with the VPP; interagency work, including workshops and training; administration; and training for new volunteers. These also involve safety planning, housing / financial / legal advice and education (understanding domestic violence)		
Callaghan et al (2020)[12, 13]	Grey lit.	Pre-post	Third: DV charities / organisations	*"Make a Change"*<br>Perpetrator programme<br>• Group-based intervention for perpetrators<br>Psychological support<br>• Individual support for (ex)partners	DVA	13
Calvert et al (2015)[14]	Peer review	Pre-post	Public: Tertiary psychotherapy service	-<br>Psychological support<br>• Group cognitive analytic therapy	SVA (CSA)	6
Clarke & Llewelyn (1994)[15]	Peer review	Pre-post	Public: NHS	-<br>Psychological support<br>• Cognitive analytic therapy	SVA (CSA)	4
Clarke & Pearson (2000)[16]	Peer review	Pre-post	Public: NHS	-<br>Psychological support<br>• Cognitive analytic therapy	SVA (CSA)	4
Cook et al (1991)[17]	Peer review	Pre-post	Public	-<br>Perpetrator programme<br>• Group treatment for sex offenders	SVA	1
Co-ordinated Action Against Domestic	Grey lit.	Data-set / service evaluation	Third	-<br>Advocacy<br>• 14 IDVA services, with types of support	DVA	11

Study	Peer review or grey lit	Study design	Setting/ sector	Type of intervention	Type of abuse*	No. of outcomes
Abuse (2012)[18, 19]				including safety planning, support with MARAC, liaison and support with police, support with criminal court cases, liaison and support with probation, support with civil orders, support with housing, financial benefits advice and support, support with immigration, health and wellbeing advice and support, and support with children		
Coy and Kelly (2011)[20]	Grey lit.	Service evaluation	Third	-<br>Advocacy<br>• Four IDVAs in different settings (police station, hospital A&E department, a community based DV project, and a women only VAW organisation)	DVA	2
Dheensa & Penny (2019)[21]	Grey lit.	Service evaluation	Third	-<br>Advocacy<br>• Learning disability ISVA	SVA	1
Dobash et al (1999)[22]	Peer review	Non-randomised comparative	Public	*"IDVPP"* and *"CHANGE"*<br>Perpetrator programme<br>• Two perpetrator programmes, both focused on challenging group work	DVA	3
Ellis (2012)[23]	Peer review	Pre-post	Third?	*"The Butterfly Programme"*<br>Psychosocial support<br>• Group therapy	SVA (CSA)	6
Farrell & Buckley (1999)[24]	Peer review	Pre-post	Public	*"The Domestic Violence Unit"*<br>Specialist DV police teams<br>• Police officers dedicated to dealing with DV cases with specialist training.	DVA	5
Ferrari et al (2018)[25]	Peer review	Randomised controlled trial	Third	*"PATH"*<br>Advocacy<br>• Specialist psychological advocates providing	DVA	6

Study	Peer review or grey lit	Study design	Setting/ sector	Type of intervention	Type of abuse*	No. of outcomes
				1:1 support on top of regular advocacy provision		
Friendship et al. (2003)[26]	Peer review	Pre-post with retrospectively selected comparison group	Public: Prisons	*“SOTP”*<br>Perpetrator programme<br>• Two programmes based on the cognitive behavioural model, using group sessions	DVA	3
Garry & Munro (2020)[27-29]	Grey lit.	Service evaluation	Third	*“The Rape and Sexual Violence Project (RSVP) adult counselling service”*<br>Psychological support<br>• Face-to-face counselling. Clients can also access RSVPs other support services	SVA	1
Geoghegan-Fittall et al (2020)[30]	Grey lit.	Service evaluation	Third	*“The Croydon Drive Project”*<br>Perpetrator programme<br>• Direct and indirect interventions and activity disruption. Interventions may be singular or concurrent	DVA	3
Gilchrist et al (2021)[31, 32]	Peer review	Randomised controlled trial	Mixed: NHS and voluntary sector	*“ADVANCE”*<br>Perpetrator programme<br>• Manualised evidence-informed tailored intervention focusing on developing healthy relationships	DVA	14
Goosey et al (2017)[33]	Peer review	Randomised controlled trial	Mixed: community safety team, police, drug and alcohol service, Thames Valley Probation, Youth Offending	-<br>Multi-service<br>• Integrated cased management using the Domestic Abuse Service Coordination model, including support for victims, counselling for perpetrators, and follow up	DVA	1

Study	Peer review or grey lit	Study design	Setting/ sector	Type of intervention	Type of abuse*	No. of outcomes
			Service, Domestic Abuse Perpetrator Service, Berkshire Women's Aid, Bracknell Forest Homes	police visits		
Halliwell et al (2019)[34]	Peer review	Non-randomised comparative	Mixed: Public and Third	-<br>Advocacy<br>• A hospital based IDVA who provided support and advice, risk assessment and safety planning, referral, partnership work and training hospital staff	DVA	8
Hester et al (2019)[35-38]	Grey lit	Service evaluation using RCT design	Third	*"Drive"*<br>Perpetrator programme<br>• Intensive case management and multi-agency response. Uses disruption, support and behaviour change interventions alongside protective work<br>Advocacy<br>• One-to-one IDVA support for the associated victims-survivors	DVA	10
Hester & Westmarland (2005)[39]	Grey lit.	Service evaluation	Mixed: Third, public	-<br>Multiple / multi-service<br>• 27 domestic violence projects split in to seven packages (criminal and civil justice, protection and prevention, Black and other ethnic minorities, health, multi-service, education and rural work). A sub selection of the 27 that report relevant outcomes and use the included methodology were extracted, including: Bradford Staying Put, Hammersmith and Fulham Standing Together, Northampton Sunflower Centre,	DVA	17

Study	Peer review or grey lit	Study design	Setting/ sector	Type of intervention	Type of abuse*	No. of outcomes
				Cheshire and Taunton – use of photographic evidence, Croydon Domestic Violence Advocate Service, Northampton Sunflower Centre		
Howarth et al (2009)[98, 99]	Grey lit	Service evaluation	Third	-<br>Advocacy<br>• Seven IDVA services in a range of rurality's, sizes and age of service	DVA	13
Howarth and Robinson (2016)[100]	Peer review	Pre-post	Unclear	-<br>Advocacy<br>• Seven IDVA services. Support provided included safety planning, housing and accommodation, child-related support, access to justice systems, health and wellbeing, and welfare and immigration support	DVA	4
Karatzias et al (2016)[101]	Peer review	Pre-post	Public: NHS	*"Trauma Recovery and Empowerment Model"*<br>Psychological support<br>• Group psychotherapy using cognitive behavioural and skills training techniques	DVA<br>SVA<br>(CSA)	5
Kelley et al (2022)[40]	Peer review	Non-randomised comparative	Private: Privately owned prison	*"Principle-based correctional counselling"*<br>Perpetrator programme<br>• Classes including modules on building rapport, exploration of reality, exploration of thought, exploration of feelings/behaviour.<br>• All participants also participated in the required CBT based Sexual offender Treatment programme	SVA	6
Kelly et al	Grey lit.	Service	Public	*"Domestic Violence Matters"*	DVA	5

Study	Peer review or grey lit	Study design	Setting/ sector	Type of intervention	Type of abuse*	No. of outcomes
(1999)[41]		evaluation		Specialist domestic violence police teams<br>• Five support workers who worked alongside the police to support women where an arrest of a perpetrator occurred.		
Kelly & Westmarland (2015)[42]	Grey lit.	Service evaluation	Third	*"DVPP"*<br>Perpetrator programme<br>• Twelve Respect accredited DVPPs. All have manuals and involved group work.<br>Unclear – Women's support service<br>• Each perpetrator programme had an integrated women's support service, however no further details are provided.	DVA	7
Koppensteiner et al (2019)[43]	Grey lit.	Randomised controlled trial	Public: Police	*"Project 360"*<br>Specialist domestic violence police teams<br>• Engagement workers who contact victims and offer further assistance, including information on legal options, referrals to support services, and safety planning	DVA	1
Lindsay et al (2011)[44]	Peer review	Pre-post	Unclear	-<br>Perpetrator programme<br>• A sex offender treatment programme, including modules on disclosure, pathways into offending, cognitive distortions, childhood abuse, victim awareness, relapse prevention, and adaptive attachment	SVA	2
Lindsay et al. (1998)[45]	Peer review	Pre-post	Public: Psychology / psychiatry department	-<br>Perpetrator programme<br>• Group sessions focused on sex offending and cognitive change, including taking responsibility, victim awareness and behaviours consistent with offending	SVA	1

Study	Peer review or grey lit	Study design	Setting/ sector	Type of intervention	Type of abuse*	No. of outcomes
Lowe et al (2017)[46]	Peer review	Pre-post	Third	-<br>Psychological support<br>• Group therapy. Sessions used a person centred, psychoeducational approach with elements of cognitive behavioural therapy	SVA (CSA)	3
McConnell et al (2017)[47, 48]	Peer review	Pre-post	Third	*"Caring Dads Safer Children"*<br>Perpetrator programme<br>• Includes seven treatment targets: anger/hostility; family cohesion/ coparenting/domestic violence; perceptions of the child as a problem; use of corporal punishment and other aversive behaviours; positive and involved parent-child relationship; self-centeredness; and misuse of substances	DVA	4
McCracken & Deave (2012)[49]	Grey lit.	Service evaluation	Third	*"Caring Dads Cymru"*<br>Perpetrator programme<br>• Group work programme based on cognitive behavioural therapy and motivational models of intervention	DVA	4
Michie & Lindsay (2012)[50]	Peer review	Non-randomised comparative	Public: NHS	-<br>Perpetrator programme<br>• Cognitive behavioural therapy with an empathy component	SVA	1
Morgan et al (2019)[51, 52]	Peer review	Pre-post	Third	*"RADAR/ADAPT"*<br>Perpetrator programme<br>• A multiagency initiative that provides a tiered needs-led approach. It provides a programme for perpetrators and support for victims	DVA	4
Morrison &	Peer review	Pre-post	Public: Local	-	SVA	1

Study	Peer review or grey lit	Study design	Setting/ sector	Type of intervention	Type of abuse*	No. of outcomes
Treliving (2002)[53]			psychotherapy service	Psychological support<br>• Group therapy aiming to provide men with a safe space to explore their difficulties and the impact of childhood sexual abuse	(CSA)	
Murphy et al (2007)[54]	Peer review	Pre-post	Public: Intellectual disability services	-<br>Perpetrator programme<br>• Group cognitive behavioural therapy for sexually abusive men	SVA	4
Oasis (2016)[55, 56]	Grey lit	Service evaluation	Third	-<br>Multi-service<br>• Counselling (6 sessions plus more if required)<br>• Refuge: a 12-bed refuge providing temporary emergency accommodation. Staff provide emotional and practical support	DVA	3
Ormston et al (2016)[57]	Grey lit.	Service evaluation	Third	*"The Caledonian System"*<br>Perpetrator programme<br>• Perpetrator programme covering lifelong change, responsibility for and to self, relationships, sexual respect, men and women, and children and fathering.<br>Multi-service<br>• Women's service including safety planning, information, advice, and emotional support to (ex)partners	DVA	6
Payne et al (2007)[58]	Peer review	Pre-post	Public (assumed as authors are clinical psychologists)	-<br>Psychological support<br>• Client centred group therapy	DVA<br>SVA<br>(CSA)	3
Peckham et al (2007)[59]	Peer review	Pre-post	Public: local community intellectual disability service	-<br>Psychological support<br>• Survivors group, involving educating clients and carers about sexual abuse, and weekly	SVA	6

Study	Peer review or grey lit	Study design	Setting/ sector	Type of intervention	Type of abuse*	No. of outcomes
				sessions using drawings, videos, dolls and pictures		
Robinson (2003)[60]	Grey lit.	Service evaluation	Mixed: Public (police, criminal justice) and third sector	*"Women's Safety Unit"*<br>Multi-service<br>• Advocacy, specialist counselling, legal services, housing services, refuge provision, target hardening and collecting evidence	DVA<br>SVA	11
Robinson (2004)[61]	Grey lit.	Service evaluation	Mixed: Public (police, probation, health, WSU, social services, housing, health) and third sector	-<br>MARAC<br>• Multiagency meeting which delegates actions to workers. Agencies include police, social services, probation, health, and education (where relevant)	DVA	6
Robinson (2006)[62]	Peer review	Pre-post	Mixed: Police, probation, health, WSU, social services, housing, health, third sector	-<br>MARAC<br>• Monthly multiagency meetings to discuss high risk victims, to share information and take actions to reduce future harm.	DVA	6
Robjant et al (2017)[63]	Peer review	Pre-post	Third	-<br>Psychological support<br>• Narrative exposure therapy, a therapy specifically designed for victims of trauma	SVA	2
Rose et al (2012)[64]	Peer review	Pre-post	Public: Community Learning Disability Services	-<br>Perpetrator programme<br>• Group programme with topics including sex education, emotion recognition, cognitive distortions, motivation for offences, empathy and relapse prevention	SVA	4
Ross et al.	Peer review	Non-	Public: Magistrate's	-	DVA	4

Study	Peer review or grey lit	Study design	Setting/ sector	Type of intervention	Type of abuse*	No. of outcomes
(2022)[65]		randomised comparison	Court	Advocacy<br>• An IDVA service based at a Specialist Domestic Abuse Court		
Ryan et al (2005)[66]	Peer review	Randomised controlled trial	Public: NHS psychology departments	-<br>Psychological support<br>• Individual and group therapy, focused on experiences and consequences of CSA	SVA (CSA)	4
SafeLives[67]	Grey lit.	Dataset / service evaluation	Third	-<br>Refuge<br>• No information	DVA	12
SafeLives[68]	Grey lit.	Dataset / service evaluation	Third	-<br>MARAC<br>• No information	DVA	3
SafeLives[69, 70]	Grey lit.	Dataset / service evaluation	Third	-<br>Sexual violence services<br>• No information	SVA	19
SafeLives[71]	Grey lit.	Dataset / service evaluation	Third	-<br>Health services<br>• No information	DVA	12
Safelives[72]	Grey lit.	Dataset / service evaluation	Third	-<br>Helpline<br>• No information	DVA	11
SafeLives[73-77]	Grey lit.	Dataset / service evaluation	Third	-<br>IDVA<br>• No information	DVA	12
SafeLives[78-82]	Grey lit.	Dataset / service evaluation	Third	-<br>Outreach<br>• No information	DVA	12

Study	Peer review or grey lit	Study design	Setting/ sector	Type of intervention	Type of abuse*	No. of outcomes
Sharpe et al (2001)[83]	Peer review	Pre-post	Public: Psychological therapies service	*"The Male Childhood Sexual Abuse Group"*<br>Psychological support<br>• Group therapy, no further information	SVA (CSA)	2
Skyner & Waters (1999)[84]	Peer review	NR	Mixed: Public (probation service) and third sector (NSPCC)	*"The Domestic Violence Prevention Programme"*<br>Perpetrator programme<br>• A cognitive behavioural programme with a variety of learning styles. A parallel support package is provided to (ex)partners and their children throughout	DVA	4
Smith et al (1995)[85]	Peer review	Pre-post	Public: Specialised therapy unit	*"Breakfree"*<br>Psychological support<br>• Individual therapy, daytime drop in facility, telephone contact, out-of-hours paging service, limited befriending service and a 'time out' facility where a client can stay in a safe environment for 1-2 nights	SVA (CSA)	3
Smith et al (2015)[86]	Peer review	Pre-post	Third	*"Domestic Abuse Recovering Together (DART)"*<br>Psychological support<br>• Mother and child sessions, designed to strengthen their relationship and support recovery. Followed by separate peer group sessions	DVA	3
Stanley et al (2011)[87]	Grey lit.	Service evaluation	Mixed: Third and Public (NHS, City Council)	*"Strength to change"*<br>Perpetrator programme<br>• Individual and group sessions over 40 weeks. Advocacy and advice are offered where appropriate. Sessions include mindfulness techniques, cognitive approaches, counselling, behavioural techniques.<br>• A women's service is available for	DVA	4

Study	Peer review or grey lit	Study design	Setting/ sector	Type of intervention	Type of abuse*	No. of outcomes
				(ex)partners offering practical and emotional support		
Stanley et al (2021)[88]	Grey lit	Service evaluation	Third	*"VOICES"*<br>Multi-service<br>• Trauma informed approach. Practitioners were given a new assessment framework, training and planning tools. No information on the specific types of activities or interventions is reported	DVA	6
Stanley et al (2021)[89]	Grey lit	Service evaluation	Third	*"SafeLives Co-Designed Pilots (SLCDPs)"*<br>Multi-service<br>• A suite of interventions for survivors, their children and perpetrators, using a whole family approach. This included community IDVA support, complex needs IDVA support, step down and recovery groups, children and young peoples work and 'Engage', which worked with the whole family	DVA	5
Strang et al (2017)[90]	Peer review	Randomised controlled trial	Mixed: Public (police), third sector	*"CARA"*<br>Perpetrator programme<br>• A two-day offender workshop, focusing on raising awareness, and moving offenders from denial and minimisation to acceptance and responsibility of harm, and conflict resolution	DVA	3
Taylor-Dunn & Erol (2019)[91, 92]	Grey lit	Service evaluation	Third	-<br>Advocacy<br>• Intensive one-to-one IDVA support within an immediate to medium term time-frame, implementing individual support plans and risk assessments to reduce risk and encourage recovery support	DVA	5

Study	Peer review or grey lit	Study design	Setting/ sector	Type of intervention	Type of abuse*	No. of outcomes
The Institute for Public Safety, Crime & Justice, University of Northampton (2019)[93]	Grey lit.	Service evaluation	Third	*"SHE"*<br>Psychological support<br>• A 'minimum support service (MSS)' with regular telephone check-ins, with an emphasis on safety and wellbeing, and an 'integrated support service (ISS+)' offering a face-to-face person-centred programme<br>Perpetrator programme<br>• 'Evolve', a programme domestic violence perpetrator programme working towards Respect accreditation	DVA	5
Trevillon et al (2014)[94]	Peer review	Non-randomised comparative	Public: Community mental health team	-<br>Advocacy<br>• Domestic violence training for clinicians, domestic violence manual for clinicians, mental illness training for domestic violence advisors, direct referral pathway to domestic violence advocacy, provision of a domestic violence advocacy service, comprising emotional and practical support, domestic violence education, support groups, safety planning and legal/housing support	DVA	11
Vallely et al (2005)[95]	Grey lit.	Service evaluation	Mixed: Public (Crown Prosecution Service, Police, Magistrates' Court, Probation service, local authorities) and Third sector	-<br>Specialist domestic violence court<br>• The service included a dedicated sitting for pre-trial matters and sentencing, allocated weekly; specialist magistrates with domestic violence training, specially trained CPS lawyers, and an independent advocacy service	DVA	5
Webster (2015)[96]	Grey lit.	Service evaluation	Third	*"Kent and Medway IDVA"*<br>Advocacy	DVA	2

Study	Peer review or grey lit	Study design	Setting/ sector	Type of intervention	Type of abuse*	No. of outcomes
				• Up to 21 IDVAs working with domestic abuse victims. No further information.		
Westmarland & Alderson (2013)[97]	Peer review	Pre-post	Third	-<br>Psychological support<br>• Face-to-face counselling, following a woman-centred, empowerment model, with elements of cognitive behavioural therapy, goal setting and rational emotive behaviour therapy	SVA	1
Williamson & Boyle (2012)[98]	Peer review	Pre-post	Public: NHS	*"IDVA Advocacy Intervention Programme"*<br>Advocacy<br>• No details reported	DVA	2
Williamson & Hester (2009)[99]	Grey lit.	Service evaluation	Third	*"South Tyneside Domestic Abuse Perpetrator Programme"*<br>Perpetrator programme<br>• A 'pro-feminist broadly cognitive behavioural model combined with gender analysis' approach. Group and one-to-one sessions.	DVA	2
Women's Aid (2022)[100-105]	Grey lit.	Service evaluation	Third	*"No Woman Turned Away"*<br>Housing (specialist caseworkers)<br>• Caseworkers provide telephone and email support to women looking for a refuge space	DVA	1
Women's Resource Centre (2011b)[106]	Grey lit.	Service evaluation	Third	*"Women and Girls Network"*<br>Psychological support<br>Counselling using trauma therapy, cognitive behavioural therapy, and integrated person-centred and feminist counselling practice. The organisation also offers a telephone helpline, body therapies, group programmes and specialist support projects	DVA	1
Women's Resource	Grey lit.	Service evaluation	Third	*"Ashiana"*<br>Multi-service	DVA	11

Study	Peer review or grey lit	Study design	Setting/ sector	Type of intervention	Type of abuse*	No. of outcomes
Centre (2011a)[107]				• Refuge: Safe accommodation across three schemes for South Asian, Turkish and Iranian women. Residents can stay for up to 1 year.<br>• Outreach: To provide support and reduce repeat victimisation. Ashiana also runs a DV support group for women in the wider community, as well as an end of therapy support group.		

1. ADVA. and S.P. Associates., *REPAIR (Resolve to End the Perpetration of Abuse in Relationships): A Community- and Whole-family-based Intervention Programme Targeting Perpetrators of Domestic Violence and Abuse in Devon. An evaluation of a three-year Invest to Save (ISB) PROJECT*. 2009: Exeter.
2. Advance, *'A Place to go Like This': Breaking the cycle of harm for mothers involved in offending who are survivors of domestic abuse, and their children.* 2020.
3. Barber, M., R. Jenkins, and C. Jones, *A survivor's group for women who have a learning disability.* British Journal of Developmental Disabilities, 2000. **46**(90, Pt 1): p. 31-41.
4. Bloomfield, S. and L. Dixon, *An outcome evaluation of the integrated domestic abuse programme (IDAP) and community domestic violence programme (CDVP).* London: National Offender Management Service, 2015.
5. Bowen, E., *Evaluation of a community based domestic violence offender rehabilitation programme. Unpublished doctoral thesis*, in *Centre for Forensic and Family Psychology*. 2004, University of Birmingham.
6. Bowen, E., *Therapeutic Environment and Outcomes in a UK Domestic Violence Perpetrator Program.* Small Group Research, 2010. **41**(2): p. 198-220.
7. Bowen, E. and E. Gilchrist, *Predicting dropout of court-mandated treatment in a British sample of domestic violence offenders.* Psychology, Crime & Law, 2006. **12**(5): p. 573-587.
8. Bowen, E., E. Gilchrist, and A.R. Beech, *Change in treatment has no relationship with subsequent re-offending in UK domestic violence sample: A preliminary study.* International Journal of Offender Therapy and Comparative Criminology, 2008. **52**(5): p. 598-614.
9. Bowen, E., E.A. Gilchrist, and A.R. Beech, *An Examination of the Impact of Community-Based Rehabilitation on the Offending Behaviour of Male Domestic Violence Offenders and the Characteristics Associated with Recidivism.* Legal and Criminological Psychology, 2005. **10**(2): p. 189-209.
10. Brady, F., et al., *Narrative exposure therapy for survivors of human trafficking: feasibility randomised controlled trial.* BJPsych Open, 2021. **7**(6).
11. Burton, S., L. Regan, and L. Kelly, *Supporting women and challenging men: lessons from the Domestic Violence Intervention Project.* 1998.
12. Callaghan, J., et al., *Make a Change: An evaluation of the implementation of an early response intervention for those who have used abusive behaviours in their intimate relationships*. 2020.
13. Respect and Women's Aid Federation England, *Make a Change Executive Summary*.
14. Calvert, R., S. Kellett, and T. Hagan, *Group cognitive analytic therapy for female survivors of childhood sexual abuse.* British Journal of Clinical Psychology, 2015. **54**(4): p. 391-413.
15. Clarke, S. and S. Llewelyn, *Personal constructs of survivors of childhood sexual abuse receiving cognitive analytic therapy.* British Journal of Medical Psychology, 1994. **67(3)**: p. 273-289.
16. Clarke, S. and C. Pearson, *Personal constructs of male survivors of childhood sexual abuse receiving cognitive analytic therapy.* British Journal of Medical Psychology, 2000. **73(2)**: p. 169-177.
17. Cook, D.A., et al., *The Berkeley group: ten years' experience of a group for non-violent sex offenders.* The British Journal of Psychiatry, 1991. **158**(2): p. 238-243.

18. Co-Ordinated Action Against Domestic, A., *Insights into domestic abuse 1: a place of greater safety.* 2012.
19. Co-ordinated Action Against Domestic Abuse (CAADA), *Insights National Dataset 2011–12 Appendix to: A place of greater safety*. 2012.
20. Coy, M. and L. Kelly, *Islands in the stream: an evaluation of four London independent domestic violence advocacy schemes*. 2011.
21. Dheensa, S., G. Penny, and Safelink, *"In my way…" Evaluation Of Learning Disabilities Sexual Abuse Support Services*.
22. Dobash, R.P., et al., *A research evaluation of British programmes for violent men.* Journal of Social Policy, 1999. **28**(2): p. 205-233.
23. Ellis, F., *Rehabilitation programme for adult survivors of childhood sexual abuse.* Journal of public mental health, 2012. **11**(2): p. 88-92.
24. Farrell, G. and A. Buckley, *Evaluation of a UK police domestic violence unit using repeat victimisation as a performance indicator.* The Howard Journal of Criminal Justice, 1999. **38**(1): p. 42-53.
25. Ferrari, G., et al., *Psychological advocacy towards healing (PATH): A randomized controlled trial of a psychological intervention in a domestic violence service setting.* Plos One, 2018. **13**(11).
26. Friendship, C., R.E. Mann, and A.R. Beech, *Evaluation of a national prison-based treatment program for sexual offenders in England and Wales.* Journal of interpersonal violence, 2003. **18**(7): p. 744-759.
27. Garry, K. and P. Goodwin, *Adult Counselling Service Phase two evaluation report: year 1 2020-21*. 2021, Merida Associates.
28. Karen Garry and Ellie Munro, *Adult Counselling Service Interim evaluation | May 2019*. 2019.
29. Karen Garry and Ellie Munro, *Adult Counselling Service Evaluation report – phase one 2016-2020*. 2020.
30. Geoghegan-Fittall, S., C. Keeble, and D. Wunsch, *The Croydon Drive Project: A 2-year Evaluation Final Report*. 2020.
31. Gilchrist, E., et al., *Using the Behaviour Change Wheel to design an intervention for partner abusive men in drug and alcohol treatment.* Pilot and feasibility studies, 2021. **7**(1): p. 1-14.
32. Gilchrist, G., et al., *ADVANCE integrated group intervention to address both substance use and intimate partner abuse perpetration by men in substance use treatment: a feasibility randomised controlled trial.* BMC public health, 2021. **21**(1): p. 1-20.
33. Goosey, J., L. Sherman, and P. Neyroud, *Integrated case management of repeated intimate partner violence: A randomized, controlled trial.* Cambridge Journal of Evidence-Based Policing, 2017. **1**(2): p. 174-189.
34. Halliwell, G., et al., *Cry for health: a quantitative evaluation of a hospital-based advocacy intervention for domestic violence and abuse.* Bmc Health Services Research, 2019. **19**(1).
35. Hester, M., et al., *Evaluation of Year 2 of the Drive Project – A Pilot to Address High Risk Perpetrators of Domestic Abuse*. 2019.
36. Hester, M., et al., *Evaluation of the Drive Project – a pilot to address high-risk perpetrators of domestic abuse Year 1 Feasibility Study*. 2017.
37. Hester, M., et al., *Evaluation of the Drive Project – A Three-year Pilot to Address High-risk, High-harm Perpetrators of Domestic Abuse*. 2019.
38. Hester, M., et al., *Evaluation of the Drive Project – A Three-year Pilot to Address High-risk, High-harm Perpetrators of Domestic Abuse EXECUTIVE SUMMARY*. 2020.
39. Hester, M. and N. Westmarland, *Tackling domestic violence: effective interventions and approaches*. 2005: Home Office Research, Development and Statistics Directorate.

40. Kelley, T.M., et al., *The Efficacy of Principle-Based Correctional Counseling for Improving the Self-Control and Mental Health of People Incarcerated for Sexual Violence.* Violence Against Women, 2022. **28**(2): p. 573-592.
41. Kelly, L., *Domestic Violence Matters: an evaluation of a development project*. 1999.
42. Kelly, L. and N. Westmarland, *DOMESTIC VIOLENCE PERPETRATOR PROGRAMMES STEPS TOWARDS CHANGE: Project Mirabal Final Report*. 2015.
43. Koppensteiner, M.F., J. Matheson, and R. Plugor, *Project 360: An intervention to address victim-police engagement in repeat domestic violence cases*. 2017.
44. Lindsay, W.R., et al., *Comparing offenders against women and offenders against children on treatment outcome in offenders with intellectual disability.* Journal of Applied Research in Intellectual Disabilities, 2011. **24**(4): p. 361-369.
45. Lindsay, W.R., et al., *The treatment of six men with a learning disability convicted of sex offences with children.* British Journal of Clinical Psychology, 1998. **37**: p. 83-98.
46. Lowe, M., et al., *CORE assessment of adult survivors abused as children: A NAPAC group therapy evaluation.* Counselling & Psychotherapy Research, 2017. **17**(1): p. 71-79.
47. McConnell, N., et al., *Caring dads safer children*. 2014.
48. McConnell, N., M. Barnard, and J. Taylor, *Caring Dads Safer Children: Families' Perspectives on an Intervention for Maltreating Fathers.* Psychology of Violence, 2017. **7**(3): p. 406-416.
49. McCracken, K. and T. Deave, *Evaluation of the Caring Dads Cymru programme. Final report.* 2012.
50. Michie, A.M. and W.R. Lindsay, *A treatment component designed to enhance empathy in sex offenders with an intellectual disability.* The British Journal of Forensic Practice, 2012. **14**(1): p. 40-48.
51. Morgan, S. and J. Parkes, *The Hampshire Domestic Abuse Prevention Partnership 2016-2018: process and outcomes evaluation.* 2018.
52. Morgan, S.A., B.M.S. McCausland, and J. Parkes, *Baseline characteristics and outcomes of the main perpetrator programme within the Hampshire Domestic Abuse Prevention Partnership, UK: A mixed methods study.* Plos One, 2019. **14**(7).
53. Morrison, A. and L. Treliving, *Evaluation of outcome in a dynamically orientated group for adult males who have been sexually abused in childhood.* British Journal of Psychotherapy, 2002. **19**: p. 59-75.
54. Murphy, G., et al., *Cognitive-behavioural treatment for men with intellectual disabilities and sexually abusive behaviour: A pilot study.* Journal of Intellectual Disability Research, 2007. **51(11)**: p. 902-912.
55. Oasis. *Impact report 2013-2014*. 2014; Available from: https://issuu.com/kristinagwynne/docs/oasis_annual_report_2013-2014-web/1.
56. Oasis, *Impact Report 2015-2016.* 2016.
57. Ormston, R., C. Mullholland, and L. Setterfield, *Caledonian system evaluation: analysis of a programme for tackling domestic abuse in Scotland*. 2016, Edinburgh: Scotland. Scottish Government Social Research. vi, 88.
58. Payne, A., H. Liebling-Kalifani, and S. Joseph, *Client-centred group therapy for survivors of interpersonal trauma: A pilot investigation.* Counselling & Psychotherapy Research, 2007. **7**(2): p. 100-105.
59. Peckham, N.G., S. Howlett, and A. Corbett, *Evaluating a survivors group pilot for a women with significant intellectual disabilities who have been sexually abused.* Journal of Applied Research in Intellectual Disabilities, 2007. **20**(4): p. 308-322.

60. Robinson, A.L., *The Cardiff Women's Safety Unit: A multi-agency approach to domestic violence.* Final Evaluation Report. Cardiff University: School of Social Sciences.(Available online at http://www. cf. ac. uk/socsi/whoswho/robinson. html), 2003.
61. Robinson, A.L., *Domestic Violence MARACs (Multi-Agency Risk Assesment Conferences) for Very High-risk Victims in Cardiff, Wales: A Process and Outcome Evaluation*. 2004.
62. Robinson, A.L., *Reducing repeat victimization among high-risk victims of domestic violence: The benefits of a coordinated community response in Cardiff, Wales.* Violence Against Women, 2006. **12(8)**: p. 761-788.
63. Robjant, K., J. Roberts, and C. Katona, *Treating posttraumatic stress disorder in female victims of trafficking using narrative exposure therapy: A retrospective audit.* Frontiers in Psychiatry, 2017. **8**.
64. Rose, J., et al., *A sex offender treatment group for men with intellectual disabilities in a community setting.* The British Journal of Forensic Practice, 2012.
65. Ross, J., J. Sebire, and H. Strang, *Tracking Repeat Victimisation After Domestic Abuse Cases Are Heard With and Without Independent Domestic Violence Advisors (IDVAs) in an English Magistrate's Court.* Cambridge Journal of Evidence-Based Policing, 2022: p. 1-15.
66. Ryan, M., et al., *A prospective study of the effectiveness of group and individual psychotherapy for women CSA survivors.* Psychology and Psychotherapy: Theory, Research and Practice, 2005. **78(4)**: p. 465-480.
67. SafeLives, *Insights refuge England and Wales dataset 2015-18 Adult refuge services*. 2018.
68. SafeLives, *Defining Marac Outcomes National Pilot*. 2012.
69. SafeLives, *Insights England and Wales dataset 2018-19 Sexual violence services* 2019.
70. SafeLives, *Insights SV dataset 2020-21 Sexual violence (SV) services*. 2021.
71. SafeLives, *Insights health England and Wales dataset 2015-2018 Adults health-based services*. 2018.
72. SafeLives, *Insights helpline England and Wales dataset 2014-17 Adult helpline services*. 2017.
73. SafeLives, *Insights Idva national dataset 2012–13 Adult independent domestic violence advisor (Idva) services*. 2015.
74. SafeLives, *Insights Idva national dataset 2013–14 Adult independent domestic violence advisor (Idva) services*. 2015.
75. SafeLives, *Insights Idva England and Wales dataset 2016-17 Adult independent domestic violence advisor (Idva) services*. 2017.
76. SafeLives, *Insights Idva England and Wales dataset 2018-19 Adult Independent domestic violence advisor (Idva) services*. 2019.
77. SafeLives, *Insights Idva dataset 2020-21 Adult Independent domestic violence advisor (Idva) services*. 2021.
78. SafeLives, *Insights outreach national dataset 2012–13 Adult outreach services*. 2015.
79. SafeLives, *Insights outreach national dataset 2013–14 Adult outreach services.* 2015.
80. SafeLives, *Insights outreach England and Wales dataset 2016-17 Adult outreach services.* 2017.
81. SafeLives, *Insights outreach England and Wales dataset 2018-19 Adult outreach services.* 2019.
82. SafeLives, *Insights outreach dataset 2020-21 Adult outreach services*. 2021.
83. Sharpe, J., et al., *Group analytic therapy for male survivors of childhood sexual abuse.* Group Analysis, 2001. **34**: p. 195-209.
84. Skyner, D.R. and J. Waters, *Working with perpetrators of domestic violence to protect women and children: a partnership between Cheshire Probation Service and the NSPCC.* Child Abuse Review: Journal of the British Association for the Study and Prevention of Child Abuse and Neglect, 1999. **8**(1): p. 46-54.

85. Smith, D., et al., *Adults with a history of child sexual abuse: Evaluation of a pilot therapy service.* British Medical Journal, 1995. **310(6988)**: p. 1175-1178.
86. Smith, E., et al., *Strengthening the mother-child relationship following domestic abuse: Service evaluation.* Child Abuse Review, 2015. **24**(4): p. 261-273.
87. Stanley, N., et al., *An evaluation of a new initiative for male perpetrators of domestic violence* 2011.
88. Stanley, N. and a. et, *Roadmap evaluation: final report - VOICES*. 2021, Bristol: Women's Aid
SafeLives. 279.
89. Stanley, N. and a. et, *Roadmap evaluation: final report - SLCDPs*. 2021, Bristol: Women's Aid
SafeLives. 279.
90. Strang, H., et al., *Reducing the harm of intimate partner violence: Randomized controlled trial of the Hampshire Constabulary CARA Experiment.* Cambridge Journal of Evidence-Based Policing, 2017. **1**(2): p. 160-173.
91. Holly Taylor-Dunn and Rosie Erol, *Evaluation of SaferPlaces' Independent Domestic Violence Advisor Services.* 2019.
92. Taylor-Dunn, H. and R. Erol, *Improving the safety and well-being of domestic abuse survivors: The role of a specialist organisation in supporting the work of Independent Domestic Violence Advisors.* Crime Prevention and Community Safety, 2021. **23**(2): p. 115-136.
93. Eve; The Institute for Public Safety, C.J., *2019*. Evaluation of SHE.
94. Trevillion, K., et al., *Linking abuse and recovery through advocacy: An observational study.* Epidemiology and Psychiatric Sciences, 2014. **23**(1): p. 99-113.
95. Vallely, C., *Evaluation of domestic violence pilot sites at Caerphilly (Gwent) and Croydon 2004/05.* 2005.
96. Webster, R., *Kent and Medway Independent Domestic Violence Advisor Service Impact Evaluation FINAL REPORT*. 2015.
97. Westmarland, N. and S. Alderson, *The health, mental health, and well-being benefits of rape crisis counseling.* Journal of Interpersonal Violence, 2013. **28**(17): p. 3265-3282.
98. Williamson, D. and A. Boyle, *Results of the domestic violence advocacy program at addenbrookes hospital.* Academic Emergency Medicine, 2012. **19**: p. 741.
99. Williamson, E. and M. Hester, *Evaluation of the South Tyneside Domestic Abuse Perpetrator Programme (STDAPP) 2006-2008.* University of Bristol, 2009.
100. Women's Aid, *Nowhere To Turn, 2022: Findings from the sixth year of the No Woman Turned Away project*. 2022.
101. Miles, C. and K. Smith, *Nowhere to turn, 2018: findings from the second year of the No Woman Turned away project*. 2018, Bristol: Women's Aid. 48.
102. Women's Aid, *NOWHERE TO TURN Findings from the first year of the No Woman Turned Away project*. 2017.
103. Women's Aid, *NOWHERE TO TURN Findings from the third year of the No Woman Turned Away project*. 2019.
104. Women's Aid, *NOWHERE TO TURN Findings from the fourth year of the No Woman Turned Away project*. 2020, Women's Aid Federation of England.
105. Women's Aid, *NOWHERE TO TURN 2021 Findings from the fifth year of the No Woman Turned Away project*. 2021.
106. Women's Resource, C., *Women and Girls Network: the WGN empowerment model - redressing negative individual and social impacts of gendered violence through holistic counselling.* 2011.

107. Women's Resource, C., *Ashiana Network: journey towards safety.* 2011.
