# Supplementary material for: Trends in outcomes used to measure the effectiveness of UK-based support interventions and services targeted at adults with experience of domestic and sexual violence and abuse: a scoping review
Source: BMJ Open. 2024 Apr 30;14(4):e074452. doi: 10.1136/bmjopen-2023-074452 (PMC11086554; doi:10.1136/bmjopen-2023-074452)
Supplement: Supplementary data [file bmjopen-2023-074452supp005.pdf]

Appendix 5: Outcome measures by domain and subdomain

Category	Domain	Subdomain	Measure	N
**Health**	**Mental health**	**Depression**		**16**
			Beck Depression Inventory	6
			Hospital Anxiety and Depression Scale - Depression	4
			Patient Health Questionnaire	3
			PROMIS depression Short Form	1
			Single item – “Is the client experiencing depression?”	1
			Single item – reduction in feeling depressed	1
		**Anxiety**		**10**
			Hospital Anxiety and Depression Scale - Anxiety	4
			Generalized Anxiety Disorder Scale	3
			PROMIS Anxiety Short Form	1
			Spiel State Anxiety	1
			Single item – “Is the client experiencing anxiety?”	1
		**Trauma**		**12**
			Primary care PTSD screen	2
			Impact of Event Scale	2
			PTSD Checklist for DSM-5 (PCL-5)	1
			PTSD Symptom Scale	1
			Posttraumatic Diagnostic Scale	1
			Post-traumatic Stress Disorder Scale	1
			Changes in Outlook Questionnaire	1
			The Clinician Administered PTSD Scale	1
			PTSD Checklist (PCL-C)	1
			Single item – “I have “flashbacks” about what happened”	1
		**General mental health**		**4**
			General Health Questionnaire (GHQ-28)	2
			Single item - "Current mental health rating"	1

Category	Domain	Subdomain	Measure	N
			No information – reported outcome is "Improved mental health"	1
		**Psychological symptoms**		**19**
			The Clinical Outcomes in Routine Evaluation - Outcome Measure (CORE-OM)	5
			The Symptom Checklist 90 revised (SCL-90R)	4
			Brief Symptom Inventory	2
			Jehu Beliefs Inventory	2
			The Shutdown Dissociation Scale (Shu-Dis)	1
			Dissociative Experiences Scale (DES)	1
			Belief Inventory	1
			Social Activity and Distress Scale	1
			Delusions, Symptoms and States Inventory	1
			Single item – “Is the client experiencing emotional instability?”	1
		**Anger**		**2**
			The Novaco Anger Scale	1
			PROMIS Anger Short Form	1
		**Understanding feelings**$		2
			Three Principles Inventory$	1
			Single item - "Understanding feelings better"$	1
		**Self-Harm / Suicide**$		**5**
			Single item – reduction in experiencing suicidal thoughts or feelings$	1
			Single item – reduction in self harm$	1
			Single item – “Is the client experiencing self-harm?” $	1
			Single item – “Is the client experiencing suicidal thoughts?” $	1
			Single item – “Is the client experiencing suicidal behaviours?” $	1
		**Healthcare utilisation / resource use**$		**5**
			Number / percent of participants using healthcare resources (e.g., A&E, GP, psychiatrist)$	3
			Number / percent of participants using community health and social service resources (e.g., health visitor, drug worker) $	1
			Days of admission$	1
		**Mental wellbeing**$		**4**

Category	Domain	Subdomain	Measure	N
			Short Warwick–Edinburgh Mental Wellbeing Scale (SWEMWBS) $	4
				2
		**Self-care$**	Single item - "Is the client experiencing trouble sleeping?" $	1
			Single item - "Is the client experiencing problems with eating?" $	1
				**3**
		**Quality of life$**	Short Form 12 (SF12) $	2
			Manchester Short Assessment of Quality of Life$	1
		**Health, mental health and wellbeing$**		**1**
			Taking Back Control Tool$	1
	**Physical health**	**Self-reported physical health**		**8**
			EQ-5D	3
			Visual analogue scale for physical health	2
			Single item – "Current physical health (poor/fair/good/very good/excellent)"	1
			No information – reported outcome is "Improved physical health (DV specific)"	1
			No information – reported outcome is "Improved physical health (not DV specific)"	1
		**Alcohol and drug use**		**5**
			Alcohol Use Disorders Identification Test (AUDIT) $	1
			Single item – "Current drug use (excessive/high/moderate/low/none)" $	1
			Single item – "Current alcohol use (excessive/high/moderate/low/none)" $	1
			Single item – "Alcohol problem (yes/no)" $	1
			Single item – "Drug problem (yes/no)" $	1
		**Healthcare utilisation / resource use$**		**5**
			Number / percent of participants using healthcare resources (e.g., A&E, GP, psychiatrist)$	3
			Number / percent of participants using community health and social service resources (e.g., health visitor, drug worker) $	1
			Days of admission$	1
		**Quality of life$**		**3**
			Short Form 12 (SF12) $	2

Category	Domain	Subdomain	Measure	N
			Manchester Short Assessment of Quality of Life$	1
		**Health, mental health and wellbeing$**		**1**
			Taking Back Control Tool$	1
	**Wellbeing**	**Wellbeing**		**7**
			Outcome Star	1
			Adult wellbeing scale	1
			Benefits of Therapy scale	1
			Personal Outcomes and Wellbeing Record (POWeR)	1
			Victim survey (perceived wellbeing - safety and wellbeing)	1
			Wellbeing survey (9 domains)	1
			CORE monitoring form	1
			Perceived Severity of Problems/Concerns Questionnaire	1
		**Social ties/ relationships**		**5**
			Social Inclusion Scale$	1
			Inventory of interpersonal problems 32 (IIP-32) $	1
			Single item - "Current social and community support (no social contact / negative social contacts / some positive contacts / majority positive contacts / all positive)" $	1
			No information – reported outcome is "Improved relationship with family"$	1
			No information – reported outcome is "Improved social support"$	1
		**Understanding feelings**		**2**
			Three Principles Inventory$	1
			Single item - "Understanding feelings better"$	1
		**Mental wellbeing$**		**4**
			Short Warwick–Edinburgh Mental Wellbeing Scale (SWEMWBS) $	4
		**Self-care$**		**2**

Category	Domain	Subdomain	Measure	N
			Single item - "Is the client experiencing trouble sleeping?" $	1
			Single item - "Is the client experiencing problems with eating?" $	1
		**Quality of life$**		**3**
			Short Form 12 (SF12) $	2
			Manchester Short Assessment of Quality of Life$	1
		**Health, mental health and wellbeing$**		**1**
			Taking Back Control Tool$	1
**Behavioural**	**Behavioural**	**Challenging behaviour**		**1**
			Challenging Behaviour Inventory	1
		**Self-Harm / Suicide$**		**5**
			Single item – reduction in experiencing suicidal thoughts or feelings$	1
			Single item – reduction in self harm$	1
			Single item – "Is the client experiencing self-harm?" $	1
			Single item – "Is the client experiencing suicidal thoughts?" $	1
			Single item – "Is the client experiencing suicidal behaviours?" $	1
		**Alcohol and drug use$**		**5**
			Alcohol Use Disorders Identification Test (AUDIT) $	1
			Single item – "Current drug use (excessive/high/moderate/low/none)" $	1
			Single item – "Current alcohol use (excessive/high/moderate/low/none)" $	1
			Single item – "Alcohol problem (yes/no)" $	1
			Single item – "Drug problem (yes/no)" $	1
**Empowerment**	**Empowerment**	**Self-esteem**		**12**
			Rosenberg's self-esteem scale	7
			Culture Free Self Esteem Inventory	2
			The Self Concept Questionnaire	1
			Self-esteem Assessment Tool	1
			No information – reported outcome is "Improved self-esteem"	1
		**Coping and resilience**		**3**
			Resilience Research Centre Adult Resilience Measure (RRC-ARM)	1

Category	Domain	Subdomain	Measure	N
			Cope and Recover Questionnaire	1
			No information – reported outcome is "Improved coping"	1
		**Locus of control**		**1**
			Review of Personal Effectiveness with Locus of Control	
		**Empowerment**		**5**
			The Empowerment Star	2
			Measure of Victim Empowerment Related to Safety Scale (MOVERS)	1
			Coping and confidence outcome measure	2
		**Assertiveness**		**1**
			Assertiveness Questionnaire	1
		**Knowledge**		**1**
			Goal Attainment Scale	1
		**Social ties/ relationships**[$]		**5**
			Social Inclusion Scale[$]	1
			Inventory of interpersonal problems 32 (IIP-32) [$]	1
			Single item - "Current social and community support (no social contact / negative social contacts / some positive contacts / majority positive contacts / all positive)" [$]	1
			No information – reported outcome is "Improved relationship with family"[$]	1
			No information – reported outcome is "Improved social support"[$]	1
		**Understanding feelings**[$]		**2**
			Three Principles Inventory[$]	1
			Single item - "Understanding feelings better"[$]	1
				2
			No information – reported outcome "Independence"	1
		**Independence**		
			No information – reported outcome "Increased likelihood of employment "	1
		**Awareness of support**		1

Category	Domain	Subdomain	Measure	N
			Number of criminal justice contacts	1
**Socio-economic circumstances**	**Socio-economic circumstances**	**Employment/ education**		**5**
			Single item - "Employment status (FT employed/PT employed/unemployed/caring for children/family)"	1
			Single item - "Employment status (paid employment/voluntary work/education/retired)"	1
			Single item - "Returned to/begun employment or education"	1
			Single item - "Education, employment and training (none / occasional / some sustained / sustained / sustained and fulfilling)"	1
			No information – reported outcome "Increased likelihood of employment"[$]	1
		**Finances**		**3**
			No information – reported outcome "Financial savings"	1
			Single item - "Perception of income (high/comfortable/managing with treats/managing with occasional treats/essentials only/struggling)"	1
			Single item - "Current financial situation (significant financial problems/being reliant on others/unable to pay for essentials/managing essentials but nothing left over/comfortably managing and no financial concerns)"	1
		**Housing**		**4**
			No information – reported outcome "Access to housing"	1
			Single item – "Current housing (homeless / transitory / unsuitable / temporary / secure)"	1
			Single item – "Number of women accommodated in suitable refuge spaces"	1
			Number / percent of participants using supported accommodation resources (e.g., weeks spent in refuge / staffed accommodation / B&B, boarding house or hotel / homeless and living with friends or family)	1
				**1**
				5
		**Social ties/ relationships**[$]	Social Inclusion Scale[$]	1
			Inventory of interpersonal problems 32 (IIP-32) [$]	1
			Single item - "Current social and community support (no social contact /	1

Category	Domain	Subdomain	Measure	N
			negative social contacts / some positive contacts / majority positive contacts / all positive)" $	
			No information – reported outcome is "Improved relationship with family"$	1
			No information – reported outcome is "Improved social support"$	1
**Changing perpetrator behaviour**	**Changing perpetrator behaviour**	**Anger**		**1**
			The Novaco Anger Scale	1
		**Perpetrator needs**		**1**
			Distribution of needs	1
		**Attitudes to women / violence**		**20**
			Inventory of Beliefs About Wife Beating	1
			Questionnaire on Attitudes Consistent with Sexual Offending (QACSO)	3
			Socio-Sexual Knowledge and Attitudes Assessment – Revised (SSKAAT-R)	1
			The Intimate Partner Violence Responsibility Attribution Scale (IPVRAS)	1
			URICA-DV Readiness to change	1
			Propensity for Abusiveness Scale (PAS)	2
			Paulhus Deception Scale (PDS)	1
			Interpersonal Reactivity Index (IRI)	1
			Sexual Attitudes and Knowledge Scale	1
			Victim Empathy Scale-Adapted	1
			Sexual Offenders Self Appraisal Scale	1
			Treatment motivation Questionnaire	1
			Interpersonal Dependency Inventory	1
			Attitudes Toward Exhibitionism Questionnaire	1
			No information - "Attitudes to the offence"	1
			No information - "Attitudes towards women"	1
			No information - "Acceptance of responsibility"	1
		**Desirable responding**		**3**
			Balanced Inventory of Desirable Reporting	3
		**Locus of control / self-control**		**6**
			Multidimensional Locus of Control Scale	1
			Rotter's Locus of Control Scale	1
			Brief self-control scale (BSCS)	1
			Low self-control scale (LSCS)	1

Category	Domain	Subdomain	Measure	N
			Levenson Locus of Control	1
			The Nowicki-Strickland Locus of Control Scale	1
		**Reasons for violence**		**2**
			Number of reasons for violence given	1
			Number of participants reporting each of 7 potential reasons for abuse	1
**Victim-perpetrator relationship**	**Victim-perpetrator relationship**	**Victim-perpetrator relationship**		**12**
			The Communication Patterns Questionnaire-Short Form (CPQ-SF)	1
			Respectful communication	1
			Awareness of self and others	1
			Attitude to partner (self-partner correlation)	1
			Single item - "Relationship status"	1
			Single item - "Hopes for the relationship"	1
			Single item - "Living circumstances (with perpetrator / not living together / intermittently living together)	6
**Parenting**	**Parenting**	**Parenting**		**7**
			Parent Empowerment and Efficacy Measure (PEEM)	1
			Camberwell Assessment of Need for Mothers (Short version)	1
			Parenting Stress Index	2
			Parental Acceptance-Rejection Questionnaire (PARQ)	2
			The Parental Locus of Control Scale	1
**DSVA**	**Experience of DSVA**	**Impact of abuse**		**2**
			Number of participants experiencing each of 18 potential impacts of abuse	1
			Mean number of impacts of abuse	
		**Indicators of ongoing abuse**		**5**
			Additional MARACs/Reappearance at MARAC	3
			Other evidence of abuse (e.g. harassing texts)	1
			Cases reopened	1
		**Perceived safety**		**7**
			Safety Behaviour Checklist	1
			Single item - "Partner fearful (1= never to 4=often)"	1

Category	Domain	Subdomain	Measure	N
			Single item - "Feeling of safety (much safer / somewhat safer / no change / less safe)"	1
			Safety (6 items)	2
			Single item - "How safe do you feel? (not safe / a little unsafe / somewhat safe / very safe)	1
			No information - reported outcome is "increased safety"	1
			No information - reported outcome is "avoidance of forced marriage"	1
		**Revictimization**		**4**
			Average number of repeat victims per month	1
			Number / percent of repeat victims	1
			Number / percent of women reporting repeat victimisation (for IPA)	1
			Number / percent of women disclosing further incidents of violence to IDVA	1
		**Presence of abuse**$		**46**
			Severity of Abuse Grid - presence: Number / percent of people responding yes to the presence of abuse (reported for physical abuse, sexual abuse, harassment and stalking and jealous and controlling behaviour) $	10*
			Severity of Abuse Grid - cessation: Number / percent of people reporting no abuse$	11*
			Severity of Abuse Grid - reduction in presence: Number / percent of people reporting a reduction of abuse (reported for physical abuse, sexual abuse, harassment and stalking and jealous and controlling behaviour) $	1
			Severity of Abuse Grid - multiple types: Number / percent of people reporting multiple types of abuse$	7*
			Number / percent of participants experience concurrent domestic abuse as well as sexual abuse$	1
			Number / percent of participants who have been subjected to adult sex offences$	1
			Number / percent of men abusing each month$	1
			Space for Action Survey (% responding yes to 12 indicators) $	1
			Physical and Sexual Violence Survey (% responding yes to 7 indicators) $	1
			Harassment and other abusive acts survey (% responding yes to 6 indicators) $	1
			Fathering survey / using children against a partner (% responding yes to 5 indicators) $	2

Category	Domain	Subdomain	Measure	N
			Social media perpetration / victimisation survey$	1
			Composite Abuse Scale$	3
			Abusive Behaviour Inventory$	2
			Controlling Behaviour Inventory$	3
		**Number of abusive incidents or behaviours$**		**4**
			Mean number of abusive incidents per man$	1
			Mean number of abusive behaviours reported by participants$	1
			Number / percent of participants experiencing between 0 and 7 behaviours (reported for emotional, physical and sexual behaviours) $	1
			IMPACT monitoring toolkit (reported for emotional, physical, and sexual behaviours) $	1
		**Severity of abuse$**		**29**
			Severity of Abuse Grid - severity: Number / percent of participants reporting high / moderate / standard severity (reported for physical abuse, sexual abuse, harassment and stalking and jealous and controlling behaviour) $	8*,±
			Severity of Abuse Grid - reduction in severity: Number / percent of participants reporting a reduction of high / moderate / standard / unknown severity (reported for physical abuse, sexual abuse, harassment and stalking and jealous and controlling behaviour) $	1
			Severity of Abuse Grid – escalation in severity: Number / percent of participants reporting escalation in severity of abuse$	2
			Severity of Abuse Grid - severity: Number / percent of participants reporting any form of severe abuse$	2
			Severity of Abuse Grid - high severity: Number / percent of participants reporting severe abuse (reported for physical abuse, sexual abuse, harassment and stalking and jealous and controlling behaviour) $	1
			Severity of Abuse Grid - severity: Number / percent of participants reporting multiple types of abuse that are high severity$	6*
			Severity of Abuse Grid - severity: Number / percent of participants reporting at least one type of abuse that is high severity$	6*
			Cambridge Crime Harm Index$	3
		**Frequency of abuse$**		**14**

Category	Domain	Subdomain	Measure	N
			Severity of Abuse Grid - frequency: Number / percent of participants reporting high / moderate / standard / unknown frequency (reported for physical abuse, sexual abuse, harassment and stalking and jealous and controlling behaviour) $	5*
			Severity of Abuse Grid - frequency escalation: Number / percent of participants reporting an escalation in frequency$	6*
			Frequency of abusive behaviours (scale 1 = never -3 = often) $	1
			Frequency rating of past violence (none (1) -> frequent (10) $	1
			Frequency rating of current violence (none (1) -> frequent (10) $	1
		**Duration of abuse$**		**1**
			Number / percent of victims with incidents recorded over a period longer than 12 months$	1
		**Severity and frequency of abuse$**		**13**
			Severity of Abuse Grid - severity and frequency escalation: Number / percent of participants reporting any escalation in severity or frequency of abuse$	7*
			Severity of Abuse Grid - severity and frequency escalation: Number / percent of participants reporting at least one form of abuse that is high severity and escalating in frequency or severity$	6*
		**Risk$**		**18**
			Spousal Assault Risk Assessment$	2
			DASH risk (high/med/standard) $	2
			Drive-DASH (mean score) $	1
			DASH risk (number with 1-14 assessments who had a decrease in score) $	1
			Change in risk in perpetrators (measurement tool unclear) $	1
			Weighted change in risk in perpetrators (measurement tool unclear) $	1
			IDVA perception of change in risk (do not know / increased / limited or no reduction / moderate reduction / significant reduction) $	1
			IDVA rated reduction in risk (percentage reporting 'some reduction in risk') $	1
			IDVA perception of sustainability of change in risk (Do not know / short term / medium term / long term / risk is permanently eliminated) $	1
			Men's perception of risk of using violence again (never/not sure/possibly) $	1

Category	Domain	Subdomain	Measure	N
			Risk factors - Perpetrators' crimogenic behaviour and aggravating problems (frequency and percent of 7 factors) $	1
			Risk factors - Specific features of abuse (frequency and percent of 10 factors) $	1
			Risk factors - Victims' appraisals of threat (frequency and percent of 3 factors) $	1
			Risk factors - Factors relating to children (frequency and percent of 3 factors) $	1
			Risk factors - Factors relating to victim (frequency and percent of 4 factors) $	1
			Probability of none / standard / moderate / and high abuse (reported for physical abuse / sexual abuse / harassment and stalking / jealous and controlling behaviour) $	1
		**Police incidents**$		**39**
			Average / total number of police incidents$	1
			Average / total number of DV related police incidents$	2
			Average / total number of incidents recorded by the police per month$	1
			Average number of DV complaints per month$	1
			Average number of arrests made per month$	1
			Average number of victims refusing to make a complaint per month$	1
			Average number of mental health related DV complaints per month$	1
			Average number of alcohol related DV complaints per month$	1
			Average number of drug related DV complaints per month$	1
			Average number of concern for children reports per month$	1
			Number / percent of participants that committed DV related police incidents$	1
			Number / percent of participants that committed (unspecified) police incidents$	1
			Number / percent of participants with (unspecified) arrests$	1
			Number / percent of calls to domestic incidents$	1
			Number / percent of repeat calls to domestic incidents$	1
			Number / percent of repeat calls to domestic violence incidents (number with 2 incidents, 3 incidents, 4 incidents… 14 incidents) $	1
			Number / percent of DV incidents where the perpetrator was not present$	1
			Number / percent of DV incidents that were crimed$	1
			Number / percent of DV incidents that led to arrests$	2

Category	Domain	Subdomain	Measure	N
			Number / percent of incidents with an arrest where a statement is given$	1
			Number / percent of incidents with an arrest and a statement is given where it is then retracted$	1
			Number / percent of crimes where perpetrator was charged/summonsed$	1
			Number / percent of police incidents before and after a MARAC$	1
			Number / percent of high / medium / low severity police incidents before and after a MARAC$	1
			Number / percent of women experiencing no further post-MARAC incidents of violence or abuse$	1
			Number / percent of women experiencing no further post-MARAC police complaints or call outs$	2
			Number / percent of women experiencing no further post-MARAC police complaints$	2
			Number / percent of women experiencing no further post-MARAC police call outs$	2
			Number / percent of women experiencing either additional post-MARAC police complaints or call outs$	1
			Number / percent of women experiencing both additional post-MARAC police complaints and call outs$	1
			Percent change in total calls to domestic incidents$	1
			Percent change in total calls to domestic incidents (as proportion of all calls) $	1
			Frequency of police call outs (never / once / 2-5 times / 6-10 times) $	1
			Cost of police incidents$	1
	**Perpetration of DSVA**	**Reoffending**		**20**
			Number / percent with proven reoffending (DV, core violence or any offence)	1
			Number / percent who reoffended (police incidents)	1
			Number / percent who committed further sex offences	2
			Number / percent who reoffended (no further details)	1
			Number / percent with repeat prosecution for violence against partner	1
			Number / percent with reconviction for another sexual offence	1
			Number / percent with reconviction for another violence offence	1
			Number / percent with reconviction for any offence	2

Category	Domain	Subdomain	Measure	N
			Number / percent linked to another crime (suspect or charged)	1
			Number / percent linked to another DVA crime (suspect or charged)	1
			Frequency of re-arrests for DVA	1
			Prevalence of re-arrests for DVA	1
			Number / percent of crimes that that were DVA related	1
			Number of incidents of DV whilst on the programme	1
			Number of incidents of non-DV crime whilst on the programme	1
			Number / percent of partners reporting subsequent violence	1
			Number / percent of partners reporting 'frequent violence' at follow up	1
			Time to reoffend	1
		**Criminal justice outcomes**		**16**
			Average number of people charged per month	1
			Average number of cases proceeding through court per month	1
			Average number of days between arrest and case finalisation	1
			Number / percent of cases discontinued	2
			Number / percent of cases bound over / other / found not guilty or absolute discharge / convicted	1
			Number / percent of victims retracting	1
			Number / percent of trials that went ahead on scheduled date	1
			Number / percent of trials that received a conviction	1
			Number / percent of charges that received a conviction	1
			Number / percent of guilty and not guilty pleas	1
			Number / percent of defendants who had the following sentences: conditional discharge / financial penalty / custody / community punishment order / community rehabilitation order	1
			Number / percent of cases with the following convictions: custodial sentence / non-custodial sentence / conditional discharge	1
			Number / percent of evidence types in CPS files (victim statement / police statement / defendant interview / witness statement / medical statement / case exhibits / 999 tape / forensic)	1
			Improvements specific to the 'CPS Criteria for Success'	1

Category	Domain	Subdomain	Measure	N
			Average monetary penalties (fines and compensation)	1
		**Presence of abuse**$		**46**
			Severity of Abuse Grid - presence: Number / percent of people responding yes to the presence of abuse (reported for physical abuse, sexual abuse, harassment and stalking and jealous and controlling behaviour) $	10*
			Severity of Abuse Grid - cessation: Number / percent of people reporting no abuse$	11*
			Severity of Abuse Grid - reduction in presence: Number / percent of people reporting a reduction of abuse (reported for physical abuse, sexual abuse, harassment and stalking and jealous and controlling behaviour) $	1
			Severity of Abuse Grid - multiple types: Number / percent of people reporting multiple types of abuse$	7*
			Number / percent of participants experience concurrent domestic abuse as well as sexual abuse$	1
			Number / percent of participants who have been subjected to adult sex offences$	1
			Number / percent of men abusing each month$	1
			Space for Action Survey (% responding yes to 12 indicators) $	1
			Physical and Sexual Violence Survey (% responding yes to 7 indicators) $	1
			Harassment and other abusive acts survey (% responding yes to 6 indicators) $	1
			Fathering survey / using children against a partner (% responding yes to 5 indicators) $	2
			Social media perpetration / victimisation survey$	1
			Composite Abuse Scale$	3
			Abusive Behaviour Inventory$	2
			Controlling Behaviour Inventory$	3
		**Number of abusive incidents or behaviours**$		**4**
			Mean number of abusive incidents per man$	1
			Mean number of abusive behaviours reported by participants$	1
			Number / percent of participants experiencing between 0 and 7 behaviours (reported for emotional, physical and sexual behaviours) $	1
			IMPACT monitoring toolkit (reported for emotional, physical, and sexual behaviours) $	1
		**Severity of abuse**$		**29**

Category	Domain	Subdomain	Measure	N
			Severity of Abuse Grid - severity: Number / percent of participants reporting high / moderate / standard severity (reported for physical abuse, sexual abuse, harassment and stalking and jealous and controlling behaviour) $	8*,±
			Severity of Abuse Grid - reduction in severity: Number / percent of participants reporting a reduction of high / moderate / standard / unknown severity (reported for physical abuse, sexual abuse, harassment and stalking and jealous and controlling behaviour) $	1
			Severity of Abuse Grid – escalation in severity: Number / percent of participants reporting escalation in severity of abuse$	2
			Severity of Abuse Grid - severity: Number / percent of participants reporting any form of severe abuse$	2
			Severity of Abuse Grid - high severity: Number / percent of participants reporting severe abuse (reported for physical abuse, sexual abuse, harassment and stalking and jealous and controlling behaviour) $	1
			Severity of Abuse Grid - severity: Number / percent of participants reporting multiple types of abuse that are high severity$	6*
			Severity of Abuse Grid - severity: Number / percent of participants reporting at least one type of abuse that is high severity$	6*
			Cambridge Crime Harm Index$	3
		**Frequency of abuse$**		**14**
			Severity of Abuse Grid - frequency: Number / percent of participants reporting high / moderate / standard / unknown frequency (reported for physical abuse, sexual abuse, harassment and stalking and jealous and controlling behaviour) $	5*
			Severity of Abuse Grid - frequency escalation: Number / percent of participants reporting an escalation in frequency$	6*
			Frequency of abusive behaviours (scale 1 = never -3 = often) $	1
			Frequency rating of past violence (none (1) -> frequent (10) $	1
			Frequency rating of current violence (none (1) -> frequent (10) $	1
		**Duration of abuse$**		**1**
			Number / percent of victims with incidents recorded over a period longer than 12 months$	1
		**Severity and frequency of abuse$**		**13**
			Severity of Abuse Grid - severity and frequency escalation: Number / percent of participants reporting any escalation in severity or frequency of abuse$	7*
			Severity of Abuse Grid - severity and frequency escalation: Number / percent	6*

Category	Domain	Subdomain	Measure	N
			of participants reporting at least one form of abuse that is high severity and escalating in frequency or severity$	
		**Risk$**		**18**
			Spousal Assault Risk Assessment$	2
			DASH risk (high/med/standard) $	2
			Drive-DASH (mean score) $	1
			DASH risk (number with 1-14 assessments who had a decrease in score) $	1
			Change in risk in perpetrators (measurement tool unclear) $	1
			Weighted change in risk in perpetrators (measurement tool unclear) $	1
			IDVA perception of change in risk (do not know / increased / limited or no reduction / moderate reduction / significant reduction) $	1
			IDVA rated reduction in risk (percentage reporting 'some reduction in risk') $	1
			IDVA perception of sustainability of change in risk (Do not know / short term / medium term / long term / risk is permanently eliminated) $	1
			Men's perception of risk of using violence again (never/not sure/possibly) $	1
			Risk factors - Perpetrators' crimogenic behaviour and aggravating problems (frequency and percent of 7 factors) $	1
			Risk factors - Specific features of abuse (frequency and percent of 10 factors) $	1
			Risk factors - Victims' appraisals of threat (frequency and percent of 3 factors) $	1
			Risk factors - Factors relating to children (frequency and percent of 3 factors) $	1
			Risk factors - Factors relating to victim (frequency and percent of 4 factors) $	1
			Probability of none / standard / moderate / and high abuse (reported for physical abuse / sexual abuse / harassment and stalking / jealous and controlling behaviour) $	1
		**Police incidents$**		**39**
			Average / total number of police incidents$	1
			Average / total number of DV related police incidents$	2
			Average / total number of incidents recorded by the police per month$	1
			Average number of DV complaints per month$	1
			Average number of arrests made per month$	1

Category	Domain	Subdomain	Measure	N
			Average number of victims refusing to make a complaint per month$	1
			Average number of mental health related DV complaints per month$	1
			Average number of alcohol related DV complaints per month$	1
			Average number of drug related DV complaints per month$	1
			Average number of concern for children reports per month$	1
			Number / percent of participants that committed DV related police incidents$	1
			Number / percent of participants that committed (unspecified) police incidents$	1
			Number / percent of participants with (unspecified) arrests$	1
			Number / percent of calls to domestic incidents$	1
			Number / percent of repeat calls to domestic incidents$	1
			Number / percent of repeat calls to domestic violence incidents (number with 2 incidents, 3 incidents, 4 incidents… 14 incidents) $	1
			Number / percent of DV incidents where the perpetrator was not present$	1
			Number / percent of DV incidents that were crimed$	1
			Number / percent of DV incidents that led to arrests$	2
			Number / percent of incidents with an arrest where a statement is given$	1
			Number / percent of incidents with an arrest and a statement is given where it is then retracted$	1
			Number / percent of crimes where perpetrator was charged/summonsed$	1
			Number / percent of police incidents before and after a MARAC$	1
			Number / percent of high / medium / low severity police incidents before and after a MARAC$	1
			Number / percent of women experiencing no further post-MARAC incidents of violence or abuse$	1
			Number / percent of women experiencing no further post-MARAC police complaints or call outs$	2
			Number / percent of women experiencing no further post-MARAC police complaints$	2
			Number / percent of women experiencing no further post-MARAC police call outs$	2
			Number / percent of women experiencing either additional post-MARAC police complaints or call outs	1

Category	Domain	Subdomain	Measure	N
			Number / percent of women experiencing both additional post-MARAC police complaints and call outs	1
			Percent change in total calls to domestic incidents	1
			Percent change in total calls to domestic incidents (as proportion of all calls)	1
			Frequency of police call outs (never / once / 2-5 times / 6-10 times)	1
			Cost of police incidents	1

Note: Severity of Abuse Grid: there was a diverse approach to how it was used by different authors in the literature, , therefore we decided to split outcomes according to the various ways in which it was operationalised, rather than just reporting 'Severity of Abuse Grid' as an outcome.

* Note: Some studies do not explicitly specify using the Severity of Abuse Grid, but this has been presumed because the wording used when reporting outcomes matches exactly.

± Note: One study uses the outcome measure to assess perpetrators using abusive behaviours, rather than victim-survivor experiences of abuse.

$ Note: These outcomes and subdomains appear in more than one domain.
