# Supplementary material for: Trends in outcomes used to measure the effectiveness of UK-based support interventions and services targeted at adults with experience of domestic and sexual violence and abuse: a scoping review
Source: BMJ Open. 2024 Apr 30;14(4):e074452. doi: 10.1136/bmjopen-2023-074452 (PMC11086554; doi:10.1136/bmjopen-2023-074452)
Supplement: Supplementary data [file bmjopen-2023-074452supp006.pdf]

Frequency of outcomes by outcome domain, and the number of studies reporting one or more outcomes per outcome domains by intervention type, source of study, violence type and sector

		Outcome measure domain										
		Mental health	Physical health	Wellbeing	Behavioural	Empowerment	Socio-economic circumstances	Changing perpetrator beliefs and skills	Victim-perpetrator relationship	Parenting	Experience of DSVA	Perpetration of DSVA
Number of studies reporting at least one outcome		29	11	19	5	21	9	14	10	5	42	46
Number of total outcome measures		85	22	25	11	32	17	33	12	7	189	201
Number of total unique outcome measures		52	16	21	11	23	17	28	7	5	106	119
**Type of intervention**	**No. of studies**											
Perpetrator programme	27	2	1	2	1	1	1	14	4	3	14	21
Victim support:												
Psychological support	21	17	1	7	2	10	2	0	0	1	1	0
Advocacy	14	5	4	4	0	4	2	0	2	1	12	11
Multi-service	11	4	4	4	1	5	1	0	0	0	8	5
Housing	2	0	0	0	0	0	1	0	1	0	1	1
MARAC	3	0	0	0	0	0	0	0	0	0	3	2
Specialist domestic violence police teams	3	0	0	1	0	0	0	0	0	0	2	2
Specialist domestic violence court	1	0	0	0	0	0	0	0	0	0	0	1
Sexual violence service	1	1	1	1	1	1	1	0	0	0	1	1
Helpline	1	0	0	0	0	0	0	0	1	0	1	1
Outreach	1	0	0	0	0	0	0	0	1	0	1	1
Health	1	0	0	0	0	0	1	0	1	0	1	1
Other / Unclear	1	0	0	0	0	0	0	0	0	0	1	1

**Source**												
Peer reviewed	41	22	6	10	3	11	3	9	1	3	13	19
Grey literature	39	7	5	9	2	10	6	5	9	2	29	27
**Type of violence**												
Domestic violence and abuse	53	11	9	12	2	10	6	8	10	5	40	41
Sexual violence and abuse	15	7	2	4	2	5	1	6	0	0	1	4
Domestic and sexual violence and abuse	3	2	0	0	0	1	0	0	0	0	1	1
Childhood sexual abuse	9	9	0	3	1	5	2	0	0	0	0	0
**Sector**												
Third	39	12	7	14	3	12	7	5	9	4	27	23
Public	26	13	2	3	1	8	2	5	0	1	5	10
Mixed	12	3	2	1	1	0	0	2	1	0	9	11
Private	1	1	0	1	0	1	0	1	0	0	0	0
Unclear	2	0	0	0	0	0	0	1	0	0	1	2

Note: Sixteen studies reported an outcome that fits into multiple domains therefore some outcomes are represented by multiple domains. Additionally, 38 studies reported an outcome that was categorised as both the experience of DSVA and perpetration of DSVA, because the specific outcome could represent either domain depending on who is completing the outcome measure.
Note: Two studies have outcome domains that are represented in multiple intervention types (perpetrator programme, unclear/other and advocacy), because they report a perpetrator programme and an associated women's service, but it is unclear which intervention/service the outcomes were measured as part of.
Note: The number of studies for the different intervention/service types is more than the total number of studies (80) because 7 studies reported a perpetrator programme and a women's service.
