# Supplementary material for: Trends in outcomes used to measure the effectiveness of UK-based support interventions and services targeted at adults with experience of domestic and sexual violence and abuse: a scoping review
Source: BMJ Open. 2024 Apr 30;14(4):e074452. doi: 10.1136/bmjopen-2023-074452 (PMC11086554; doi:10.1136/bmjopen-2023-074452)
Supplement: Supplementary data [file bmjopen-2023-074452supp007.pdf]

Figure 4: Temporal trends in outcome domains reported in DVSA evaluations, 1991 to 2022

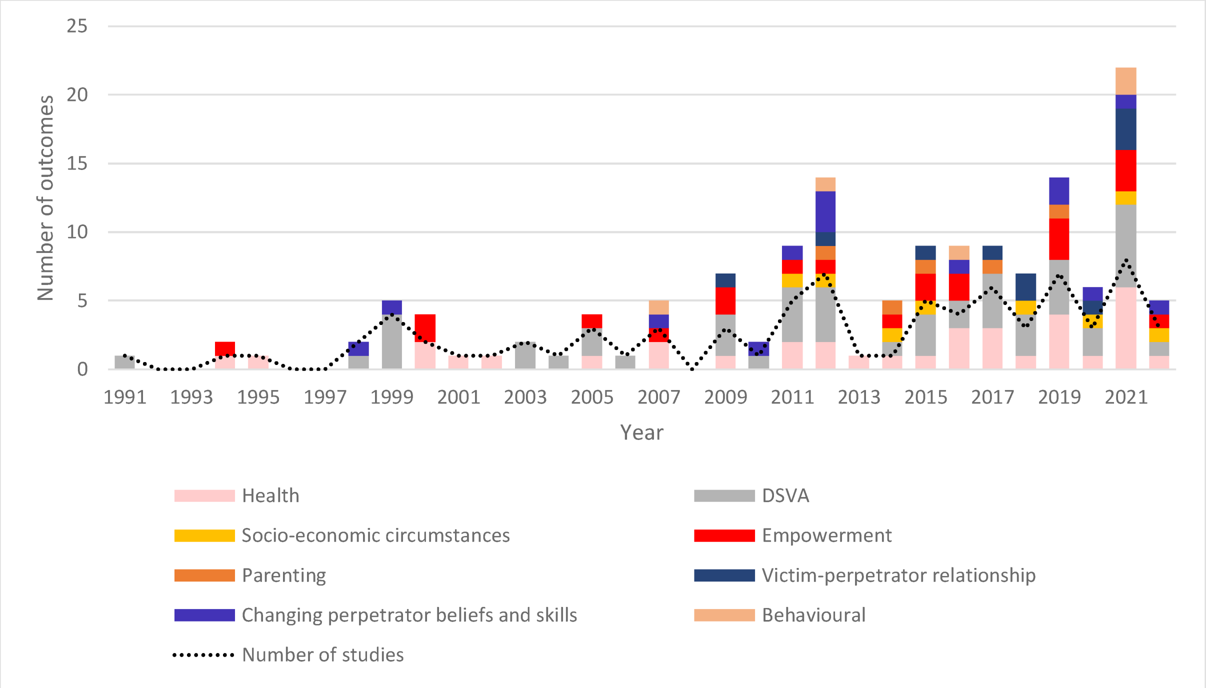

Note: For ease of interpretation, mental health, physical health and wellbeing domains have been combined into one health domain, and the experience of DSVA and perpetration of DSVA domains have been combined into a single 'DSVA' domain.
Note: Sixteen studies reported an outcome that crosses two or more domains therefore some outcomes are represented by multiple domains. Additionally, 38 studies reported an outcome that was categorised as both the experience of DSVA and perpetration of DSVA, because the specific outcome could represent either domain depending on who is completing the outcome measure.
