# Supplementary material for: Trends in outcomes used to measure the effectiveness of UK-based support interventions and services targeted at adults with experience of domestic and sexual violence and abuse: a scoping review
Source: BMJ Open. 2024 Apr 30;14(4):e074452. doi: 10.1136/bmjopen-2023-074452 (PMC11086554; doi:10.1136/bmjopen-2023-074452)
Supplement: Author's manuscript [file bmjopen-2023-074452.draft_revisions.pdf]

BMJ Open is committed to open peer review. As part of this commitment we make the peer review history of every article we publish publicly available.

When an article is published we post the peer reviewers' comments and the authors' responses online. We also post the versions of the paper that were used during peer review. These are the versions that the peer review comments apply to.

The versions of the paper that follow are the versions that were submitted during the peer review process. They are not the versions of record or the final published versions. They should not be cited or distributed as the published version of this manuscript.

BMJ Open is an open access journal and the full, final, typeset and author-corrected version of record of the manuscript is available on our site with no access controls, subscription charges or pay-per-view fees (http://bmjopen.bmj.com).

If you have any questions on BMJ Open's open peer review process please email info.bmjopen@bmj.com

BMJ Open

Trends in outcomes used to measure the effectiveness of UK-based support interventions and services targeted at adults with experience of domestic and sexual violence and abuse: A scoping review.

Journal:	*BMJ Open*
Manuscript ID	bmjopen-2023-074452
Article Type:	Original research
Date Submitted by the Author:	11-Apr-2023
Complete List of Authors:	Carlisle, Sophie ; King's College London, Health Service and Population Research<br>Bunce, Annie; City University of London<br>Prina, Matthew; Newcastle University, Population Health Sciences Institute<br>Cook, Elizabeth; City University of London, Violence and Society Centre<br>Barbosa, Estela; City University of London,<br>McManus, Sally; City University of London; National Centre for Social Research<br>Feder, Gene; University of Bristol, Community based medicine<br>Lewis, Natalia; University of Bristol, Centre for Academic Primary Care, Population Health Sciences, Bristol Medical School; National Institute for Health Research Collaboration for Leadership in Applied Health Research and Care North Thames at Bart's Health NHS Trust, Queen Mary University of London, Centre for Primary Care and Public Health
Keywords:	Systematic Review, PUBLIC HEALTH, MENTAL HEALTH

SCHOLARONE™ Manuscripts

I, the Submitting Author has the right to grant and does grant on behalf of all authors of the Work (as defined in the below author licence), an exclusive licence and/or a non-exclusive licence for contributions from authors who are: i) UK Crown employees; ii) where BMJ has agreed a CC-BY licence shall apply, and/or iii) in accordance with the terms applicable for US Federal Government officers or employees acting as part of their official duties; on a worldwide, perpetual, irrevocable, royalty-free basis to BMJ Publishing Group Ltd ("BMJ") its licensees and where the relevant Journal is co-owned by BMJ to the co-owners of the Journal, to publish the Work in this journal and any other BMJ products and to exploit all rights, as set out in our licence.

The Submitting Author accepts and understands that any supply made under these terms is made by BMJ to the Submitting Author unless you are acting as an employee on behalf of your employer or a postgraduate student of an affiliated institution which is paying any applicable article publishing charge ("APC") for Open Access articles. Where the Submitting Author wishes to make the Work available on an Open Access basis (and intends to pay the relevant APC), the terms of reuse of such Open Access shall be governed by a Creative Commons licence – details of these licences and which Creative Commons licence will apply to this Work are set out in our licence referred to above.

Other than as permitted in any relevant BMJ Author's Self Archiving Policies, I confirm this Work has not been accepted for publication elsewhere, is not being considered for publication elsewhere and does not duplicate material already published. I confirm all authors consent to publication of this Work and authorise the granting of this licence.

For peer review only

Trends in outcomes used to measure the effectiveness of UK-based support interventions and services targeted at adults with experience of domestic and sexual violence and abuse: A scoping review.

Sophie Carlisle[1], Annie Bunce[3], Matthew Prina[5], Elizabeth Cook[3], Estela Barbosa[3], Sally McManus[3,4], Gene Feder[2], Natalia Lewis[2]

For peer review only

Abstract

Objectives: In the UK, a range of support services and interventions are available to people who have experienced or perpetrated domestic and sexual violence and abuse (DSVA). However, it is currently not clear which outcomes and outcome measures are used to assess their effectiveness. The objective of this review is to summarise, map and identify trends in outcome measures in evaluations and reports of DSVA services and interventions in the UK.

Design: Scoping review

Data sources: MEDLINE, EMBASE, PsycINFO, Social Policy and Practice, ASSIA, IBSS, Sociological abstracts and SSCI electronic databases were searched from inception until 21st June 2022. Grey literature sources were identified and searched.

Eligibility: We included randomised controlled trials, non-randomised comparative studies, pre-post studies and service evaluations, with at least one outcome relating to the effectiveness of the support intervention or service for people who have experienced and/or perpetrated DSVA. Outcomes had to be assessed at baseline and at least one more time-point, or compared to a comparison group.

Charting methods: Outcome measures were extracted, iteratively thematically grouped into categories, domains and subdomains, and trends were explored.

For peer review only - http://bmjopen.bmj.com/site/about/guidelines.xhtml

Results: 80 studies reporting 87 interventions or services were included. A total of 426 outcome measures were extracted, of which 200 were used more than once. The most commonly reported subdomain was DSVA perpetration, and cessation of abuse according to the Severity of Abuse Grid was the most common individual outcome. Analysis of temporal trends showed that the number of studies and outcomes used has increased since the 1990s.

Conclusions: Our findings highlight inconsistencies between studies in outcome measurement. The increase in the number of studies and variety of measures suggests that as evaluation of these services and interventions matures, there is an increased need for a core of common, reliable metrics to aid comparability.

Protocol registration: https://osf.io/frh2e

Article summary

Strengths and limitations of this study

- This scoping review adhered to PRISMA guidelines to robustly and systematically map effectiveness outcome measures used in evaluations of support interventions and services for people who have experienced and/or perpetrated DSVA.
- It included an extensive grey literature search to be comprehensive and inclusive.
- An advisory group consisting of representatives from six third sector organisations has been involved in the development of the review.
- The review has not included services or interventions for children who have experienced DSVA, interventions aimed at staff training, and is limited to the UK.
- Further research is urgently required to work towards agreement on which outcome measures should be prioritised and adopted, whilst also avoiding burden on systems and people going through it.

For peer review only - http://bmjopen.bmj.com/site/about/guidelines.xhtml

Key words

Scoping review, domestic abuse, sexual violence, perpetrator programme, outcomes

Author affiliations

1 Department of Health Service and Population Research, Institute of Psychiatry, Psychology and Neuroscience, King's College London, De Crespigny Park, London, UK

2 Centre for Academic Primary Care, Bristol Medical School, University of Bristol, Bristol BS8 2PS, UK

3 Violence and Society Centre, City, University of London, London, UK

4 National Centre for Social Research, London, UK

5 Population Health Sciences Institute, Faculty of Medical Sciences, Newcastle University, Newcastle, NE4 5PL, UK

Corresponding author

Sophie Carlisle: Department of Health Service and Population Research, Institute of Psychiatry, Psychology and Neuroscience, King's College London, De Crespigny Park, London, UK. Sophie.r.carlisle@kcl.ac.uk

Author Contributions

All authors contributed to the conceptualisation of the review. SC produced the first draft, carried out searches, screening, extraction and analysis. AB was the second reviewer. All co-authors reviewed the manuscript and approved the final version.

Funding statement

This research was supported by the UK Prevention Research Partnership (Violence, Health and Society; MR-VO49879/1), which is funded by the British Heart Foundation, Chief

For peer review only - http://bmjopen.bmj.com/site/about/guidelines.xhtml

Scientist Office of the Scottish Government Health and Social Care Directorates, Engineering and Physical Sciences Research Council, Economic and Social Research Council, Health and Social Care Research and Development Division (Welsh Government), Medical Research Council, National Institute for Health and Care Research, Natural Environment Research Council, Public Health Agency (Northern Ireland), The Health Foundation, and Wellcome. The views expressed in this Article are those of the authors and not necessarily those of the UK Prevention Research Partnership or any other funder. The funder had no input in the design of the protocol or conduct of the review.

Competing interests statement

None declared.

Data availability statement

No data are available.

Word Count:

For peer review only

For peer review only - http://bmjopen.bmj.com/site/about/guidelines.xhtml

Introduction

Domestic and sexual violence and abuse (DSVA; comprising range of different behaviours and experiences, see Appendix 1) represents a significant international public health issue [1-3]. Approximately 1 in 3 women and 1 in 7 men in England and Wales have experienced domestic violence and abuse in their lifetime [4]. Globally, over 35% of women have experienced either physical and/or sexual intimate partner violence or non-partner sexual violence [5]. The UK does not currently routinely collect data on perpetrators, however studies have found that the prevalence of DSVA perpetration ranges from 11.6-80.0% depending on country, gender and definition of DSVA [6, 7]. Perpetrators are typically male [8] and are often serial perpetrators with more than one victim [9, 10]. The impacts of DSVA are profound and diverse. As well as visible and immediate consequences such as physical injury or death, DSVA has been associated with longer-term physical health [11-17] and mental health problems [18-21]. Other areas impacted by DSVA include homelessness [22], access to finances, employment and education [23], and increased demand on the criminal justice system [24], social services [25], and police [26].

Given the wide-ranging impacts of DSVA, the support options available to people who have experienced it are diverse. Interventions and services include refuges, outreach, counselling, financial or legal advice, helplines and advocates. In the UK, these are often provided by third sector organisations (see Appendix 1), but can also be located in the public and private sectors. Previous systematic reviews of the global evidence have found benefits of such interventions, including housing interventions improving mental health, perceived safety and stress [27], economic interventions reducing levels of domestic violence and increasing empowerment [28], advocacy interventions improving quality of life and depression [29], and psychological therapies reducing depression and anxiety [30]. Service evaluations and annual reports produced by local DSVA organisations have also found associations between service use and improved perceptions of safety, quality of life, health and wellbeing, and

For peer review only - http://bmjopen.bmj.com/site/about/guidelines.xhtml

confidence [31-35]. Finally, reviews of perpetrator programmes (i.e., interventions targeted at people who use violence) have provided some evidence of reductions in the perpetration and experience of abusive behaviours, although highlighted methodological issues [36, 37].

Despite existing reviews identifying potential benefits of specific types of support services and interventions, there is limited understanding of which outcomes and outcome measures are currently being used both within and across UK-based support services and interventions when assessing their effectiveness [38, 39]. The specific outcome measures that are being used appear to vary, making it difficult to compare and synthesise the overall evidence [40]. Identifying the most commonly utilised outcome measures will allow for meta-analysis, leading to more rigorous evaluations of the literature, and may also guide interventions and services on which outcome measures to use. Scoping reviews present an opportunity to map the evidence, and are ideal for summarising heterogenous evidence [41, 42]. This scoping review aims to summarise and map trends in the outcome measures being used to evaluate support interventions and services for DSVA.

Objectives

Specific objectives of this scoping review include:

1. Identify and thematically group outcome measures that have been used to assess the effectiveness of support interventions and services for adults who have experienced and/or perpetrated DSVA.
2. Identify which outcome measures have been used most frequently.
3. Explore whether the outcome measures used differ depending on; the type of intervention or service in terms of service user and type of support, the type of violence the intervention or service relates to, the sector/setting, the source of the study (peer review or grey literature), and over time.

For peer review only

Review question

What outcomes relating to effectiveness have been measured in intervention studies and service evaluations of UK-based support programmes for adults who have experienced and/or perpetrated DSVA?

Methods

Protocol and registration

The protocol was developed according to the Preferred Reporting Items for Systematic Reviews and Meta-Analysis Extension for Scoping Reviews (PRISMA-ScR) [43] and published on Open Science Framework: https://osf.io/frh2e [44]. The PRISMA-ScR checklist is available in Appendix 3.

Eligibility criteria

We included any UK-based secondary or tertiary prevention support services and interventions aimed at adults who had experienced or perpetrated DSVA. Randomised controlled trials (RCTs), non-randomised comparative trials, pre-post studies and service evaluations that reported effectiveness outcomes at two or more time-points or made comparisons to another group were included, so that cause and effect could be inferred. Perpetrator programmes were considered for several reasons. Firstly, including them allows the measurement of DSVA without placing the burden on people who have experienced DSVA to change someone else's behaviour. This ties in to the UK government's new perpetrator strategy [45], which intends to place the onus on perpetrators changing their behaviour, alongside the recovery of those who have experienced DSVA. Finally, perpetrator programmes provide support for perpetrators to change their behaviour, and many offer associated support to (ex)partners or referral to appropriate support. Details of the full eligibility criteria can be found in Table 1.

For peer review only - http://bmjopen.bmj.com/site/about/guidelines.xhtml

Table 1: PICO Inclusion and Exclusion Criteria

	Inclusion	Exclusion
Population	• Adults (16 years or older) who have experienced DSVA.<br>• Adults (16 years or older) who have perpetrated DSVA.	• Children<br>• Adults who have not experienced and/or perpetrated DSVA
Intervention (and service)	• Any secondary or tertiary prevention intervention and/or service for DSVA e.g., housing (e.g., refuges, housing workers, resettlements), Advocacy (e.g., Independent Domestic Violence Advisers/Advocates (IDVAs), Independent Sexual Violence Advisers/Advocates (ISVAs)), outreach, open access (e.g., helplines, drop-ins, online chats), psychological support (e.g., support groups, counselling, befriending), legal support, financial support, multi-agency risk assessment conferences (MARACs), and police-based services.<br>• Perpetrator programmes (see Appendix 1).<br>• Entry to the intervention had to be determined by the either the experience of DSVA (for victim-survivor support interventions) or perpetrating DSVA (for perpetrator programmes).	• Pharmacological<br>• Primary prevention<br>• Not primarily aimed at people with experience of DSVA
Comparison	• RCTs:<br>○ Another type of included intervention or service.<br>○ Usual care<br>○ No intervention or service<br>• Pre-post designs:<br>○ Before and after the intervention or service	• No comparison
Outcome	• Any outcomes used to measure effectiveness of DSVA support services and interventions.	• Those not focussed on the effectiveness of the intervention or service (e.g., process evaluation outcomes such as satisfaction, staff training, etc)
Setting	• UK based	• Not UK based

For peer review only - http://bmjopen.bmj.com/site/about/guidelines.xhtml

	Inclusion	Exclusion
Study design	• RCT<br>• Nonrandomised comparative<br>• Pre-post<br>• Service evaluation	• Cross-sectional, case control, case study<br>• Qualitative<br>• Letters to the editor, think pieces, descriptive only
Dates	• 1982-present	• Pre-1982

Information sources and search strategy

MEDLINE, EMBASE, PsycINFO, Social Policy and Practice, ASSIA, IBSS, Sociological abstracts and SSCI electronic databases were searched from inception until 21st June 2022. Grey literature sources were also identified, including searching electronic grey literature databases (National Grey Literature Collection, EThOS, Social Care Online and the Violence Against Women Network) (Appendix 2), a call for evidence which was shared with research networks and UK-based DSVA services and organisations, and searches of relevant UK charity and organisation websites. Finally, backwards and forwards citation tracking of all included studies, as well as reference searching of identified systematic reviews was undertaken to identify further relevant studies. The search results were exported into EndNote and duplicates were removed.

Selection of sources of evidence

The de-duplicated citations were uploaded into Rayyan [46]. Titles and abstracts, followed by full-texts, were screened according to the inclusion and exclusion criteria. A 20% sample was independently screened by a second reviewer at each stage, and all disagreements were resolved by discussion, with a third reviewer where needed. Risk of bias assessments are not necessary for scoping reviews [41].

For peer review only - http://bmjopen.bmj.com/site/about/guidelines.xhtml

Data charting process and data items

Data charting took place using a piloted excel spreadsheet. Data items extracted included the study citation, study design, participant demographics, description of the service or intervention and comparison (if applicable), setting, and the outcome measures used. Data extraction and charting was carried out by one reviewer, and checked for errors by a second reviewer. Where data was missing, corresponding authors were contacted and asked to supply said data.

Synthesis of results

A narrative synthesis was conducted to summarise the included interventions and services and the effectiveness outcomes they measured. The unit of analysis was the outcome measure(s) reported. Interventions and services were grouped by the type of support offered, based on the intervention and service types outlined in the protocol. Where studies reported more than one form of support as part of the intervention or service, they were classified as “multi-service”. Where the same intervention or service produced multiple reports (e.g., from different years), these were combined into one entry during data charting so that all outcomes ever reported by that service were extracted, but were not double counted if multiple reports used the same outcome.

Effectiveness outcomes reported by studies were grouped thematically by type to create categories, domains and subdomains. The groupings and the names for the sub-domains, domains and categories were developed iteratively. All outcome measures were firstly listed in an excel spreadsheet. This included standardised and unstandardised questionnaires and single item measures. A column was added, describing what the outcome measured (e.g., the Beck Depression Inventory [47] measures depression). These sub-domains were then grouped into broader domains (e.g., depression is a mental health-related outcome). Where

For peer review only

applicable, these were grouped into again even broader categories (e.g., mental health is a form of overall health).

A list of all relevant outcomes and domains, and how frequently they were reported was made. Comparisons were made between outcomes reported by studies in different sectors, over time, and by service user (i.e., perpetrator or victim-survivor) and support type (i.e., psychological support, housing, combinations), or violence type (i.e., domestic violence and abuse [DVA], sexual violence and abuse [SVA], childhood sexual abuse [CSA]).

Patient and public involvement

An advisory group was set up comprising representatives from six specialist DSVA organisations who are involved in the delivery, planning, funding or support of specialist DSVA support services in the UK. The group inputted to the design of the study protocol, interpretation of the data, and provided insight regarding the challenges in measuring the effectiveness of support services in the third sector. Their input resulted in several changes, including the broadening of the scope of this review to all effectiveness outcomes, rather than a narrower focus on only outcomes relating to reducing violence, to reflect the priorities of the sector.

Results

Selection of sources of evidence

After duplications were removed, a total of 12431 records and 869 reports were screened for inclusion. Eighty studies from 111 reports were included in this review (see

). Data extracted from the included studies are summarised in Appendix 4 [48-154].

For peer review only - http://bmjopen.bmj.com/site/about/guidelines.xhtml

Characteristics of sources of evidence

The 80 studies described a total of 87 interventions or services, with seven studies describing both a perpetrator programme and an associated support service for (ex)partners of participating perpetrators. All studies were conducted between 1991 and 2022.

Description of studies

Study design

Many of the included studies were mixed-method service evaluations; for these studies, only the methodology relevant to the included outcomes is reported as the other methods do not meet the review eligibility criteria (e.g., qualitative, case studies). The most common study design was pre-post single group designs (n=53), of these 14 included additional time-points at mid-intervention or follow-up. Eight studies were RCTs, and ten used a non-randomised comparative design. For one study, the design could not be determined. Eight studies used secondary analysis of datasets, which collated data from multiple services. These were primarily publications produced by SafeLives, a UK-based charity dedicated to ending domestic abuse, using their Insights system, an outcome measurement tool which allows services across England and Wales to collect data according to various measurement indicators.

Perpetrator programmes

Perpetrator programmes were grouped together. They were typically described as behaviour change programmes based on a cognitive behavioural model, and whilst many included additional elements such as psychological support, these were often described insufficient detail. Of the 80 included studies, 27 described a perpetrator programme.

For peer review only

Victim support interventions and services

Interventions and services providing support for those who had experienced DSVA were grouped by type of support provided, based on initial scoping of the literature. The most common were psychological support (n=21), advocacy (n=14) and multi-service (n=11). Three studies each reported on multi-agency risk assessment conferences (MARACs) and specialist domestic violence police teams. Two studies reported housing interventions or services. One study each reported on sexual violence services, helpline services, outreach services, and adult health-based services. One study described a women's service linked to an associated perpetrator programme, but the specific type of support offered was unclear. Several studies evaluated the effectiveness of more than one intervention or service.

Type of DSVA

The studies were categorised as being primarily aimed at DVA, SVA, both DVA and SVA (DSVA), or CSA. Categorisations were based on how the study or service described itself, however it should be noted that the type of violence/abuse was often not clearly defined. Thus, these categorisations should be considered as having ambiguous boundaries, and interpreted with caution.

The majority (n=53; 66.3%) of the studies were aimed at DVA, whilst 24 (29.3%) were aimed specifically at SVA. Of these, nine studies were specifically targeted at adults who had experienced CSA. Three studies (3.8%) were aimed at both DVA and SVA, or DSVA.

Setting

Just under half of studies were conducted in the third sector (n=39; 47.6%). Twenty-six studies were conducted in the public sector, and 12 spanned multiple sectors. One was conducted in a privately owned prison, and for one study the setting was unclear.

For peer review only - http://bmjopen.bmj.com/site/about/guidelines.xhtml

Types of outcomes

The 80 included studies measured a total of 426 outcomes. Of these 200 (47.0%) were used more than once. In total, there were 282 unique outcomes, 57 of which were used more than once, and 226 were used only once. The number of effectiveness outcomes measured by each individual study ranged from one to 19, with a mean average of 5.23.

Outcomes were grouped thematically into 11 domains and 49 subdomains. The 11 domains included mental health, physical health, general wellbeing (all of which fall under the broad category of 'health'), the experience of DSVA and the perpetration of DSVA (which fall under the broad category of 'DSVA'), empowerment, socio-economic circumstances, behavioural outcomes, the victim-perpetrator relationship, parenting, and changing perpetrator beliefs and skills (Appendix 5**Error! Reference source not found.**).

Many of the outcome measures reported spanned two or more domains (Figure 2). For instance, 'health' as an overarching category included three domains: mental health, physical health and general wellbeing. 'Quality of life' as a subdomain spanned physical health, mental health and general wellbeing domains, based on the identified outcome measures and their specific items. For example, the 12-item Short Form (SF-12) is a measure of quality of life and general health, which includes items relating to physical health, mental health, and general wellbeing. Similarly, whilst the overarching category of DSVA contained two primary domains: the experience of DSVA and the perpetration of DSVA, many outcome measures could fall into either domain, depending on who the respondent was (i.e., the perpetrator or the victim-survivor). Thus, there was substantial overlap between these two subdomains. Many of the outcomes within the DSVA category, whilst defined slightly differently, were not distinct. For instance, there is significant overlap between the concept of 'revictimization' and the outcome 'presence of abuse' when measured using a pre-post design (if the victim-survivor indicates that they are still experiencing abuse post-

For peer review only - http://bmjopen.bmj.com/site/about/guidelines.xhtml

intervention, then this indicates ‘revictimization’). There is similarly overlap between reoffending and revictimization (although we cannot presume a direct overlap, as a perpetrator may have multiple victims) and between ‘reoffending’ and ‘police incidents’ (although not all reoffending will be recorded by police therefore this subdomain may be broader).

For each of the 11 domains, the total number of outcomes, the total number of unique outcomes, and the number of studies reporting at least one outcome that fell within that domain, were explored. The domain ‘perpetration of abuse’ has the highest number of studies reporting at least one outcome, the most total outcome measures, and the most unique outcome measures (Table 2).

For peer review only

Table 2. Frequency of outcomes by outcome domain, and the number of studies reporting one or more outcomes per outcome domains by intervention type, source of study, violence type and sector

		Outcome measure domain										
		Mental health	Physical health	Wellbeing	Behavioural	Empowerment	Socio-economic circumstances	Changing perpetrator beliefs and skills	Victim-perpetrator relationship	Parenting	Experience of DSVA	Perpetration of DSVA
Number of studies reporting at least one outcome		29	11	19	5	21	9	14	10	5	42	46
Number of total outcome measures		85	22	25	11	32	17	33	12	7	189	201
Number of total unique outcome measures		52	16	21	11	23	17	28	7	5	106	119
**Type of intervention**	**Number of studies**											
Perpetrator programme	27	2	1	2	1	1	1	14	4	3	14	21
Victim support:												
Psychological support	21	17	1	7	2	10	2	0	0	1	1	0
Advocacy	14	5	4	4	0	4	2	0	2	1	12	11
Multi-service	11	4	4	4	1	5	1	0	0	0	8	5
Housing	2	0	0	0	0	0	1	0	1	0	1	1
MARAC	3	0	0	0	0	0	0	0	0	0	3	2
Specialist DV police teams	3	0	0	1	0	0	0	0	0	0	2	2
Specialist DV court	1	0	0	0	0	0	0	0	0	0	0	1
Sexual violence service	1	1	1	1	1	1	1	0	0	0	1	1
Helpline	1	0	0	0	0	0	0	0	1	0	1	1
Outreach	1	0	0	0	0	0	0	0	1	0	1	1

For peer review only - http://bmjopen.bmj.com/site/about/guidelines.xhtml

Health	1	0	0	0	0	0	1	0	1	0	1	1
Other / Unclear	1	0	0	0	0	0	0	0	0	0	1	1
**Source**												
Peer reviewed	41	22	6	10	3	11	3	9	1	3	13	19
Grey literature	39	7	5	9	2	10	6	5	9	2	29	27
**Type of violence**												
DVA	53	11	9	12	2	10	6	8	10	5	40	41
SVA	15	7	2	4	2	5	1	6	0	0	1	4
Both	3	2	0	0	0	1	0	0	0	0	1	1
CSA	9	9	0	3	1	5	2	0	0	0	0	0
**Sector**												
Third	39	12	7	14	3	12	7	5	9	4	27	23
Public	26	13	2	3	1	8	2	5	0	1	5	10
Mixed	12	3	2	1	1	0	0	2	1	0	9	11
Private	1	1	0	1	0	1	0	1	0	0	0	0
Unclear	2	0	0	0	0	0	0	1	0	0	1	2

Note: CSA: Childhood sexual abuse; DSVA: Domestic and sexual violence and abuse; DV: Domestic violence; DVA: domestic violence and abuse: MARAC: Multi-agency risk assessment conference; SVA: Sexual violence and abuse
Note: Sixteen studies reported an outcome that fits into multiple domains therefore some outcomes are represented by multiple domains. Additionally, 38 studies reported an outcome that was categorised as both the experience of DSVA and perpetration of DSVA, because the specific outcome could represent either domain depending on who is completing the outcome measure.
Note: Two studies have outcome domains that are represented in multiple intervention types (perpetrator programme, unclear/other and advocacy), because they report a perpetrator programme and an associated women's service, but it is unclear which intervention/service the outcomes were measured as part of.
Note: The number of studies for the different intervention/service types is more than the total number of studies (80) because 7 studies reported a perpetrator programme and associated women's service.

For peer review only - http://bmjopen.bmj.com/site/about/guidelines.xhtml

Of the three most common outcome measures across intervention types, all fell within the DSVA category (experience of DSVA / perpetration of DSVA; Table 3). Cessation of abuse according to the Severity of Abuse Grid (SAG) [155] was the most common outcome (n=11), followed by the presence of abuse (n=10) and the severity of abuse (n=8), both also measured by the SAG. The Rosenberg Self-Esteem Scale [156], and two more measures of DSVA from the SAG (multiple types of abuse; abuse that is escalating in severity or frequency) were each reported by seven studies.

For peer review only

Table 3: Most commonly reported outcome measures

Outcome	Subdomain	Domain	No. of studies
**Overall**			
1. Cessation of abuse measured by the SAG [155]	Presence of abuse	Experience of DSVA/ perpetration of DSVA	11
2. Presence of abuse measured by the SAG* [155]	Presence of abuse	Experience of DSVA/ perpetration of DSVA	10
3. Severity of abuse measured by the SAG* [155]	Severity of abuse	Experience of DSVA/ perpetration of DSVA	8
4. RSES [156]	Self-esteem	Empowerment	7
5. Multiple types of abuse measured by the SAG [155]	Presence of abuse	Experience of DSVA/ perpetration of DSVA	7
6. Abuse that is escalating in severity or frequency as measured by the SAG [155]	Severity and frequency of abuse	Experience of DSVA/ perpetration of DSVA	7
**Peer review vs grey literature**			
**Grey literature**			
1. Cessation of abuse measured by the SAG [155]	Presence of abuse	Experience of DSVA/ perpetration of DSVA	9
2. Presence of abuse measured by the SAG* [155]	Presence of abuse	Experience of DSVA/ perpetration of DSVA	9
3. Severity of abuse measured by the SAG* [155]	Severity of abuse	Experience of DSVA/ perpetration of DSVA	8
**Peer review**			
1. RSES [156]	Self-esteem	Empowerment	6
2. BDI [47]	Depression	Mental health	5
3. CORE-OM [157]	Psychological issues	Mental health	5
**Sector**			
**Third**			
1. Cessation of abuse measured by the SAG [155]	Presence of abuse	Experience of DSVA/ perpetration of DSVA	9
2. Presence of abuse measured by the SAG* [155]	Presence of abuse	Experience of DSVA/ perpetration of DSVA	9
3. Severity of abuse measured by the SAG* [155]	Severity of abuse	Experience of DSVA/ perpetration of DSVA	8
**Public**			

For peer review only - http://bmjopen.bmj.com/site/about/guidelines.xhtml

Outcome	Subdomain	Domain	No. of studies
1. BDI [47]	Depression	Mental health	5
2. RSES [156]	Self-esteem	Empowerment	4
3. SCL-90R [158]	Psychological issues	Mental health	4
**Mixed**			
1. Cases discontinued	Criminal justice outcomes	Perpetration of DSVA	2
2. PHQ [159]	Depression	Mental health	2
3. GAD-7 [160]	Anxiety	Mental health	2
4. Cambridge Crime Harm Index [161]	Severity of abuse	Experience of DSVA/ perpetration of DSVA	2
5. PC-PTSD [162]	Trauma	Mental health	2
6. No further post-MARAC police complaints or call outs	Police incidents	Experience of DSVA/ perpetration of DSVA	2
7. No further post-MARAC police complaints	Police incidents	Experience of DSVA/ perpetration of DSVA	2
8. No further post-MARAC police call outs	Police incidents	Experience of DSVA/ perpetration of DSVA	2

Note: BDI: Beck Depression Inventory; CORE-OM: Clinical Outcomes in Routine Evaluation Outcome Measure; DSVA: Domestic and sexual violence and abuse; GAD-7: Generalised Anxiety Disorder Questionnaire; MARAC: Multi-agency risk assessment conference; PHQ: Patient Health Questionnaire; PC-PTSD-5: Primary Care PTSD Screen; RSES: Rosenberg Self-Esteem Scale; SAG: Severity of Abuse Grid; SCL-90R: Symptom Checklist 90 revised.
**Reported separately for physical abuse, sexual abuse, harassment and stalking, and jealous and controlling behaviour*

Patterns in the use of outcome

Table 2 summarises the patterns in outcome domains according to perpetrator programmes, victim support type, DSVA type, source of literature and setting/sector.

Outcomes in perpetrator programmes

Of the 27 perpetrator programmes, 14 reported at least one outcome relating to changing perpetrator behaviour, and 21 reported at least one relating to the perpetration of DSVA.

Outcomes by victim support type

The most common outcome domains for psychological support interventions and services were mental health (n=17) and empowerment (n=10), whilst advocacy, multi-service interventions and studies of MARACs primarily reported on experience of DSVA (n=12, n=8 and n=3, respectively). Specialist DV police teams reported equally on the experience of and the perpetration of DSVA (n=2 each).

For peer review only - http://bmjopen.bmj.com/site/about/guidelines.xhtml

For peer review only

Outcomes by DVSA type

Of the studies primarily focused on DVA, the majority reported at least one outcome relating to the experience of DSVA (n=40; 75.5%) and the perpetration of DSVA (n=41; 77.4%). Just under half of studies of adult SVA (n=7; 46.7%), and all studies of CSA, reported mental health outcomes (n=9; 100%).

Outcomes by source of literature

Whilst the split of included studies from the grey literature and peer reviewed literature was relatively even, studies located within the grey literature were more diverse. In total, grey literature studies reported 254 outcome measures, 178 of which were unique. In comparison, peer reviewed studies reported a total of 172 outcomes, 125 of which were unique. Peer reviewed studies had more of a tendency to report at least one outcome relating to mental health (n=22; 53.7%), whilst grey literature studies were more likely to report outcomes relating to the experience of DSVA (n=29; 74.4%).

Outcomes by setting/sector

Two thirds of studies set in the third sector reported outcomes relating to the experience of DSVA subdomain (n=27; 69.2%). Perpetration of DSVA was the most common outcome subdomain for interventions and services with a mixed setting (n=11; 91.7%), whilst mental health was the most common subdomain for those in the public sector (n=13; 50%).

Outcomes 1991 to 2022

Evaluations of DSVA support services and interventions have become more frequent over time. Nearly half (48.8%) of included studies were published in 2015-2022, with over a quarter (26.3%) published in 2019-2022 (Appendix 6).

For peer review only - http://bmjopen.bmj.com/site/about/guidelines.xhtml

Discussion

This study used scoping review methodology to map which outcomes have been used in studies assessing the effectiveness of a range of support interventions and services for people who have experienced or perpetrated DSVA. A total of 426 outcome measures were used in 80 papers and reports, describing 87 interventions and services. Less than half of the measures were used more than once.

The most common outcome domain was the perpetration of DSVA, followed closely by the experience of DSVA, both of which included outcomes relating directly to abusive and violent behaviours. This demonstrates that the measurement of DSVA has been prioritised when assessing service and intervention effectiveness, and is consistent with the common goal of many DSVA support services and organisations of ending violence and abuse [163, 164]. However, 201 and 189 unique outcome measures were charted within these two domains respectively, showing the lack of consensus regarding the specific outcomes that should be measured. The SAG, which measures several different aspects of abusive behaviours (including presence of four types of abuse, frequency, severity, and cessation of abuse), was the most frequently used outcome measure. It was primarily used by studies drawing upon the SafeLives' Insights datasets, although it was employed by several additional studies too. Despite being the most frequently reported outcome, the SAG was still utilised by only 13 of the 80 studies, illustrating the inconsistencies and variation in measures being used by these evaluations and reports.

Of the 282 unique outcomes reported, 226 were used only once. Although many of these outcomes were similar, they often differed slightly according to the unit or method of measurement, meaning that combining them would be methodologically inappropriate. This further demonstrates the disparate and highly variable nature of outcome measures currently

For peer review only - http://bmjopen.bmj.com/site/about/guidelines.xhtml

in use. Whilst this may be partly explained by the fact that different sectors and types of support interventions have different priorities, such disparity is evident even *within* sectors and intervention types. For instance, of the 153 different outcome measures reported by third sector studies, only 24.8% were used more than once, with a similarly low proportion in mixed sector and public sector studies (both 30.7%). This indicates a lack of comparability surrounding the best way to assess the effectiveness of support services and interventions, and makes synthesis such as meta-analysis less feasible.

Mapping revealed differences between the types of outcome domains frequently reported in the peer reviewed and grey literature, with peer reviewed studies focusing more on mental health related outcomes, and grey literature studies focusing primarily on DSVA. This difference may be explained by the overlap between grey literature and third sector-based studies, and the fact that third sector organisations are heavily tied to the outcomes that funders and commissioners require them to measure. With the focus on wanting to establish and reduce the prevalence of DSVA itself, and the associated focus on the perpetration of violence, it might be that more funders and commissioners are requiring these to be measured, or are prioritising these measurements above the wider impacts of DSVA.

Mapping of trends over time showed that as well as a general increase in the number of studies being conducted, there appeared to be increased diversity in outcomes measured. The two most common outcome categories, DSVA and health, have been measured fairly consistently over the past thirty years, whilst domains such as empowerment, the victim-perpetrator relationship and parenting have become more common in the past decade. This is likely reflective of an increased knowledge of the wide-ranging impacts of DSVA and its ripple effects, increased recognition of the need for holistic support, and the move towards survivor-centred outcome measurement and consultation with those with lived experience [159].

For peer review only - http://bmjopen.bmj.com/site/about/guidelines.xhtml

Challenges in outcome measurement

There are two linked challenges facing UK-based DSVA services, especially those located in the third sector. Firstly, funding of DSVA services is precarious and diminishing [166-169]. Being awarded funding requires evidence of effectiveness. However, the second challenge is that carrying out service evaluations to provide evidence of effectiveness is costly, making it even more difficult for services to secure funding. This is reflected in take-up of the aforementioned SafeLives' Insights system, which captures rich data and has likely contributed to more consistent reporting of outcomes across England and Wales. However, individual services have to pay to use the system, and the numbers of services contributing to the dataset each year has reduced, likely due to reduced funding [consultation with stakeholder advisory group].

Limitations

One limitation of this review is the unavoidable potential for double counting. For instance, the services that are included in SafeLives' Insights datasets are anonymous. Thus, it is not possible to determine if any services included in the Insights datasets have also published an independent evaluation. A second limitation of this review is that despite a comprehensive search strategy, no evaluations of support services run by by-and-for organisations were identified (see Appendix 1). This is illustrative of a systemic, cyclical and enduring issue facing these smaller services in particular, who are disproportionally underfunded and lack the resources needed to conduct and publish evaluations, meaning they are underrepresented in the literature [170-173]. Thirdly, we excluded process evaluation outcomes. Given that there may be causal links between process measures and distal outcomes, it is possible that some relevant outcomes were missed. Finally, given the primary objectives of the review were to map and chart outcome measures being used, we have not made any assessments regarding the validity or relevance of any particular measure. Therefore, whilst we have identified the most commonly used outcome measures,

For peer review only - http://bmjopen.bmj.com/site/about/guidelines.xhtml

that is not to say that these are the most helpful, informative or important to people with lived experience or front-line service providers.

Implications and recommendations

This review has demonstrated that the types of outcomes measured when assessing the effectiveness of support interventions and services for people who have experienced DSVA vary widely. This has implications for systematic reviews and evidence syntheses looking to assess the state of the literature, as well as for the allocation of funding and the development of policies related to outcome measurement. Increased consensus between researchers, service providers, and funders is needed. This will allow for more meaningful syntheses of the literature, as well as building a larger evidence base, so that a better understanding of the most effective means of support for people who have experienced DSVA can be reached. Further research is needed to determine which outcomes and outcome measures are most appropriate, valid and relevant, in order to work towards a consensus and build a shared evidence base to enable future meta-analyses. This work should be carried out as a collaboration between people who have experienced abuse and their families, service providers and, in the UK, commissioners, to ensure researchers and evaluators are measuring the outcomes that matter most to the people who matter most. Such approaches have previously been used to develop outcome frameworks in related areas [40].

Conclusions

A broad range of outcome measures have been used to assess support interventions for people who have experienced or perpetrated DSVA. There is growing recognition across various sectors of the potential benefits of a core set of outcomes, which include increasing consistency and consensus [174, 175]. We need to take stock of the current state of the evidence and reconceptualise what a core set of outcomes should look like across and within sectors. This core outcome set needs to be co-produced by a range of stakeholders,

For peer review only - http://bmjopen.bmj.com/site/about/guidelines.xhtml

including those with experience of DSVA and those providing front line support. For this to be feasible a trade-off needs to occur. Burden needs to be minimised by not requiring excessive data collection, but instead, collection of only the most relevant outcome measures according to the specific purpose of the intervention or service. At the same time, within-service consistency needs to be increased, as well as supporting comparability across sectors. Further research is needed to better understand which outcomes should be prioritised in order to strike this balance.

For peer review only

For peer review only - http://bmjopen.bmj.com/site/about/guidelines.xhtml

References

1. UK Faculty of Public Health, *The role of public health in the prevention of violence* 2016.
2. Savage-Borne, J. and A. Maitra. *Intimate partner violence as a public health problem*. 2021; Available from: https://blogs.bmj.com/bmj/2021/03/25/intimate-partner-violence-as-a-public-health-problem/.
3. World Health Organization. *Voilence against women*. 2021; Available from: https://www.who.int/news-room/fact-sheets/detail/violence-against-women.
4. Office for National Statistics (ONS), *Domestic abuse prevalence and victim characteristics Year ending March 2022*, Office for National Statistics (ONS), Editor. 2022.
5. World Health Organization, *Global and regional estimates of violence against women: prevalence and health effects of intimate partner violence and non-partner sexual violence*. 2013: World Health Organization.
6. Fulu, E., et al., *Prevalence of and factors associated with male perpetration of intimate partner violence: findings from the UN Multi-country Cross-sectional Study on Men and Violence in Asia and the Pacific.* The lancet global health, 2013. **1**(4): p. e187-e207.
7. Herbert, A., et al., *Risk factors for intimate partner violence and abuse among adolescents and young adults: findings from a UK population-based cohort.* Wellcome open research, 2020. **5**.
8. Hester, M., *Who Does What to Whom?: Gender and Domestic Violence Perpetrators*. 2009: University of Bristol [in association with] Northern Rock Foundation Bristol.
9. Robinson, A.L., *Serial domestic abuse in Wales: an exploratory study into its definition, prevalence, correlates, and management.* Victims & Offenders, 2017. **12**(5): p. 643-662.
10. Gilchrist, G., et al., *The prevalence and factors associated with ever perpetrating intimate partner violence by men receiving substance use treatment in Brazil and England: A cross-cultural comparison.* Drug and Alcohol Review, 2017. **36**(1): p. 34-51.
11. Basile, K.C., et al., *National intimate partner and sexual violence survey: 2010 summary report.* 2011.
12. Breiding, M.J., M.C. Black, and G.W. Ryan, *Chronic disease and health risk behaviors associated with intimate partner violence—18 US states/territories, 2005.* Annals of epidemiology, 2008. **18**(7): p. 538-544.
13. Chandan, J.S., et al., *Risk of cardiometabolic disease and all-cause mortality in female survivors of domestic abuse.* Journal of the American Heart Association, 2020. **9**(4): p. e014580.
14. Crofford, L.J., *Violence, stress, and somatic syndromes.* Trauma, Violence, & Abuse, 2007. **8**(3): p. 299-313.
15. John, R., et al., *Domestic violence: prevalence and association with gynaecological symptoms.* BJOG: An International Journal of Obstetrics & Gynaecology, 2004. **111**(10): p. 1128-1132.
16. Leserman, J. and D.A. Drossman, *Relationship of abuse history to functional gastrointestinal disorders and symptoms: some possible mediating mechanisms.* Trauma, Violence, & Abuse, 2007. **8**(3): p. 331-343.
17. Tjaden, P. and N. Thoennes, *Prevalence and consequences of male-to-female and female-to-male intimate partner violence as measured by the National Violence Against Women Survey.* Violence against women, 2000. **6**(2): p. 142-161.
18. McManus, S., et al., *Receiving threatening or obscene messages from a partner and mental health, self-harm and suicidality: results from the Adult Psychiatric Morbidity Survey.* Social psychiatry and psychiatric epidemiology, 2021: p. 1-11.

For peer review only - http://bmjopen.bmj.com/site/about/guidelines.xhtml

19. Povey, D., et al., *Homicides, Firearm Offences and Intimate Violence 2006/07 (Supplementary Volume 2 to Crime in England and Wales 2006/07) Home Office Statistical Bulletin 03/08 [online]. London: Home Office Research Development and Statistics Directorate*. 2008.
20. McManus, S., et al., *Intimate Partner Violence, Suicidality, and Self-Harm: A Probability Sample Survey of the General Population in England.* Suicidality, and Self-Harm: A Probability Sample Survey of the General Population in England, 2022.
21. Jonas, S., et al., *Gender differences in intimate partner violence and psychiatric disorders in England: results from the 2007 adult psychiatric morbidity survey.* Epidemiology and psychiatric sciences, 2014. **23**(2): p. 189-199.
22. St. Mungo's, *Rebuilding Shattered Lives [online]*. 2014.
23. Women's Aid, *The Domestic Abuse Report 2019: The Economics of Abuse Report Summary*. 2019.
24. Crown Prosecution Service, *Violence against Women and Girls Report 10th Edition 2016-17*. 2017.
25. Office for National Statistics (ONS), *Domestic abuse and the criminal justice system*. 2021.
26. ONS, *Domestic abuse in England and Wales overview: November 2021. Figures on domestic abuse from police recorded crime and a number of different organisations.* 2021.
27. Yakubovich, A.R., et al., *Housing interventions for women experiencing intimate partner violence: a systematic review.* The Lancet Public Health, 2021.
28. Kiani, Z., et al., *A systematic review: Empowerment interventions to reduce domestic violence?* Aggression and Violent Behavior, 2021. **58**: p. 101585.
29. Rivas, C., et al., *Advocacy interventions to reduce or eliminate violence and promote the physical and psychosocial well-being of women who experience intimate partner abuse: A systematic review.* Campbell Systematic Reviews, 2016. **12**(1): p. 1-202.
30. Hameed, M., et al., *Psychological therapies for women who experience intimate partner violence.* Cochrane database of systematic reviews, 2020(7).
31. Advance, *Annual Report and Accounts*. 2021.
32. Refuge, *Annual Report 2020–2021 and financial statements*. 2021.
33. Yellow Door, *Annual Review and Accounts 2020-2021*. 2021.
34. Oasis, *Impact*. 2019.
35. Taylor-Dunn, H. and R. Erol, *Evaluation of SaferPlaces' Independent Domestic Violence Advisor Services.* 2019.
36. Lilley-Walker, S.-J., M. Hester, and W. Turner, *Evaluation of European domestic violence perpetrator programmes: Toward a model for designing and reporting evaluations related to perpetrator treatment interventions.* International journal of offender therapy and comparative criminology, 2018. **62**(4): p. 868-884.
37. Akoensi, T.D., et al., *Domestic violence perpetrator programs in Europe, Part II: A systematic review of the state of evidence.* International journal of offender therapy and comparative criminology, 2013. **57**(10): p. 1206-1225.
38. National Institute for Health and Care Excellence. *Domestic violence and abuse: multi-agency working: Public health guideline [PH50]. Recommendations for research*. 2014; Available from: https://www.nice.org.uk/guidance/ph50/chapter/5-Recommendations-for-research.
39. James Lind Alliance. *Sexual Violence Top 10*. 2022; Available from: https://www.jla.nihr.ac.uk/priority-setting-partnerships/sexual-violence/top-10-priorities.htm.
40. Powell, C., et al., *Development of core outcome sets for family and child-focused interventions for child maltreatment and domestic violence*. 2021.
41. Munn, Z., et al., *Systematic review or scoping review? Guidance for authors when choosing between a systematic or scoping review approach.* BMC medical research methodology, 2018. **18**: p. 1-7.

For peer review only

42. Peterson, J., et al., *Understanding scoping reviews: Definition, purpose, and process.* Journal of the American Association of Nurse Practitioners, 2017. **29**(1): p. 12-16.
43. Tricco, A.C., et al., *PRISMA extension for scoping reviews (PRISMA-ScR): checklist and explanation.* Annals of internal medicine, 2018. **169**(7): p. 467-473.
44. Carlisle, S., et al. *Measuring the effectiveness of UK-based support interventions and/or services targeted at adults who have experienced domestic and sexual violence and abuse (DSVA): A scoping review*. 2022; Available from: https://osf.io/frh2e.
45. Home Office, *Policy Paper: Tackling perpetrators*. 2022.
46. Ouzzani, M., et al., *Rayyan — a web and mobile app for systematic reviews.* Systematic Reviews, 2016. **5**(210).
47. Beck, A.T., R.A. Steer, and G.K. Brown, *Beck depression inventory*. 1987: Harcourt Brace Jovanovich New York:.
48. ADVA. and S.P. Associates., *REPAIR (Resolve to End the Perpetration of Abuse in Relationships): A Community- and Whole-family-based Intervention Programme Targeting Perpetrators of Domestic Violence and Abuse in Devon. An evaluation of a three-year Invest to Save (ISB) PROJECT*. 2009: Exeter.
49. Advance, *'A Place to go Like This': Breaking the cycle of harm for mothers involved in offending who are survivors of domestic abuse, and their children.* 2020.
50. Barber, M., R. Jenkins, and C. Jones, *A survivor's group for women who have a learning disability.* British Journal of Developmental Disabilities, 2000. **46**(90, Pt 1): p. 31-41.
51. Bloomfield, S. and L. Dixon, *An outcome evaluation of the integrated domestic abuse programme (IDAP) and community domestic violence programme (CDVP).* London: National Offender Management Service, 2015.
52. Bowen, E., *Evaluation of a community based domestic violence offender rehabilitation programme. Unpublished doctoral thesis*, in *Centre for Forensic and Family Psychology*. 2004, University of Birmingham.
53. Bowen, E., *Therapeutic Environment and Outcomes in a UK Domestic Violence Perpetrator Program.* Small Group Research, 2010. **41**(2): p. 198-220.
54. Bowen, E. and E. Gilchrist, *Predicting dropout of court-mandated treatment in a British sample of domestic violence offenders.* Psychology, Crime & Law, 2006. **12**(5): p. 573-587.
55. Bowen, E., E. Gilchrist, and A.R. Beech, *Change in treatment has no relationship with subsequent re-offending in UK domestic violence sample: A preliminary study.* International Journal of Offender Therapy and Comparative Criminology, 2008. **52**(5): p. 598-614.
56. Bowen, E., E.A. Gilchrist, and A.R. Beech, *An Examination of the Impact of Community-Based Rehabilitation on the Offending Behaviour of Male Domestic Violence Offenders and the Characteristics Associated with Recidivism.* Legal and Criminological Psychology, 2005. **10**(2): p. 189-209.
57. Brady, F., et al., *Narrative exposure therapy for survivors of human trafficking: feasibility randomised controlled trial.* BJPsych Open, 2021. **7**(6).
58. Burton, S., L. Regan, and L. Kelly, *Supporting women and challenging men: lessons from the Domestic Violence Intervention Project.* 1998.
59. Jane Callaghan, et al., *Make a Change: An evaluation of the implementation of an early response intervention for those who have used abusive behaviours in their intimate relationships*. 2020.
60. Respect and Women's Aid Federation England, *Make a Change Executive Summary*.
61. Calvert, R., S. Kellett, and T. Hagan, *Group cognitive analytic therapy for female survivors of childhood sexual abuse.* British Journal of Clinical Psychology, 2015. **54**(4): p. 391-413.
62. Clarke, S. and S. Llewelyn, *Personal constructs of survivors of childhood sexual abuse receiving cognitive analytic therapy.* British Journal of Medical Psychology, 1994. **67(3)**: p. 273-289.

For peer review only - http://bmjopen.bmj.com/site/about/guidelines.xhtml

63. Clarke, S. and C. Pearson, *Personal constructs of male survivors of childhood sexual abuse receiving cognitive analytic therapy.* British Journal of Medical Psychology, 2000. **73(2)**: p. 169-177.
64. Cook, D.A., et al., *The Berkeley group: ten years' experience of a group for non-violent sex offenders.* The British Journal of Psychiatry, 1991. **158**(2): p. 238-243.
65. Co-Ordinated Action Against Domestic, A., *Insights into domestic abuse 1: a place of greater safety.* 2012.
66. Co-ordinated Action Against Domestic Abuse (CAADA), *Insights National Dataset 2011–12 Appendix to: A place of greater safety*. 2012.
67. Coy, M. and L. Kelly, *Islands in the stream: an evaluation of four London independent domestic violence advocacy schemes*. 2011.
68. Safelink, *"In my way…" Evaluation Of Learning Disabilities Sexual Abuse Support Services*.
69. Dobash, R.P., et al., *A research evaluation of British programmes for violent men.* Journal of Social Policy, 1999. **28**(2): p. 205-233.
70. Ellis, F., *Rehabilitation programme for adult survivors of childhood sexual abuse.* Journal of Public Mental Health, 2012. **11**(2): p. 88-92.
71. Farrell, G. and A. Buckley, *Evaluation of a UK police domestic violence unit using repeat victimisation as a performance indicator.* The Howard Journal of Criminal Justice, 1999. **38**(1): p. 42-53.
72. Ferrari, G., et al., *Psychological advocacy towards healing (PATH): A randomized controlled trial of a psychological intervention in a domestic violence service setting.* Plos One, 2018. **13**(11).
73. Friendship, C., R.E. Mann, and A.R. Beech, *Evaluation of a national prison-based treatment program for sexual offenders in England and Wales.* Journal of interpersonal violence, 2003. **18**(7): p. 744-759.
74. Garry, K. and P. Goodwin, *Adult Counselling Service Phase two evaluation report: year 1 2020-21*. 2021, Merida Associates.
75. Karen Garry and Ellie Munro, *Adult Counselling Service Interim evaluation | May 2019*. 2019.
76. Karen Garry and Ellie Munro, *Adult Counselling Service Evaluation report – phase one 2016-2020*. 2020.
77. Geoghegan-Fittall, S., C. Keeble, and D. Wunsch, *The Croydon Drive Project: A 2-year Evaluation Final Report*. 2020.
78. Gilchrist, G., et al., *ADVANCE integrated group intervention to address both substance use and intimate partner abuse perpetration by men in substance use treatment: a feasibility randomised controlled trial.* BMC public health, 2021. **21**(1): p. 1-20.
79. Goosey, J., L. Sherman, and P. Neyroud, *Integrated case management of repeated intimate partner violence: A randomized, controlled trial.* Cambridge Journal of Evidence-Based Policing, 2017. **1**(2): p. 174-189.
80. Halliwell, G., et al., *Cry for health: a quantitative evaluation of a hospital-based advocacy intervention for domestic violence and abuse.* Bmc Health Services Research, 2019. **19**(1).
81. Hester, M. and N. Westmarland, *Tackling domestic violence: effective interventions and approaches*. 2005: Home Office Research, Development and Statistics Directorate.
82. Hester, M., et al., *Evaluation of Year 2 of the Drive Project – A Pilot to Address High Risk Perpetrators of Domestic Abuse*. 2019.
83. Hester, M., et al., *Evaluation of the Drive Project – a pilot to address high-risk perpetrators of domestic abuse Year 1 Feasibility Study*. 2017.
84. Hester, M., et al., *Evaluation of the Drive Project – A Three-year Pilot to Address High-risk, High-harm Perpetrators of Domestic Abuse*. 2019.
85. Hester, M., et al., *Evaluation of the Drive Project – A Three-year Pilot to Address High-risk, High-harm Perpetrators of Domestic Abuse EXECUTIVE SUMMARY.* 2020.

For peer review only - http://bmjopen.bmj.com/site/about/guidelines.xhtml

86. Howarth, E. and et al., *Safety in numbers: a multi-site evaluation of Independent Domestic Violence Advisor Services.* 2009.
87. Howarth, E. and A. Robinson, *Responding Effectively to Women Experiencing Severe Abuse: Identifying Key Components of a British Advocacy Intervention.* Violence against Women, 2016. **22**(1): p. 41-63.
88. Karatzias, T., et al., *Group psychotherapy for female adult survivors of interpersonal psychological trauma: a preliminary study in Scotland.* Journal of Mental Health, 2016. **25**(6): p. 512-519.
89. Kelley, T.M., et al., *The Efficacy of Principle-Based Correctional Counseling for Improving the Self-Control and Mental Health of People Incarcerated for Sexual Violence.* Violence Against Women, 2022. **28**(2): p. 573-592.
90. Liz Kelly, *Domestic Violence Matters: an evaluation of a development project*. 1999.
91. Kelly, L. and N. Westmarland, *DOMESTIC VIOLENCE PERPETRATOR PROGRAMMES STEPS TOWARDS CHANGE: Project Mirabal Final Report*. 2015.
92. Koppensteiner, M.F., J. Matheson, and R. Plugor, *Project 360: An intervention to address victim-police engagement in repeat domestic violence cases*. 2017.
93. Lindsay, W.R., et al., *Comparing offenders against women and offenders against children on treatment outcome in offenders with intellectual disability.* Journal of Applied Research in Intellectual Disabilities, 2011. **24**(4): p. 361-369.
94. Lindsay, W.R., et al., *The treatment of six men with a learning disability convicted of sex offences with children.* British Journal of Clinical Psychology, 1998. **37**: p. 83-98.
95. Lowe, M., et al., *CORE assessment of adult survivors abused as children: A NAPAC group therapy evaluation.* Counselling & Psychotherapy Research, 2017. **17**(1): p. 71-79.
96. McConnell, N., M. Barnard, and J. Taylor, *Caring Dads Safer Children: Families' Perspectives on an Intervention for Maltreating Fathers.* Psychology of Violence, 2017. **7**(3): p. 406-416.
97. McConnell, N., et al., *Caring dads safer children*. 2014.
98. McCracken, K. and T. Deave, *Evaluation of the Caring Dads Cymru programme. Final report.* 2012.
99. Michie, A.M. and W.R. Lindsay, *A treatment component designed to enhance empathy in sex offenders with an intellectual disability.* The British Journal of Forensic Practice, 2012. **14**(1): p. 40-48.
100. Morgan, S.A., B.M.S. McCausland, and J. Parkes, *Baseline characteristics and outcomes of the main perpetrator programme within the Hampshire Domestic Abuse Prevention Partnership, UK: A mixed methods study.* Plos One, 2019. **14**(7).
101. Morgan, S. and J. Parkes, *The Hampshire Domestic Abuse Prevention Partnership 2016-2018: process and outcomes evaluation.* 2018.
102. Morrison, A. and L. Treliving, *Evaluation of outcome in a dynamically orientated group for adult males who have been sexually abused in childhood.* British Journal of Psychotherapy, 2002. **19**: p. 59-75.
103. Murphy, G. and C. Melvin, *Cognitive behavioural therapy for sexual offenders with autism spectrum disorders: A case study.* Whitby, Peggy Schaefer [Ed] (2020) Cases on teaching sexuality education to individuals with autism (pp 164-182) xxii, 289 pp Hershey, PA, US: Information Science Reference/IGI Global; US, 2020: p. 164-182.
104. Oasis, *Impact Report 2015-2016.* 2016.
105. Oasis. *Impact report 2013-2014*. 2014; Available from: https://issuu.com/kristinagwynne/docs/oasis_annual_report_2013-2014-web/1.
106. Ormston, R., C. Mullholland, and L. Setterfield, *Caledonian system evaluation: analysis of a programme for tackling domestic abuse in Scotland*. 2016, Edinburgh: Scotland. Scottish Government Social Research. vi, 88.

For peer review only - http://bmjopen.bmj.com/site/about/guidelines.xhtml

107. Payne, A., H. Liebling-Kalifani, and S. Joseph, *Client-centred group therapy for survivors of interpersonal trauma: A pilot investigation.* Counselling & Psychotherapy Research, 2007. **7**(2): p. 100-105.
108. Peckham, N.G., S. Howlett, and A. Corbett, *Evaluating a survivors group pilot for a women with significant intellectual disabilities who have been sexually abused.* Journal of Applied Research in Intellectual Disabilities, 2007. **20**(4): p. 308-322.
109. Robinson, A.L., *The Cardiff Women's Safety Unit: A multi-agency approach to domestic violence.* Final Evaluation Report. Cardiff University: School of Social Sciences.(Available online at http://www. cf. ac. uk/socsi/whoswho/robinson. html), 2003.
110. Robinson, A.L., *Domestic Violence MARACs (Multi-Agency Risk Assesment Conferences) for Very High-risk Victims in Cardiff, Wales: A Process and Outcome Evaluation.* 2004.
111. Robinson, A.L., *Reducing repeat victimization among high-risk victims of domestic violence: The benefits of a coordinated community response in Cardiff, Wales.* Violence Against Women, 2006. **12(8)**: p. 761-788.
112. Robjant, K., J. Roberts, and C. Katona, *Treating posttraumatic stress disorder in female victims of trafficking using narrative exposure therapy: A retrospective audit.* Frontiers in Psychiatry, 2017. **8**.
113. Rose, J., et al., *A sex offender treatment group for men with intellectual disabilities in a community setting.* The British Journal of Forensic Practice, 2012.
114. Ross, J., J. Sebire, and H. Strang, *Tracking Repeat Victimisation After Domestic Abuse Cases Are Heard With and Without Independent Domestic Violence Advisors (IDVAs) in an English Magistrate's Court.* Cambridge Journal of Evidence-Based Policing, 2022: p. 1-15.
115. Ryan, M., et al., *A prospective study of the effectiveness of group and individual psychotherapy for women CSA survivors.* Psychology and Psychotherapy: Theory, Research and Practice, 2005. **78(4)**: p. 465-480.
116. SafeLives, *Insights Idva national dataset 2012–13 Adult independent domestic violence advisor (Idva) services*. 2015.
117. SafeLives, *Insights Idva national dataset 2013–14 Adult independent domestic violence advisor (Idva) services*. 2015.
118. SafeLives, *Insights Idva England and Wales dataset 2016-17 Adult independent domestic violence advisor (Idva) services*. 2017.
119. SafeLives, *Insights Idva England and Wales dataset 2018-19 Adult Independent domestic violence advisor (Idva) services*. 2019.
120. SafeLives, *Insights Idva dataset 2020-21 Adult Independent domestic violence advisor (Idva) services*. 2021.
121. SafeLives, *Insights health England and Wales dataset 2015-2018 Adults health-based services*. 2018.
122. SafeLives, *Insights helpline England and Wales dataset 2014-17 Adult helpline services*. 2017.
123. SafeLives, *Defining Marac Outcomes National Pilot*. 2012.
124. SafeLives, *Insights outreach national dataset 2012–13 Adult outreach services*. 2015.
125. SafeLives, *Insights outreach national dataset 2013–14 Adult outreach services.* 2015.
126. SafeLives, *Insights outreach England and Wales dataset 2016-17 Adult outreach services*. 2017.
127. SafeLives, *Insights outreach England and Wales dataset 2018-19 Adult outreach services*. 2019.
128. SafeLives, *Insights outreach dataset 2020-21 Adult outreach services*. 2021.
129. SafeLives, *Insights England and Wales dataset 2018-19 Sexual violence services* 2019.
130. SafeLives, *Insights SV dataset 2020-21 Sexual violence (SV) services*. 2021.
131. Sharpe, J., et al., *Group analytic therapy for male survivors of childhood sexual abuse.* Group Analysis, 2001. **34**: p. 195-209.

For peer review only - http://bmjopen.bmj.com/site/about/guidelines.xhtml

132. Skyner, D.R. and J. Waters, *Working with perpetrators of domestic violence to protect women and children: a partnership between Cheshire Probation Service and the NSPCC.* Child Abuse Review: Journal of the British Association for the Study and Prevention of Child Abuse and Neglect, 1999. **8**(1): p. 46-54.
133. Smith, D., et al., *Adults with a history of child sexual abuse: Evaluation of a pilot therapy service.* British Medical Journal, 1995. **310(6988)**: p. 1175-1178.
134. Smith, E., et al., *Strengthening the mother-child relationship following domestic abuse: Service evaluation.* Child Abuse Review, 2015. **24**(4): p. 261-273.
135. Stanley, N., et al., *An evaluation of a new initiative for male perpetrators of domestic violence.* 2011.
136. Stanley, N. and a. et, *Roadmap evaluation: final report*. 2021, Bristol: Women's Aid SafeLives. 279.
137. Strang, H., et al., *Reducing the harm of intimate partner violence: Randomized controlled trial of the Hampshire Constabulary CARA Experiment.* Cambridge Journal of Evidence-Based Policing, 2017. **1**(2): p. 160-173.
138. Holly Taylor-Dunn and Rosie Erol, *Evaluation of SaferPlaces' Independent Domestic Violence Advisor Services.* 2019.
139. Taylor-Dunn, H. and R. Erol, *Improving the safety and well-being of domestic abuse survivors: The role of a specialist organisation in supporting the work of Independent Domestic Violence Advisors.* Crime Prevention and Community Safety, 2021. **23**(2): p. 115-136.
140. Eve; The Institute for Public Safety, C.J., *2019*. Evaluation of SHE.
141. Trevillion, K., et al., *Linking abuse and recovery through advocacy: An observational study.* Epidemiology and Psychiatric Sciences, 2014. **23**(1): p. 99-113.
142. Christine, V. and et al., *Evaluation of domestic violence pilot sites at Caerphilly (Gwent) and Croydon 2004/05.* 2005.
143. Webster, R., *Kent and Medway Independent Domestic Violence Advisor Service Impact Evaluation FINAL REPORT*. 2015.
144. Westmarland, N. and S. Alderson, *The Health, Mental Health, and Well-Being Benefits of Rape Crisis Counseling.* Journal of Interpersonal Violence, 2013. **28**(17): p. 3265-3282.
145. Williamson, D. and A. Boyle, *Results of the domestic violence advocacy program at addenbrookes hospital.* Academic Emergency Medicine, 2012. **19**: p. 741.
146. Williamson, E. and M. Hester, *Evaluation of the South Tyneside Domestic Abuse Perpetrator Programme (STDAPP) 2006-2008.* University of Bristol, 2009.
147. Aid., W.s., *Nowhere To Turn, 2022: Findings from the sixth year of the No Woman Turned Away project*. 2022, Women's Aid: Bristol.
148. Miles, C. and K. Smith, *Nowhere to turn, 2018: findings from the second year of the No Woman Turned away project*. 2018, Bristol: Women's Aid. 48.
149. Women's Aid, *NOWHERE TO TURN Findings from the first year of the No Woman Turned Away project*. 2017.
150. Women's Aid, *NOWHERE TO TURN Findings from the third year of the No Woman Turned Away project*. 2019.
151. Women's Aid, *NOWHERE TO TURN Findings from the fourth year of the No Woman Turned Away project*. 2020.
152. Women's Aid, *NOWHERE TO TURN 2021 Findings from the fifth year of the No Woman Turned Away project*. 2021.
153. Women's Resource, C., *Women and Girls Network: the WGN empowerment model - redressing negative individual and social impacts of gendered violence through holistic counselling.* 2011.
154. Women's Resource, C., *Ashiana Network: journey towards safety.* 2011.
155. Campbell, J.C. and K.L. Soeken, *Women's responses to battering over time: an analysis of change.* Journal of Interpersonal violence, 1999. **14**(1): p. 21-40.

For peer review only - http://bmjopen.bmj.com/site/about/guidelines.xhtml

156. Rosenberg, M., *Rosenberg self-esteem scale.* Journal of Religion and Health, 1965.
157. Evans, C., et al., *Towards a standardised brief outcome measure: Psychometric properties and utility of the CORE–OM.* The British Journal of Psychiatry, 2002. **180**(1): p. 51-60.
158. Derogatis, L.R., *SCL-90-R: Administration, scoring & procedures manual-II for the (revised) version and other instruments of the psychopathology rating scale series.* Clinical Psychometric Research., 1992: p. 1-16.
159. Kroenke, K., R.L. Spitzer, and J.B. Williams, *The PHQ-9: validity of a brief depression severity measure.* Journal of general internal medicine, 2001. **16**(9): p. 606-613.
160. Spitzer, R.L., et al., *A brief measure for assessing generalized anxiety disorder: the GAD-7.* Archives of internal medicine, 2006. **166**(10): p. 1092-1097.
161. Sherman, L., P.W. Neyroud, and E. Neyroud, *The Cambridge crime harm index: Measuring total harm from crime based on sentencing guidelines.* Policing: a journal of policy and practice, 2016. **10**(3): p. 171-183.
162. Prins, A., et al., *The primary care PTSD screen for DSM-5 (PC-PTSD-5).[measurement instrument].* Universität Bielefeld: Bielefeld, Germany, 2015.
163. Women Against Violence Europe, *WAVE Report 2015: On the Role of Specialist Women's Support Services in Europe*. 2016.
164. Women's Aid. *What we believe: Our values*. 2022; Available from: https://www.womensaid.org.uk/what-we-believe/.
165. Robotham, D., A. Sweeney, and C. Perôt, *Violence, abuse and mental health network: Survivors' priority themes and questions for research—Consultation report, 2019.* Retrieved June, 2019. **26**: p. 2020.
166. Macy, R.J., et al., *Domestic violence and sexual assault services: Historical concerns and contemporary challenges.* Journal of Interpersonal Violence, 2010. **25**(1): p. 3-32.
167. SafeLives, *We only do bones here': Why London needs a whole-health approach to domestic abuse*. 2021, SafeLives: London.
168. Wiley, K., *Implementing Domestic Violence Policy: When Accountability Trumps Mission.* Affilia, 2020. **35**(4): p. 533-551.
169. Women's Aid, *Fragile funding landscape: The extent of local authority commissioning in the domestic abuse refuge sector in England 2020*. 2021, Women's Aid: Bristol.
170. Towers, J. and S. Walby, *Measuring the impact of cuts in public expenditure on the provision of services to prevent violence against women and girls.* 2012.
171. Corry, D., *Hearing women's voices: Why women 2018.* . 2018.
172. Imkaan, *From Survival to Sustainability: Critical Issues for the Specialist Black and 'Minority Ethnic'Ending Violence Against Women and Girls Sector in the UK*. 2018, Imkaan London.
173. Heady, L., A. Kail, and C. Yeowart, *Understanding the stability and sustainability of the violence against women voluntary sector.* 2011.
174. Sullivan, R. and E. Sharples, *The CSA Centre's Evaluation Fund: A Reflection*. 2018, Barkingside: Centre of expertise on child sexual abuse.
175. Home Office, *Violence against women and girls services: commissioning toolkit*. 2022, Home Office: London.

For peer review only - http://bmjopen.bmj.com/site/about/guidelines.xhtml

FIGURE 1: PRISMA FLOW DIAGRAM

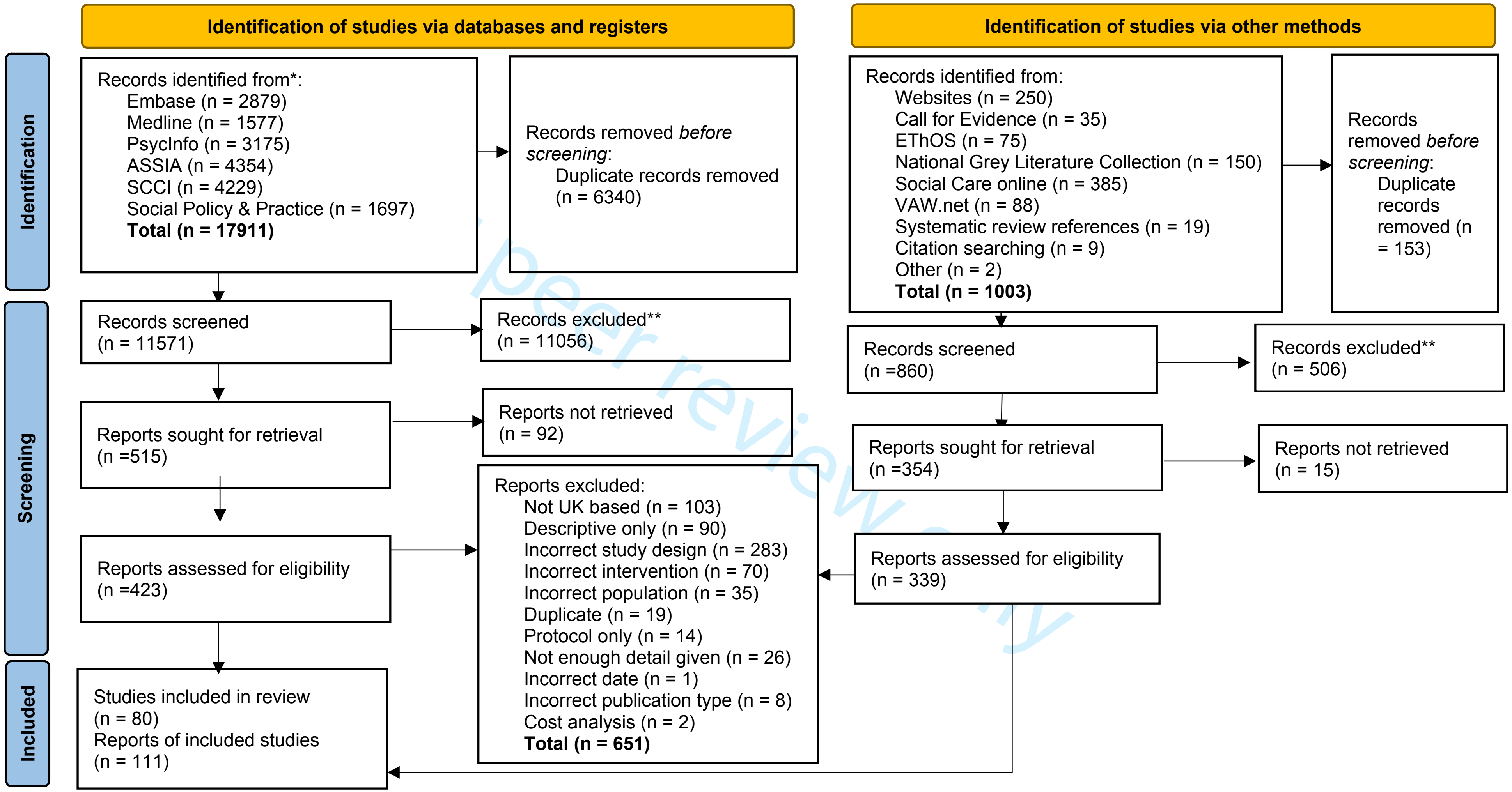

From: Page MJ, McKenzie JE, Bossuyt PM, Boutron I, Hoffmann TC, Mulrow CD, et al. The PRISMA 2020 statement: an updated guideline for reporting systematic reviews. BMJ 2021;372:n71. doi: 10.1136/bmj.n71. For more information, visit: http://www.prisma-statement.org/

Figure 2: Venn diagram of outcome categories, domains and subdomains

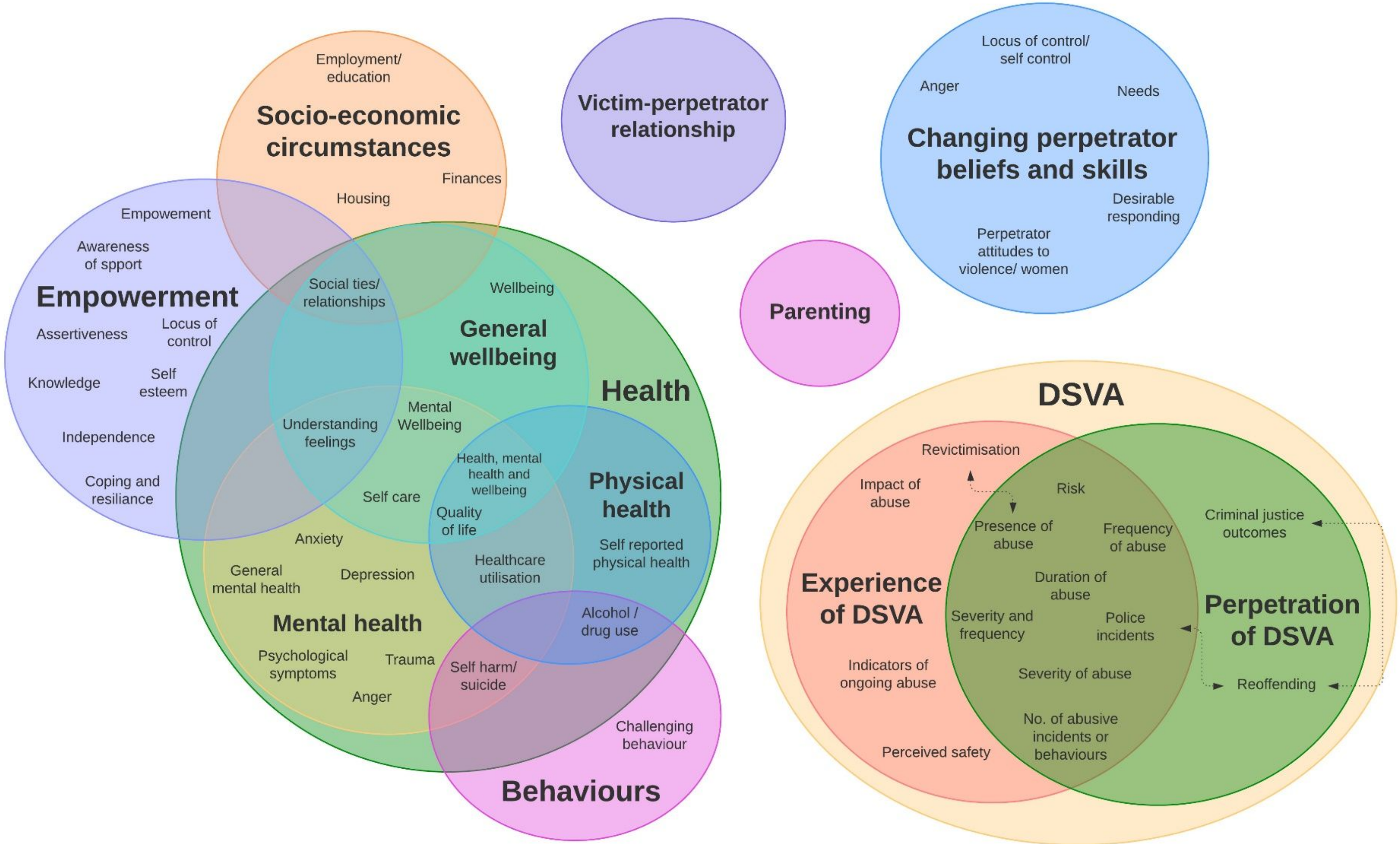

Note: DSVA: Domestic and sexual violence and abuse

Appendix 1: Glossary

Advocacy: Adapted from the National Institute for Health and Clinical Excellence (NICE) Domestic Violence and Abuse: Multi-agency Working public health guideline (PH50). In general, advocacy for people who have experienced DSVA includes: legal, housing and financial advice: access to and use of community resources such as refuges, emergency housing and psychological interventions; and safety planning advice. The activities may differ according to the level of risk facing the person. Crisis advocacy involves working with the person for a limited period of time (they may then be referred on to more specialist agencies). Practitioners providing advocacy can also provide ongoing support and informal counselling. The intensity of the advocacy provided may vary. It may last for a year – or longer, if the person is particularly vulnerable.

By-and-for: By-and-for services are lived experience organisation, where all of the staff, senior managers, Board of Trustees, and women and girls using services are Black and minoritised women. Such services have a Black feminist ethos, locating themselves as anti-racist, social justice and intersectional organisations.

Domestic and sexual violence and abuse (DSVA): This review works to the UK government definition of domestic violence and abuse[1], and The Istanbul Convention[2], The World Health Organisation[3], and The Rome Statute of the International Criminal Court's (ICC) Elements of Crimes definitions of sexual violence and abuse[4]. In brief, domestic violence and abuse is defined as behaviour within an intimate relationship or family which includes acts of physical violence, sexual violence, emotional or psychological abuse, and controlling behaviours. This also includes 'honour'-based violence and FGM. Sexual violence and abuse is defined as any non-consensual act of sexual nature, extending to attempts to obtain a sexual act, unwanted sexual comments or advances, or acts to traffic, using force, threat of force or coercion.

Independent domestic violence advisers (IDVAs): Adapted from the NICE PH50 guideline. Also known as independent domestic violence advocates, IDVAs work primarily with people at high risk of domestic violence and abuse, independently of any one agency, to secure their safety and the safety of their children. Serving as the primary point of contact, IDVAs normally work with their clients from the point of crisis to assess the level of risk, discuss the options and develop plans that address their immediate safety, as well as longer-term solutions. In many areas they are funded by the local community safety partnership, in some areas they are funded by the police or local authorities.

Intervention: Adapted from the NICE Glossary. A treatment, service, project, or programme.

Intimate partner violence (IPV): Adapted from the World Health Organisation's definition. Intimate partner violence refers to behaviour within an intimate relationship that causes physical, sexual or psychological harm, including acts of physical aggression, sexual coercion, psychological abuse and controlling behaviours. This definition covers violence by both current and former spouses and partners.

---

[1] https://www.gov.uk/government/publications/new-government-domestic-violence-and-abuse-definition/circular-0032013-new-government-domestic-violence-and-abuse-definition
[2] https://rm.coe.int/168008482e
[3] https://apps.who.int/iris/bitstream/handle/10665/42495/9241545615_eng.pdf
[4] https://www.icc-cpi.int/sites/default/files/Publications/Elements-of-Crimes.pdf

For peer review only

Organisation: In the context of this review, organisations refer exclusively to those that carry out activities relating to DSVA. Organisations may include both specialist service providers, and organisations that do not directly provider services (such as women's rights organisations that may focus on activities such as campaigning, research, supporting specialist services, training, and education, for issues relating to DSVA, for example).

People who have experienced DSVA: Throughout this review, people who experience DSVA refers to those who are victims or survivors of DSVA.

Perpetrator programmes: Any group or individual intervention for people who have perpetrated DSVA, with the aim of reducing the incidence of violence by changing attitudes, behaviours and/or beliefs of perpetrators.

Primary, secondary, and tertiary prevention: Primary prevention refers to any action, strategy or policy that works to stop domestic violence from occurring in the first place. Primary prevention seeks to reduce the overall likelihood that anyone will become a victim or perpetrator of domestic violence by creating conditions that make violence less likely to occur. Secondary prevention refers to intervening and responding to violence that has already occurred in order to stop violence from happening again. Secondary prevention activities can include refuge, counselling, and safety planning. Tertiary prevention refers to ongoing support to victims and ongoing accountability for abusers. Tertiary prevention activities address the long-term consequences of domestic violence. They can include support groups and other resources for survivors to help them heal so that they do not experience abuse again. They can also include perpetrator programs for abusers to prevent them from continuing to perpetrate violence in the future.

Refuge or shelter: Adapted from the NICE PH50 guideline. Residential service – a safe house – provided for adults (usually women) and children who are experiencing domestic violence and abuse.

Service provider: Primary, community, including third sector, secondary and tertiary care providers of health and social care services, including prison health services and criminal justice agencies. In the context of DSVA, the service providers are predominantly third sector providers. Third sector service providers may be specialist DSVA providers (i.e., they only provide services relating to DSVA) or non-specialist-DSVA providers (i.e., they provide specialist support services for DSVA as well as support services for other issues). Outside of the third sector, service providers such as the NHS, police and courts may provide some DSVA specific services.

Third sector organisations: In this review the definition of third sector services provided by the National Audit Office has been adopted. 'Third sector organisations' is a term used to describe the range of organisations that are neither public sector nor private sector. It includes voluntary and community organisations (both registered charities and other organisations such as associations, self-help groups and community groups), social enterprises, mutuals and co-operatives. Third sector organisations generally: are independent of government. This is also an important part of the history and culture of the sector; are 'value-driven'. This means they are motivated by the desire to achieve social goals (for example, improving public welfare, the environment or economic well-being) rather than the desire to distribute profit; and reinvest any surpluses generated in the pursuit of their goals. For this reason they are sometimes called 'not-for-profit organisations'. A better term is 'not-for-personal-profit'. In many cases, third sector organisations need to make surpluses (or 'profits') to be financially sustainable.

For peer review only

Appendix 2: Example search strategies

Peer review example: Medline

1. exp United kingdom/
2. (national health service* or nhs*).ti,ab,in.
3. (english not ((published or publication* or translat* or written or language* or speak* or literature or citation*) adj5 english)).ti,ab.
4. (gb or "g.b." or britain* or (british* not "british columbia") or uk or "u.k." or united kingdom* or (england* not "new england") or northern ireland* or northern irish* or scotland* or scottish* or ((wales or "south wales") not "new south wales") or welsh*).ti,ab,jw,in.
5. (bangor or "bangor's" or cardiff or "cardiff's" or newport or "newport's" or st asaph or "st asaph's" or st davids or swansea or "swansea's").ti,ab,in,cq.
6. (bath or "bath's" or ((birmingham not alabama*) or ("birmingham's" not alabama*) or bradford or "bradford's" or brighton or "brighton's" or bristol or "bristol's" or carlisle* or "carlisle's" or (cambridge not (massachusetts* or boston* or harvard*)) or ("cambridge's" not (massachusetts* or boston* or harvard*)) or (canterbury not zealand*) or ("canterbury's" not zealand*) or chelmsford or "chelmsford's" or chester or "chester's" or chichester or "chichester's" or coventry or "coventry's" or derby or "derby's" or (durham not (carolina* or nc)) or ("durham's" not (carolina* or nc)) or ely or "ely's" or exeter or "exeter's" or gloucester or "gloucester's" or hereford or "hereford's" or hull or "hull's" or lancaster or "lancaster's" or leeds* or leicester or "leicester's" or (lincoln not nebraska*) or ("lincoln's" not nebraska*) or (liverpool not (new south wales* or nsw)) or ("liverpool's" not (new south wales* or nsw)) or ((london not (ontario* or ont or toronto*)) or ("london's" not (ontario* or ont or toronto*)) or manchester or "manchester's" or (newcastle not (new south wales* or nsw)) or ("newcastle's" not (new south wales* or nsw)) or norwich or "norwich's" or nottingham or "nottingham's" or oxford or "oxford's" or peterborough or "peterborough's" or plymouth or "plymouth's" or portsmouth or "portsmouth's" or preston or "preston's" or ripon or "ripon's" or salford or "salford's" or salisbury or "salisbury's" or sheffield or "sheffield's" or southampton or "southampton's" or st albans or stoke or "stoke's" or sunderland or "sunderland's" or truro or "truro's" or wakefield or "wakefield's" or wells or westminster or "westminster's" or winchester or "winchester's" or wolverhampton or "wolverhampton's" or (worcester not (massachusetts* or boston* or harvard*)) or ("worcester's" not (massachusetts* or boston* or harvard*)) or (york not ("new york*" or ny or ontario* or ont or toronto*)) or ("york's" not ("new york*" or ny or ontario* or ont or toronto*))))).ti,ab,in.
7. (aberdeen or "aberdeen's" or dundee or "dundee's" or edinburgh or "edinburgh's" or glasgow or "glasgow's" or inverness or (perth not australia*) or ("perth's" not australia*) or stirling or "stirling's").ti,ab,in.
8. (armagh or "armagh's" or belfast or "belfast's" or lisburn or "lisburn's" or londonderry or "londonderry's" or derry or "derry's" or newry or "newry's").ti,ab,in.
9. (exp africa/ or exp americas/ or exp antarctic regions/ or exp arctic regions/ or exp asia/ or exp australia/ or exp oceania/) not (exp United Kingdom/ or europe/)

10. (child adj3 maltreat*).ti,ab,kw.
11. (sexual adj3 harass*).ti,ab,kw.
12. (batter* adj3 (wom#n or wife or wives)).ti,ab,kw.

13. rape*.ti,ab,kw.
14. (beat* adj3 (partner* or spous* or wom#n or wife or wives or m#n or husband*)).ti,ab,kw.
15. (sexual adj3 coercion).ti,ab,kw.
16. female genital mutilation.ti,ab,kw.
17. forced marriage.ti,ab,kw.
18. sex traffic*.ti,ab,kw.
19. revenge porn.ti,ab,kw.
20. controlling behaviour.ti,ab,kw.
21. prostitut*.ti,ab,kw.
22. sex work*.ti,ab,kw.
23. stalking.ti,ab,kw.
24. (drink adj3 spik*).ti,ab,kw.

25. domestic abuse service*.ti,ab,kw.
26. women's project*.ti,ab,kw.
27. women's resource centre.ti,ab,kw.
28. safety unit*.ti,ab,kw.
29. domestic abuse support service*.ti,ab,kw.
30. domestic abuse team*.ti,ab,kw.
31. domestic violence intervention*.ti,ab,kw.
32. women's service*.ti,ab,kw.
33. women's rights organi#ation*.ti,ab,kw.
34. domestic abuse advice and support.ti,ab,kw.
35. women's centre*.ti,ab,kw.
36. domestic abuse project*.ti,ab,kw.
37. domestic and sexual abuse project*.ti,ab,kw.
38. victim care service*.ti,ab,kw.
39. support centre*.ti,ab,kw.
40. women's welfare.ti,ab,kw.
41. speciali* service*.ti,ab,kw.
42. speciali* sexual violence service*.ti,ab,kw.
43. speciali* domestic violence service*.ti,ab,kw.
44. speciali* support.ti,ab,kw.
45. speciali* violence and abuse service*.ti,ab,kw.
46. speciali* women's voluntary sector service*.ti,ab,kw.
47. service* for survivors.ti,ab,kw.
48. victim service*.ti,ab,kw.
49. victim support*.ti,ab,kw.
50. violence against women and girls service*.ti,ab,kw.
51. domestic violence support service*.ti,ab,kw.
52. community based service*.ti,ab,kw.
53. sexual assault referral centre*.ti,ab,kw.
54. rape crisis.ti,ab,kw.
55. third sector.ti,ab,kw.
56. second tier.ti,ab,kw.
57. crisis intervention.ti,ab,kw.
58. independent domestic violence advoca*.ti,ab,kw.
59. IDVA.ti,ab,kw.
60. independent sexual violence advoca*.ti,ab,kw.
61. ISVA.ti,ab,kw.

For peer review only

62. shelter*.ti,ab,kw.
63. refuge.ti,ab,kw.
64. refuges.ti,ab,kw.
65. advoca*.ti,ab,kw.
66. counsel?ing.ti,ab,kw.
67. outreach.ti,ab,kw.
68. helpline*.ti,ab,kw.
69. emergency shelter*.ti,ab,kw.
70. supportive hous*.ti,ab,kw.
71. violence against women service*.ti,ab,kw.
72. recovery service*.ti,ab,kw.
73. speciali* victim service*.ti,ab,kw.
74. independent domestic violence advisor*.ti,ab,kw.
75. independent sexual violence advisor*.ti,ab,kw.
76. resettlement.ti,ab,kw.
77. floating support.ti,ab,kw.
78. drop-in.ti,ab,kw.
79. online chat.ti,ab,kw.
80. support group*.ti,ab,kw.
81. violence advocacy project*.ti,ab,kw.
82. group work program*.ti,ab,kw.
83. information and advice.ti,ab,kw.
84. (dedicated adj4 service*).ti,ab,kw.
85. charit*.ti,ab,kw.
86. independent.ti,ab,kw.
87. voluntary.ti,ab,kw.
88. public sector.ti,ab,kw.
89. specialist.ti,ab,kw.
90. speciali?ed.ti,ab,kw.
91. non-governmental organi#ation*.ti,ab,kw.
92. nonprofit organi#ation*.ti,ab,kw.
93. not-for-profit organi#ation*.ti,ab,kw.
94. by and for service*.ti,ab,kw.
95. by and for organi#ation*.ti,ab,kw.
96. therapy.ti,ab,kw.
97. empowerment.ti,ab,kw.
98. case management.ti,ab,kw.
99. stress management.ti,ab,kw.
100. safety behaviour*.ti,ab,kw.
101. safety strateg*.ti,ab,kw.
102. legal advice.ti,ab,kw.
103. mindfulness based stress reduction.ti,ab,kw.
104. home visit*.ti,ab,kw.
105. sanctuar*.ti,ab,kw.
106. safe house*.ti,ab,kw.
107. flexible fund*.ti,ab,kw.
108. exp battered woman/
109. exp domestic violence/ or exp child abuse/ or exp spouse abuse/ or exp gender-based violence/ or exp intimate partner violence/ or exp physical abuse/ or exp rape/
110. exp partner violence/
111. exp family violence/

For peer review only

112. exp coercion/
113. exp emotional abuse/
114. exp sexual violence/ or exp sexual abuse/ or exp sexual assault/
115. ("City of London" or Bedfordshire or Berkshire or Bristol or Buckinghamshire or Cambridgeshire or Cheshire or Cornwall or Cumbria or Derbyshire or Devon or Dorset or Durham or Yorkshire or Essex or Gloucestershire or "greater london" or "greater manchester" or "isle of wight" or "west midlands" or sussex or Hampshire or Herefordshire or Hertfordshire or Kent or Lancashire Leicestershire or Lincolnshire or Merseyside or Norfolk or Northamptonshire or Northumberland or Nottinghamshire or Oxfordshire or Rutland or Shropshire or Somerset or Staffordshire or Suffolk or Surrey or "Tyne and Wear" or Warwickshire or Wiltshire or Worcestershire or Aberdeenshire or Angus or Forfarshire or Argyll* or Ayrshire or "County of Ayr" or "County of Banff" or "County of Berwick" or "County of Bute" or "County of Clackmannan" or "East Lothian" or "Ross and Cromarty" or "West Lothian" or Banffshire or Berwickshire or Buteshire or Caithness or Clackmannanshire or Dumfriesshire or Dunbartonshire or Fife or Invernessshire or Kincardineshire or Kinrossshire or Kirkcudbrightshire or Lanarkshire or Midlothian or Moray or Nairnshire or Orkney or Peebleshire or Perthshire or Renfrewshire or Roxburghshire or Selkirkshire or Shetland or Stirlingshire or Sutherland or Wigtownshire or "Blaenau Gwent" or "Isle of Anglesey" or "Merthyr Tydfil" or "Neath Port Talbot" or "Rhondda Cynon Taff" or "Vale of Glamorgan" or Bridgend or Caerphilly or Cardiff or Carmarthenshire or Ceredigion or Conwy or Denbighshire or Flintshire or Gwynedd or Wrexham or Antrim or Armagh or Down or Fermanagh or Londonderry or derry or Swansea or Torfaen or Newport or Pembrokeshire or Powys or Monmouthshire or Tyrone).ti,ab,in.
116. 1 or 2 or 3 or 4 or 5 or 6 or 7 or 8 or 115
117. 116 not 9
118. tenant support.ti,ab,kw.
119. perpetrator programme*.ti,ab,kw.
120. (violen* adj3 (sexual or domestic or child or partner* or spous* or couple* or family or interpersonal or date or dating or gender based or coercive or conjugal or victim* or survivor* or perpetrat* or technology-facilitated or wom#n or image based or honor based or obstetric)).ti,ab,kw.
121. (abus* adj3 (sexual or domestic or child or partner* or spous* or family or couple* or gender based or wom#n or wife or wives or victim* or survivor* or perpetrat* or financ* or economic or image-based or technology-facilitated)).ti,ab,kw.
122. (assault* adj3 (sexual or partner* or spous* or victim* or survivor* or perpetrat* or couple*)).ti,ab,kw.
123. 25 or 26 or 27 or 28 or 29 or 30 or 31 or 32 or 33 or 34 or 35 or 36 or 37 or 38 or 39 or 40 or 41 or 42 or 43 or 44 or 45 or 46 or 47 or 48 or 49 or 50 or 51 or 52 or 53 or 54 or 55 or 56 or 57 or 58 or 59 or 60 or 61 or 62 or 63 or 64 or 65 or 66 or 67 or 68 or 69 or 70 or 71 or 72 or 73 or 74 or 75 or 76 or 77 or 78 or 79 or 80 or 81 or 82 or 83 or 84 or 85 or 86 or 87 or 88 or 89 or 90 or 91 or 92 or 93 or 94 or 95 or 96 or 97 or 98 or 99 or 100 or 101 or 102 or 103 or 104 or 105 or 106 or 107 or 118 or 119
124. 10 or 11 or 12 or 13 or 14 or 15 or 16 or 17 or 18 or 19 or 20 or 21 or 22 or 23 or 24 or 108 or 109 or 110 or 111 or 112 or 113 or 114 or 120 or 121 or 122
125. 117 and 123 and 124
126. limit 125 to (english language and humans)

For peer review only

Grey literature example: Social care online

(combined v4:
UK terms [
- Location:'"england"' including broader terms
- OR Location:'"wales"' including this term only
- OR Location:'"scotland"' including this term only
- OR Location:'"northern ireland"' including this term only
- OR Location:'"united kingdom"' including this term only
]
AND
service terms - all fields [
- AllFields:'"specialised service"'
- OR AllFields:'"specialist service"'
- OR AllFields:'"support service"'
- OR AllFields:'"abuse service"'
- OR AllFields:'"violence service"'
]
AND
violence terms v2 [
- SubjectTerms:'"domestic violence"' including related terms
- OR SubjectTerms:'"sexual abuse"' including related terms
- OR AllFields:'"sexual violence"'
- OR AllFields:'"domestic abuse"'
- OR AllFields:'"domestic violence"'
- OR AllFields:'"sexual abuse"'
]
)

For peer review only

Appendix 3: Preferred Reporting Items for Systematic reviews and Meta-Analyses extension for Scoping Reviews (PRISMA-ScR) Checklist

SECTION	ITEM	PRISMA-ScR CHECKLIST ITEM	REPORTED ON PAGE #
**TITLE**			
Title	1	Identify the report as a scoping review.	1
**ABSTRACT**			
Structured summary	2	Provide a structured summary that includes (as applicable): background, objectives, eligibility criteria, sources of evidence, charting methods, results, and conclusions that relate to the review questions and objectives.	1-2
**INTRODUCTION**			
Rationale	3	Describe the rationale for the review in the context of what is already known. Explain why the review questions/objectives lend themselves to a scoping review approach.	5-6
Objectives	4	Provide an explicit statement of the questions and objectives being addressed with reference to their key elements (e.g., population or participants, concepts, and context) or other relevant key elements used to conceptualize the review questions and/or objectives.	6
**METHODS**			
Protocol and registration	5	Indicate whether a review protocol exists; state if and where it can be accessed (e.g., a Web address); and if available, provide registration information, including the registration number.	7
Eligibility criteria	6	Specify characteristics of the sources of evidence used as eligibility criteria (e.g., years considered, language, and publication status), and provide a rationale.	7 and Table 1
Information sources*	7	Describe all information sources in the search (e.g., databases with dates of coverage and contact with authors to identify additional sources), as well as the date the most recent search was executed.	8
Search	8	Present the full electronic search strategy for at least 1 database, including any limits used, such that it could be repeated.	Appendix 2
Selection of sources of evidence†	9	State the process for selecting sources of evidence (i.e., screening and eligibility) included in the scoping review.	8
Data charting process‡	10	Describe the methods of charting data from the included sources of evidence (e.g., calibrated forms or forms that have been tested by the team before their use, and whether data charting was done independently or in duplicate) and any processes for obtaining and confirming data from investigators.	8
Data items	11	List and define all variables for which data were sought and any assumptions and simplifications made.	8
Critical appraisal of individual sources of evidence§	12	If done, provide a rationale for conducting a critical appraisal of included sources of evidence; describe the methods used and how this information was used in any data synthesis (if appropriate).	N/A
Synthesis of results	13	Describe the methods of handling and summarizing the data that were charted.	9

SECTION	ITEM	PRISMA-ScR CHECKLIST ITEM	REPORTED ON PAGE #
**RESULTS**			
Selection of sources of evidence	14	Give numbers of sources of evidence screened, assessed for eligibility, and included in the review, with reasons for exclusions at each stage, ideally using a flow diagram.	10 and Figure 1
Characteristics of sources of evidence	15	For each source of evidence, present characteristics for which data were charted and provide the citations.	10-14
Critical appraisal within sources of evidence	16	If done, present data on critical appraisal of included sources of evidence (see item 12).	N/A
Results of individual sources of evidence	17	For each included source of evidence, present the relevant data that were charted that relate to the review questions and objectives.	Appendix 4
Synthesis of results	18	Summarize and/or present the charting results as they relate to the review questions and objectives.	14-16
**DISCUSSION**			
Summary of evidence	19	Summarize the main results (including an overview of concepts, themes, and types of evidence available), link to the review questions and objectives, and consider the relevance to key groups.	16-18
Limitations	20	Discuss the limitations of the scoping review process.	18-19
Conclusions	21	Provide a general interpretation of the results with respect to the review questions and objectives, as well as potential implications and/or next steps.	19-20
**FUNDING**			
Funding	22	Describe sources of funding for the included sources of evidence, as well as sources of funding for the scoping review. Describe the role of the funders of the scoping review.	3

JBI = Joanna Briggs Institute; PRISMA-ScR = Preferred Reporting Items for Systematic reviews and Meta-Analyses extension for Scoping Reviews.
* Where *sources of evidence* (see second footnote) are compiled from, such as bibliographic databases, social media platforms, and Web sites.
† A more inclusive/heterogeneous term used to account for the different types of evidence or data sources (e.g., quantitative and/or qualitative research, expert opinion, and policy documents) that may be eligible in a scoping review as opposed to only studies. This is not to be confused with *information sources* (see first footnote).
‡ The frameworks by Arksey and O'Malley (6) and Levac and colleagues (7) and the JBI guidance (4, 5) refer to the process of data extraction in a scoping review as data charting.
§ The process of systematically examining research evidence to assess its validity, results, and relevance before using it to inform a decision. This term is used for items 12 and 19 instead of "risk of bias" (which is more applicable to systematic reviews of interventions) to include and acknowledge the various sources of evidence that may be used in a scoping review (e.g., quantitative and/or qualitative research, expert opinion, and policy document).

From: Tricco AC, Lillie E, Zarin W, O'Brien KK, Colquhoun H, Levac D, et al. PRISMA Extension for Scoping Reviews (PRISMAScR): Checklist and Explanation. Ann Intern Med. 2018;169:467–473. doi: 10.7326/M18-0850.

For peer review only

Appendix 4: Details of included studies

Study	Peer review or grey lit	Study design	Setting/ sector	Type of intervention	Type of abuse*	No. of outcomes
ADVA (2009)	Grey lit.	Service evaluation	Third	*"Repair"*<br>Perpetrator programme<br>• Comprising individual and group work sessions. Primarily cognitive behavioural, focusing on motivation, responsibility, safety and acknowledgment<br>Women's service<br>• Supportive befriending, information about legal and support services, expectation management, group work, telephone contact, feedback on partners' attendance, safety planning and monitoring and awareness raising	DVA	7
Advance (2020)	Grey lit.	Service evaluation	Third	*"Minerva"*<br>Multi-service<br>• Advocacy for access to other community services<br>• One-to-one and group work support on a range of issues including emotional wellbeing, physical and mental health, violence and abuse, problematic substance use<br>• Benefits and debt advice, housing	DVA	1
Barber et al (2000)	Peer review	Pre-post	Public: Learning disability hospital	-<br>Psychological support<br>• Group therapy using a person-centred therapeutic model, which aimed to run interactive and structured educative sessions, supplemented with supportive and non-confrontational discussions	SVA	4

For peer review only

Study	Peer review or grey lit	Study design	Setting/ sector	Type of intervention	Type of abuse*	No. of outcomes
Bloomfield & Dixon (2015)	Grey lit.	Non-randomised comparative	Public: National Probation Service	*“Integrated Domestic Abuse Programme (IDAP) and Community Domestic Violence Programme (CDVP)”*<br>Perpetrator programme<br>• Cognitive behavioural programme, which challenged offenders’ attitudes and beliefs to change their behaviour	DVA	2
Bowen (2010)	Peer review	Quasi experimental	Public: local probation service	*“Domestic Violence Perpetrator Programme”*<br>Perpetrator programme<br>• Psychoeducational treatment programme in a group setting, focusing on definitions of DV, socialisation, impact of DV on partners and children, empathy, sexual respect and accountability and communication	DVA	9
Brady et al (2021)	Peer review	RCT	Mixed: Third (human rights charity); unclear (‘clinical setting’)	-<br>Psychological support<br>• Narrative exposure therapy, involving co-construction of traumatic experiences and a therapy plan, to process traumatic memories. Psychoeducation was also delivered to all participants	SVA	5
Burton et al (1998)	Grey lit.	Service evaluation	Third	*“Violence Prevention Programme (VPP)”*<br>Perpetrator programme<br>• Structured group sessions designed to assist men in understanding why they abuse, how they can change it, and constructing respectful relationships with women<br>Multi-service<br>• Telephone advice and support; making contact with, and regular follow-up of, partners of men on VPP; one-to-one support sessions; two weekly support groups;	DVA	2

For peer review only

Study	Peer review or grey lit	Study design	Setting/ sector	Type of intervention	Type of abuse*	No. of outcomes
				advocacy for women users; weekly liaison with the VPP; interagency work, including workshops and training; administration; and training for new volunteers. These also involve safety planning, housing / financial / legal advice and education (understanding domestic violence)		
Callaghan et al (2020)	Grey lit.	Pre-post	Third: DV charities / organisations	*"Make a Change"*<br>Perpetrator programme<br>• Group-based intervention for perpetrators<br>Psychological support<br>• Individual support for (ex)partners	DVA	13
Calvert et al (2015)	Peer review	Pre-post	Public: Tertiary psychotherapy service	-<br>Psychological support<br>• Group cognitive analytic therapy	SVA (CSA)	6
Clarke & Llewelyn (1994)	Peer review	Pre-post	Public: NHS	-<br>Psychological support<br>• Cognitive analytic therapy	SVA (CSA)	4
Clarke & Pearson (2000)	Peer review	Pre-post	Public: NHS	-<br>Psychological support<br>• Cognitive analytic therapy	SVA (CSA)	4
Cook et al (1991)	Peer review	Pre-post	Public	-<br>Perpetrator programme<br>• Group treatment for sex offenders	SVA	1
Co-ordinated Action Against Domestic	Grey lit.	Data-set / service evaluation	Third	-<br>Advocacy<br>• 14 IDVA services, with types of support	DVA	11

For peer review only

Study	Peer review or grey lit	Study design	Setting/ sector	Type of intervention	Type of abuse*	No. of outcomes
Abuse (2012)				including safety planning, support with MARAC, liaison and support with police, support with criminal court cases, liaison and support with probation, support with civil orders, support with housing, financial benefits advice and support, support with immigration, health and wellbeing advice and support, and support with children		
Coy and Kelly (2011)	Grey lit.	Service evaluation	Third	- Advocacy<br>• Four IDVAs in different settings (police station, hospital A&E department, a community based DV project, and a women only VAW organisation)	DVA	2
Dheensa & Penny (2019)	Grey lit.	Service evaluation	Third	- Advocacy<br>• Learning disability ISVA	SVA	1
Dobash et al (1999)	Peer review	Non-randomised comparative	Public	*"IDVPP"* and *"CHANGE"*<br>Perpetrator programme<br>• Two perpetrator programmes, both focused on challenging group work	DVA	3
Ellis (2012)	Peer review	Pre-post	Third?	*"The Butterfly Programme"*<br>Psychosocial support<br>• Group therapy	SVA (CSA)	6
Farrell & Buckley (1999)	Peer review	Pre-post	Public	*"The Domestic Violence Unit"*<br>Specialist DV police teams<br>• Police officers dedicated to dealing with DV cases with specialist training.	DVA	5
Ferrari et al (2018)	Peer review	Randomised controlled trial	Third	*"PATH"*<br>Advocacy<br>• Specialist psychological advocates providing	DVA	6

For peer review only

Study	Peer review or grey lit	Study design	Setting/ sector	Type of intervention	Type of abuse*	No. of outcomes
				1:1 support on top of regular advocacy provision		
Friendship et al 2003	Peer review	Pre-post with retrospectively selected comparison group	Public: Prisons	*"SOTP"*<br>Perpetrator programme<br>• Two programmes based on the cognitive behavioural model, using group sessions	DVA	3
Garry & Munro (2020)	Grey lit.	Service evaluation	Third	*"The Rape and Sexual Violence Project (RSVP) adult counselling service"*<br>Psychological support<br>• Face-to-face counselling. Clients can also access RSVPs other support services	SVA	1
Geoghegan-Fittall et al (2020)	Grey lit.	Service evaluation	Third	*"The Croydon Drive Project"*<br>Perpetrator programme<br>• Direct and indirect interventions and activity disruption. Interventions may be singular or concurrent	DVA	3
Gilchrist et al (2021)	Peer review	Randomised controlled trial	Mixed: NHS and voluntary sector	*"ADVANCE"*<br>Perpetrator programme<br>• Manualised evidence-informed tailored intervention focusing on developing healthy relationships	DVA	14
Goosey et al (2017)	Peer review	Randomised controlled trial	Mixed: community safety team, police, drug and alcohol service, Thames Valley Probation, Youth Offending	-<br>Multi-service<br>• Integrated cased management using the Domestic Abuse Service Coordination model, including support for victims, counselling for perpetrators, and follow up	DVA	1

For peer review only

Study	Peer review or grey lit	Study design	Setting/ sector	Type of intervention	Type of abuse*	No. of outcomes
			Service, Domestic Abuse Perpetrator Service, Berkshire Women's Aid, Bracknell Forest Homes	police visits		
Halliwell et al (2019)	Peer review	Non-randomised comparative	Mixed: Public and Third	- Advocacy<br>• A hospital based IDVA who provided support and advice, risk assessment and safety planning, referral, partnership work and training hospital staff	DVA	8
Hester & Westmarland (2005)	Grey lit.	Service evaluation	Mixed: Third, public	- Multiple / multi-service<br>• 27 domestic violence projects split in to seven packages (criminal and civil justice, protection and prevention, Black and other ethnic minorities, health, multi-service, education and rural work). A sub selection of the 27 that report relevant outcomes and use the included methodology were extracted, including: Bradford Staying Put, Hammersmith and Fulham Standing Together, Northampton Sunflower Centre, Cheshire and Taunton – use of photographic evidence, Croydon Domestic Violence Advocate Service, Northampton Sunflower Centre	DVA	17
Hester et al (2019)	Grey lit	Service evaluation using RCT design	Third	*"Drive"*<br>Perpetrator programme<br>• Intensive case management and multi-agency response. Uses disruption, support and behaviour change interventions	DVA	10

For peer review only - http://bmjopen.bmj.com/site/about/guidelines.xhtml

Study	Peer review or grey lit	Study design	Setting/ sector	Type of intervention	Type of abuse*	No. of outcomes
				alongside protective work<br>Advocacy<br>• One-to-one IDVA support for the associated victims-survivors		
Howarth and Robinson (2016)	Peer review	Pre-post	Unclear	-<br>Advocacy<br>• Seven IDVA services. Support provided included safety planning, housing and accommodation, child-related support, access to justice systems, health and wellbeing, and welfare and immigration support	DVA	4
Howarth et al (2009)	Grey lit	Service evaluation	Third	-<br>Advocacy<br>• Seven IDVA services in a range of rurality's, sizes and age of service	DVA	13
Karatzias et al (2016)	Peer review	Pre-post	Public: NHS	*"Trauma Recovery and Empowerment Model"*<br>Psychological support<br>• Group psychotherapy using cognitive behavioural and skills training techniques	DVA<br>SVA<br>(CSA)	5
Kelly et al (1999)	Grey lit.	Service evaluation	Public	*"Domestic Violence Matters"*<br>Specialist domestic violence police teams<br>• Five support workers who worked alongside the police to support women where an arrest of a perpetrator occurred.	DVA	5
Kelley et al (2022)	Peer review	Non-randomised comparative	Private: Privately owned prison	*"Principle-based correctional counselling"*<br>Perpetrator programme<br>• Classes including modules on building rapport, exploration of reality, exploration of thought, exploration of feelings/behaviour.<br>• All participants also participated in the	SVA	6

Study	Peer review or grey lit	Study design	Setting/ sector	Type of intervention	Type of abuse*	No. of outcomes
				required CBT based Sexual offender Treatment programme		
Kelly & Westmarland (2015)	Grey lit.	Service evaluation	Third	*"DVPP"*<br>Perpetrator programme<br>• Twelve Respect accredited DVPPs. All have manuals and involved group work.<br>Unclear – Women's support service<br>• Each perpetrator programme had an integrated women's support service, however no further details are provided.	DVA	7
Koppensteiner et al (2019)	Grey lit.	Randomised controlled trial	Public: Police	*"Project 360"*<br>Specialist domestic violence police teams<br>• Engagement workers who contact victims and offer further assistance, including information on legal options, referrals to support services, and safety planning	DVA	1
Lindsay et al (2011)	Peer review	Pre-post	Unclear	-<br>Perpetrator programme<br>• A sex offender treatment programme, including modules on disclosure, pathways into offending, cognitive distortions, childhood abuse, victim awareness, relapse prevention, and adaptive attachment	SVA	2
Lindsay et al. (1998)	Peer review	Pre-post	Public: Psychology / psychiatry department	-<br>Perpetrator programme<br>• Group sessions focused on sex offending and cognitive change, including taking responsibility, victim awareness and behaviours consistent with offending	SVA	1
Lowe et al (2017)	Peer review	Pre-post	Third	-<br>Psychological support	SVA (CSA)	3

For peer review only - http://bmjopen.bmj.com/site/about/guidelines.xhtml

Study	Peer review or grey lit	Study design	Setting/ sector	Type of intervention	Type of abuse*	No. of outcomes
				• Group therapy. Sessions used a person centred, psychoeducational approach with elements of cognitive behavioural therapy		
McConnell et al (2017)	Peer review	Pre-post	Third	*"Caring Dads Safer Children"*<br>Perpetrator programme<br>• Includes seven treatment targets: anger/hostility; family cohesion/ coparenting/domestic violence; perceptions of the child as a problem; use of corporal punishment and other aversive behaviours; positive and involved parent-child relationship; self-centeredness; and misuse of substances	DVA	4
McCracken & Deave (2012)	Grey lit.	Service evaluation	Third	*"Caring Dads Cymru"*<br>Perpetrator programme<br>• Group work programme based on cognitive behavioural therapy and motivational models of intervention	DVA	4
Michie & Lindsay (2012)	Peer review	Non-randomised comparative	Public: NHS	-<br>Perpetrator programme<br>• Cognitive behavioural therapy with an empathy component	SVA	1
Morgan et al (2019)	Peer review	Pre-post	Third	*"RADAR/ADAPT"*<br>Perpetrator programme<br>• A multiagency initiative that provides a tiered needs-led approach. It provides a programme for perpetrators and support for victims	DVA	4
Morrison & Treliving (2002)	Peer review	Pre-post	Public: Local psychotherapy service	-<br>Psychological support<br>• Group therapy aiming to provide men with a	SVA (CSA)	1

Study	Peer review or grey lit	Study design	Setting/ sector	Type of intervention	Type of abuse*	No. of outcomes
				safe space to explore their difficulties and the impact of childhood sexual abuse		
Murphy et al (2007)	Peer review	Pre-post	Public: Intellectual disability services	-<br>Perpetrator programme<br>• Group cognitive behavioural therapy for sexually abusive men	SVA	4
Oasis (2016)	Grey lit	Service evaluation	Third	-<br>Multi-service<br>• Counselling (6 sessions plus more if required)<br>• Refuge: a 12-bed refuge providing temporary emergency accommodation. Staff provide emotional and practical support	DVA	3
Ormston et al (2016)	Grey lit.	Service evaluation	Third	*"The Caledonian System"*<br>Perpetrator programme<br>• Perpetrator programme covering lifelong change, responsibility for and to self, relationships, sexual respect, men and women, and children and fathering.<br>Multi-service<br>• Women's service including safety planning, information, advice, and emotional support to (ex)partners	DVA	6
Payne et al (2007)	Peer review	Pre-post	Public (assumed as authors are clinical psychologists)	-<br>Psychological support<br>• Client centred group therapy	DVA<br>SVA<br>(CSA)	3
Peckham et al (2007)	Peer review	Pre-post	Public: local community intellectual disability service	-<br>Psychological support<br>• Survivors group, involving educating clients and carers about sexual abuse, and weekly sessions using drawings, videos, dolls and pictures	SVA	6

For peer review only

Study	Peer review or grey lit	Study design	Setting/ sector	Type of intervention	Type of abuse*	No. of outcomes
Robinson (2003)	Grey lit.	Service evaluation	Mixed: Public (police, criminal justice) and third sector	*"Women's Safety Unit"*<br>Multi-service<br>• Advocacy, specialist counselling, legal services, housing services, refuge provision, target hardening and collecting evidence	DVA<br>SVA	11
Robinson (2004)	Grey lit.	Service evaluation	Mixed: Public (police, probation, health, WSU, social services, housing, health) and third sector	-<br>MARAC<br>• Multiagency meeting which delegates actions to workers. Agencies include police, social services, probation, health, and education (where relevant)	DVA	6
Robinson (2006)	Peer review	Pre-post	Mixed: Police, probation, health, WSU, social services, housing, health, third sector	-<br>MARAC<br>• Monthly multiagency meetings to discuss high risk victims, to share information and take actions to reduce future harm.	DVA	6
Robjant et al (2017)	Peer review	Pre-post	Third	-<br>Psychological support<br>• Narrative exposure therapy, a therapy specifically designed for victims of trauma	SVA	2
Rose et al (2012)	Peer review	Pre-post	Public: Community Learning Disability Services	-<br>Perpetrator programme<br>• Group programme with topics including sex education, emotion recognition, cognitive distortions, motivation for offences, empathy and relapse prevention	SVA	4
Ross et al. (2022)	Peer review	Non-randomised comparison	Public: Magistrate's Court	-<br>Advocacy<br>• An IDVA service based at a Specialist	DVA	4

For peer review only

Study	Peer review or grey lit	Study design	Setting/ sector	Type of intervention	Type of abuse*	No. of outcomes
				Domestic Abuse Court		
Ryan et al (2005)	Peer review	Randomised controlled trial	Public: NHS psychology departments	- Psychological support<br>• Individual and group therapy, focused on experiences and consequences of CSA	SVA (CSA)	4
SafeLives	Grey lit.	Dataset / service evaluation	Third	- Refuge<br>• No information	DVA	12
SafeLives	Grey lit.	Dataset / service evaluation	Third	- MARAC<br>• No information	DVA	3
SafeLives	Grey lit.	Dataset / service evaluation	Third	- Sexual violence services<br>• No information	SVA	19
SafeLives	Grey lit.	Dataset / service evaluation	Third	- Health services<br>• No information	DVA	12
Safelives	Grey lit.	Dataset / service evaluation	Third	- Helpline<br>• No information	DVA	11
SafeLives	Grey lit.	Dataset / service evaluation	Third	- IDVA<br>• No information	DVA	12
SafeLives	Grey lit.	Dataset / service evaluation	Third	- Outreach<br>• No information	DVA	12
Sharpe et al	Peer review	Pre-post	Public:	*"The Male Childhood Sexual Abuse Group"*	SVA	2

For peer review only

Study	Peer review or grey lit	Study design	Setting/ sector	Type of intervention	Type of abuse*	No. of outcomes
(2001)			Psychological therapies service	Psychological support<br>• Group therapy, no further information	(CSA)	
Skyner & Waters (1999)	Peer review	NR	Mixed: Public (probation service) and third sector (NSPCC)	*"The Domestic Violence Prevention Programme"*<br>Perpetrator programme<br>• A cognitive behavioural programme with a variety of learning styles. A parallel support package is provided to (ex)partners and their children throughout	DVA	4
Smith et al (1995)	Peer review	Pre-post	Public: Specialised therapy unit	*"Breakfree"*<br>Psychological support<br>• Individual therapy, daytime drop in facility, telephone contact, out-of-hours paging service, limited befriending service and a 'time out' facility where a client can stay in a safe environment for 1-2 nights	SVA (CSA)	3
Smith et al (2015)	Peer review	Pre-post	Third	*"Domestic Abuse Recovering Together (DART)"*<br>Psychological support<br>• Mother and child sessions, designed to strengthen their relationship and support recovery. Followed by separate peer group sessions	DVA	3
Stanley et al (2011)	Grey lit.	Service evaluation	Mixed: Third and Public (NHS, City Council)	*"Strength to change"*<br>Perpetrator programme<br>• Individual and group sessions over 40 weeks. Advocacy and advice are offered where appropriate. Sessions include mindfulness techniques, cognitive approaches, counselling, behavioural techniques.<br>• A women's service is available for (ex)partners offering practical and emotional support	DVA	4

For peer review only

Study	Peer review or grey lit	Study design	Setting/ sector	Type of intervention	Type of abuse*	No. of outcomes
Stanley et al (2021)	Grey lit	Service evaluation	Third	*"VOICES"*<br>Multi-service<br>• Trauma informed approach. Practitioners were given a new assessment framework, training and planning tools. No information on the specific types of activities or interventions is reported	DVA	6
Stanley et al (2021)	Grey lit	Service evaluation	Third	*"SafeLives Co-Designed Pilots (SLCDPs)"*<br>Multi-service<br>• A suite of interventions for survivors, their children and perpetrators, using a whole family approach. This included community IDVA support, complex needs IDVA support, step down and recovery groups, children and young peoples work and 'Engage', which worked with the whole family	DVA	5
Strang et al (2017)	Peer review	Randomised controlled trial	Mixed: Public (police), third sector	*"CARA"*<br>Perpetrator programme<br>• A two-day offender workshop, focusing on raising awareness, and moving offenders from denial and minimisation to acceptance and responsibility of harm, and conflict resolution	DVA	3
Taylor-Dunn & Erol (2019)	Grey lit	Service evaluation	Third	-<br>Advocacy<br>• Intensive one-to-one IDVA support within an immediate to medium term time-frame, implementing individual support plans and risk assessments to reduce risk and encourage recovery support	DVA	5
The Institute	Grey lit.	Service	Third	*"SHE"*	DVA	5

For peer review only

Study	Peer review or grey lit	Study design	Setting/ sector	Type of intervention	Type of abuse*	No. of outcomes
for Public Safety, Crime & Justice, University of Northampton (2019)		evaluation		Psychological support<br>• A 'minimum support service (MSS)' with regular telephone check-ins, with an emphasis on safety and wellbeing, and an 'integrated support service (ISS+)' offering a face-to-face person-centred programme<br>Perpetrator programme<br>• 'Evolve', a programme domestic violence perpetrator programme working towards Respect accreditation		
Trevillon et al (2014)	Peer review	Non-randomised comparative	Public: Community mental health team	-<br>Advocacy<br>• Domestic violence training for clinicians, domestic violence manual for clinicians, mental illness training for domestic violence advisors, direct referral pathway to domestic violence advocacy, provision of a domestic violence advocacy service, comprising emotional and practical support, domestic violence education, support groups, safety planning and legal/housing support	DVA	11
Vallely et al (2005)	Grey lit.	Service evaluation	Mixed: Public (Crown Prosecution Service, Police, Magistrates' Court, Probation service, local authorities) and Third sector	-<br>Specialist domestic violence court<br>• The service included a dedicated sitting for pre-trial matters and sentencing, allocated weekly; specialist magistrates with domestic violence training, specially trained CPS lawyers, and an independent advocacy service	DVA	5
Webster (2015)	Grey lit.	Service evaluation	Third	*"Kent and Medway IDVA"*<br>Advocacy<br>• Up to 21 IDVAs working with domestic abuse victims. No further information.	DVA	2

For peer review only

Study	Peer review or grey lit	Study design	Setting/ sector	Type of intervention	Type of abuse*	No. of outcomes
Westmarland & Alderson (2013)	Peer review	Pre-post	Third	-<br>Psychological support<br>• Face-to-face counselling, following a woman-centred, empowerment model, with elements of cognitive behavioural therapy, goal setting and rational emotive behaviour therapy	SVA	1
Williamson & Boyle (2012)	Peer review	Pre-post	Public: NHS	*"IDVA Advocacy Intervention Programme"*<br>Advocacy<br>• No details reported	DVA	2
Williamson & Hester (2009)	Grey lit.	Service evaluation	Third	*"South Tyneside Domestic Abuse Perpetrator Programme"*<br>Perpetrator programme<br>• A 'pro-feminist broadly cognitive behavioural model combined with gender analysis' approach. Group and one-to-one sessions.	DVA	2
Women's Aid (2022)	Grey lit.	Service evaluation	Third	*"No Woman Turned Away"*<br>Housing (specialist caseworkers)<br>• Caseworkers provide telephone and email support to women looking for a refuge space	DVA	1
Women's Resource Centre (2011a)	Grey lit.	Service evaluation	Third	*"Ashiana"*<br>Multi-service<br>• Refuge: Safe accommodation across three schemes for South Asian, Turkish and Iranian women. Residents can stay for up to 1 year.<br>• Outreach: To provide support and reduce repeat victimisation. Ashiana also runs a DV support group for women in the wider community, as well as an end of therapy support group.	DVA	11

For peer review only

Study	Peer review or grey lit	Study design	Setting/ sector	Type of intervention	Type of abuse*	No. of outcomes
Women's Resource Centre (2011b)	Grey lit.	Service evaluation	Third	*"Women and Girls Network"*<br>Psychological support<br>• Counselling using trauma therapy, cognitive behavioural therapy, and integrated person-centred and feminist counselling practice. The organisation also offers a telephone helpline, body therapies, group programmes and specialist support projects	DVA	1

For peer review only

For peer review only

Appendix 5: Outcome measures by domain and subdomain

Category	Domain	Subdomain	Measure	N
**Health**	**Mental health**	**Depression**		**16**
			Beck Depression Inventory	6
			Hospital Anxiety and Depression Scale - Depression	4
			Patient Health Questionnaire	3
			PROMIS depression Short Form	1
			Single item – "Is the client experiencing depression?"	1
			Single item – reduction in feeling depressed	1
		**Anxiety**		**10**
			Hospital Anxiety and Depression Scale - Anxiety	4
			Generalized Anxiety Disorder Scale	3
			PROMIS Anxiety Short Form	1
			Spiel State Anxiety	1
			Single item – "Is the client experiencing anxiety?"	1
		**Trauma**		**12**
			Primary care PTSD screen	2
			Impact of Event Scale	2
			PTSD Checklist for DSM-5 (PCL-5)	1
			PTSD Symptom Scale	1
			Posttraumatic Diagnostic Scale	1
			Post-traumatic Stress Disorder Scale	1
			Changes in Outlook Questionnaire	1
			The Clinician Administered PTSD Scale	1
			PTSD Checklist (PCL-C)	1
			Single item – "I have "flashbacks" about what happened"	1
		**General mental health**		**4**
			General Health Questionnaire (GHQ-28)	2

For peer review only

Category	Domain	Subdomain	Measure	N
			Single item - "Current mental health rating"	1
			No information – reported outcome is "Improved mental health"	1
		**Psychological symptoms**		**19**
			The Clinical Outcomes in Routine Evaluation - Outcome Measure (CORE-OM)	5
			The Symptom Checklist 90 revised (SCL-90R)	4
			Brief Symptom Inventory	2
			Jehu Beliefs Inventory	2
			The Shutdown Dissociation Scale (Shu-Dis)	1
			Dissociative Experiences Scale (DES)	1
			Belief Inventory	1
			Social Activity and Distress Scale	1
			Delusions, Symptoms and States Inventory	1
			Single item – "Is the client experiencing emotional instability?"	1
		**Anger**		**2**
			The Novaco Anger Scale	1
			PROMIS Anger Short Form	1
		**Understanding feelings**$		2
			Three Principles Inventory$	1
			Single item - "Understanding feelings better"$	1
		**Self-Harm / Suicide**$		**5**
			Single item – reduction in experiencing suicidal thoughts or feelings$	1
			Single item – reduction in self harm$	1
			Single item – "Is the client experiencing self-harm?" $	1
			Single item – "Is the client experiencing suicidal thoughts?" $	1
			Single item – "Is the client experiencing suicidal behaviours?" $	1
		**Healthcare utilisation / resource use**$		**5**
			Number / percent of participants using healthcare resources (e.g., A&E, GP, psychiatrist)$	3
			Number / percent of participants using community health and social service resources (e.g., health visitor, drug worker) $	1
			Days of admission$	1

For peer review only

Category	Domain	Subdomain	Measure	N
		**Mental wellbeing**[$]		**4**
			Short Warwick–Edinburgh Mental Wellbeing Scale (SWEMWBS)[$]	4
				2
		**Self-care**[$]	Single item - "Is the client experiencing trouble sleeping?"[$]	1
			Single item - "Is the client experiencing problems with eating?"[$]	1
				**3**
		**Quality of life**[$]	Short Form 12 (SF12)[$]	2
			Manchester Short Assessment of Quality of Life[$]	1
		**Health, mental health and wellbeing**[$]		**1**
			Taking Back Control Tool[$]	1
	**Physical health**	**Self-reported physical health**		**8**
			EQ-5D	3
			Visual analogue scale for physical health	2
			Single item – "Current physical health (poor/fair/good/very good/excellent)"	1
			No information – reported outcome is "Improved physical health (DV specific)"	1
			No information – reported outcome is "Improved physical health (not DV specific)"	1
		**Alcohol and drug use**		**5**
			Alcohol Use Disorders Identification Test (AUDIT)[$]	1
			Single item – "Current drug use (excessive/high/moderate/low/none)"[$]	1
			Single item – "Current alcohol use (excessive/high/moderate/low/none)"[$]	1
			Single item – "Alcohol problem (yes/no)"[$]	1
			Single item – "Drug problem (yes/no)"[$]	1
		**Healthcare utilisation / resource use**[$]		**5**
			Number / percent of participants using healthcare resources (e.g., A&E, GP, psychiatrist)[$]	3
			Number / percent of participants using community health and social service resources (e.g., health visitor, drug worker)[$]	1
			Days of admission[$]	1
		**Quality of life**[$]		**3**

For peer review only

Category	Domain	Subdomain	Measure	N
			Short Form 12 (SF12) $	2
			Manchester Short Assessment of Quality of Life$	1
		**Health, mental health and wellbeing$**		**1**
			Taking Back Control Tool$	1
	**Wellbeing**	**Wellbeing**		**7**
			Outcome Star	1
			Adult wellbeing scale	1
			Benefits of Therapy scale	1
			Personal Outcomes and Wellbeing Record (POWeR)	1
			Victim survey (perceived wellbeing - safety and wellbeing)	1
			Wellbeing survey (9 domains)	1
			CORE monitoring form	1
			Perceived Severity of Problems/Concerns Questionnaire	1
		**Social ties/ relationships**		**5**
			Social Inclusion Scale$	1
			Inventory of interpersonal problems 32 (IIP-32) $	1
			Single item - "Current social and community support (no social contact / negative social contacts / some positive contacts / majority positive contacts / all positive)" $	1
			No information – reported outcome is "Improved relationship with family"$	1
			No information – reported outcome is "Improved social support"$	1
		**Understanding feelings**		**2**
			Three Principles Inventory$	1
			Single item - "Understanding feelings better"$	1
		**Mental wellbeing$**		**4**
			Short Warwick–Edinburgh Mental Wellbeing Scale (SWEMWBS) $	4

For peer review only

Category	Domain	Subdomain	Measure	N
				**2**
		**Self-care**[$]	Single item - "Is the client experiencing trouble sleeping?" [$]	1
			Single item - "Is the client experiencing problems with eating?" [$]	1
				**3**
		**Quality of life**[$]	Short Form 12 (SF12) [$]	2
			Manchester Short Assessment of Quality of Life[$]	1
		**Health, mental health and wellbeing**[$]		**1**
			Taking Back Control Tool[$]	1
**Behavioural**	**Behavioural**	**Challenging behaviour**		**1**
			Challenging Behaviour Inventory	1
		**Self-Harm / Suicide**[$]		**5**
			Single item – reduction in experiencing suicidal thoughts or feelings[$]	1
			Single item – reduction in self harm[$]	1
			Single item – "Is the client experiencing self-harm?" [$]	1
			Single item – "Is the client experiencing suicidal thoughts?" [$]	1
			Single item – "Is the client experiencing suicidal behaviours?" [$]	1
		**Alcohol and drug use**[$]		**5**
			Alcohol Use Disorders Identification Test (AUDIT) [$]	1
			Single item – "Current drug use (excessive/high/moderate/low/none)" [$]	1
			Single item – "Current alcohol use (excessive/high/moderate/low/none)" [$]	1
			Single item – "Alcohol problem (yes/no)" [$]	1
			Single item – "Drug problem (yes/no)" [$]	1
**Empowerment**	**Empowerment**	**Self-esteem**		**12**
			Rosenberg's self-esteem scale	7
			Culture Free Self Esteem Inventory	2
			The Self Concept Questionnaire	1
			Self-esteem Assessment Tool	1
			No information – reported outcome is "Improved self-esteem"	1
		**Coping and**		**3**

For peer review only

Category	Domain	Subdomain	Measure	N
		**resilience**	Resilience Research Centre Adult Resilience Measure (RRC-ARM)	1
			Cope and Recover Questionnaire	1
			No information – reported outcome is "Improved coping"	1
		**Locus of control**		**1**
			Review of Personal Effectiveness with Locus of Control	
		**Empowerment**		**5**
			The Empowerment Star	2
			Measure of Victim Empowerment Related to Safety Scale (MOVERS)	1
			Coping and confidence outcome measure	2
		**Assertiveness**		**1**
			Assertiveness Questionnaire	1
		**Knowledge**		**1**
			Goal Attainment Scale	1
		**Social ties/ relationships$**		**5**
			Social Inclusion Scale$	1
			Inventory of interpersonal problems 32 (IIP-32) $	1
			Single item - "Current social and community support (no social contact / negative social contacts / some positive contacts / majority positive contacts / all positive)" $	1
			No information – reported outcome is "Improved relationship with family"$	1
			No information – reported outcome is "Improved social support"$	1
		**Understanding feelings$**		**2**
			Three Principles Inventory$	1
			Single item - "Understanding feelings better"$	1
				2
			No information – reported outcome "Independence"	1
		**Independence**		
			No information – reported outcome "Increased likelihood of employment "	1
		**Awareness of**		1

For peer review only

Category	Domain	Subdomain	Measure	N
		**support**		
			Number of criminal justice contacts	1
**Socio-economic circumstances**	**Socio-economic circumstances**	**Employment/ education**		**5**
			Single item - "Employment status (FT employed/PT employed/unemployed/caring for children/family)"	1
			Single item - "Employment status (paid employment/voluntary work/education/retired)"	1
			Single item - "Returned to/begun employment or education"	1
			Single item - "Education, employment and training (none / occasional / some sustained / sustained / sustained and fulfilling)"	1
			No information – reported outcome "Increased likelihood of employment"[$]	1
		**Finances**		**3**
			No information – reported outcome "Financial savings"	1
			Single item - "Perception of income (high/comfortable/managing with treats/managing with occasional treats/essentials only/struggling)"	1
			Single item - "Current financial situation (significant financial problems/being reliant on others/unable to pay for essentials/managing essentials but nothing left over/comfortably managing and no financial concerns)"	1
		**Housing**		**4**
			No information – reported outcome "Access to housing"	1
			Single item – "Current housing (homeless / transitory / unsuitable / temporary / secure)"	1
			Single item – "Number of women accommodated in suitable refuge spaces"	1
			Number / percent of participants using supported accommodation resources (e.g., weeks spent in refuge / staffed accommodation / B&B, boarding house or hotel / homeless and living with friends or family)	1
				**1**
				5
		**Social ties/ relationships**[$]	Social Inclusion Scale[$]	1
			Inventory of interpersonal problems 32 (IIP-32) [$]	1

For peer review only

Category	Domain	Subdomain	Measure	N
			Single item - "Current social and community support (no social contact / negative social contacts / some positive contacts / majority positive contacts / all positive)" $	1
			No information – reported outcome is "Improved relationship with family"$	1
			No information – reported outcome is "Improved social support"$	1
**Changing perpetrator behaviour**	**Changing perpetrator behaviour**	**Anger**		**1**
			The Novaco Anger Scale	1
		**Perpetrator needs**		**1**
			Distribution of needs	1
		**Attitudes to women / violence**		**20**
			Inventory of Beliefs About Wife Beating	1
			Questionnaire on Attitudes Consistent with Sexual Offending (QACSO)	3
			Socio-Sexual Knowledge and Attitudes Assessment – Revised (SSKAAT-R)	1
			The Intimate Partner Violence Responsibility Attribution Scale (IPVRAS)	1
			URICA-DV Readiness to change	1
			Propensity for Abusiveness Scale (PAS)	2
			Paulhus Deception Scale (PDS)	1
			Interpersonal Reactivity Index (IRI)	1
			Sexual Attitudes and Knowledge Scale	1
			Victim Empathy Scale-Adapted	1
			Sexual Offenders Self Appraisal Scale	1
			Treatment motivation Questionnaire	1
			Interpersonal Dependency Inventory	1
			Attitudes Toward Exhibitionism Questionnaire	1
			No information - "Attitudes to the offence"	1
			No information - "Attitudes towards women"	1
			No information - "Acceptance of responsibility"	1
		**Desirable responding**		**3**
			Balanced Inventory of Desirable Reporting	3
		**Locus of control / self-control**		**6**
			Multidimensional Locus of Control Scale	1
			Rotter's Locus of Control Scale	1
			Brief self-control scale (BSCS)	1

Category	Domain	Subdomain	Measure	N
			Low self-control scale (LSCS)	1
			Levenson Locus of Control	1
			The Nowicki-Strickland Locus of Control Scale	1
		**Reasons for violence**		**2**
			Number of reasons for violence given	1
			Number of participants reporting each of 7 potential reasons for abuse	1
**Victim-perpetrator relationship**	**Victim-perpetrator relationship**	**Victim-perpetrator relationship**		**12**
			The Communication Patterns Questionnaire-Short Form (CPQ-SF)	1
			Respectful communication	1
			Awareness of self and others	1
			Attitude to partner (self-partner correlation)	1
			Single item - "Relationship status"	1
			Single item - "Hopes for the relationship"	1
			Single item - "Living circumstances (with perpetrator / not living together / intermittently living together)	6
**Parenting**	**Parenting**	**Parenting**		**7**
			Parent Empowerment and Efficacy Measure (PEEM)	1
			Camberwell Assessment of Need for Mothers (Short version)	1
			Parenting Stress Index	2
			Parental Acceptance-Rejection Questionnaire (PARQ)	2
			The Parental Locus of Control Scale	1
**DSVA**	**Experience of DSVA**	**Impact of abuse**		**2**
			Number of participants experiencing each of 18 potential impacts of abuse	1
			Mean number of impacts of abuse	
		**Indicators of ongoing abuse**		**5**
			Additional MARACs/Reappearance at MARAC	3
			Other evidence of abuse (e.g. harassing texts)	1
			Cases reopened	1
		**Perceived safety**		**7**
			Safety Behaviour Checklist	1

For peer review only

Category	Domain	Subdomain	Measure	N
			Single item - "Partner fearful (1= never to 4=often)"	1
			Single item - "Feeling of safety (much safer / somewhat safer / no change / less safe)"	1
			Safety (6 items)	2
			Single item - "How safe do you feel? (not safe / a little unsafe / somewhat safe / very safe)	1
			No information - reported outcome is "increased safety"	1
			No information - reported outcome is "avoidance of forced marriage"	1
		**Revictimization**		**4**
			Average number of repeat victims per month	1
			Number / percent of repeat victims	1
			Number / percent of women reporting repeat victimisation (for IPA)	1
			Number / percent of women disclosing further incidents of violence to IDVA	1
		**Presence of abuse**[$]		**46**
			Severity of Abuse Grid - presence: Number / percent of people responding yes to the presence of abuse (reported for physical abuse, sexual abuse, harassment and stalking and jealous and controlling behaviour)[$]	10*
			Severity of Abuse Grid - cessation: Number / percent of people reporting no abuse[$]	11*
			Severity of Abuse Grid - reduction in presence: Number / percent of people reporting a reduction of abuse (reported for physical abuse, sexual abuse, harassment and stalking and jealous and controlling behaviour)[$]	1
			Severity of Abuse Grid - multiple types: Number / percent of people reporting multiple types of abuse[$]	7*
			Number / percent of participants experience concurrent domestic abuse as well as sexual abuse[$]	1
			Number / percent of participants who have been subjected to adult sex offences[$]	1
			Number / percent of men abusing each month[$]	1
			Space for Action Survey (% responding yes to 12 indicators)[$]	1
			Physical and Sexual Violence Survey (% responding yes to 7 indicators)[$]	1
			Harassment and other abusive acts survey (% responding yes to 6 indicators)[$]	1
			Fathering survey / using children against a partner (% responding yes to 5	2

Category	Domain	Subdomain	Measure	N
			indicators) $	
			Social media perpetration / victimisation survey$	1
			Composite Abuse Scale$	3
			Abusive Behaviour Inventory$	2
			Controlling Behaviour Inventory$	3
		**Number of abusive incidents or behaviours$**		**4**
			Mean number of abusive incidents per man$	1
			Mean number of abusive behaviours reported by participants$	1
			Number / percent of participants experiencing between 0 and 7 behaviours (reported for emotional, physical and sexual behaviours) $	1
			IMPACT monitoring toolkit (reported for emotional, physical, and sexual behaviours) $	1
		**Severity of abuse$**		**29**
			Severity of Abuse Grid - severity: Number / percent of participants reporting high / moderate / standard severity (reported for physical abuse, sexual abuse, harassment and stalking and jealous and controlling behaviour) $	8*,±
			Severity of Abuse Grid - reduction in severity: Number / percent of participants reporting a reduction of high / moderate / standard / unknown severity (reported for physical abuse, sexual abuse, harassment and stalking and jealous and controlling behaviour) $	1
			Severity of Abuse Grid – escalation in severity: Number / percent of participants reporting escalation in severity of abuse$	2
			Severity of Abuse Grid - severity: Number / percent of participants reporting any form of severe abuse$	2
			Severity of Abuse Grid - high severity: Number / percent of participants reporting severe abuse (reported for physical abuse, sexual abuse, harassment and stalking and jealous and controlling behaviour) $	1
			Severity of Abuse Grid - severity: Number / percent of participants reporting multiple types of abuse that are high severity$	6*
			Severity of Abuse Grid - severity: Number / percent of participants reporting at least one type of abuse that is high severity$	6*
			Cambridge Crime Harm Index$	3

For peer review only

Category	Domain	Subdomain	Measure	N
		**Frequency of abuse**[$]		**14**
			Severity of Abuse Grid - frequency: Number / percent of participants reporting high / moderate / standard / unknown frequency (reported for physical abuse, sexual abuse, harassment and stalking and jealous and controlling behaviour) [$]	5*
			Severity of Abuse Grid - frequency escalation: Number / percent of participants reporting an escalation in frequency[$]	6*
			Frequency of abusive behaviours (scale 1 = never -3 = often) [$]	1
			Frequency rating of past violence (none (1) -> frequent (10) [$]	1
			Frequency rating of current violence (none (1) -> frequent (10) [$]	1
		**Duration of abuse**[$]		**1**
			Number / percent of victims with incidents recorded over a period longer than 12 months[$]	1
		**Severity and frequency of abuse**[$]		**13**
			Severity of Abuse Grid - severity and frequency escalation: Number / percent of participants reporting any escalation in severity or frequency of abuse[$]	7*
			Severity of Abuse Grid - severity and frequency escalation: Number / percent of participants reporting at least one form of abuse that is high severity and escalating in frequency or severity[$]	6*
		**Risk**[$]		**18**
			Spousal Assault Risk Assessment[$]	2
			DASH risk (high/med/standard) [$]	2
			Drive-DASH (mean score) [$]	1
			DASH risk (number with 1-14 assessments who had a decrease in score) [$]	1
			Change in risk in perpetrators (measurement tool unclear) [$]	1
			Weighted change in risk in perpetrators (measurement tool unclear) [$]	1
			IDVA perception of change in risk (do not know / increased / limited or no reduction / moderate reduction / significant reduction) [$]	1
			IDVA rated reduction in risk (percentage reporting 'some reduction in risk') [$]	1
			IDVA perception of sustainability of change in risk (Do not know / short term / medium term / long term / risk is permanently eliminated) [$]	1

For peer review only

Category	Domain	Subdomain	Measure	N
			Men's perception of risk of using violence again (never/not sure/possibly) [$]	1
			Risk factors - Perpetrators' crimogenic behaviour and aggravating problems (frequency and percent of 7 factors) [$]	1
			Risk factors - Specific features of abuse (frequency and percent of 10 factors) [$]	1
			Risk factors - Victims' appraisals of threat (frequency and percent of 3 factors) [$]	1
			Risk factors - Factors relating to children (frequency and percent of 3 factors) [$]	1
			Risk factors - Factors relating to victim (frequency and percent of 4 factors) [$]	1
			Probability of none / standard / moderate / and high abuse (reported for physical abuse / sexual abuse / harassment and stalking / jealous and controlling behaviour) [$]	1
		**Police incidents[$]**		**39**
			Average / total number of police incidents[$]	1
			Average / total number of DV related police incidents[$]	2
			Average / total number of incidents recorded by the police per month[$]	1
			Average number of DV complaints per month[$]	1
			Average number of arrests made per month[$]	1
			Average number of victims refusing to make a complaint per month[$]	1
			Average number of mental health related DV complaints per month[$]	1
			Average number of alcohol related DV complaints per month[$]	1
			Average number of drug related DV complaints per month[$]	1
			Average number of concern for children reports per month[$]	1
			Number / percent of participants that committed DV related police incidents[$]	1
			Number / percent of participants that committed (unspecified) police incidents[$]	1
			Number / percent of participants with (unspecified) arrests[$]	1
			Number / percent of calls to domestic incidents[$]	1
			Number / percent of repeat calls to domestic incidents[$]	1
			Number / percent of repeat calls to domestic violence incidents (number with 2 incidents, 3 incidents, 4 incidents… 14 incidents) [$]	1
			Number / percent of DV incidents where the perpetrator was not present[$]	1
			Number / percent of DV incidents that were crimed[$]	1

For peer review only

Category	Domain	Subdomain	Measure	N
			Number / percent of DV incidents that led to arrests$	2
			Number / percent of incidents with an arrest where a statement is given$	1
			Number / percent of incidents with an arrest and a statement is given where it is then retracted$	1
			Number / percent of crimes where perpetrator was charged/summonsed$	1
			Number / percent of police incidents before and after a MARAC$	1
			Number / percent of high / medium / low severity police incidents before and after a MARAC$	1
			Number / percent of women experiencing no further post-MARAC incidents of violence or abuse$	1
			Number / percent of women experiencing no further post-MARAC police complaints or call outs$	2
			Number / percent of women experiencing no further post-MARAC police complaints$	2
			Number / percent of women experiencing no further post-MARAC police call outs$	2
			Number / percent of women experiencing either additional post-MARAC police complaints or call outs$	1
			Number / percent of women experiencing both additional post-MARAC police complaints and call outs$	1
			Percent change in total calls to domestic incidents$	1
			Percent change in total calls to domestic incidents (as proportion of all calls) $	1
			Frequency of police call outs (never / once / 2-5 times / 6-10 times) $	1
			Cost of police incidents$	1
	**Perpetration of DSVA**	**Reoffending**		**20**
			Number / percent with proven reoffending (DV, core violence or any offence)	1
			Number / percent who reoffended (police incidents)	1
			Number / percent who committed further sex offences	2
			Number / percent who reoffended (no further details)	1
			Number / percent with repeat prosecution for violence against partner	1
			Number / percent with reconviction for another sexual offence	1
			Number / percent with reconviction for another violence offence	1

For peer review only

Category	Domain	Subdomain	Measure	N
			Number / percent with reconviction for any offence	2
			Number / percent linked to another crime (suspect or charged)	1
			Number / percent linked to another DVA crime (suspect or charged)	1
			Frequency of re-arrests for DVA	1
			Prevalence of re-arrests for DVA	1
			Number / percent of crimes that that were DVA related	1
			Number of incidents of DV whilst on the programme	1
			Number of incidents of non-DV crime whilst on the programme	1
			Number / percent of partners reporting subsequent violence	1
			Number / percent of partners reporting 'frequent violence' at follow up	1
			Time to reoffend	1
		**Criminal justice outcomes**		**16**
			Average number of people charged per month	1
			Average number of cases proceeding through court per month	1
			Average number of days between arrest and case finalisation	1
			Number / percent of cases discontinued	2
			Number / percent of cases bound over / other / found not guilty or absolute discharge / convicted	1
			Number / percent of victims retracting	1
			Number / percent of trials that went ahead on scheduled date	1
			Number / percent of trials that received a conviction	1
			Number / percent of charges that received a conviction	1
			Number / percent of guilty and not guilty pleas	1
			Number / percent of defendants who had the following sentences: conditional discharge / financial penalty / custody / community punishment order / community rehabilitation order	1
			Number / percent of cases with the following convictions: custodial sentence / non-custodial sentence / conditional discharge	1
			Number / percent of evidence types in CPS files (victim statement / police statement / defendant interview / witness statement / medical statement / case exhibits / 999 tape / forensic)	1

For peer review only

Category	Domain	Subdomain	Measure	N
			Improvements specific to the 'CPS Criteria for Success'	1
			Average monetary penalties (fines and compensation)	1
		**Presence of abuse$**		**46**
			Severity of Abuse Grid - presence: Number / percent of people responding yes to the presence of abuse (reported for physical abuse, sexual abuse, harassment and stalking and jealous and controlling behaviour) $	10*
			Severity of Abuse Grid - cessation: Number / percent of people reporting no abuse$	11*
			Severity of Abuse Grid - reduction in presence: Number / percent of people reporting a reduction of abuse (reported for physical abuse, sexual abuse, harassment and stalking and jealous and controlling behaviour) $	1
			Severity of Abuse Grid - multiple types: Number / percent of people reporting multiple types of abuse$	7*
			Number / percent of participants experience concurrent domestic abuse as well as sexual abuse$	1
			Number / percent of participants who have been subjected to adult sex offences$	1
			Number / percent of men abusing each month$	1
			Space for Action Survey (% responding yes to 12 indicators) $	1
			Physical and Sexual Violence Survey (% responding yes to 7 indicators) $	1
			Harassment and other abusive acts survey (% responding yes to 6 indicators) $	1
			Fathering survey / using children against a partner (% responding yes to 5 indicators) $	2
			Social media perpetration / victimisation survey$	1
			Composite Abuse Scale$	3
			Abusive Behaviour Inventory$	2
			Controlling Behaviour Inventory$	3
		**Number of abusive incidents or behaviours$**		**4**
			Mean number of abusive incidents per man$	1
			Mean number of abusive behaviours reported by participants$	1
			Number / percent of participants experiencing between 0 and 7 behaviours (reported for emotional, physical and sexual behaviours) $	1
			IMPACT monitoring toolkit (reported for emotional, physical, and sexual behaviours) $	1

For peer review only

Category	Domain	Subdomain	Measure	N
		**Severity of abuse**$		**29**
			Severity of Abuse Grid - severity: Number / percent of participants reporting high / moderate / standard severity (reported for physical abuse, sexual abuse, harassment and stalking and jealous and controlling behaviour) $	8*,±
			Severity of Abuse Grid - reduction in severity: Number / percent of participants reporting a reduction of high / moderate / standard / unknown severity (reported for physical abuse, sexual abuse, harassment and stalking and jealous and controlling behaviour) $	1
			Severity of Abuse Grid – escalation in severity: Number / percent of participants reporting escalation in severity of abuse$	2
			Severity of Abuse Grid - severity: Number / percent of participants reporting any form of severe abuse$	2
			Severity of Abuse Grid - high severity: Number / percent of participants reporting severe abuse (reported for physical abuse, sexual abuse, harassment and stalking and jealous and controlling behaviour) $	1
			Severity of Abuse Grid - severity: Number / percent of participants reporting multiple types of abuse that are high severity$	6*
			Severity of Abuse Grid - severity: Number / percent of participants reporting at least one type of abuse that is high severity$	6*
			Cambridge Crime Harm Index$	3
		**Frequency of abuse**$		**14**
			Severity of Abuse Grid - frequency: Number / percent of participants reporting high / moderate / standard / unknown frequency (reported for physical abuse, sexual abuse, harassment and stalking and jealous and controlling behaviour) $	5*
			Severity of Abuse Grid - frequency escalation: Number / percent of participants reporting an escalation in frequency$	6*
			Frequency of abusive behaviours (scale 1 = never -3 = often) $	1
			Frequency rating of past violence (none (1) -> frequent (10) $	1
			Frequency rating of current violence (none (1) -> frequent (10) $	1
		**Duration of abuse**$		**1**
			Number / percent of victims with incidents recorded over a period longer than 12 months$	1
		**Severity and frequency of abuse**$		**13**
			Severity of Abuse Grid - severity and frequency escalation: Number / percent of participants reporting any escalation in severity or frequency of abuse$	7*

For peer review only

Category	Domain	Subdomain	Measure	N
			Severity of Abuse Grid - severity and frequency escalation: Number / percent of participants reporting at least one form of abuse that is high severity and escalating in frequency or severity$	6*
		**Risk$**		**18**
			Spousal Assault Risk Assessment$	2
			DASH risk (high/med/standard) $	2
			Drive-DASH (mean score) $	1
			DASH risk (number with 1-14 assessments who had a decrease in score) $	1
			Change in risk in perpetrators (measurement tool unclear) $	1
			Weighted change in risk in perpetrators (measurement tool unclear) $	1
			IDVA perception of change in risk (do not know / increased / limited or no reduction / moderate reduction / significant reduction) $	1
			IDVA rated reduction in risk (percentage reporting 'some reduction in risk') $	1
			IDVA perception of sustainability of change in risk (Do not know / short term / medium term / long term / risk is permanently eliminated) $	1
			Men's perception of risk of using violence again (never/not sure/possibly) $	1
			Risk factors - Perpetrators' crimogenic behaviour and aggravating problems (frequency and percent of 7 factors) $	1
			Risk factors - Specific features of abuse (frequency and percent of 10 factors) $	1
			Risk factors - Victims' appraisals of threat (frequency and percent of 3 factors) $	1
			Risk factors - Factors relating to children (frequency and percent of 3 factors) $	1
			Risk factors - Factors relating to victim (frequency and percent of 4 factors) $	1
			Probability of none / standard / moderate / and high abuse (reported for physical abuse / sexual abuse / harassment and stalking / jealous and controlling behaviour) $	1
		**Police incidents$**		**39**
			Average / total number of police incidents$	1
			Average / total number of DV related police incidents$	2
			Average / total number of incidents recorded by the police per month$	1
			Average number of DV complaints per month$	1
			Average number of arrests made per month$	1

For peer review only

Category	Domain	Subdomain	Measure	N
			Average number of victims refusing to make a complaint per month$	1
			Average number of mental health related DV complaints per month$	1
			Average number of alcohol related DV complaints per month$	1
			Average number of drug related DV complaints per month$	1
			Average number of concern for children reports per month$	1
			Number / percent of participants that committed DV related police incidents$	1
			Number / percent of participants that committed (unspecified) police incidents$	1
			Number / percent of participants with (unspecified) arrests$	1
			Number / percent of calls to domestic incidents$	1
			Number / percent of repeat calls to domestic incidents$	1
			Number / percent of repeat calls to domestic violence incidents (number with 2 incidents, 3 incidents, 4 incidents… 14 incidents) $	1
			Number / percent of DV incidents where the perpetrator was not present$	1
			Number / percent of DV incidents that were crimed$	1
			Number / percent of DV incidents that led to arrests$	2
			Number / percent of incidents with an arrest where a statement is given$	1
			Number / percent of incidents with an arrest and a statement is given where it is then retracted$	1
			Number / percent of crimes where perpetrator was charged/summonsed$	1
			Number / percent of police incidents before and after a MARAC$	1
			Number / percent of high / medium / low severity police incidents before and after a MARAC$	1
			Number / percent of women experiencing no further post-MARAC incidents of violence or abuse$	1
			Number / percent of women experiencing no further post-MARAC police complaints or call outs$	2
			Number / percent of women experiencing no further post-MARAC police complaints$	2
			Number / percent of women experiencing no further post-MARAC police call outs$	2
			Number / percent of women experiencing either additional post-MARAC police complaints or call outs	1

For peer review only

Category	Domain	Subdomain	Measure	N
			Number / percent of women experiencing both additional post-MARAC police complaints and call outs	1
			Percent change in total calls to domestic incidents	1
			Percent change in total calls to domestic incidents (as proportion of all calls)	1
			Frequency of police call outs (never / once / 2-5 times / 6-10 times)	1
			Cost of police incidents	1

Note: Severity of Abuse Grid: there was a diverse approach to how it was used by different authors in the literature, , therefore we decided to split outcomes according to the various ways in which it was operationalised, rather than just reporting 'Severity of Abuse Grid' as an outcome.

* Note: Some studies do not explicitly specify using the Severity of Abuse Grid, but this has been presumed because the wording used when reporting outcomes matches exactly.

± Note: One study uses the outcome measure to assess perpetrators using abusive behaviours, rather than victim-survivor experiences of abuse.

$ Note: These outcomes and subdomains appear in more than one domain.

For peer review only

Figure 3: Temporal trends in outcome domains reported in DVSA evaluations, 1991 to 2022

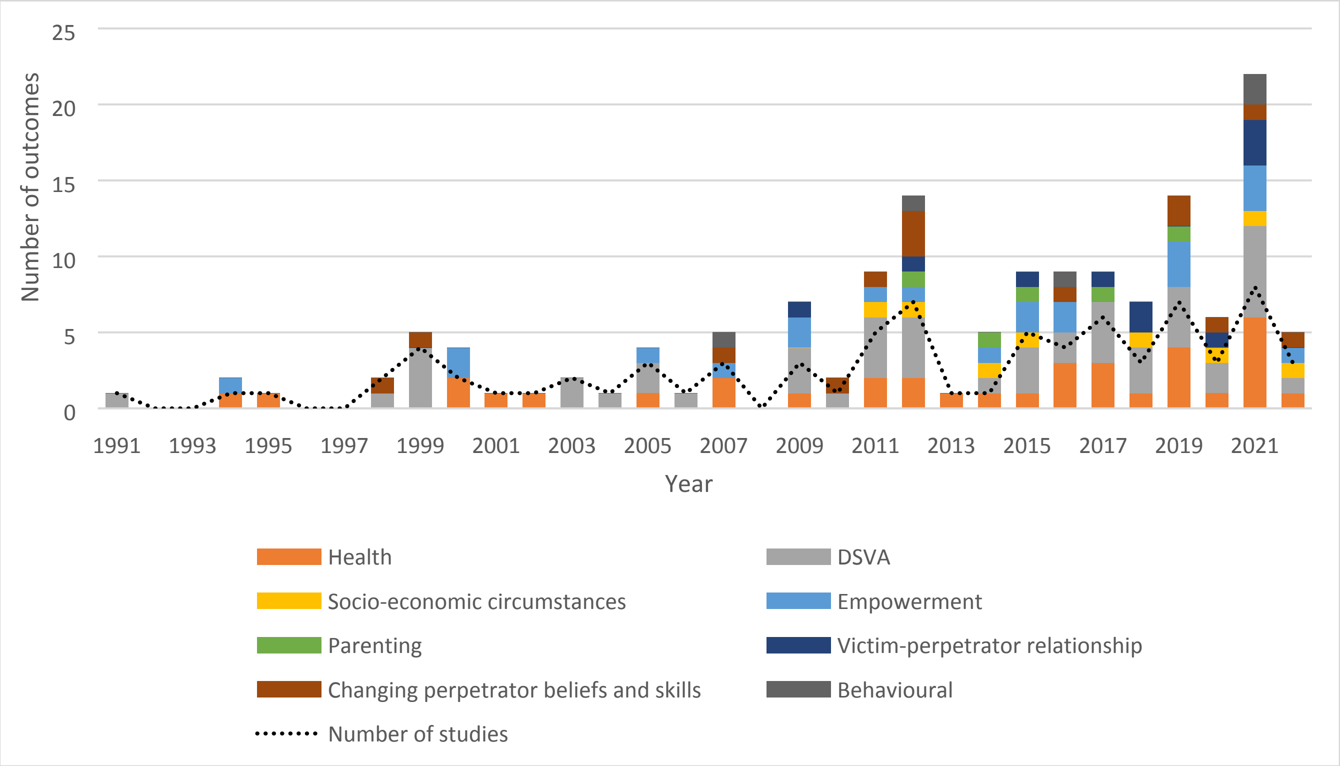

Note: For ease of interpretation, mental health, physical health and wellbeing domains have been combined into one health domain, and the experience of DSVA and perpetration of DSVA domains have been combined into a single 'DSVA' domain.
Note: Sixteen studies reported an outcome that crosses two or more domains therefore some outcomes are represented by multiple domains. Additionally, 38 studies reported an outcome that was categorised as both the experience of DSVA and perpetration of DSVA, because the specific outcome could represent either domain depending on who is completing the outcome measure.

BMJ Open

Trends in outcomes used to measure the effectiveness of UK-based support interventions and services targeted at adults with experience of domestic and sexual violence and abuse: A scoping review.

Journal:	*BMJ Open*
Manuscript ID	bmjopen-2023-074452.R1
Article Type:	Original research
Date Submitted by the Author:	13-Oct-2023
Complete List of Authors:	Carlisle, Sophie ; King's College London, Health Service and Population Research<br>Bunce, Annie; City University of London<br>Prina, Matthew; Newcastle University, Population Health Sciences Institute<br>Cook, Elizabeth; City University of London, Violence and Society Centre<br>Barbosa, Estela; University of Bristol, Population Health Sciences, Bristol Medical School; UKPRP VISION Consortium<br>McManus, Sally; City University of London; National Centre for Social Research<br>Feder, Gene; University of Bristol, Community based medicine<br>Lewis, Natalia; University of Bristol, Centre for Academic Primary Care, Population Health Sciences, Bristol Medical School; National Institute for Health Research Collaboration for Leadership in Applied Health Research and Care North Thames at Bart's Health NHS Trust, Queen Mary University of London, Centre for Primary Care and Public Health
**Primary Subject Heading**:	Public health
Secondary Subject Heading:	Public health
Keywords:	Systematic Review, PUBLIC HEALTH, MENTAL HEALTH

SCHOLARONE™
Manuscripts

I, the Submitting Author has the right to grant and does grant on behalf of all authors of the Work (as defined in the below author licence), an exclusive licence and/or a non-exclusive licence for contributions from authors who are: i) UK Crown employees; ii) where BMJ has agreed a CC-BY licence shall apply, and/or iii) in accordance with the terms applicable for US Federal Government officers or employees acting as part of their official duties; on a worldwide, perpetual, irrevocable, royalty-free basis to BMJ Publishing Group Ltd ("BMJ") its licensees and where the relevant Journal is co-owned by BMJ to the co-owners of the Journal, to publish the Work in this journal and any other BMJ products and to exploit all rights, as set out in our licence.

The Submitting Author accepts and understands that any supply made under these terms is made by BMJ to the Submitting Author unless you are acting as an employee on behalf of your employer or a postgraduate student of an affiliated institution which is paying any applicable article publishing charge ("APC") for Open Access articles. Where the Submitting Author wishes to make the Work available on an Open Access basis (and intends to pay the relevant APC), the terms of reuse of such Open Access shall be governed by a Creative Commons licence – details of these licences and which Creative Commons licence will apply to this Work are set out in our licence referred to above.

Other than as permitted in any relevant BMJ Author's Self Archiving Policies, I confirm this Work has not been accepted for publication elsewhere, is not being considered for publication elsewhere and does not duplicate material already published. I confirm all authors consent to publication of this Work and authorise the granting of this licence.

For peer review only

Trends in outcomes used to measure the effectiveness of UK-based support interventions and services targeted at adults with experience of domestic and sexual violence and abuse: A scoping review.

Sophie Carlisle[1], Annie Bunce[3], Matthew Prina[5], Elizabeth Cook[3], Estela Barbosa[2], Sally McManus[3,4], Gene Feder[2], Natalia V Lewis[2]

Corresponding author

Sophie Carlisle: Department of Health Service and Population Research, Institute of Psychiatry, Psychology and Neuroscience, King's College London, De Crespigny Park, London, UK. Telephone/fax: N/A. Email: Sophie.r.carlisle@kcl.ac.uk

Author affiliations

1 Department of Health Service and Population Research, Institute of Psychiatry, Psychology and Neuroscience, King's College London, De Crespigny Park, London, UK

2 Bristol Medical School, University of Bristol, Bristol BS8 2PS, UK

3 Violence and Society Centre, City, University of London, London, UK

4 National Centre for Social Research, London, UK

5 Population Health Sciences Institute, Faculty of Medical Sciences, Newcastle University, Newcastle, NE4 5PL, UK

Key words

Scoping review, domestic abuse, sexual violence, perpetrator programme, outcomes

For peer review only

For peer review only - http://bmjopen.bmj.com/site/about/guidelines.xhtml

Author Contributions

All authors (SC, AB, MP, EC, SM, GF, NL) contributed to the conceptualisation and design of the review. SC carried out searches, screening, extraction, data charting, and analysis. AB was the second reviewer and contributed to the screening and extraction stages. SC produced the first draft of the manuscript and subsequent revised versions following valuable input and refinement from the co-authors (AB, MP, EC, SM, GF, NL). All authors approved the final version.

Funding statement

This research was supported by the UK Prevention Research Partnership (Violence, Health and Society; MR-VO49879/1), which is funded by the British Heart Foundation, Chief Scientist Office of the Scottish Government Health and Social Care Directorates, Engineering and Physical Sciences Research Council, Economic and Social Research Council, Health and Social Care Research and Development Division (Welsh Government), Medical Research Council, National Institute for Health and Care Research, Natural Environment Research Council, Public Health Agency (Northern Ireland), The Health Foundation, and Wellcome. The views expressed in this Article are those of the authors and not necessarily those of the UK Prevention Research Partnership or any other funder. The funder had no input in the design of the protocol or conduct of the review.

Competing interests statement

None declared.

Data availability statement

All data relevant to the study are included in the article and uploaded as supplementary information.

For peer review only

Word count: 4673

Abstract

Objectives: In the UK, a range of support services and interventions are available to people who have experienced or perpetrated domestic and sexual violence and abuse (DSVA). However, it is currently not clear which outcomes and outcome measures are used to assess their effectiveness. The objective of this review is to summarise, map and identify trends in outcome measures in evaluations and reports of DSVA services and interventions in the UK.

Design: Scoping review

Data sources: MEDLINE, EMBASE, PsycINFO, Social Policy and Practice, ASSIA, IBSS, Sociological abstracts and SSCI electronic databases were searched from inception until 21st June 2022. Grey literature sources were identified and searched.

Eligibility: We included randomised controlled trials, non-randomised comparative studies, pre-post studies and service evaluations, with at least one outcome relating to the effectiveness of the support intervention or service for people who have experienced and/or perpetrated DSVA. Outcomes had to be assessed at baseline and at least one more time-point, or compared to a comparison group.

Charting methods: Outcome measures were extracted, iteratively thematically grouped into categories, domains and subdomains, and trends were explored.

Results: 80 studies reporting 87 interventions or services were included. A total of 426 outcome measures were extracted, of which 200 were used more than once. The most commonly reported subdomain was DSVA perpetration. Cessation of abuse according to the Severity of Abuse Grid was the most common individual outcome. Analysis of temporal trends showed that the number of studies and outcomes used has increased since the 1990s.

For peer review only - http://bmjopen.bmj.com/site/about/guidelines.xhtml

Conclusions: Our findings highlight inconsistencies between studies in outcome measurement. The increase in the number of studies and variety of measures suggests that as evaluation of these services and interventions matures, there is an increased need for a core of common, reliable metrics to aid comparability.

Protocol registration: https://osf.io/frh2e

Article summary

Strengths and limitations of this study

- This scoping review adhered to PRISMA 2020 guidelines to robustly and systematically map effectiveness outcome measures used in evaluations of support interventions and services for people who have experienced and/or perpetrated DSVA.
- It included an extensive grey literature search to be comprehensive and inclusive.
- An advisory group consisting of representatives from six third sector organisations has been involved in the development of the review.
- The review has not included services or interventions for children who have experienced DSVA, interventions aimed at staff training, and is limited to the UK.
- Further research is urgently required to work towards agreement on which outcome measures should be prioritised and adopted, whilst also avoiding burden on systems and people experiencing DSVA.

For peer review only

Introduction

Domestic and sexual violence and abuse (DSVA; comprising a range of different behaviours and experiences, see Appendix 1) represents a significant international public health issue [1-3]. Approximately 1 in 3 women and 1 in 7 men in England and Wales have experienced domestic violence and abuse in their lifetime [4]. Globally, over 35% of women have experienced either physical and/or sexual intimate partner violence or non-partner sexual violence [5]. Poverty, homelessness, and insecure immigration status heighten DSVA vulnerability [6, 7], and the risk of experiencing DSVA is also higher for younger adults, lesbian, gay, bisexual and transgender (LGBT) people [8-11], and people with long term illness, disability or mental health problems [12, 13]. The UK does not currently routinely collect data on perpetrators, however studies have found that the prevalence of DSVA perpetration ranges from 11.6-80.0% depending on country, gender and definition of DSVA [14, 15]. Perpetrators are typically male [16] and are often serial perpetrators with more than one victim [17, 18]. The impacts of DSVA are profound and diverse. As well as visible and immediate consequences such as physical injury or death, DSVA has been associated with longer-term physical health [19-25] and mental health problems [26-29]. Other areas impacted by DSVA include homelessness [30], access to finances, employment and education [31], and increased demand on the criminal justice system [32], social services [33], and police [34].

Given the wide-ranging impacts of DSVA, the support options available to people who have experienced it are diverse. Interventions and services include refuges, outreach, counselling, financial or legal advice, helplines and advocates. In the UK, these are often provided by third sector organisations (organisations that are neither public nor private sector, including voluntary and community organisations; see Appendix 1), but can also be provided by services located in the public and private sectors. Some services are targeted at particular groups with heightened vulnerability to DSVA, such as LGBT+ and Black and Minority Ethnic

For peer review only

(BME) victim-survivors or victim-survivors with disability, however provision for these groups is stark and disproportionately underfunded [35-37]. Previous systematic reviews of the global evidence have found benefits of such interventions, including housing interventions improving mental health, perceived safety and stress [38], economic interventions reducing levels of domestic violence and increasing empowerment [39], advocacy interventions improving quality of life and depression [40], and psychological therapies reducing depression and anxiety [41]. Service evaluations and annual reports produced by local DSVA organisations have also found associations between service use and improved perceptions of safety, quality of life, health and wellbeing, and confidence [42-46]. Finally, reviews of perpetrator programmes (i.e., interventions targeted at people who use violence) have provided some evidence of reductions in the perpetration and experience of abusive behaviours, although highlighted methodological issues [47, 48].

Despite existing reviews identifying potential benefits of specific types of support services and interventions, there is limited understanding of which outcomes and outcome measures are currently being used both within and across UK-based support services and interventions when assessing their effectiveness [49, 50]. The specific outcome measures that are being used appear to vary, making it difficult to compare and synthesise the overall evidence [51]. Identifying the most commonly utilised outcome measures will allow for meta-analysis, leading to more rigorous evaluations of the literature, and may also guide interventions and services on which outcome measures to use. Scoping reviews present an opportunity to map the evidence, and are ideal for summarising heterogenous evidence [52, 53]. This scoping review aims to summarise and map trends in the outcome measures being used to evaluate UK-based support interventions and services for DSVA.

Objectives

Specific objectives of this scoping review include:

For peer review only

1. Identify and thematically group outcome measures that have been used to assess the effectiveness of UK-based support interventions and services for adults who have experienced and/or perpetrated DSVA.
2. Identify which outcome measures have been used most frequently.
3. Explore whether the outcome measures differ by: the type of victim-survivor or perpetrator support; DSVA type; source of the study; study setting; and over time.

Review question

What outcomes relating to effectiveness have been measured in intervention studies and service evaluations of UK-based support programmes for adults who have experienced and/or perpetrated DSVA?

Methods

Protocol and registration

The protocol was developed according to the Preferred Reporting Items for Systematic Reviews and Meta-Analysis Extension for Scoping Reviews (PRISMA-ScR) [54] and published on Open Science Framework: https://osf.io/frh2e [55]. The PRISMA-ScR checklist is available in Appendix 3.

Eligibility criteria

We included any UK-based secondary or tertiary prevention support services and interventions aimed at adults who had experienced or perpetrated DSVA. Primary prevention services and interventions were excluded as they aim to prevent DSVA before it occurs, and therefore focus on individuals or populations who have not necessarily experienced violence. Interventions and services that were not specifically aimed at DSVA, such as general trauma interventions, were only included if the majority of participants (>50%) had experienced DSVA. Randomised controlled trials (RCTs), non-randomised comparative trials, pre-post

For peer review only - http://bmjopen.bmj.com/site/about/guidelines.xhtml

studies and service evaluations that reported effectiveness outcomes at two or more time-points or made comparisons to another group were included, so that cause and effect could be inferred. Perpetrator programmes were considered for several reasons. Firstly, including them allows the measurement of DSVA without placing the burden on people who have experienced DSVA to change someone else's behaviour. This ties in to the UK government's new perpetrator strategy [56], which intends to place the onus on perpetrators changing their behaviour, alongside the recovery of those who have experienced DSVA. Finally, perpetrator programmes provide support for perpetrators to change their behaviour, and many offer associated support to (ex)partners or referral to appropriate support. Details of the full eligibility criteria can be found in Table 1.

Table 1: PICO Inclusion and Exclusion Criteria

	Inclusion	Exclusion
Population	• Adults (16 years or older) who have experienced DSVA.<br>• Adults (16 years or older) who have perpetrated DSVA.	• Children<br>• Adults who have not experienced and/or perpetrated DSVA
Intervention (and service)	• Any secondary or tertiary prevention intervention and/or service for DSVA e.g., housing (e.g., refuges, housing workers, resettlements), Advocacy (e.g., Independent Domestic Violence Advisers/Advocates (IDVAs), Independent Sexual Violence Advisers/Advocates (ISVAs)), outreach, open access (e.g., helplines, drop-ins, online chats), psychological support (e.g., support groups, counselling, befriending), legal support, financial support, multi-agency risk assessment conferences (MARACs), and police-based services.<br>• Perpetrator programmes (see Appendix 1).<br>• Entry to the intervention had to be determined by the either the experience of DSVA (for victim-survivor support interventions) or perpetrating DSVA (for perpetrator programmes).	• Pharmacological<br>• Primary prevention<br>• Not primarily aimed at people with experience of DSVA

For peer review only - http://bmjopen.bmj.com/site/about/guidelines.xhtml

	Inclusion	Exclusion
Comparison	• RCTs:<br>○ Another type of included intervention or service.<br>○ Usual care<br>○ No intervention or service<br>• Pre-post designs:<br>○ Before and after the intervention or service	• No comparison
Outcome	• Any outcomes used to measure effectiveness of DSVA support services and interventions.	• Those not focussed on the effectiveness of the intervention or service (e.g., process evaluation outcomes such as satisfaction, staff training, etc)
Setting	• UK based	• Not UK based
Study design	• RCT<br>• Nonrandomised comparative<br>• Pre-post<br>• Service evaluation	• Cross-sectional, case control, case study<br>• Qualitative<br>• Letters to the editor, think pieces, descriptive only
Dates	• 1982-present	• Pre-1982

Information sources and search strategy

MEDLINE, EMBASE, PsycINFO, Social Policy and Practice, ASSIA, IBSS, Sociological abstracts and SSCI electronic databases were searched from inception until 21st June 2022. Grey literature sources were also identified, including searching electronic grey literature databases (National Grey Literature Collection, EThOS, Social Care Online and the Violence Against Women Network), a call for evidence which was shared with research networks and UK-based DSVA services and organisations, and searches of relevant UK charity and organisation websites. Finally, backwards and forwards citation tracking of all included studies, as well as reference searching of identified systematic reviews was undertaken to identify further relevant studies. The search results were exported into EndNote and duplicates were removed. An example of the search strategy for the peer review search and the grey literature search can be found in Appendix 2.

For peer review only - http://bmjopen.bmj.com/site/about/guidelines.xhtml

Selection of sources of evidence

The de-duplicated citations were uploaded into Rayyan [57]. Titles and abstracts, followed by full-texts, were screened according to the inclusion and exclusion criteria. A 20% sample was independently screened by a second reviewer at each stage, and all disagreements were resolved by discussion, with a third reviewer where needed. Risk of bias assessments are not necessary for scoping reviews [52].

Data charting process and data items

Data charting took place using a piloted excel spreadsheet. Data items extracted included the study citation, study design, participant demographics, description of the service or intervention and comparison (if applicable), setting, and the outcome measures used. All outcomes that were used to assess the effectiveness of DSVA support interventions and services were extracted. Data extraction and charting was carried out by one reviewer, and checked for errors by a second reviewer. Where data was missing, corresponding authors were contacted and asked to supply said data. After two weeks, if there had been no response, corresponding authors were contacted a second time and given a minimum of an additional two weeks to respond.

Synthesis of results

A narrative synthesis was conducted to summarise the included interventions and services and the effectiveness outcomes they measured. The unit of analysis was the outcome measure(s) reported. Interventions and services were grouped by the type of support offered, based on the intervention and service types outlined in the protocol. Where studies reported more than one form of support as part of the intervention or service, they were classified as "multi-service". Where the same intervention or service produced multiple reports (e.g., from different years), these were combined into one entry during data charting

For peer review only - http://bmjopen.bmj.com/site/about/guidelines.xhtml

so that all outcomes ever reported by that service were extracted, but were not double counted if multiple reports used the same outcome.

Effectiveness outcomes reported by studies were grouped thematically by type to create categories, domains and subdomains. The groupings and the names for the sub-domains, domains and categories were developed iteratively. All outcome measures were firstly listed in an excel spreadsheet. This included standardised and unstandardised questionnaires and single item measures. A column was added, describing what the outcome measured (e.g., the Beck Depression Inventory [58] measures depression). These sub-domains were then grouped into broader domains (e.g., depression is a mental health-related outcome). Where applicable, these were grouped into again even broader categories (e.g., mental health is a form of overall health).

A list of all relevant outcomes and domains, and how frequently they were reported was made. Comparisons were made between outcomes reported by studies in different sectors, over time, and by service user (i.e., perpetrator or victim-survivor) and support type (i.e., psychological support, housing, combinations), or violence type (i.e., domestic violence and abuse [DVA], sexual violence and abuse [SVA], childhood sexual abuse [CSA]).

Patient and public involvement

An advisory group was set up comprising representatives from six specialist DSVA organisations who are involved in the delivery, planning, funding or support of specialist DSVA support services in the UK. Specifically, the group included representatives from two second-tier (i.e., organisations that support front-line services but do not provide services themselves) domestic abuse organisations, one second-tier organisation for violence against BME women and girls, one domestic abuse organisation that provides a range of front-line services, one service focusing specifically on supporting male victims, working with

For peer review only - http://bmjopen.bmj.com/site/about/guidelines.xhtml

perpetrators of domestic violence, and working with young people using violence in close relationships, and one service focusing specifically on sexual violence and abuse. The group inputted to the design of the study protocol, interpretation of the data, and provided insight regarding the challenges in measuring the effectiveness of support services in the third sector. Their input resulted in several changes, including the broadening of the scope of this review to all effectiveness outcomes, rather than a narrower focus on only outcomes relating to reducing violence, to reflect the priorities of the sector.

Results

Selection of sources of evidence

After duplications were removed, a total of 12431 records and 869 reports were screened for inclusion. Eighty studies from 111 reports were included in this review (see Figure 1). Data extracted from the included studies are summarised in Appendix 4 [46, 59-168].

Characteristics of sources of evidence

The 80 studies described a total of 87 interventions or services, with seven studies describing both a perpetrator programme and an associated support service for (ex)partners of participating perpetrators. All studies were conducted between 1991 and 2022.

Description of studies

Study design

Many of the included studies were mixed-method service evaluations; for these studies, only the methodology relevant to the included outcomes is reported as the other methods do not meet the review eligibility criteria (e.g., qualitative, case studies). The most common study design was pre-post single group designs (n=53), of these 14 included additional time-points at mid-intervention or follow-up. Eight studies were RCTs, and ten used a non-randomised comparative design. For one study, the design could not be determined. Eight studies used

For peer review only - http://bmjopen.bmj.com/site/about/guidelines.xhtml

secondary analysis of datasets, which collated data from multiple services. These were primarily publications produced by SafeLives, a UK-based charity dedicated to ending domestic abuse, using their Insights system, an outcome measurement tool which allows services across England and Wales to collect data according to various measurement indicators.

Perpetrator programmes

Perpetrator programmes were grouped together. They were typically described as behaviour change programmes based on a cognitive behavioural model, and whilst many included additional elements such as psychological support, these were often described in insufficient detail. Of the 80 included studies, 27 described a perpetrator programme.

Victim-survivor support interventions and services

Interventions and services providing support for those who had experienced DSVA were grouped by type of support provided, based on initial scoping of the literature. The most common were psychological support (n=21), advocacy (n=14) and multi-service (n=11). Three studies each reported on multi-agency risk assessment conferences (MARACs) and specialist domestic violence police teams. Two studies reported housing interventions or services. One study each reported on sexual violence services, a specialist domestic violence court, helpline services, outreach services, and adult health-based services. One study described a women's service linked to an associated perpetrator programme, but the specific type of support offered was unclear. Several studies evaluated the effectiveness of more than one intervention or service. Of the 60 victim-survivor support interventions and services, two were specifically targeted at male victim-survivors, one at BME women, one at victims of trafficking, and one at women with disabilities. There was no evidence for interventions or services targeted specifically at LGBT+ victim-survivors.

For peer review only

Type of DSVA

The studies were categorised as being primarily aimed at DVA, SVA, both DVA and SVA (DSVA), or CSA. Categorisations were based on how the study or service described itself, however it should be noted that the type of violence/abuse was often not clearly defined. Thus, these categorisations should be considered as having ambiguous boundaries, and interpreted with caution.

The majority (n=53; 66.3%) of the studies were aimed at DVA, whilst 24 (29.3%) were aimed specifically at SVA. Of these, nine studies were specifically targeted at adults who had experienced CSA. Three studies (3.8%) were aimed at both DVA and SVA, or DSVA.

Source of the studies

Of the 80 studies and 111 reports, 41 studies from 47 reports were located in the peer-reviewed literature, and 39 studies from 64 reports were located in the grey literature.

Setting

Just under half of studies were conducted in the third sector (n=39; 47.6%). Twenty-six studies were conducted in the public sector, and 12 spanned multiple sectors. One was conducted in a privately owned prison, and for one study the setting was unclear.

Types of outcomes

The 80 included studies measured a total of 426 outcomes. Of these 200 (47.0%) were used more than once. In total, there were 282 unique outcomes, 57 of which were used more than once, and 226 were used only once. The number of effectiveness outcomes measured by each individual study ranged from one to 19, with a mean average of 5.23.

For peer review only

For peer review only - http://bmjopen.bmj.com/site/about/guidelines.xhtml

Outcomes were grouped thematically into 11 domains and 49 subdomains. The 11 domains included mental health, physical health, general wellbeing (all of which fall under the broad category of 'health'), the experience of DSVA and the perpetration of DSVA (which fall under the broad category of 'DSVA'), empowerment, socio-economic circumstances, behavioural outcomes, the victim-perpetrator relationship, parenting, and changing perpetrator beliefs and skills (Appendix 5).

Many of the outcome measures reported spanned two or more domains (Figure 2). For instance, 'health' as an overarching category included three domains: mental health, physical health and general wellbeing. 'Quality of life' as a subdomain spanned physical health, mental health and general wellbeing domains, based on the identified outcome measures and their specific items. For example, the 12-item Short Form (SF-12) is a measure of quality of life and general health, which includes items relating to physical health, mental health, and general wellbeing. Similarly, the overarching category of DSVA contained two domains: the experience of DSVA and the perpetration of DSVA. However, many outcome measures could fall into either domain, depending on who the respondent was (i.e., the perpetrator or the victim-survivor). For example, the outcome 'presence of abuse' would fall into the 'experience of DSVA' domain if the respondent was the victim-survivor, and the 'perpetration of DSVA' domain if the respondent was the perpetrator. Thus, there was substantial overlap between these two subdomains. Many of the outcomes within the DSVA category, whilst defined slightly differently, were not distinct. For instance, there is significant overlap between the concept of 'revictimization' and the outcome 'presence of abuse' when measured using a pre-post design (if the victim-survivor indicates that they are still experiencing abuse post-intervention, then this indicates 'revictimization'). There is similarly overlap between reoffending and revictimization (although we cannot presume a direct overlap, as a perpetrator may have multiple victims) and between 'reoffending' and 'police incidents' (although not all reoffending will be recorded by police therefore this subdomain may be broader).

For peer review only - http://bmjopen.bmj.com/site/about/guidelines.xhtml

For each of the 11 domains, the total number of outcomes, the total number of unique outcomes, and the number of studies reporting at least one outcome that fell within that domain, were explored. The domain 'perpetration of abuse' has the highest number of studies reporting at least one outcome, the most total outcome measures, and the most unique outcome measures (Table 2).

For peer review only

For peer review only - http://bmjopen.bmj.com/site/about/guidelines.xhtml

Table 2. Frequency of outcomes by outcome domain, and the number of studies reporting one or more outcomes per outcome domains by intervention type, source of study, violence type and sector

		Outcome measure domain										
		Mental health	Physical health	Wellbeing	Behavioural	Empowerment	Socio-economic circumstances	Changing perpetrator beliefs and skills	Victim-perpetrator relationship	Parenting	Experience of DSVA	Perpetration of DSVA
Number of studies reporting at least one outcome		29	11	19	5	21	9	14	10	5	42	46
Number of total outcome measures		85	22	25	11	32	17	33	12	7	189	201
Number of total unique outcome measures		52	16	21	11	23	17	28	7	5	106	119
**Type of intervention**	**Number of studies**											
Perpetrator programme	27	2	1	2	1	1	1	14	4	3	14	21
Victim support:												
Psychological support	21	17	1	7	2	10	2	0	0	1	1	0
Advocacy	14	5	4	4	0	4	2	0	2	1	12	11
Multi-service	11	4	4	4	1	5	1	0	0	0	8	5
Housing	2	0	0	0	0	0	1	0	1	0	1	1
MARAC	3	0	0	0	0	0	0	0	0	0	3	2
Specialist DV police teams	3	0	0	1	0	0	0	0	0	0	2	2
Specialist DV court	1	0	0	0	0	0	0	0	0	0	0	1
Sexual violence service	1	1	1	1	1	1	1	0	0	0	1	1
Helpline	1	0	0	0	0	0	0	0	1	0	1	1
Outreach	1	0	0	0	0	0	0	0	1	0	1	1

For peer review only

For peer review only - http://bmjopen.bmj.com/site/about/guidelines.xhtml

Health	1	0	0	0	0	0	1	0	1	0	1	1
Other / Unclear	1	0	0	0	0	0	0	0	0	0	1	1
**Source**												
Peer reviewed	41	22	6	10	3	11	3	9	1	3	13	19
Grey literature	39	7	5	9	2	10	6	5	9	2	29	27
**Type of violence**												
DVA	53	11	9	12	2	10	6	8	10	5	40	41
SVA	15	7	2	4	2	5	1	6	0	0	1	4
Both	3	2	0	0	0	1	0	0	0	0	1	1
CSA	9	9	0	3	1	5	2	0	0	0	0	0
**Sector**												
Third	39	12	7	14	3	12	7	5	9	4	27	23
Public	26	13	2	3	1	8	2	5	0	1	5	10
Mixed	12	3	2	1	1	0	0	2	1	0	9	11
Private	1	1	0	1	0	1	0	1	0	0	0	0
Unclear	2	0	0	0	0	0	0	1	0	0	1	2

Note: CSA: Childhood sexual abuse; DSVA: Domestic and sexual violence and abuse; DV: Domestic violence; DVA: domestic violence and abuse: MARAC: Multi-agency risk assessment conference; SVA: Sexual violence and abuse

Note: Sixteen studies reported an outcome that fits into multiple domains therefore some outcomes are represented by multiple domains. Additionally, 38 studies reported an outcome that was categorised as both the experience of DSVA and perpetration of DSVA, because the specific outcome could represent either domain depending on who is completing the outcome measure.

Note: Two studies have outcome domains that are represented in multiple intervention types (perpetrator programme, unclear/other and advocacy), because they report a perpetrator programme and an associated women's service, but it is unclear which intervention/service the outcomes were measured as part of.

Note: The number of studies for the different intervention/service types is more than the total number of studies (80) because 7 studies reported a perpetrator programme and associated women's service.

For peer review only

Of the three most common outcome measures across intervention types, all fell within the DSVA category (experience of DSVA / perpetration of DSVA; Table 3). Cessation of abuse according to the Severity of Abuse Grid (SAG) [169] was the most common outcome (n=11), followed by the presence of abuse (n=10) and the severity of abuse (n=8), both also measured by the SAG. The Rosenberg Self-Esteem Scale [170], and two more measures of DSVA from the SAG (multiple types of abuse; abuse that is escalating in severity or frequency) were each reported by seven studies.

For peer review only

Table 3: Most commonly reported outcome measures

Outcome	Subdomain	Domain	No. of studies
**Overall**			
1. Cessation of abuse measured by the SAG [169]	Presence of abuse	Experience of DSVA/ perpetration of DSVA	11
2. Presence of abuse measured by the SAG* [169]	Presence of abuse	Experience of DSVA/ perpetration of DSVA	10
3. Severity of abuse measured by the SAG* [169]	Severity of abuse	Experience of DSVA/ perpetration of DSVA	8
4. RSES [170]	Self-esteem	Empowerment	7
5. Multiple types of abuse measured by the SAG [169]	Presence of abuse	Experience of DSVA/ perpetration of DSVA	7
6. Abuse that is escalating in severity or frequency as measured by the SAG [169]	Severity and frequency of abuse	Experience of DSVA/ perpetration of DSVA	7
**Peer review vs grey literature**			
**Grey literature**			
1. Cessation of abuse measured by the SAG [169]	Presence of abuse	Experience of DSVA/ perpetration of DSVA	9
2. Presence of abuse measured by the SAG* [169]	Presence of abuse	Experience of DSVA/ perpetration of DSVA	9
3. Severity of abuse measured by the SAG* [169]	Severity of abuse	Experience of DSVA/ perpetration of DSVA	8
**Peer review**			
1. RSES [170]	Self-esteem	Empowerment	6
2. BDI [58]	Depression	Mental health	5
3. CORE-OM [171]	Psychological issues	Mental health	5
**Sector**			
**Third**			
1. Cessation of abuse measured by the SAG [169]	Presence of abuse	Experience of DSVA/ perpetration of DSVA	9
2. Presence of abuse measured by the SAG* [169]	Presence of abuse	Experience of DSVA/ perpetration of DSVA	9
3. Severity of abuse measured by the SAG* [169]	Severity of abuse	Experience of DSVA/ perpetration of DSVA	8
**Public**			

Outcome	Subdomain	Domain	No. of studies
1. BDI [58]	Depression	Mental health	5
2. RSES [170]	Self-esteem	Empowerment	4
3. SCL-90R [172]	Psychological issues	Mental health	4
**Mixed**			
1. Cases discontinued	Criminal justice outcomes	Perpetration of DSVA	2
2. PHQ [173]	Depression	Mental health	2
3. GAD-7 [174]	Anxiety	Mental health	2
4. Cambridge Crime Harm Index [175]	Severity of abuse	Experience of DSVA/ perpetration of DSVA	2
5. PC-PTSD [176]	Trauma	Mental health	2
6. No further post-MARAC police complaints or call outs	Police incidents	Experience of DSVA/ perpetration of DSVA	2
7. No further post-MARAC police complaints	Police incidents	Experience of DSVA/ perpetration of DSVA	2
8. No further post-MARAC police call outs	Police incidents	Experience of DSVA/ perpetration of DSVA	2

Note: BDI: Beck Depression Inventory; CORE-OM: Clinical Outcomes in Routine Evaluation Outcome Measure; DSVA: Domestic and sexual violence and abuse; GAD-7: Generalised Anxiety Disorder Questionnaire; MARAC: Multi-agency risk assessment conference; PHQ: Patient Health Questionnaire; PC-PTSD-5: Primary Care PTSD Screen; RSES: Rosenberg Self-Esteem Scale; SAG: Severity of Abuse Grid; SCL-90R: Symptom Checklist 90 revised.
**Reported separately for physical abuse, sexual abuse, harassment and stalking, and jealous and controlling behaviour*

Patterns in the use of outcome

Table 2 summarises the patterns in outcome domains according to perpetrator programmes, victim support type, DSVA type, source of literature and setting/sector.

Outcomes in perpetrator programmes

Of the 27 perpetrator programmes, 14 reported at least one outcome relating to changing perpetrator behaviour, and 21 reported at least one relating to the perpetration of DSVA.

Outcomes by victim support type

The most common outcome domains for psychological support interventions and services were mental health (n=17) and empowerment (n=10), whilst advocacy, multi-service interventions and studies of MARACs primarily reported on experience of DSVA (n=12, n=8 and n=3, respectively). Specialist DV police teams reported equally on the experience of and the perpetration of DSVA (n=2 each).

For peer review only - http://bmjopen.bmj.com/site/about/guidelines.xhtml

For peer review only

Outcomes by type of DSVA

Of the studies primarily focused on DVA, the majority reported at least one outcome relating to the experience of DSVA (n=40; 75.5%) and the perpetration of DSVA (n=41; 77.4%). Just under half of studies of adult SVA (n=7; 46.7%), and all studies of CSA, reported mental health outcomes (n=9; 100%).

Outcomes by source of studies

Whilst the split of included studies from the grey literature and peer reviewed literature was relatively even, studies located within the grey literature were more diverse. In total, grey literature studies reported 254 outcome measures, 178 of which were unique. In comparison, peer reviewed studies reported a total of 172 outcomes, 125 of which were unique. Peer reviewed studies had more of a tendency to report at least one outcome relating to mental health (n=22; 53.7%), whilst grey literature studies were more likely to report outcomes relating to the experience of DSVA (n=29; 74.4%).

Outcomes by setting

Two thirds of studies set in the third sector reported outcomes relating to the experience of DSVA subdomain (n=27; 69.2%). Perpetration of DSVA was the most common outcome subdomain for interventions and services with a mixed setting (n=11; 91.7%), whilst mental health was the most common subdomain for those in the public sector (n=13; 50%).

Outcomes 1991 to 2022

Evaluations of DSVA support services and interventions have become more frequent over time. Nearly half (48.8%) of included studies were published in 2015-2022, with over a quarter (26.3%) published in 2019-2022 (Appendix 6).

For peer review only - http://bmjopen.bmj.com/site/about/guidelines.xhtml

Discussion

This study used scoping review methodology to map which outcomes have been used in studies assessing the effectiveness of a range of support interventions and services for people who have experienced or perpetrated DSVA. A total of 426 outcome measures were used in 80 papers and reports, describing 87 interventions and services. Less than half of the measures were used more than once.

The most common outcome domain was the perpetration of DSVA, followed closely by the experience of DSVA, both of which included outcomes relating directly to abusive and violent behaviours. This demonstrates that the measurement of DSVA has been prioritised when assessing service and intervention effectiveness, and is consistent with the common goal of many DSVA support services and organisations of ending violence and abuse [177, 178]. However, 201 and 189 unique outcome measures were charted within these two domains respectively, showing the lack of consensus regarding the specific outcomes that should be measured. The SAG, which measures several different aspects of abusive behaviours (including presence of four types of abuse, frequency, severity, and cessation of abuse), was the most frequently used outcome measure. It was primarily used by studies drawing upon the SafeLives' Insights datasets, although it was employed by several additional studies too. Despite being the most frequently reported outcome, the SAG was still utilised by only 13 of the 80 studies, illustrating the inconsistencies and variation in measures being used by these evaluations and reports.

Of the 282 unique outcomes reported, 226 were used only once. Although many of these outcomes were similar, they often differed slightly according to the unit or method of measurement, meaning that combining them would be methodologically inappropriate. This further demonstrates the disparate and highly variable nature of outcome measures currently

For peer review only - http://bmjopen.bmj.com/site/about/guidelines.xhtml

in use. Whilst this may be partly explained by the fact that different sectors and types of support interventions have different priorities, such disparity is evident even *within* sectors and intervention types. For instance, of the 153 different outcome measures reported by third sector studies, only 24.8% were used more than once, with a similarly low proportion in mixed sector and public sector studies (both 30.7%). This indicates a lack of comparability surrounding the best way to assess the effectiveness of support services and interventions, and makes synthesis such as meta-analysis less feasible.

Mapping revealed differences between the types of outcome domains frequently reported in the peer reviewed and grey literature, with peer reviewed studies focusing more on mental health related outcomes, and grey literature studies focusing primarily on DSVA. This difference may be explained by the overlap between grey literature and third sector-based studies, and the fact that third sector organisations are heavily tied to the outcomes that funders and commissioners require them to measure. With the focus on wanting to establish and reduce the prevalence of DSVA itself, and the associated focus on the perpetration of violence, it might be that more funders and commissioners are requiring these to be measured, or are prioritising these measurements above the wider impacts of DSVA.

Mapping of trends over time showed that as well as a general increase in the number of studies being conducted, there appeared to be increased diversity in outcomes measured. The two most common outcome categories, DSVA and health, have been measured fairly consistently over the past thirty years, whilst domains such as empowerment, the victim-perpetrator relationship and parenting have become more common in the past decade. This is likely reflective of an increased knowledge of the wide-ranging impacts of DSVA and its ripple effects, increased recognition of the need for holistic support, and the move towards survivor-centred outcome measurement and consultation with those with lived experience [179].

For peer review only - http://bmjopen.bmj.com/site/about/guidelines.xhtml

Challenges in outcome measurement

There are two linked challenges facing UK-based DSVA services, especially those located in the third sector. Firstly, funding of DSVA services is precarious and diminishing [180-183]. Being awarded funding requires evidence of effectiveness. However, the second challenge is that carrying out service evaluations to provide evidence of effectiveness is costly, making it even more difficult for services to secure funding. This is reflected in take-up of the aforementioned SafeLives' Insights system, which captures rich data and has likely contributed to more consistent reporting of outcomes across England and Wales. However, individual services have to pay to use the system, and the numbers of services contributing to the dataset each year has reduced, likely due to reduced funding [consultation with stakeholder advisory group].

Limitations

One limitation of this review is the unavoidable potential for double counting. For instance, the services that are included in SafeLives' Insights datasets are anonymous. Thus, it is not possible to determine if any services included in the Insights datasets have also published an independent evaluation. A second limitation of this review is that despite a comprehensive search strategy, no evaluations of support services run by by-and-for organisations were identified (see Appendix 1). This is illustrative of a systemic, cyclical and enduring issue facing these smaller services in particular, who are disproportionally underfunded and lack the resources needed to conduct and publish evaluations, meaning they are underrepresented in the literature [36, 184-186]. Additionally, because of time and resource restrictions, only 20% of records were dual screened during the title/abstract and full text screening stages, which does increase the risk of relevant studies being incorrectly excluded. Further, we excluded process evaluation outcomes. Given that there may be causal links between process measures and distal outcomes, it is possible that some relevant outcomes were missed. Finally, given the primary objectives of the review were to

For peer review only - http://bmjopen.bmj.com/site/about/guidelines.xhtml

map and chart outcome measures being used, we have not made any assessments regarding the validity or relevance of any particular measure. Therefore, whilst we have identified the most commonly used outcome measures, that is not to say that these are the most helpful, informative or important to people with lived experience or front-line service providers.

Implications and recommendations

This review has demonstrated that the types of outcomes measured when assessing the effectiveness of support interventions and services for people who have experienced DSVA vary widely. This has implications for systematic reviews and evidence syntheses looking to assess the state of the literature, as well as for the allocation of funding and the development of policies related to outcome measurement. Increased consensus between researchers, service providers, and funders is needed. This will allow for more meaningful syntheses of the literature, as well as building a larger evidence base, so that a better understanding of the most effective means of support for people who have experienced DSVA can be reached. Further research is needed to determine which outcomes and outcome measures are most appropriate, valid and relevant, in order to work towards a consensus and build a shared evidence base to enable future meta-analyses. This work should be carried out as a collaboration between people who have experienced abuse and their families, service providers and, in the UK, commissioners, to ensure researchers and evaluators are measuring the outcomes that matter most to the people who matter most. Such approaches have previously been used to develop outcome frameworks in related areas [51]. This work should also consider whether the outcomes and outcome measures most appropriate and relevant vary by different characteristics or populations, such as male victim-survivors, BME populations, or those from the LGBT+ community. Any outcome frameworks that are developed should consider this and be adaptable to the different target populations of various services.

For peer review only - http://bmjopen.bmj.com/site/about/guidelines.xhtml

Conclusions

A broad range of outcome measures have been used to assess support interventions for people who have experienced or perpetrated DSVA. There is growing recognition across various sectors of the potential benefits of a core set of outcomes, which include increasing consistency and consensus [187, 188]. We need to take stock of the current state of the evidence and reconceptualise what a core set of outcomes should look like across and within sectors. This core outcome set needs to be co-produced by a range of stakeholders, including those with experience of DSVA and those providing front line support. For this to be feasible a trade-off needs to occur. Burden needs to be minimised by not requiring excessive data collection, but instead, collection of only the most relevant outcome measures according to the specific purpose of the intervention or service. At the same time, within-service consistency needs to be increased, as well as supporting comparability across sectors. Further research is needed to better understand which outcomes should be prioritised in order to strike this balance.

For peer review only

Figure legends:

Figure 1. PRISMA flow diagram

Figure 2. Venn diagram of outcome categories, domains and subdomains

Ethical approval statement: Not applicable as this was a scoping review.

For peer review only

For peer review only - http://bmjopen.bmj.com/site/about/guidelines.xhtml

References

1. UK Faculty of Public Health, *The role of public health in the prevention of violence* 2016.
2. Savage-Borne, J. and A. Maitra. *Intimate partner violence as a public health problem*. 2021; Available from: https://blogs.bmj.com/bmj/2021/03/25/intimate-partner-violence-as-a-public-health-problem/.
3. World Health Organization. *Voilence against women*. 2021; Available from: https://www.who.int/news-room/fact-sheets/detail/violence-against-women.
4. Office for National Statistics (ONS), *Domestic abuse prevalence and victim characteristics Year ending March 2022*, Office for National Statistics (ONS), Editor. 2022.
5. World Health Organization, *Global and regional estimates of violence against women: prevalence and health effects of intimate partner violence and non-partner sexual violence*. 2013: World Health Organization.
6. Femi-Ajao, O., S. Kendal, and K. Lovell, *A qualitative systematic review of published work on disclosure and help-seeking for domestic violence and abuse among women from ethnic minority populations in the UK.* Ethnicity & Health, 2020. **25**(5): p. 732-746.
7. AVA, A., *Breaking down the barriers: findings of the national commission on domestic and sexual violence and multiple disadvantage*. 2020.
8. Donovan, C., et al., *Comparing domestic abuse in same sex and heterosexual relationships.* Unpublished manuscript, University of Sunderland, Sunderland, England, and University of Bristol, Bristol, England, 2006.
9. Roch, A., G. Ritchie, and J. Morton, *Transgender people's experiences of domestic abuse.* Scotland: LGBT Youth Scotland and the Equality Network, 2010.
10. Valentine, S.E., et al., *Disparities in exposure to intimate partner violence among transgender/gender nonconforming and sexual minority primary care patients.* LGBT health, 2017. **4**(4): p. 260-267.
11. Langenderfer-Magruder, L., et al., *Experiences of intimate partner violence and subsequent police reporting among lesbian, gay, bisexual, transgender, and queer adults in Colorado: Comparing rates of cisgender and transgender victimization.* Journal of interpersonal violence, 2016. **31**(5): p. 855-871.
12. Khalifeh, H., et al., *Violence against people with disability in England and Wales: findings from a national cross-sectional survey.* PloS one, 2013. **8**(2): p. e55952.
13. Trevillion, K., et al., *Experiences of domestic violence and mental disorders: a systematic review and meta-analysis.* PloS one, 2012. **7**(12): p. e51740.
14. Fulu, E., et al., *Prevalence of and factors associated with male perpetration of intimate partner violence: findings from the UN Multi-country Cross-sectional Study on Men and Violence in Asia and the Pacific.* The lancet global health, 2013. **1**(4): p. e187-e207.
15. Herbert, A., et al., *Risk factors for intimate partner violence and abuse among adolescents and young adults: findings from a UK population-based cohort.* Wellcome open research, 2020. **5**.
16. Hester, M., *Who Does What to Whom?: Gender and Domestic Violence Perpetrators*. 2009: University of Bristol [in association with] Northern Rock Foundation Bristol.
17. Robinson, A.L., *Serial domestic abuse in Wales: an exploratory study into its definition, prevalence, correlates, and management.* Victims & Offenders, 2017. **12**(5): p. 643-662.
18. Gilchrist, G., et al., *The prevalence and factors associated with ever perpetrating intimate partner violence by men receiving substance use treatment in Brazil and England: A cross-cultural comparison.* Drug and Alcohol Review, 2017. **36**(1): p. 34-51.
19. Basile, K.C., et al., *National intimate partner and sexual violence survey: 2010 summary report.* 2011.

For peer review only - http://bmjopen.bmj.com/site/about/guidelines.xhtml

20. Breiding, M.J., M.C. Black, and G.W. Ryan, *Chronic disease and health risk behaviors associated with intimate partner violence—18 US states/territories, 2005.* Annals of epidemiology, 2008. **18**(7): p. 538-544.
21. Chandan, J.S., et al., *Risk of cardiometabolic disease and all-cause mortality in female survivors of domestic abuse.* Journal of the American Heart Association, 2020. **9**(4): p. e014580.
22. Crofford, L.J., *Violence, stress, and somatic syndromes.* Trauma, Violence, & Abuse, 2007. **8**(3): p. 299-313.
23. John, R., et al., *Domestic violence: prevalence and association with gynaecological symptoms.* BJOG: An International Journal of Obstetrics & Gynaecology, 2004. **111**(10): p. 1128-1132.
24. Leserman, J. and D.A. Drossman, *Relationship of abuse history to functional gastrointestinal disorders and symptoms: some possible mediating mechanisms.* Trauma, Violence, & Abuse, 2007. **8**(3): p. 331-343.
25. Tjaden, P. and N. Thoennes, *Prevalence and consequences of male-to-female and female-to-male intimate partner violence as measured by the National Violence Against Women Survey.* Violence against women, 2000. **6**(2): p. 142-161.
26. McManus, S., et al., *Receiving threatening or obscene messages from a partner and mental health, self-harm and suicidality: results from the Adult Psychiatric Morbidity Survey.* Social psychiatry and psychiatric epidemiology, 2021: p. 1-11.
27. Povey, D., et al., *Homicides, Firearm Offences and Intimate Violence 2006/07 (Supplementary Volume 2 to Crime in England and Wales 2006/07) Home Office Statistical Bulletin 03/08 [online]. London: Home Office Research Development and Statistics Directorate*. 2008.
28. McManus, S., et al., *Intimate Partner Violence, Suicidality, and Self-Harm: A Probability Sample Survey of the General Population in England.* Suicidality, and Self-Harm: A Probability Sample Survey of the General Population in England, 2022.
29. Jonas, S., et al., *Gender differences in intimate partner violence and psychiatric disorders in England: results from the 2007 adult psychiatric morbidity survey.* Epidemiology and psychiatric sciences, 2014. **23**(2): p. 189-199.
30. St. Mungo's, *Rebuilding Shattered Lives [online]*. 2014.
31. Women's Aid, *The Domestic Abuse Report 2019: The Economics of Abuse Report Summary*. 2019.
32. Crown Prosecution Service, *Violence against Women and Girls Report 10th Edition 2016-17*. 2017.
33. Office for National Statistics (ONS), *Domestic abuse and the criminal justice system*. 2021.
34. ONS, *Domestic abuse in England and Wales overview: November 2021. Figures on domestic abuse from police recorded crime and a number of different organisations.* 2021.
35. Thiara, R.K., G. Hague, and A. Mullender, *Losing out on both counts: disabled women and domestic violence.* Disability & Society, 2011. **26**(6): p. 757-771.
36. Imkaan, *From Survival to Sustainability: Critical Issues for the Specialist Black and 'Minority Ethnic'Ending Violence Against Women and Girls Sector in the UK*. 2018, Imkaan London.
37. Colgan, F., C. Hunter, and A. McKearney, *'Staying alive': The impact of 'austerity cuts' on the LGBT Voluntary and Community Sector (VCS) in England and Wales*. 2014, TUC.
38. Yakubovich, A.R., et al., *Housing interventions for women experiencing intimate partner violence: a systematic review.* The Lancet Public Health, 2021.
39. Kiani, Z., et al., *A systematic review: Empowerment interventions to reduce domestic violence?* Aggression and Violent Behavior, 2021. **58**: p. 101585.
40. Rivas, C., et al., *Advocacy interventions to reduce or eliminate violence and promote the physical and psychosocial well-being of women who experience intimate partner abuse: A systematic review.* Campbell Systematic Reviews, 2016. **12**(1): p. 1-202.

For peer review only - http://bmjopen.bmj.com/site/about/guidelines.xhtml

41. Hameed, M., et al., *Psychological therapies for women who experience intimate partner violence.* Cochrane database of systematic reviews, 2020(7).
42. Advance, *Annual Report and Accounts*. 2021.
43. Refuge, *Annual Report 2020–2021 and financial statements*. 2021.
44. Yellow Door, *Annual Review and Accounts 2020-2021*. 2021.
45. Oasis, *Impact*. 2019.
46. Holly Taylor-Dunn and Rosie Erol, *Evaluation of SaferPlaces' Independent Domestic Violence Advisor Services.* 2019.
47. Lilley-Walker, S.-J., M. Hester, and W. Turner, *Evaluation of European domestic violence perpetrator programmes: Toward a model for designing and reporting evaluations related to perpetrator treatment interventions.* International journal of offender therapy and comparative criminology, 2018. **62**(4): p. 868-884.
48. Akoensi, T.D., et al., *Domestic violence perpetrator programs in Europe, Part II: A systematic review of the state of evidence.* International journal of offender therapy and comparative criminology, 2013. **57**(10): p. 1206-1225.
49. National Institute for Health and Care Excellence. *Domestic violence and abuse: multi-agency working: Public health guideline [PH50]. Recommendations for research*. 2014; Available from: https://www.nice.org.uk/guidance/ph50/chapter/5-Recommendations-for-research.
50. James Lind Alliance. *Sexual Violence Top 10*. 2022; Available from: https://www.jla.nihr.ac.uk/priority-setting-partnerships/sexual-violence/top-10-priorities.htm.
51. Powell, C., et al., *Development of core outcome sets for family and child-focused interventions for child maltreatment and domestic violence*. 2021.
52. Munn, Z., et al., *Systematic review or scoping review? Guidance for authors when choosing between a systematic or scoping review approach.* BMC medical research methodology, 2018. **18**: p. 1-7.
53. Peterson, J., et al., *Understanding scoping reviews: Definition, purpose, and process.* Journal of the American Association of Nurse Practitioners, 2017. **29**(1): p. 12-16.
54. Tricco, A.C., et al., *PRISMA extension for scoping reviews (PRISMA-ScR): checklist and explanation.* Annals of internal medicine, 2018. **169**(7): p. 467-473.
55. Carlisle, S., et al. *Measuring the effectiveness of UK-based support interventions and/or services targeted at adults who have experienced domestic and sexual violence and abuse (DSVA): A scoping review*. 2022; Available from: https://osf.io/frh2e.
56. Home Office, *Policy Paper: Tackling perpetrators*. 2022.
57. Ouzzani, M., et al., *Rayyan — a web and mobile app for systematic reviews.* Systematic Reviews, 2016. **5**(210).
58. Beck, A.T., R.A. Steer, and G.K. Brown, *Beck depression inventory*. 1987: Harcourt Brace Jovanovich New York:.
59. ADVA. and S.P. Associates., *REPAIR (Resolve to End the Perpetration of Abuse in Relationships): A Community- and Whole-family-based Intervention Programme Targeting Perpetrators of Domestic Violence and Abuse in Devon. An evaluation of a three-year Invest to Save (ISB) PROJECT*. 2009: Exeter.
60. Advance, *'A Place to go Like This': Breaking the cycle of harm for mothers involved in offending who are survivors of domestic abuse, and their children.* 2020.
61. Barber, M., R. Jenkins, and C. Jones, *A survivor's group for women who have a learning disability.* British Journal of Developmental Disabilities, 2000. **46**(90, Pt 1): p. 31-41.
62. Bloomfield, S. and L. Dixon, *An outcome evaluation of the integrated domestic abuse programme (IDAP) and community domestic violence programme (CDVP).* London: National Offender Management Service, 2015.

For peer review only - http://bmjopen.bmj.com/site/about/guidelines.xhtml

63. Bowen, E., *Evaluation of a community based domestic violence offender rehabilitation programme. Unpublished doctoral thesis*, in *Centre for Forensic and Family Psychology*. 2004, University of Birmingham.
64. Bowen, E., *Therapeutic Environment and Outcomes in a UK Domestic Violence Perpetrator Program.* Small Group Research, 2010. **41**(2): p. 198-220.
65. Bowen, E. and E. Gilchrist, *Predicting dropout of court-mandated treatment in a British sample of domestic violence offenders.* Psychology, Crime & Law, 2006. **12**(5): p. 573-587.
66. Bowen, E., E. Gilchrist, and A.R. Beech, *Change in treatment has no relationship with subsequent re-offending in UK domestic violence sample: A preliminary study.* International Journal of Offender Therapy and Comparative Criminology, 2008. **52**(5): p. 598-614.
67. Bowen, E., E.A. Gilchrist, and A.R. Beech, *An Examination of the Impact of Community-Based Rehabilitation on the Offending Behaviour of Male Domestic Violence Offenders and the Characteristics Associated with Recidivism.* Legal and Criminological Psychology, 2005. **10**(2): p. 189-209.
68. Brady, F., et al., *Narrative exposure therapy for survivors of human trafficking: feasibility randomised controlled trial.* BJPsych Open, 2021. **7**(6).
69. Burton, S., L. Regan, and L. Kelly, *Supporting women and challenging men: lessons from the Domestic Violence Intervention Project.* 1998.
70. Callaghan, J., et al., *Make a Change: An evaluation of the implementation of an early response intervention for those who have used abusive behaviours in their intimate relationships*. 2020.
71. Respect and Women's Aid Federation England, *Make a Change Executive Summary*.
72. Calvert, R., S. Kellett, and T. Hagan, *Group cognitive analytic therapy for female survivors of childhood sexual abuse.* British Journal of Clinical Psychology, 2015. **54**(4): p. 391-413.
73. Clarke, S. and S. Llewelyn, *Personal constructs of survivors of childhood sexual abuse receiving cognitive analytic therapy.* British Journal of Medical Psychology, 1994. **67(3)**: p. 273-289.
74. Clarke, S. and C. Pearson, *Personal constructs of male survivors of childhood sexual abuse receiving cognitive analytic therapy.* British Journal of Medical Psychology, 2000. **73(2)**: p. 169-177.
75. Cook, D.A., et al., *The Berkeley group: ten years' experience of a group for non-violent sex offenders.* The British Journal of Psychiatry, 1991. **158**(2): p. 238-243.
76. Co-Ordinated Action Against Domestic, A., *Insights into domestic abuse 1: a place of greater safety.* 2012.
77. Co-ordinated Action Against Domestic Abuse (CAADA), *Insights National Dataset 2011–12 Appendix to: A place of greater safety*. 2012.
78. Coy, M. and L. Kelly, *Islands in the stream: an evaluation of four London independent domestic violence advocacy schemes*. 2011.
79. Dheensa, S., G. Penny, and Safelink, *"In my way…" Evaluation Of Learning Disabilities Sexual Abuse Support Services*.
80. Dobash, R.P., et al., *A research evaluation of British programmes for violent men.* Journal of Social Policy, 1999. **28**(2): p. 205-233.
81. Ellis, F., *Rehabilitation programme for adult survivors of childhood sexual abuse.* Journal of Public Mental Health, 2012. **11**(2): p. 88-92.
82. Farrell, G. and A. Buckley, *Evaluation of a UK police domestic violence unit using repeat victimisation as a performance indicator.* The Howard Journal of Criminal Justice, 1999. **38**(1): p. 42-53.
83. Ferrari, G., et al., *Psychological advocacy towards healing (PATH): A randomized controlled trial of a psychological intervention in a domestic violence service setting.* Plos One, 2018. **13**(11).

For peer review only

84. Friendship, C., R.E. Mann, and A.R. Beech, *Evaluation of a national prison-based treatment program for sexual offenders in England and Wales.* Journal of interpersonal violence, 2003. **18**(7): p. 744-759.
85. Garry, K. and P. Goodwin, *Adult Counselling Service Phase two evaluation report: year 1 2020-21*. 2021, Merida Associates.
86. Karen Garry and Ellie Munro, *Adult Counselling Service Interim evaluation | May 2019*. 2019.
87. Karen Garry and Ellie Munro, *Adult Counselling Service Evaluation report – phase one 2016-2020*. 2020.
88. Geoghegan-Fittall, S., C. Keeble, and D. Wunsch, *The Croydon Drive Project: A 2-year Evaluation Final Report*. 2020.
89. Gilchrist, E., et al., *Using the Behaviour Change Wheel to design an intervention for partner abusive men in drug and alcohol treatment.* Pilot and feasibility studies, 2021. **7**(1): p. 1-14.
90. Gilchrist, G., et al., *ADVANCE integrated group intervention to address both substance use and intimate partner abuse perpetration by men in substance use treatment: a feasibility randomised controlled trial.* BMC public health, 2021. **21**(1): p. 1-20.
91. Goosey, J., L. Sherman, and P. Neyroud, *Integrated case management of repeated intimate partner violence: A randomized, controlled trial.* Cambridge Journal of Evidence-Based Policing, 2017. **1**(2): p. 174-189.
92. Halliwell, G., et al., *Cry for health: a quantitative evaluation of a hospital-based advocacy intervention for domestic violence and abuse.* Bmc Health Services Research, 2019. **19**(1).
93. Hester, M., et al., *Evaluation of Year 2 of the Drive Project – A Pilot to Address High Risk Perpetrators of Domestic Abuse*. 2019.
94. Hester, M., et al., *Evaluation of the Drive Project – a pilot to address high-risk perpetrators of domestic abuse Year 1 Feasibility Study*. 2017.
95. Hester, M., et al., *Evaluation of the Drive Project – A Three-year Pilot to Address High-risk, High-harm Perpetrators of Domestic Abuse*. 2019.
96. Hester, M., et al., *Evaluation of the Drive Project – A Three-year Pilot to Address High-risk, High-harm Perpetrators of Domestic Abuse EXECUTIVE SUMMARY.* 2020.
97. Hester, M. and N. Westmarland, *Tackling domestic violence: effective interventions and approaches*. 2005: Home Office Research, Development and Statistics Directorate.
98. Howarth, E. and et al., *Safety in numbers: a multi-site evaluation of Independent Domestic Violence Advisor Services.* 2009.
99. Howarth, E., et al., *Safety in numbers: summary of findings and recommendations from a multi-site evaluation of Independent Domestic Violence Advisors.* 2009.
100. Howarth, E. and A. Robinson, *Responding Effectively to Women Experiencing Severe Abuse: Identifying Key Components of a British Advocacy Intervention.* Violence against Women, 2016. **22**(1): p. 41-63.
101. Karatzias, T., et al., *Group psychotherapy for female adult survivors of interpersonal psychological trauma: a preliminary study in Scotland.* Journal of Mental Health, 2016. **25**(6): p. 512-519.
102. Kelley, T.M., et al., *The Efficacy of Principle-Based Correctional Counseling for Improving the Self-Control and Mental Health of People Incarcerated for Sexual Violence.* Violence Against Women, 2022. **28**(2): p. 573-592.
103. Kelly, L., *Domestic Violence Matters: an evaluation of a development project*. 1999.
104. Kelly, L. and N. Westmarland, *DOMESTIC VIOLENCE PERPETRATOR PROGRAMMES STEPS TOWARDS CHANGE: Project Mirabal Final Report*. 2015.
105. Koppensteiner, M.F., J. Matheson, and R. Plugor, *Project 360: An intervention to address victim-police engagement in repeat domestic violence cases*. 2017.
106. Lindsay, W.R., et al., *Comparing offenders against women and offenders against children on treatment outcome in offenders with intellectual disability.* Journal of Applied Research in Intellectual Disabilities, 2011. **24**(4): p. 361-369.

For peer review only - http://bmjopen.bmj.com/site/about/guidelines.xhtml

107. Lindsay, W.R., et al., *The treatment of six men with a learning disability convicted of sex offences with children.* British Journal of Clinical Psychology, 1998. **37**: p. 83-98.
108. Lowe, M., et al., *CORE assessment of adult survivors abused as children: A NAPAC group therapy evaluation.* Counselling & Psychotherapy Research, 2017. **17**(1): p. 71-79.
109. McConnell, N., et al., *Caring dads safer children*. 2014.
110. McConnell, N., M. Barnard, and J. Taylor, *Caring Dads Safer Children: Families' Perspectives on an Intervention for Maltreating Fathers.* Psychology of Violence, 2017. **7**(3): p. 406-416.
111. McCracken, K. and T. Deave, *Evaluation of the Caring Dads Cymru programme. Final report.* 2012.
112. Michie, A.M. and W.R. Lindsay, *A treatment component designed to enhance empathy in sex offenders with an intellectual disability.* The British Journal of Forensic Practice, 2012. **14**(1): p. 40-48.
113. Morgan, S. and J. Parkes, *The Hampshire Domestic Abuse Prevention Partnership 2016-2018: process and outcomes evaluation.* 2018.
114. Morgan, S.A., B.M.S. McCausland, and J. Parkes, *Baseline characteristics and outcomes of the main perpetrator programme within the Hampshire Domestic Abuse Prevention Partnership, UK: A mixed methods study.* Plos One, 2019. **14**(7).
115. Morrison, A. and L. Treliving, *Evaluation of outcome in a dynamically orientated group for adult males who have been sexually abused in childhood.* British Journal of Psychotherapy, 2002. **19**: p. 59-75.
116. Murphy, G., et al., *Cognitive-behavioural treatment for men with intellectual disabilities and sexually abusive behaviour: A pilot study.* Journal of Intellectual Disability Research, 2007. **51(11)**: p. 902-912.
117. Oasis. *Impact report 2013-2014*. 2014; Available from: https://issuu.com/kristinagwynne/docs/oasis_annual_report_2013-2014-web/1.
118. Oasis, *Impact Report 2015-2016.* 2016.
119. Ormston, R., C. Mullholland, and L. Setterfield, *Caledonian system evaluation: analysis of a programme for tackling domestic abuse in Scotland*. 2016, Edinburgh: Scotland. Scottish Government Social Research. vi, 88.
120. Payne, A., H. Liebling-Kalifani, and S. Joseph, *Client-centred group therapy for survivors of interpersonal trauma: A pilot investigation.* Counselling & Psychotherapy Research, 2007. **7**(2): p. 100-105.
121. Peckham, N.G., S. Howlett, and A. Corbett, *Evaluating a survivors group pilot for a women with significant intellectual disabilities who have been sexually abused.* Journal of Applied Research in Intellectual Disabilities, 2007. **20**(4): p. 308-322.
122. Robinson, A.L., *The Cardiff Women's Safety Unit: A multi-agency approach to domestic violence.* Final Evaluation Report. Cardiff University: School of Social Sciences.(Available online at http://www. cf. ac. uk/socsi/whoswho/robinson. html), 2003.
123. Robinson, A.L., *Domestic Violence MARACs (Multi-Agency Risk Assesment Conferences) for Very High-risk Victims in Cardiff, Wales: A Process and Outcome Evaluation.* 2004.
124. Robinson, A.L., *Reducing repeat victimization among high-risk victims of domestic violence: The benefits of a coordinated community response in Cardiff, Wales.* Violence Against Women, 2006. **12(8)**: p. 761-788.
125. Robjant, K., J. Roberts, and C. Katona, *Treating posttraumatic stress disorder in female victims of trafficking using narrative exposure therapy: A retrospective audit.* Frontiers in Psychiatry, 2017. **8**.
126. Rose, J., et al., *A sex offender treatment group for men with intellectual disabilities in a community setting.* The British Journal of Forensic Practice, 2012.
127. Ross, J., J. Sebire, and H. Strang, *Tracking Repeat Victimisation After Domestic Abuse Cases Are Heard With and Without Independent Domestic Violence Advisors (IDVAs) in an English Magistrate's Court.* Cambridge Journal of Evidence-Based Policing, 2022: p. 1-15.

For peer review only - http://bmjopen.bmj.com/site/about/guidelines.xhtml

128. Ryan, M., et al., *A prospective study of the effectiveness of group and individual psychotherapy for women CSA survivors.* Psychology and Psychotherapy: Theory, Research and Practice, 2005. **78(4)**: p. 465-480.
129. SafeLives, *Insights refuge England and Wales dataset 2015-18 Adult refuge services*. 2018.
130. SafeLives, *Defining Marac Outcomes National Pilot*. 2012.
131. SafeLives, *Insights England and Wales dataset 2018-19 Sexual violence services* 2019.
132. SafeLives, *Insights SV dataset 2020-21 Sexual violence (SV) services*. 2021.
133. SafeLives, *Insights health England and Wales dataset 2015-2018 Adults health-based services*. 2018.
134. SafeLives, *Insights helpline England and Wales dataset 2014-17 Adult helpline services*. 2017.
135. SafeLives, *Insights Idva national dataset 2012–13 Adult independent domestic violence advisor (Idva) services*. 2015.
136. SafeLives, *Insights Idva national dataset 2013–14 Adult independent domestic violence advisor (Idva) services*. 2015.
137. SafeLives, *Insights Idva England and Wales dataset 2016-17 Adult independent domestic violence advisor (Idva) services*. 2017.
138. SafeLives, *Insights Idva England and Wales dataset 2018-19 Adult Independent domestic violence advisor (Idva) services*. 2019.
139. SafeLives, *Insights Idva dataset 2020-21 Adult Independent domestic violence advisor (Idva) services*. 2021.
140. SafeLives, *Insights outreach national dataset 2012–13 Adult outreach services*. 2015.
141. SafeLives, *Insights outreach national dataset 2013–14 Adult outreach services.* 2015.
142. SafeLives, *Insights outreach England and Wales dataset 2016-17 Adult outreach services*. 2017.
143. SafeLives, *Insights outreach England and Wales dataset 2018-19 Adult outreach services*. 2019.
144. SafeLives, *Insights outreach dataset 2020-21 Adult outreach services*. 2021.
145. Sharpe, J., et al., *Group analytic therapy for male survivors of childhood sexual abuse.* Group Analysis, 2001. **34**: p. 195-209.
146. Skyner, D.R. and J. Waters, *Working with perpetrators of domestic violence to protect women and children: a partnership between Cheshire Probation Service and the NSPCC.* Child Abuse Review: Journal of the British Association for the Study and Prevention of Child Abuse and Neglect, 1999. **8**(1): p. 46-54.
147. Smith, D., et al., *Adults with a history of child sexual abuse: Evaluation of a pilot therapy service.* British Medical Journal, 1995. **310(6988)**: p. 1175-1178.
148. Smith, E., et al., *Strengthening the mother-child relationship following domestic abuse: Service evaluation.* Child Abuse Review, 2015. **24**(4): p. 261-273.
149. Stanley, N., et al., *An evaluation of a new initiative for male perpetrators of domestic violence* 2011.
150. Stanley, N. and a. et, *Roadmap evaluation: final report - VOICES*. 2021, Bristol: Women's Aid SafeLives. 279.
151. Stanley, N. and a. et, *Roadmap evaluation: final report - SLCDPs*. 2021, Bristol: Women's Aid SafeLives. 279.
152. Strang, H., et al., *Reducing the harm of intimate partner violence: Randomized controlled trial of the Hampshire Constabulary CARA Experiment.* Cambridge Journal of Evidence-Based Policing, 2017. **1**(2): p. 160-173.
153. Taylor-Dunn, H. and R. Erol, *Improving the safety and well-being of domestic abuse survivors: The role of a specialist organisation in supporting the work of Independent Domestic Violence Advisors.* Crime Prevention and Community Safety, 2021. **23**(2): p. 115-136.
154. Eve; The Institute for Public Safety, C.J., *2019*. Evaluation of SHE.

For peer review only - http://bmjopen.bmj.com/site/about/guidelines.xhtml

155. Trevillion, K., et al., *Linking abuse and recovery through advocacy: An observational study.* Epidemiology and Psychiatric Sciences, 2014. **23**(1): p. 99-113.
156. Vallely, C., *Evaluation of domestic violence pilot sites at Caerphilly (Gwent) and Croydon 2004/05.* 2005.
157. Webster, R., *Kent and Medway Independent Domestic Violence Advisor Service Impact Evaluation FINAL REPORT*. 2015.
158. Westmarland, N. and S. Alderson, *The Health, Mental Health, and Well-Being Benefits of Rape Crisis Counseling.* Journal of Interpersonal Violence, 2013. **28**(17): p. 3265-3282.
159. Williamson, D. and A. Boyle, *Results of the domestic violence advocacy program at addenbrookes hospital.* Academic Emergency Medicine, 2012. **19**: p. 741.
160. Williamson, E. and M. Hester, *Evaluation of the South Tyneside Domestic Abuse Perpetrator Programme (STDAPP) 2006-2008.* University of Bristol, 2009.
161. Miles, C. and K. Smith, *Nowhere to turn, 2018: findings from the second year of the No Woman Turned away project*. 2018, Bristol: Women's Aid. 48.
162. Women's Aid, *Nowhere To Turn, 2022: Findings from the sixth year of the No Woman Turned Away project*. 2022, Women's Aid: Bristol.
163. Women's Aid, *NOWHERE TO TURN Findings from the first year of the No Woman Turned Away project*. 2017.
164. Women's Aid, *NOWHERE TO TURN Findings from the third year of the No Woman Turned Away project*. 2019.
165. Women's Aid, *NOWHERE TO TURN Findings from the fourth year of the No Woman Turned Away project*. 2020.
166. Women's Aid, *NOWHERE TO TURN 2021 Findings from the fifth year of the No Woman Turned Away project*. 2021.
167. Women's Resource, C., *Women and Girls Network: the WGN empowerment model - redressing negative individual and social impacts of gendered violence through holistic counselling.* 2011.
168. Women's Resource, C., *Ashiana Network: journey towards safety.* 2011.
169. Campbell, J.C. and K.L. Soeken, *Women's responses to battering over time: an analysis of change.* Journal of Interpersonal violence, 1999. **14**(1): p. 21-40.
170. Rosenberg, M., *Rosenberg self-esteem scale.* Journal of Religion and Health, 1965.
171. Evans, C., et al., *Towards a standardised brief outcome measure: Psychometric properties and utility of the CORE–OM.* The British Journal of Psychiatry, 2002. **180**(1): p. 51-60.
172. Derogatis, L.R., *SCL-90-R: Administration, scoring & procedures manual-II for the (revised) version and other instruments of the psychopathology rating scale series.* Clinical Psychometric Research., 1992: p. 1-16.
173. Kroenke, K., R.L. Spitzer, and J.B. Williams, *The PHQ-9: validity of a brief depression severity measure.* Journal of general internal medicine, 2001. **16**(9): p. 606-613.
174. Spitzer, R.L., et al., *A brief measure for assessing generalized anxiety disorder: the GAD-7.* Archives of internal medicine, 2006. **166**(10): p. 1092-1097.
175. Sherman, L., P.W. Neyroud, and E. Neyroud, *The Cambridge crime harm index: Measuring total harm from crime based on sentencing guidelines.* Policing: a journal of policy and practice, 2016. **10**(3): p. 171-183.
176. Prins, A., et al., *The primary care PTSD screen for DSM-5 (PC-PTSD-5).[measurement instrument].* Universität Bielefeld: Bielefeld, Germany, 2015.
177. Women Against Violence Europe, *WAVE Report 2015: On the Role of Specialist Women's Support Services in Europe*. 2016.
178. Women's Aid. *What we believe: Our values*. 2022; Available from: https://www.womensaid.org.uk/what-we-believe/.

For peer review only - http://bmjopen.bmj.com/site/about/guidelines.xhtml

179. Robotham, D., A. Sweeney, and C. Perôt, *Violence, abuse and mental health network: Survivors' priority themes and questions for research—Consultation report, 2019.* Retrieved June, 2019. **26**: p. 2020.
180. Macy, R.J., et al., *Domestic violence and sexual assault services: Historical concerns and contemporary challenges.* Journal of Interpersonal Violence, 2010. **25**(1): p. 3-32.
181. SafeLives, *We only do bones here': Why London needs a whole-health approach to domestic abuse*. 2021, SafeLives: London.
182. Wiley, K., *Implementing Domestic Violence Policy: When Accountability Trumps Mission.* Affilia, 2020. **35**(4): p. 533-551.
183. Women's Aid, *Fragile funding landscape: The extent of local authority commissioning in the domestic abuse refuge sector in England 2020*. 2021, Women's Aid: Bristol.
184. Towers, J. and S. Walby, *Measuring the impact of cuts in public expenditure on the provision of services to prevent violence against women and girls.* 2012.
185. Corry, D., *Hearing women's voices: Why women 2018.* . 2018.
186. Heady, L., A. Kail, and C. Yeowart, *Understanding the stability and sustainability of the violence against women voluntary sector.* 2011.
187. Sullivan, R. and E. Sharples, *The CSA Centre's Evaluation Fund: A Reflection*. 2018, Barkingside: Centre of expertise on child sexual abuse.
188. Home Office, *Violence against women and girls services: commissioning toolkit*. 2022, Home Office: London.

For peer review only

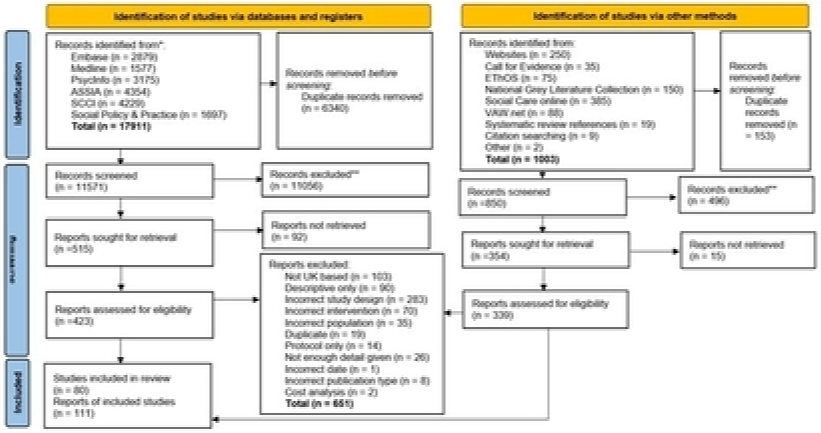

PRISMA flow diagram

35x18mm (300 x 300 DPI)

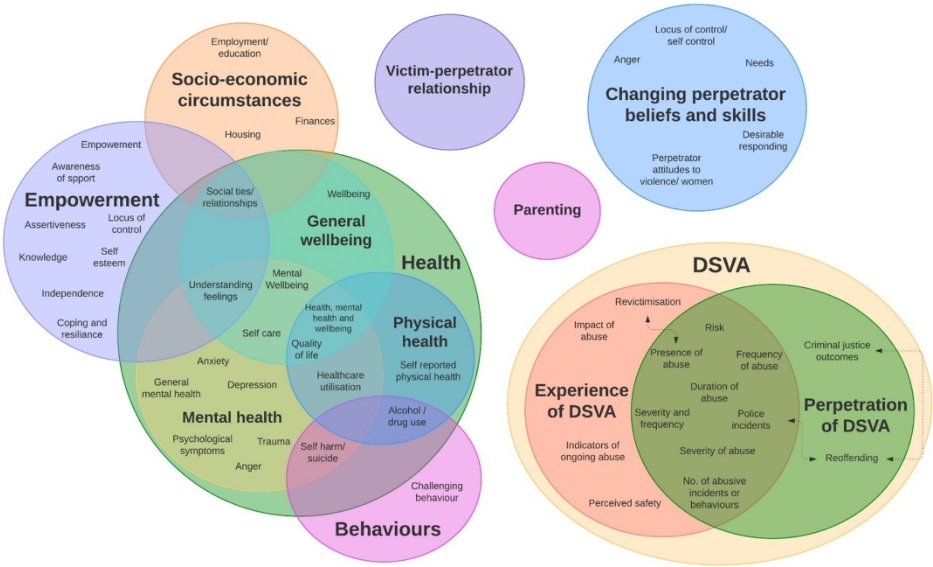

Venn diagram of outcome categories, domains and subdomains

167x99mm (300 x 300 DPI)

Appendix 1: Glossary

Advocacy: Adapted from the National Institute for Health and Clinical Excellence (NICE) Domestic Violence and Abuse: Multi-agency Working public health guideline (PH50)[1]. In general, advocacy for people who have experienced DSVA includes: legal, housing and financial advice: access to and use of community resources such as refuges, emergency housing and psychological interventions; and safety planning advice. The activities may differ according to the level of risk facing the person. Crisis advocacy involves working with the person for a limited period of time (they may then be referred on to more specialist agencies). Practitioners providing advocacy can also provide ongoing support and informal counselling. The intensity of the advocacy provided may vary. It may last for a year – or longer, if the person is particularly vulnerable.

By-and-for: By-and-for services are lived experience organisation, where all of the staff, senior managers, Board of Trustees, and women and girls using services are Black and minoritised women. Such services have a Black feminist ethos, locating themselves as anti-racist, social justice and intersectional organisations.

Domestic and sexual violence and abuse (DSVA): This review works to the UK government definition of domestic violence and abuse[2], and The Istanbul Convention[3], The World Health Organisation[4], and The Rome Statute of the International Criminal Court's (ICC) Elements of Crimes definitions of sexual violence and abuse[5]. In brief, domestic violence and abuse is defined as behaviour within an intimate relationship or family which includes acts of physical violence, sexual violence, emotional or psychological abuse, and controlling behaviours. This also includes 'honour'-based violence and FGM. Sexual violence and abuse is defined as any non-consensual act of sexual nature, extending to attempts to obtain a sexual act, unwanted sexual comments or advances, or acts to traffic, using force, threat of force or coercion.

Independent domestic violence advisers (IDVAs): Adapted from the NICE PH50 guideline. Also known as independent domestic violence advocates, IDVAs work primarily with people at high risk of domestic violence and abuse, independently of any one agency, to secure their safety and the safety of their children. Serving as the primary point of contact, IDVAs normally work with their clients from the point of crisis to assess the level of risk, discuss the options and develop plans that address their immediate safety, as well as longer-term solutions. In many areas they are funded by the local community safety partnership, in some areas they are funded by the police or local authorities.

Intervention: Adapted from the NICE Glossary. A treatment, service, project, or programme.

Intimate partner violence (IPV): Adapted from the World Health Organisation's definition. Intimate partner violence refers to behaviour within an intimate relationship that causes physical, sexual or psychological harm, including acts of physical aggression, sexual coercion, psychological abuse and controlling behaviours. This definition covers violence by both current and former spouses and partners.

Organisation: In the context of this review, organisations refer exclusively to those that carry out activities relating to DSVA. Organisations may include both specialist service providers, and organisations that do not directly provider services (such as women's rights organisations that may focus on activities such as campaigning, research, supporting specialist services, training, and education, for issues relating to DSVA, for example).

For peer review only

People who have experienced DSVA: Throughout this review, people who experience DSVA refers to those who are victims or survivors of DSVA.

Perpetrator programmes: Any group or individual intervention for people who have perpetrated DSVA, with the aim of reducing the incidence of violence by changing attitudes, behaviours and/or beliefs of perpetrators.

Primary, secondary, and tertiary prevention: Primary prevention refers to any action, strategy or policy that works to stop domestic violence from occurring in the first place. Primary prevention seeks to reduce the overall likelihood that anyone will become a victim or perpetrator of domestic violence by creating conditions that make violence less likely to occur. Secondary prevention refers to intervening and responding to violence that has already occurred in order to stop violence from happening again. Secondary prevention activities can include refuge, counselling, and safety planning. Tertiary prevention refers to ongoing support to victims and ongoing accountability for abusers. Tertiary prevention activities address the long-term consequences of domestic violence. They can include support groups and other resources for survivors to help them heal so that they do not experience abuse again. They can also include perpetrator programs for abusers to prevent them from continuing to perpetrate violence in the future.

Refuge or shelter: Adapted from the NICE PH50 guideline. Residential service – a safe house – provided for adults (usually women) and children who are experiencing domestic violence and abuse.

Service provider: Primary, community, including third sector, secondary and tertiary care providers of health and social care services, including prison health services and criminal justice agencies. In the context of DSVA, the service providers are predominantly third sector providers. Third sector service providers may be specialist DSVA providers (i.e., they only provide services relating to DSVA) or non-specialist-DSVA providers (i.e., they provide specialist support services for DSVA as well as support services for other issues). Outside of the third sector, service providers such as the NHS, police and courts may provide some DSVA specific services.

Third sector organisations: In this review the definition of third sector services provided by the National Audit Office has been adopted. ‘Third sector organisations’ is a term used to describe the range of organisations that are neither public sector nor private sector. It includes voluntary and community organisations (both registered charities and other organisations such as associations, self-help groups and community groups), social enterprises, mutuals and co-operatives. Third sector organisations generally: are independent of government. This is also an important part of the history and culture of the sector; are ‘value-driven’. This means they are motivated by the desire to achieve social goals (for example, improving public welfare, the environment or economic well-being) rather than the desire to distribute profit; and reinvest any surpluses generated in the pursuit of their goals. For this reason they are sometimes called ‘not-for-profit organisations’. A better term is ‘not-for-personal-profit’. In many cases, third sector organisations need to make surpluses (or ‘profits’) to be financially sustainable.

For peer review only

References

1. National Institute for Health and Care Excellence. *Domestic violence and abuse: multi-agency working: Public health guideline [PH50]. Recommendations for research*. 2014; Available from: https://www.nice.org.uk/guidance/ph50/chapter/5-Recommendations-for-research.
2. Home Office. *Circular 003/2013: new government domestic violence and abuse definition*. 2013; Available from: https://www.gov.uk/government/publications/new-government-domestic-violence-and-abuse-definition/circular-0032013-new-government-domestic-violence-and-abuse-definition.
3. Council of Europe, *Council of Europe Convention on preventing and combating violence against women and domestic violence (CETS No. 210)*. 2011.
4. World Health Organization, *World report on violence and health*. 2002.
5. International Criminal Court, *Elements of Crime*. 2013.

For peer review only

Appendix 2: Example search strategies

Search strategy development process

Search terms were identified during initial scoping and protocol development stages. An initial list of search terms was drafted based on terms found in the literature and based on search terms used in systematic reviews in related areas. This initial list of terms was shared with the review team and with the Patient and Public Involvement advisory group, who added any additional terms thought to be relevant to the review's research question.

Peer review example: Medline

1. exp United kingdom/
2. (national health service* or nhs*).ti,ab,in.
3. (english not ((published or publication* or translat* or written or language* or speak* or literature or citation*) adj5 english)).ti,ab.
4. (gb or "g.b." or britain* or (british* not "british columbia") or uk or "u.k." or united kingdom* or (england* not "new england") or northern ireland* or northern irish* or scotland* or scottish* or ((wales or "south wales") not "new south wales") or welsh*).ti,ab,jw,in.
5. (bangor or "bangor's" or cardiff or "cardiff's" or newport or "newport's" or st asaph or "st asaph's" or st davids or swansea or "swansea's").ti,ab,in,cq.
6. (bath or "bath's" or ((birmingham not alabama*) or ("birmingham's" not alabama*) or bradford or "bradford's" or brighton or "brighton's" or bristol or "bristol's" or carlisle* or "carlisle's" or (cambridge not (massachusetts* or boston* or harvard*)) or ("cambridge's" not (massachusetts* or boston* or harvard*)) or (canterbury not zealand*) or ("canterbury's" not zealand*) or chelmsford or "chelmsford's" or chester or "chester's" or chichester or "chichester's" or coventry or "coventry's" or derby or "derby's" or (durham not (carolina* or nc)) or ("durham's" not (carolina* or nc)) or ely or "ely's" or exeter or "exeter's" or gloucester or "gloucester's" or hereford or "hereford's" or hull or "hull's" or lancaster or "lancaster's" or leeds* or leicester or "leicester's" or (lincoln not nebraska*) or ("lincoln's" not nebraska*) or (liverpool not (new south wales* or nsw)) or ("liverpool's" not (new south wales* or nsw)) or ((london not (ontario* or ont or toronto*)) or ("london's" not (ontario* or ont or toronto*)) or manchester or "manchester's" or (newcastle not (new south wales* or nsw)) or ("newcastle's" not (new south wales* or nsw)) or norwich or "norwich's" or nottingham or "nottingham's" or oxford or "oxford's" or peterborough or "peterborough's" or plymouth or "plymouth's" or portsmouth or "portsmouth's" or preston or "preston's" or ripon or "ripon's" or salford or "salford's" or salisbury or "salisbury's" or sheffield or "sheffield's" or southampton or "southampton's" or st albans or stoke or "stoke's" or sunderland or "sunderland's" or truro or "truro's" or wakefield or "wakefield's" or wells or westminster or "westminster's" or winchester or "winchester's" or wolverhampton or "wolverhampton's" or (worcester not (massachusetts* or boston* or harvard*)) or ("worcester's" not (massachusetts* or boston* or harvard*)) or (york not ("new york*" or ny or ontario* or ont or toronto*)) or ("york's" not ("new york*" or ny or ontario* or ont or toronto*))))).ti,ab,in.
7. (aberdeen or "aberdeen's" or dundee or "dundee's" or edinburgh or "edinburgh's" or glasgow or "glasgow's" or inverness or (perth not australia*) or ("perth's" not australia*) or stirling or "stirling's").ti,ab,in.
8. (armagh or "armagh's" or belfast or "belfast's" or lisburn or "lisburn's" or londonderry or "londonderry's" or derry or "derry's" or newry or "newry's").ti,ab,in.
9. (exp africa/ or exp americas/ or exp antarctic regions/ or exp arctic regions/ or exp asia/ or exp australia/ or exp oceania/) not (exp United Kingdom/ or europe/)

10. (child adj3 maltreat*).ti,ab,kw.
11. (sexual adj3 harass*).ti,ab,kw.
12. (batter* adj3 (wom#n or wife or wives)).ti,ab,kw.
13. rape*.ti,ab,kw.
14. (beat* adj3 (partner* or spous* or wom#n or wife or wives or m#n or husband*)).ti,ab,kw.
15. (sexual adj3 coercion).ti,ab,kw.
16. female genital mutilation.ti,ab,kw.
17. forced marriage.ti,ab,kw.
18. sex traffic*.ti,ab,kw.
19. revenge porn.ti,ab,kw.
20. controlling behaviour.ti,ab,kw.
21. prostitut*.ti,ab,kw.
22. sex work*.ti,ab,kw.
23. stalking.ti,ab,kw.
24. (drink adj3 spik*).ti,ab,kw.

25. domestic abuse service*.ti,ab,kw.
26. women's project*.ti,ab,kw.
27. women's resource centre.ti,ab,kw.
28. safety unit*.ti,ab,kw.
29. domestic abuse support service*.ti,ab,kw.
30. domestic abuse team*.ti,ab,kw.
31. domestic violence intervention*.ti,ab,kw.
32. women's service*.ti,ab,kw.
33. women's rights organi#ation*.ti,ab,kw.
34. domestic abuse advice and support.ti,ab,kw.
35. women's centre*.ti,ab,kw.
36. domestic abuse project*.ti,ab,kw.
37. domestic and sexual abuse project*.ti,ab,kw.
38. victim care service*.ti,ab,kw.
39. support centre*.ti,ab,kw.
40. women's welfare.ti,ab,kw.
41. speciali* service*.ti,ab,kw.
42. speciali* sexual violence service*.ti,ab,kw.
43. speciali* domestic violence service*.ti,ab,kw.
44. speciali* support.ti,ab,kw.
45. speciali* violence and abuse service*.ti,ab,kw.
46. speciali* women's voluntary sector service*.ti,ab,kw.
47. service* for survivors.ti,ab,kw.
48. victim service*.ti,ab,kw.
49. victim support*.ti,ab,kw.
50. violence against women and girls service*.ti,ab,kw.
51. domestic violence support service*.ti,ab,kw.
52. community based service*.ti,ab,kw.
53. sexual assault referral centre*.ti,ab,kw.
54. rape crisis.ti,ab,kw.
55. third sector.ti,ab,kw.
56. second tier.ti,ab,kw.
57. crisis intervention.ti,ab,kw.
58. independent domestic violence advoca*.ti,ab,kw.
59. IDVA.ti,ab,kw.
60. independent sexual violence advoca*.ti,ab,kw.
61. ISVA.ti,ab,kw.

For peer review only

62. shelter*.ti,ab,kw.
63. refuge.ti,ab,kw.
64. refuges.ti,ab,kw.
65. advoca*.ti,ab,kw.
66. counsel?ing.ti,ab,kw.
67. outreach.ti,ab,kw.
68. helpline*.ti,ab,kw.
69. emergency shelter*.ti,ab,kw.
70. supportive hous*.ti,ab,kw.
71. violence against women service*.ti,ab,kw.
72. recovery service*.ti,ab,kw.
73. speciali* victim service*.ti,ab,kw.
74. independent domestic violence advisor*.ti,ab,kw.
75. independent sexual violence advisor*.ti,ab,kw.
76. resettlement.ti,ab,kw.
77. floating support.ti,ab,kw.
78. drop-in.ti,ab,kw.
79. online chat.ti,ab,kw.
80. support group*.ti,ab,kw.
81. violence advocacy project*.ti,ab,kw.
82. group work program*.ti,ab,kw.
83. information and advice.ti,ab,kw.
84. (dedicated adj4 service*).ti,ab,kw.
85. charit*.ti,ab,kw.
86. independent.ti,ab,kw.
87. voluntary.ti,ab,kw.
88. public sector.ti,ab,kw.
89. specialist.ti,ab,kw.
90. speciali?ed.ti,ab,kw.
91. non-governmental organi#ation*.ti,ab,kw.
92. nonprofit organi#ation*.ti,ab,kw.
93. not-for-profit organi#ation*.ti,ab,kw.
94. by and for service*.ti,ab,kw.
95. by and for organi#ation*.ti,ab,kw.
96. therapy.ti,ab,kw.
97. empowerment.ti,ab,kw.
98. case management.ti,ab,kw.
99. stress management.ti,ab,kw.
100. safety behaviour*.ti,ab,kw.
101. safety strateg*.ti,ab,kw.
102. legal advice.ti,ab,kw.
103. mindfulness based stress reduction.ti,ab,kw.
104. home visit*.ti,ab,kw.
105. sanctuar*.ti,ab,kw.
106. safe house*.ti,ab,kw.
107. flexible fund*.ti,ab,kw.
108. exp battered woman/
109. exp domestic violence/ or exp child abuse/ or exp spouse abuse/ or exp gender-based violence/ or exp intimate partner violence/ or exp physical abuse/ or exp rape/
110. exp partner violence/
111. exp family violence/
112. exp coercion/
113. exp emotional abuse/
114. exp sexual violence/ or exp sexual abuse/ or exp sexual assault/

For peer review only

115. ("City of London" or Bedfordshire or Berkshire or Bristol or Buckinghamshire or Cambridgeshire or Cheshire or Cornwall or Cumbria or Derbyshire or Devon or Dorset or Durham or Yorkshire or Essex or Gloucestershire or "greater london" or "greater manchester" or "isle of wight" or "west midlands" or sussex or Hampshire or Herefordshire or Hertfordshire or Kent or Lancashire Leicestershire or Lincolnshire or Merseyside or Norfolk or Northamptonshire or Northumberland or Nottinghamshire or Oxfordshire or Rutland or Shropshire or Somerset or Staffordshire or Suffolk or Surrey or "Tyne and Wear" or Warwickshire or Wiltshire or Worcestershire or Aberdeenshire or Angus or Forfarshire or Argyll* or Ayrshire or "County of Ayr" or "County of Banff" or "County of Berwick" or "County of Bute" or "County of Clackmannan" or "East Lothian" or "Ross and Cromarty" or "West Lothian" or Banffshire or Berwickshire or Buteshire or Caithness or Clackmannanshire or Dumfriesshire or Dunbartonshire or Fife or Invernessshire or Kincardineshire or Kinrossshire or Kirkcudbrightshire or Lanarkshire or Midlothian or Moray or Nairnshire or Orkney or Peebleshire or Perthshire or Renfrewshire or Roxburghshire or Selkirkshire or Shetland or Stirlingshire or Sutherland or Wigtownshire or "Blaenau Gwent" or "Isle of Anglesey" or "Merthyr Tydfil" or "Neath Port Talbot" or "Rhondda Cynon Taff" or "Vale of Glamorgan" or Bridgend or Caerphilly or Cardiff or Carmarthenshire or Ceredigion or Conwy or Denbighshire or Flintshire or Gwynedd or Wrexham or Antrim or Armagh or Down or Fermanagh or Londonderry or derry or Swansea or Torfaen or Newport or Pembrokeshire or Powys or Monmouthshire or Tyrone).ti,ab,in.
116. 1 or 2 or 3 or 4 or 5 or 6 or 7 or 8 or 115
117. 116 not 9
118. tenant support.ti,ab,kw.
119. perpetrator programme*.ti,ab,kw.
120. (violen* adj3 (sexual or domestic or child or partner* or spous* or couple* or family or interpersonal or date or dating or gender based or coercive or conjugal or victim* or survivor* or perpetrat* or technology-facilitated or wom#n or image based or honor based or obstetric)).ti,ab,kw.
121. (abus* adj3 (sexual or domestic or child or partner* or spous* or family or couple* or gender based or wom#n or wife or wives or victim* or survivor* or perpetrat* or financ* or economic or image-based or technology-facilitated)).ti,ab,kw.
122. (assault* adj3 (sexual or partner* or spous* or victim* or survivor* or perpetrat* or couple*)).ti,ab,kw.
123. 25 or 26 or 27 or 28 or 29 or 30 or 31 or 32 or 33 or 34 or 35 or 36 or 37 or 38 or 39 or 40 or 41 or 42 or 43 or 44 or 45 or 46 or 47 or 48 or 49 or 50 or 51 or 52 or 53 or 54 or 55 or 56 or 57 or 58 or 59 or 60 or 61 or 62 or 63 or 64 or 65 or 66 or 67 or 68 or 69 or 70 or 71 or 72 or 73 or 74 or 75 or 76 or 77 or 78 or 79 or 80 or 81 or 82 or 83 or 84 or 85 or 86 or 87 or 88 or 89 or 90 or 91 or 92 or 93 or 94 or 95 or 96 or 97 or 98 or 99 or 100 or 101 or 102 or 103 or 104 or 105 or 106 or 107 or 118 or 119
124. 10 or 11 or 12 or 13 or 14 or 15 or 16 or 17 or 18 or 19 or 20 or 21 or 22 or 23 or 24 or 108 or 109 or 110 or 111 or 112 or 113 or 114 or 120 or 121 or 122
125. 117 and 123 and 124
126. limit 125 to (english language and humans)

For peer review only

Grey literature example: Social care online

(combined v4:
UK terms [
 - Location:"'england"' including broader terms
 - OR Location:"'wales"' including this term only
 - OR Location:"'scotland"' including this term only
 - OR Location:"'northern ireland"' including this term only
 - OR Location:"'united kingdom"' including this term only
]
AND
service terms - all fields [
 - AllFields:"'specialised service"'
 - OR AllFields:"'specialist service"'
 - OR AllFields:"'support service"'
 - OR AllFields:"'abuse service"'
 - OR AllFields:"'violence service"'
]
AND
violence terms v2 [
 - SubjectTerms:"'domestic violence"' including related terms
 - OR SubjectTerms:"'sexual abuse"' including related terms
 - OR AllFields:"'sexual violence"'
 - OR AllFields:"'domestic abuse"'
 - OR AllFields:"'domestic violence"'
 - OR AllFields:"'sexual abuse"'
]
)

For peer review only

Appendix 3: Preferred Reporting Items for Systematic reviews and Meta-Analyses extension for Scoping Reviews (PRISMA-ScR) Checklist

SECTION	ITEM	PRISMA-ScR CHECKLIST ITEM	REPORTED ON PAGE #
**TITLE**			
Title	1	Identify the report as a scoping review.	1
**ABSTRACT**			
Structured summary	2	Provide a structured summary that includes (as applicable): background, objectives, eligibility criteria, sources of evidence, charting methods, results, and conclusions that relate to the review questions and objectives.	3-4
**INTRODUCTION**			
Rationale	3	Describe the rationale for the review in the context of what is already known. Explain why the review questions/objectives lend themselves to a scoping review approach.	5-6
Objectives	4	Provide an explicit statement of the questions and objectives being addressed with reference to their key elements (e.g., population or participants, concepts, and context) or other relevant key elements used to conceptualize the review questions and/or objectives.	6-7
**METHODS**			
Protocol and registration	5	Indicate whether a review protocol exists; state if and where it can be accessed (e.g., a Web address); and if available, provide registration information, including the registration number.	7
Eligibility criteria	6	Specify characteristics of the sources of evidence used as eligibility criteria (e.g., years considered, language, and publication status), and provide a rationale.	7-8 and Table 1
Information sources*	7	Describe all information sources in the search (e.g., databases with dates of coverage and contact with authors to identify additional sources), as well as the date the most recent search was executed.	9
Search	8	Present the full electronic search strategy for at least 1 database, including any limits used, such that it could be repeated.	Appendix 2
Selection of sources of evidence†	9	State the process for selecting sources of evidence (i.e., screening and eligibility) included in the scoping review.	10
Data charting process‡	10	Describe the methods of charting data from the included sources of evidence (e.g., calibrated forms or forms that have been tested by the team before their use, and whether data charting was done independently or in duplicate) and any processes for obtaining and confirming data from investigators.	10
Data items	11	List and define all variables for which data were sought and any assumptions and simplifications made.	10
Critical appraisal of individual sources of evidence§	12	If done, provide a rationale for conducting a critical appraisal of included sources of evidence; describe the methods used and how this information was used in any data synthesis (if appropriate).	N/A

For peer review only

SECTION	ITEM	PRISMA-ScR CHECKLIST ITEM	REPORTED ON PAGE #
Synthesis of results	13	Describe the methods of handling and summarizing the data that were charted.	10-11
**RESULTS**			
Selection of sources of evidence	14	Give numbers of sources of evidence screened, assessed for eligibility, and included in the review, with reasons for exclusions at each stage, ideally using a flow diagram.	12 and Figure 1
Characteristics of sources of evidence	15	For each source of evidence, present characteristics for which data were charted and provide the citations.	12-16
Critical appraisal within sources of evidence	16	If done, present data on critical appraisal of included sources of evidence (see item 12).	N/A
Results of individual sources of evidence	17	For each included source of evidence, present the relevant data that were charted that relate to the review questions and objectives.	Appendix 4
Synthesis of results	18	Summarize and/or present the charting results as they relate to the review questions and objectives.	17-21
**DISCUSSION**			
Summary of evidence	19	Summarize the main results (including an overview of concepts, themes, and types of evidence available), link to the review questions and objectives, and consider the relevance to key groups.	22-23
Limitations	20	Discuss the limitations of the scoping review process.	24-25
Conclusions	21	Provide a general interpretation of the results with respect to the review questions and objectives, as well as potential implications and/or next steps.	26
**FUNDING**			
Funding	22	Describe sources of funding for the included sources of evidence, as well as sources of funding for the scoping review. Describe the role of the funders of the scoping review.	3-4

JBI = Joanna Briggs Institute; PRISMA-ScR = Preferred Reporting Items for Systematic reviews and Meta-Analyses extension for Scoping Reviews.
* Where *sources of evidence* (see second footnote) are compiled from, such as bibliographic databases, social media platforms, and Web sites.
† A more inclusive/heterogeneous term used to account for the different types of evidence or data sources (e.g., quantitative and/or qualitative research, expert opinion, and policy documents) that may be eligible in a scoping review as opposed to only studies. This is not to be confused with *information sources* (see first footnote).
‡ The frameworks by Arksey and O'Malley (6) and Levac and colleagues (7) and the JBI guidance (4, 5) refer to the process of data extraction in a scoping review as data charting.
§ The process of systematically examining research evidence to assess its validity, results, and relevance before using it to inform a decision. This term is used for items 12 and 19 instead of "risk of bias" (which is more applicable to systematic reviews of interventions) to include and acknowledge the various sources of evidence that may be used in a scoping review (e.g., quantitative and/or qualitative research, expert opinion, and policy document).

From: Tricco AC, Lillie E, Zarin W, O'Brien KK, Colquhoun H, Levac D, et al. PRISMA Extension for Scoping Reviews (PRISMAScR): Checklist and Explanation. Ann Intern Med. 2018;169:467–473. doi: 10.7326/M18-0850.

For peer review only

Appendix 4: Details of included studies

Study	Peer review or grey lit	Study design	Setting/ sector	Type of intervention	Type of abuse*	No. of outcomes
ADVA (2009)[59]	Grey lit.	Service evaluation	Third	*"Repair"*<br>Perpetrator programme<br>• Comprising individual and group work sessions. Primarily cognitive behavioural, focusing on motivation, responsibility, safety and acknowledgment<br>Women's service<br>• Supportive befriending, information about legal and support services, expectation management, group work, telephone contact, feedback on partners' attendance, safety planning and monitoring and awareness raising	DVA	7
Advance (2020)[60]	Grey lit.	Service evaluation	Third	*"Minerva"*<br>Multi-service<br>• Advocacy for access to other community services<br>• One-to-one and group work support on a range of issues including emotional wellbeing, physical and mental health, violence and abuse, problematic substance use<br>• Benefits and debt advice, housing	DVA	1
Barber et al (2000)[61]	Peer review	Pre-post	Public: Learning disability hospital	-<br>Psychological support<br>• Group therapy using a person-centred therapeutic model, which aimed to run interactive and structured educative sessions, supplemented with supportive and non-confrontational discussions	SVA	4

For peer review only

Study	Peer review or grey lit	Study design	Setting/ sector	Type of intervention	Type of abuse*	No. of outcomes
Bloomfield & Dixon (2015)[62]	Grey lit.	Non-randomised comparative	Public: National Probation Service	*"Integrated Domestic Abuse Programme (IDAP) and Community Domestic Violence Programme (CDVP)"*<br>Perpetrator programme<br>• Cognitive behavioural programme, which challenged offenders' attitudes and beliefs to change their behaviour	DVA	2
Bowen (2010)[63-67]	Peer review	Quasi experimental	Public: local probation service	*"Domestic Violence Perpetrator Programme"*<br>Perpetrator programme<br>• Psychoeducational treatment programme in a group setting, focusing on definitions of DV, socialisation, impact of DV on partners and children, empathy, sexual respect and accountability and communication	DVA	9
Brady et al (2021)[68]	Peer review	RCT	Mixed: Third (human rights charity); unclear ('clinical setting')	-<br>Psychological support<br>• Narrative exposure therapy, involving co-construction of traumatic experiences and a therapy plan, to process traumatic memories. Psychoeducation was also delivered to all participants	SVA	5
Burton et al (1998)[69]	Grey lit.	Service evaluation	Third	*"Violence Prevention Programme (VPP)"*<br>Perpetrator programme<br>• Structured group sessions designed to assist men in understanding why they abuse, how they can change it, and constructing respectful relationships with women<br>Multi-service<br>• Telephone advice and support; making contact with, and regular follow-up of, partners of men on VPP; one-to-one support sessions; two weekly support groups;	DVA	2

For peer review only

Study	Peer review or grey lit	Study design	Setting/ sector	Type of intervention	Type of abuse*	No. of outcomes
				advocacy for women users; weekly liaison with the VPP; interagency work, including workshops and training; administration; and training for new volunteers. These also involve safety planning, housing / financial / legal advice and education (understanding domestic violence)		
Callaghan et al (2020)[70, 71]	Grey lit.	Pre-post	Third: DV charities / organisations	*"Make a Change"*<br>Perpetrator programme<br>• Group-based intervention for perpetrators<br>Psychological support<br>• Individual support for (ex)partners	DVA	13
Calvert et al (2015)[72]	Peer review	Pre-post	Public: Tertiary psychotherapy service	-<br>Psychological support<br>• Group cognitive analytic therapy	SVA (CSA)	6
Clarke & Llewelyn (1994)[73]	Peer review	Pre-post	Public: NHS	-<br>Psychological support<br>• Cognitive analytic therapy	SVA (CSA)	4
Clarke & Pearson (2000)[74]	Peer review	Pre-post	Public: NHS	-<br>Psychological support<br>• Cognitive analytic therapy	SVA (CSA)	4
Cook et al (1991)[75]	Peer review	Pre-post	Public	-<br>Perpetrator programme<br>• Group treatment for sex offenders	SVA	1
Co-ordinated Action Against Domestic	Grey lit.	Data-set / service evaluation	Third	-<br>Advocacy<br>• 14 IDVA services, with types of support	DVA	11

Study	Peer review or grey lit	Study design	Setting/ sector	Type of intervention	Type of abuse*	No. of outcomes
Abuse (2012)[76, 77]				including safety planning, support with MARAC, liaison and support with police, support with criminal court cases, liaison and support with probation, support with civil orders, support with housing, financial benefits advice and support, support with immigration, health and wellbeing advice and support, and support with children		
Coy and Kelly (2011)[78]	Grey lit.	Service evaluation	Third	-<br>Advocacy<br>• Four IDVAs in different settings (police station, hospital A&E department, a community based DV project, and a women only VAW organisation)	DVA	2
Dheensa & Penny (2019)[79]	Grey lit.	Service evaluation	Third	-<br>Advocacy<br>• Learning disability ISVA	SVA	1
Dobash et al (1999)[80]	Peer review	Non-randomised comparative	Public	*"IDVPP"* and *"CHANGE"*<br>Perpetrator programme<br>• Two perpetrator programmes, both focused on challenging group work	DVA	3
Ellis (2012)[81]	Peer review	Pre-post	Third?	*"The Butterfly Programme"*<br>Psychosocial support<br>• Group therapy	SVA (CSA)	6
Farrell & Buckley (1999)[82]	Peer review	Pre-post	Public	*"The Domestic Violence Unit"*<br>Specialist DV police teams<br>• Police officers dedicated to dealing with DV cases with specialist training.	DVA	5
Ferrari et al (2018)[83]	Peer review	Randomised controlled trial	Third	*"PATH"*<br>Advocacy<br>• Specialist psychological advocates providing	DVA	6

For peer review only

Study	Peer review or grey lit	Study design	Setting/ sector	Type of intervention	Type of abuse*	No. of outcomes
				1:1 support on top of regular advocacy provision		
Friendship et al. (2003)[84]	Peer review	Pre-post with retrospectively selected comparison group	Public: Prisons	*"SOTP"*<br>Perpetrator programme<br>• Two programmes based on the cognitive behavioural model, using group sessions	DVA	3
Garry & Munro (2020)[85-87]	Grey lit.	Service evaluation	Third	*"The Rape and Sexual Violence Project (RSVP) adult counselling service"*<br>Psychological support<br>• Face-to-face counselling. Clients can also access RSVPs other support services	SVA	1
Geoghegan-Fittall et al (2020)[88]	Grey lit.	Service evaluation	Third	*"The Croydon Drive Project"*<br>Perpetrator programme<br>• Direct and indirect interventions and activity disruption. Interventions may be singular or concurrent	DVA	3
Gilchrist et al (2021)[89, 90]	Peer review	Randomised controlled trial	Mixed: NHS and voluntary sector	*"ADVANCE"*<br>Perpetrator programme<br>• Manualised evidence-informed tailored intervention focusing on developing healthy relationships	DVA	14
Goosey et al (2017)[91]	Peer review	Randomised controlled trial	Mixed: community safety team, police, drug and alcohol service, Thames Valley Probation, Youth Offending	-<br>Multi-service<br>• Integrated cased management using the Domestic Abuse Service Coordination model, including support for victims, counselling for perpetrators, and follow up	DVA	1

For peer review only

Study	Peer review or grey lit	Study design	Setting/ sector	Type of intervention	Type of abuse*	No. of outcomes
			Service, Domestic Abuse Perpetrator Service, Berkshire Women's Aid, Bracknell Forest Homes	police visits		
Halliwell et al (2019)[92]	Peer review	Non-randomised comparative	Mixed: Public and Third	- Advocacy<br>• A hospital based IDVA who provided support and advice, risk assessment and safety planning, referral, partnership work and training hospital staff	DVA	8
Hester et al (2019)[93-96]	Grey lit	Service evaluation using RCT design	Third	*"Drive"*<br>Perpetrator programme<br>• Intensive case management and multi-agency response. Uses disruption, support and behaviour change interventions alongside protective work<br>Advocacy<br>• One-to-one IDVA support for the associated victims-survivors	DVA	10
Hester & Westmarland (2005)[97]	Grey lit.	Service evaluation	Mixed: Third, public	- Multiple / multi-service<br>• 27 domestic violence projects split in to seven packages (criminal and civil justice, protection and prevention, Black and other ethnic minorities, health, multi-service, education and rural work). A sub selection of the 27 that report relevant outcomes and use the included methodology were extracted, including: Bradford Staying Put, Hammersmith and Fulham Standing Together, Northampton Sunflower Centre,	DVA	17

For peer review only

Study	Peer review or grey lit	Study design	Setting/ sector	Type of intervention	Type of abuse*	No. of outcomes
				Cheshire and Taunton – use of photographic evidence, Croydon Domestic Violence Advocate Service, Northampton Sunflower Centre		
Howarth et al (2009)[98, 99]	Grey lit	Service evaluation	Third	-<br>Advocacy<br>• Seven IDVA services in a range of rurality's, sizes and age of service	DVA	13
Howarth and Robinson (2016)[100]	Peer review	Pre-post	Unclear	-<br>Advocacy<br>• Seven IDVA services. Support provided included safety planning, housing and accommodation, child-related support, access to justice systems, health and wellbeing, and welfare and immigration support	DVA	4
Karatzias et al (2016)[101]	Peer review	Pre-post	Public: NHS	*"Trauma Recovery and Empowerment Model"*<br>Psychological support<br>• Group psychotherapy using cognitive behavioural and skills training techniques	DVA<br>SVA<br>(CSA)	5
Kelley et al (2022)[102]	Peer review	Non-randomised comparative	Private: Privately owned prison	*"Principle-based correctional counselling"*<br>Perpetrator programme<br>• Classes including modules on building rapport, exploration of reality, exploration of thought, exploration of feelings/behaviour.<br>• All participants also participated in the required CBT based Sexual offender Treatment programme	SVA	6
Kelly et al	Grey lit.	Service	Public	*"Domestic Violence Matters"*	DVA	5

For peer review only

Study	Peer review or grey lit	Study design	Setting/ sector	Type of intervention	Type of abuse*	No. of outcomes
(1999)[103]		evaluation		Specialist domestic violence police teams<br>• Five support workers who worked alongside the police to support women where an arrest of a perpetrator occurred.		
Kelly & Westmarland (2015)[104]	Grey lit.	Service evaluation	Third	*"DVPP"*<br>Perpetrator programme<br>• Twelve Respect accredited DVPPs. All have manuals and involved group work.<br>Unclear – Women's support service<br>• Each perpetrator programme had an integrated women's support service, however no further details are provided.	DVA	7
Koppensteiner et al (2019)[105]	Grey lit.	Randomised controlled trial	Public: Police	*"Project 360"*<br>Specialist domestic violence police teams<br>• Engagement workers who contact victims and offer further assistance, including information on legal options, referrals to support services, and safety planning	DVA	1
Lindsay et al (2011)[106]	Peer review	Pre-post	Unclear	-<br>Perpetrator programme<br>• A sex offender treatment programme, including modules on disclosure, pathways into offending, cognitive distortions, childhood abuse, victim awareness, relapse prevention, and adaptive attachment	SVA	2
Lindsay et al. (1998)[107]	Peer review	Pre-post	Public: Psychology / psychiatry department	-<br>Perpetrator programme<br>• Group sessions focused on sex offending and cognitive change, including taking responsibility, victim awareness and behaviours consistent with offending	SVA	1

For peer review only

Study	Peer review or grey lit	Study design	Setting/ sector	Type of intervention	Type of abuse*	No. of outcomes
Lowe et al (2017)[108]	Peer review	Pre-post	Third	-<br>Psychological support<br>• Group therapy. Sessions used a person centred, psychoeducational approach with elements of cognitive behavioural therapy	SVA (CSA)	3
McConnell et al (2017)[109, 110]	Peer review	Pre-post	Third	*"Caring Dads Safer Children"*<br>Perpetrator programme<br>• Includes seven treatment targets: anger/hostility; family cohesion/ coparenting/domestic violence; perceptions of the child as a problem; use of corporal punishment and other aversive behaviours; positive and involved parent-child relationship; self-centeredness; and misuse of substances	DVA	4
McCracken & Deave (2012)[111]	Grey lit.	Service evaluation	Third	*"Caring Dads Cymru"*<br>Perpetrator programme<br>• Group work programme based on cognitive behavioural therapy and motivational models of intervention	DVA	4
Michie & Lindsay (2012)[112]	Peer review	Non-randomised comparative	Public: NHS	-<br>Perpetrator programme<br>• Cognitive behavioural therapy with an empathy component	SVA	1
Morgan et al (2019)[113, 114]	Peer review	Pre-post	Third	*"RADAR/ADAPT"*<br>Perpetrator programme<br>• A multiagency initiative that provides a tiered needs-led approach. It provides a programme for perpetrators and support for victims	DVA	4
Morrison &	Peer review	Pre-post	Public: Local	-	SVA	1

For peer review only

Study	Peer review or grey lit	Study design	Setting/ sector	Type of intervention	Type of abuse*	No. of outcomes
Treliving (2002)[115]			psychotherapy service	Psychological support<br>• Group therapy aiming to provide men with a safe space to explore their difficulties and the impact of childhood sexual abuse	(CSA)	
Murphy et al (2007)[116]	Peer review	Pre-post	Public: Intellectual disability services	-<br>Perpetrator programme<br>• Group cognitive behavioural therapy for sexually abusive men	SVA	4
Oasis (2016)[117, 118]	Grey lit	Service evaluation	Third	-<br>Multi-service<br>• Counselling (6 sessions plus more if required)<br>• Refuge: a 12-bed refuge providing temporary emergency accommodation. Staff provide emotional and practical support	DVA	3
Ormston et al (2016)[119]	Grey lit.	Service evaluation	Third	*"The Caledonian System"*<br>Perpetrator programme<br>• Perpetrator programme covering lifelong change, responsibility for and to self, relationships, sexual respect, men and women, and children and fathering.<br>Multi-service<br>• Women's service including safety planning, information, advice, and emotional support to (ex)partners	DVA	6
Payne et al (2007)[120]	Peer review	Pre-post	Public (assumed as authors are clinical psychologists)	-<br>Psychological support<br>• Client centred group therapy	DVA<br>SVA<br>(CSA)	3
Peckham et al (2007)[121]	Peer review	Pre-post	Public: local community intellectual disability service	-<br>Psychological support<br>• Survivors group, involving educating clients and carers about sexual abuse, and weekly	SVA	6

For peer review only

Study	Peer review or grey lit	Study design	Setting/ sector	Type of intervention	Type of abuse*	No. of outcomes
				sessions using drawings, videos, dolls and pictures		
Robinson (2003)[122]	Grey lit.	Service evaluation	Mixed: Public (police, criminal justice) and third sector	*"Women's Safety Unit"*<br>Multi-service<br>• Advocacy, specialist counselling, legal services, housing services, refuge provision, target hardening and collecting evidence	DVA<br>SVA	11
Robinson (2004)[123]	Grey lit.	Service evaluation	Mixed: Public (police, probation, health, WSU, social services, housing, health) and third sector	-<br>MARAC<br>• Multiagency meeting which delegates actions to workers. Agencies include police, social services, probation, health, and education (where relevant)	DVA	6
Robinson (2006)[124]	Peer review	Pre-post	Mixed: Police, probation, health, WSU, social services, housing, health, third sector	-<br>MARAC<br>• Monthly multiagency meetings to discuss high risk victims, to share information and take actions to reduce future harm.	DVA	6
Robjant et al (2017)[125]	Peer review	Pre-post	Third	-<br>Psychological support<br>• Narrative exposure therapy, a therapy specifically designed for victims of trauma	SVA	2
Rose et al (2012)[126]	Peer review	Pre-post	Public: Community Learning Disability Services	-<br>Perpetrator programme<br>• Group programme with topics including sex education, emotion recognition, cognitive distortions, motivation for offences, empathy and relapse prevention	SVA	4
Ross et al.	Peer review	Non-	Public: Magistrate's	-	DVA	4

For peer review only

Study	Peer review or grey lit	Study design	Setting/ sector	Type of intervention	Type of abuse*	No. of outcomes
(2022)[127]		randomised comparison	Court	Advocacy<br>• An IDVA service based at a Specialist Domestic Abuse Court		
Ryan et al (2005)[128]	Peer review	Randomised controlled trial	Public: NHS psychology departments	-<br>Psychological support<br>• Individual and group therapy, focused on experiences and consequences of CSA	SVA (CSA)	4
SafeLives[129]	Grey lit.	Dataset / service evaluation	Third	-<br>Refuge<br>• No information	DVA	12
SafeLives[130]	Grey lit.	Dataset / service evaluation	Third	-<br>MARAC<br>• No information	DVA	3
SafeLives[131, 132]	Grey lit.	Dataset / service evaluation	Third	-<br>Sexual violence services<br>• No information	SVA	19
SafeLives[133]	Grey lit.	Dataset / service evaluation	Third	-<br>Health services<br>• No information	DVA	12
Safelives[134]	Grey lit.	Dataset / service evaluation	Third	-<br>Helpline<br>• No information	DVA	11
SafeLives[135 -139]	Grey lit.	Dataset / service evaluation	Third	-<br>IDVA<br>• No information	DVA	12
SafeLives[140 -144]	Grey lit.	Dataset / service evaluation	Third	-<br>Outreach<br>• No information	DVA	12

For peer review only

Study	Peer review or grey lit	Study design	Setting/ sector	Type of intervention	Type of abuse*	No. of outcomes
Sharpe et al (2001)[145]	Peer review	Pre-post	Public: Psychological therapies service	*"The Male Childhood Sexual Abuse Group"*<br>Psychological support<br>• Group therapy, no further information	SVA (CSA)	2
Skyner & Waters (1999)[146]	Peer review	NR	Mixed: Public (probation service) and third sector (NSPCC)	*"The Domestic Violence Prevention Programme"*<br>Perpetrator programme<br>• A cognitive behavioural programme with a variety of learning styles. A parallel support package is provided to (ex)partners and their children throughout	DVA	4
Smith et al (1995)[147]	Peer review	Pre-post	Public: Specialised therapy unit	*"Breakfree"*<br>Psychological support<br>• Individual therapy, daytime drop in facility, telephone contact, out-of-hours paging service, limited befriending service and a 'time out' facility where a client can stay in a safe environment for 1-2 nights	SVA (CSA)	3
Smith et al (2015)[148]	Peer review	Pre-post	Third	*"Domestic Abuse Recovering Together (DART)"*<br>Psychological support<br>• Mother and child sessions, designed to strengthen their relationship and support recovery. Followed by separate peer group sessions	DVA	3
Stanley et al (2011)[149]	Grey lit.	Service evaluation	Mixed: Third and Public (NHS, City Council)	*"Strength to change"*<br>Perpetrator programme<br>• Individual and group sessions over 40 weeks. Advocacy and advice are offered where appropriate. Sessions include mindfulness techniques, cognitive approaches, counselling, behavioural techniques.<br>• A women's service is available for	DVA	4

For peer review only

Study	Peer review or grey lit	Study design	Setting/ sector	Type of intervention	Type of abuse*	No. of outcomes
				(ex)partners offering practical and emotional support		
Stanley et al (2021)[150]	Grey lit	Service evaluation	Third	*"VOICES"*<br>Multi-service<br>• Trauma informed approach. Practitioners were given a new assessment framework, training and planning tools. No information on the specific types of activities or interventions is reported	DVA	6
Stanley et al (2021)[151]	Grey lit	Service evaluation	Third	*"SafeLives Co-Designed Pilots (SLCDPs)"*<br>Multi-service<br>• A suite of interventions for survivors, their children and perpetrators, using a whole family approach. This included community IDVA support, complex needs IDVA support, step down and recovery groups, children and young peoples work and 'Engage', which worked with the whole family	DVA	5
Strang et al (2017)[152]	Peer review	Randomised controlled trial	Mixed: Public (police), third sector	*"CARA"*<br>Perpetrator programme<br>• A two-day offender workshop, focusing on raising awareness, and moving offenders from denial and minimisation to acceptance and responsibility of harm, and conflict resolution	DVA	3
Taylor-Dunn & Erol (2019)[46, 153]	Grey lit	Service evaluation	Third	-<br>Advocacy<br>• Intensive one-to-one IDVA support within an immediate to medium term time-frame, implementing individual support plans and risk assessments to reduce risk and encourage recovery support	DVA	5

For peer review only

Study	Peer review or grey lit	Study design	Setting/ sector	Type of intervention	Type of abuse*	No. of outcomes
The Institute for Public Safety, Crime & Justice, University of Northampton (2019)[154]	Grey lit.	Service evaluation	Third	*"SHE"*<br>Psychological support<br>• A 'minimum support service (MSS)' with regular telephone check-ins, with an emphasis on safety and wellbeing, and an 'integrated support service (ISS+)' offering a face-to-face person-centred programme<br>Perpetrator programme<br>• 'Evolve', a programme domestic violence perpetrator programme working towards Respect accreditation	DVA	5
Trevillon et al (2014)[155]	Peer review	Non-randomised comparative	Public: Community mental health team	-<br>Advocacy<br>• Domestic violence training for clinicians, domestic violence manual for clinicians, mental illness training for domestic violence advisors, direct referral pathway to domestic violence advocacy, provision of a domestic violence advocacy service, comprising emotional and practical support, domestic violence education, support groups, safety planning and legal/housing support	DVA	11
Vallely et al (2005)[156]	Grey lit.	Service evaluation	Mixed: Public (Crown Prosecution Service, Police, Magistrates' Court, Probation service, local authorities) and Third sector	-<br>Specialist domestic violence court<br>• The service included a dedicated sitting for pre-trial matters and sentencing, allocated weekly; specialist magistrates with domestic violence training, specially trained CPS lawyers, and an independent advocacy service	DVA	5
Webster (2015)[157]	Grey lit.	Service evaluation	Third	*"Kent and Medway IDVA"*<br>Advocacy	DVA	2

For peer review only

Study	Peer review or grey lit	Study design	Setting/ sector	Type of intervention	Type of abuse*	No. of outcomes
				• Up to 21 IDVAs working with domestic abuse victims. No further information.		
Westmarland & Alderson (2013)[158]	Peer review	Pre-post	Third	-<br>Psychological support<br>• Face-to-face counselling, following a woman-centred, empowerment model, with elements of cognitive behavioural therapy, goal setting and rational emotive behaviour therapy	SVA	1
Williamson & Boyle (2012)[159]	Peer review	Pre-post	Public: NHS	*"IDVA Advocacy Intervention Programme"*<br>Advocacy<br>• No details reported	DVA	2
Williamson & Hester (2009)[160]	Grey lit.	Service evaluation	Third	*"South Tyneside Domestic Abuse Perpetrator Programme"*<br>Perpetrator programme<br>• A 'pro-feminist broadly cognitive behavioural model combined with gender analysis' approach. Group and one-to-one sessions.	DVA	2
Women's Aid (2022)[161-166]	Grey lit.	Service evaluation	Third	*"No Woman Turned Away"*<br>Housing (specialist caseworkers)<br>• Caseworkers provide telephone and email support to women looking for a refuge space	DVA	1
Women's Resource Centre (2011b)[167]	Grey lit.	Service evaluation	Third	*"Women and Girls Network"*<br>Psychological support<br>Counselling using trauma therapy, cognitive behavioural therapy, and integrated person-centred and feminist counselling practice. The organisation also offers a telephone helpline, body therapies, group programmes and specialist support projects	DVA	1
Women's Resource	Grey lit.	Service evaluation	Third	*"Ashiana"*<br>Multi-service	DVA	11

For peer review only

Study	Peer review or grey lit	Study design	Setting/ sector	Type of intervention	Type of abuse*	No. of outcomes
Centre (2011a)[168]				• Refuge: Safe accommodation across three schemes for South Asian, Turkish and Iranian women. Residents can stay for up to 1 year.<br>• Outreach: To provide support and reduce repeat victimisation. Ashiana also runs a DV support group for women in the wider community, as well as an end of therapy support group.		

For peer review only

For peer review only

Appendix 5: Outcome measures by domain and subdomain

Category	Domain	Subdomain	Measure	N
**Health**	**Mental health**	**Depression**		**16**
			Beck Depression Inventory	6
			Hospital Anxiety and Depression Scale - Depression	4
			Patient Health Questionnaire	3
			PROMIS depression Short Form	1
			Single item – “Is the client experiencing depression?”	1
			Single item – reduction in feeling depressed	1
		**Anxiety**		**10**
			Hospital Anxiety and Depression Scale - Anxiety	4
			Generalized Anxiety Disorder Scale	3
			PROMIS Anxiety Short Form	1
			Spiel State Anxiety	1
			Single item – “Is the client experiencing anxiety?”	1
		**Trauma**		**12**
			Primary care PTSD screen	2
			Impact of Event Scale	2
			PTSD Checklist for DSM-5 (PCL-5)	1
			PTSD Symptom Scale	1
			Posttraumatic Diagnostic Scale	1
			Post-traumatic Stress Disorder Scale	1
			Changes in Outlook Questionnaire	1
			The Clinician Administered PTSD Scale	1
			PTSD Checklist (PCL-C)	1
			Single item – “I have “flashbacks” about what happened”	1
		**General mental health**		**4**
			General Health Questionnaire (GHQ-28)	2
			Single item - "Current mental health rating"	1

For peer review only

Category	Domain	Subdomain	Measure	N
			No information – reported outcome is "Improved mental health"	1
		**Psychological symptoms**		**19**
			The Clinical Outcomes in Routine Evaluation - Outcome Measure (CORE-OM)	5
			The Symptom Checklist 90 revised (SCL-90R)	4
			Brief Symptom Inventory	2
			Jehu Beliefs Inventory	2
			The Shutdown Dissociation Scale (Shu-Dis)	1
			Dissociative Experiences Scale (DES)	1
			Belief Inventory	1
			Social Activity and Distress Scale	1
			Delusions, Symptoms and States Inventory	1
			Single item – “Is the client experiencing emotional instability?”	1
		**Anger**		**2**
			The Novaco Anger Scale	1
			PROMIS Anger Short Form	1
		**Understanding feelings**[$]		2
			Three Principles Inventory[$]	1
			Single item - "Understanding feelings better"[$]	1
		**Self-Harm / Suicide**[$]		**5**
			Single item – reduction in experiencing suicidal thoughts or feelings[$]	1
			Single item – reduction in self harm[$]	1
			Single item – “Is the client experiencing self-harm?” [$]	1
			Single item – “Is the client experiencing suicidal thoughts?” [$]	1
			Single item – “Is the client experiencing suicidal behaviours?” [$]	1
		**Healthcare utilisation / resource use**[$]		**5**
			Number / percent of participants using healthcare resources (e.g., A&E, GP, psychiatrist)[$]	3
			Number / percent of participants using community health and social service resources (e.g., health visitor, drug worker) [$]	1
			Days of admission[$]	1
		**Mental wellbeing**[$]		**4**

For peer review only

Category	Domain	Subdomain	Measure	N
			Short Warwick–Edinburgh Mental Wellbeing Scale (SWEMWBS) [$]	4<br>2
		**Self-care**[$]	Single item - "Is the client experiencing trouble sleeping?" [$]	1
			Single item - "Is the client experiencing problems with eating?" [$]	1
				**3**
		**Quality of life**[$]	Short Form 12 (SF12) [$]	2
			Manchester Short Assessment of Quality of Life[$]	1
		**Health, mental health and wellbeing**[$]		**1**
			Taking Back Control Tool[$]	1
	**Physical health**	**Self-reported physical health**		**8**
			EQ-5D	3
			Visual analogue scale for physical health	2
			Single item – "Current physical health (poor/fair/good/very good/excellent)"	1
			No information – reported outcome is "Improved physical health (DV specific)"	1
			No information – reported outcome is "Improved physical health (not DV specific)"	1
		**Alcohol and drug use**		**5**
			Alcohol Use Disorders Identification Test (AUDIT) [$]	1
			Single item – "Current drug use (excessive/high/moderate/low/none)" [$]	1
			Single item – "Current alcohol use (excessive/high/moderate/low/none)" [$]	1
			Single item – "Alcohol problem (yes/no)" [$]	1
			Single item – "Drug problem (yes/no)" [$]	1
				**5**
		**Healthcare utilisation / resource use**[$]	Number / percent of participants using healthcare resources (e.g., A&E, GP, psychiatrist)[$]	3
			Number / percent of participants using community health and social service resources (e.g., health visitor, drug worker) [$]	1
			Days of admission[$]	1
		**Quality of life**[$]		**3**
			Short Form 12 (SF12) [$]	2

For peer review only

Category	Domain	Subdomain	Measure	N
			Manchester Short Assessment of Quality of Life[$]	1
		**Health, mental health and wellbeing[$]**		**1**
			Taking Back Control Tool[$]	1
	**Wellbeing**	**Wellbeing**		**7**
			Outcome Star	1
			Adult wellbeing scale	1
			Benefits of Therapy scale	1
			Personal Outcomes and Wellbeing Record (POWeR)	1
			Victim survey (perceived wellbeing - safety and wellbeing)	1
			Wellbeing survey (9 domains)	1
			CORE monitoring form	1
			Perceived Severity of Problems/Concerns Questionnaire	1
				**5**
			Social Inclusion Scale[$]	1
			Inventory of interpersonal problems 32 (IIP-32) [$]	1
		**Social ties/ relationships**	Single item - "Current social and community support (no social contact / negative social contacts / some positive contacts / majority positive contacts / all positive)" [$]	1
			No information – reported outcome is "Improved relationship with family"[$]	1
			No information – reported outcome is "Improved social support"[$]	1
				**2**
		**Understanding feelings**	Three Principles Inventory[$]	1
			Single item - "Understanding feelings better"[$]	1
				**4**
		**Mental wellbeing[$]**		
			Short Warwick–Edinburgh Mental Wellbeing Scale (SWEMWBS) [$]	4
		**Self-care[$]**		**2**

For peer review only

Category	Domain	Subdomain	Measure	N
			Single item - "Is the client experiencing trouble sleeping?" [$]	1
			Single item - "Is the client experiencing problems with eating?" [$]	1
				**3**
		**Quality of life**[$]	Short Form 12 (SF12) [$]	2
			Manchester Short Assessment of Quality of Life[$]	1
		**Health, mental health and wellbeing**[$]		**1**
			Taking Back Control Tool[$]	1
**Behavioural**	**Behavioural**	**Challenging behaviour**		**1**
			Challenging Behaviour Inventory	1
		**Self-Harm / Suicide**[$]		**5**
			Single item – reduction in experiencing suicidal thoughts or feelings[$]	1
			Single item – reduction in self harm[$]	1
			Single item – "Is the client experiencing self-harm?" [$]	1
			Single item – "Is the client experiencing suicidal thoughts?" [$]	1
			Single item – "Is the client experiencing suicidal behaviours?" [$]	1
		**Alcohol and drug use**[$]		**5**
			Alcohol Use Disorders Identification Test (AUDIT) [$]	1
			Single item – "Current drug use (excessive/high/moderate/low/none)" [$]	1
			Single item – "Current alcohol use (excessive/high/moderate/low/none)" [$]	1
			Single item – "Alcohol problem (yes/no)" [$]	1
			Single item – "Drug problem (yes/no)" [$]	1
**Empowerment**	**Empowerment**	**Self-esteem**		**12**
			Rosenberg's self-esteem scale	7
			Culture Free Self Esteem Inventory	2
			The Self Concept Questionnaire	1
			Self-esteem Assessment Tool	1
			No information – reported outcome is "Improved self-esteem"	1
		**Coping and resilience**		**3**
			Resilience Research Centre Adult Resilience Measure (RRC-ARM)	1

For peer review only

Category	Domain	Subdomain	Measure	N
			Cope and Recover Questionnaire	1
			No information – reported outcome is "Improved coping"	1
		**Locus of control**		**1**
			Review of Personal Effectiveness with Locus of Control	
		**Empowerment**		**5**
			The Empowerment Star	2
			Measure of Victim Empowerment Related to Safety Scale (MOVERS)	1
			Coping and confidence outcome measure	2
		**Assertiveness**		**1**
			Assertiveness Questionnaire	1
		**Knowledge**		**1**
			Goal Attainment Scale	1
		**Social ties/ relationships**[$]		**5**
			Social Inclusion Scale[$]	1
			Inventory of interpersonal problems 32 (IIP-32) [$]	1
			Single item - "Current social and community support (no social contact / negative social contacts / some positive contacts / majority positive contacts / all positive)" [$]	1
			No information – reported outcome is "Improved relationship with family"[$]	1
			No information – reported outcome is "Improved social support"[$]	1
		**Understanding feelings**[$]		**2**
			Three Principles Inventory[$]	1
			Single item - "Understanding feelings better"[$]	1
				2
			No information – reported outcome "Independence"	1
		**Independence**		
			No information – reported outcome "Increased likelihood of employment "	1
		**Awareness of support**		1

Category	Domain	Subdomain	Measure	N
			Number of criminal justice contacts	1
**Socio-economic circumstances**	**Socio-economic circumstances**	**Employment/ education**		**5**
			Single item - "Employment status (FT employed/PT employed/unemployed/caring for children/family)"	1
			Single item - "Employment status (paid employment/voluntary work/education/retired)"	1
			Single item - "Returned to/begun employment or education"	1
			Single item - "Education, employment and training (none / occasional / some sustained / sustained / sustained and fulfilling)"	1
			No information – reported outcome "Increased likelihood of employment"[$]	1
		**Finances**		**3**
			No information – reported outcome "Financial savings"	1
			Single item - "Perception of income (high/comfortable/managing with treats/managing with occasional treats/essentials only/struggling)"	1
			Single item - "Current financial situation (significant financial problems/being reliant on others/unable to pay for essentials/managing essentials but nothing left over/comfortably managing and no financial concerns)"	1
		**Housing**		**4**
			No information – reported outcome "Access to housing"	1
			Single item – "Current housing (homeless / transitory / unsuitable / temporary / secure)"	1
			Single item – "Number of women accommodated in suitable refuge spaces"	1
			Number / percent of participants using supported accommodation resources (e.g., weeks spent in refuge / staffed accommodation / B&B, boarding house or hotel / homeless and living with friends or family)	1
				**1**
				5
		**Social ties/ relationships**[$]	Social Inclusion Scale[$]	1
			Inventory of interpersonal problems 32 (IIP-32) [$]	1
			Single item - "Current social and community support (no social contact /	1

For peer review only

Category	Domain	Subdomain	Measure	N
			negative social contacts / some positive contacts / majority positive contacts / all positive)" [$]	
			No information – reported outcome is "Improved relationship with family"[$]	1
			No information – reported outcome is "Improved social support"[$]	1
**Changing perpetrator behaviour**	**Changing perpetrator behaviour**	**Anger**		**1**
			The Novaco Anger Scale	1
		**Perpetrator needs**		**1**
			Distribution of needs	1
		**Attitudes to women / violence**		**20**
			Inventory of Beliefs About Wife Beating	1
			Questionnaire on Attitudes Consistent with Sexual Offending (QACSO)	3
			Socio-Sexual Knowledge and Attitudes Assessment – Revised (SSKAAT-R)	1
			The Intimate Partner Violence Responsibility Attribution Scale (IPVRAS)	1
			URICA-DV Readiness to change	1
			Propensity for Abusiveness Scale (PAS)	2
			Paulhus Deception Scale (PDS)	1
			Interpersonal Reactivity Index (IRI)	1
			Sexual Attitudes and Knowledge Scale	1
			Victim Empathy Scale-Adapted	1
			Sexual Offenders Self Appraisal Scale	1
			Treatment motivation Questionnaire	1
			Interpersonal Dependency Inventory	1
			Attitudes Toward Exhibitionism Questionnaire	1
			No information - "Attitudes to the offence"	1
			No information - "Attitudes towards women"	1
			No information - "Acceptance of responsibility"	1
		**Desirable responding**		**3**
			Balanced Inventory of Desirable Reporting	3
		**Locus of control / self-control**		**6**
			Multidimensional Locus of Control Scale	1
			Rotter's Locus of Control Scale	1
			Brief self-control scale (BSCS)	1
			Low self-control scale (LSCS)	1

For peer review only

Category	Domain	Subdomain	Measure	N
			Levenson Locus of Control	1
			The Nowicki-Strickland Locus of Control Scale	1
		**Reasons for violence**		**2**
			Number of reasons for violence given	1
			Number of participants reporting each of 7 potential reasons for abuse	1
**Victim-perpetrator relationship**	**Victim-perpetrator relationship**	**Victim-perpetrator relationship**		**12**
			The Communication Patterns Questionnaire-Short Form (CPQ-SF)	1
			Respectful communication	1
			Awareness of self and others	1
			Attitude to partner (self-partner correlation)	1
			Single item - "Relationship status"	1
			Single item - "Hopes for the relationship"	1
			Single item - "Living circumstances (with perpetrator / not living together / intermittently living together)	6
**Parenting**	**Parenting**	**Parenting**		**7**
			Parent Empowerment and Efficacy Measure (PEEM)	1
			Camberwell Assessment of Need for Mothers (Short version)	1
			Parenting Stress Index	2
			Parental Acceptance-Rejection Questionnaire (PARQ)	2
			The Parental Locus of Control Scale	1
**DSVA**	**Experience of DSVA**	**Impact of abuse**		**2**
			Number of participants experiencing each of 18 potential impacts of abuse	1
			Mean number of impacts of abuse	
		**Indicators of ongoing abuse**		**5**
			Additional MARACs/Reappearance at MARAC	3
			Other evidence of abuse (e.g. harassing texts)	1
			Cases reopened	1
		**Perceived safety**		**7**
			Safety Behaviour Checklist	1
			Single item - "Partner fearful (1= never to 4=often)"	1

For peer review only

Category	Domain	Subdomain	Measure	N
			Single item - "Feeling of safety (much safer / somewhat safer / no change / less safe)"	1
			Safety (6 items)	2
			Single item - "How safe do you feel? (not safe / a little unsafe / somewhat safe / very safe)	1
			No information - reported outcome is "increased safety"	1
			No information - reported outcome is "avoidance of forced marriage"	1
		**Revictimization**		**4**
			Average number of repeat victims per month	1
			Number / percent of repeat victims	1
			Number / percent of women reporting repeat victimisation (for IPA)	1
			Number / percent of women disclosing further incidents of violence to IDVA	1
		**Presence of abuse$**		**46**
			Severity of Abuse Grid - presence: Number / percent of people responding yes to the presence of abuse (reported for physical abuse, sexual abuse, harassment and stalking and jealous and controlling behaviour) $	10*
			Severity of Abuse Grid - cessation: Number / percent of people reporting no abuse$	11*
			Severity of Abuse Grid - reduction in presence: Number / percent of people reporting a reduction of abuse (reported for physical abuse, sexual abuse, harassment and stalking and jealous and controlling behaviour) $	1
			Severity of Abuse Grid - multiple types: Number / percent of people reporting multiple types of abuse$	7*
			Number / percent of participants experience concurrent domestic abuse as well as sexual abuse$	1
			Number / percent of participants who have been subjected to adult sex offences$	1
			Number / percent of men abusing each month$	1
			Space for Action Survey (% responding yes to 12 indicators) $	1
			Physical and Sexual Violence Survey (% responding yes to 7 indicators) $	1
			Harassment and other abusive acts survey (% responding yes to 6 indicators) $	1
			Fathering survey / using children against a partner (% responding yes to 5 indicators) $	2

For peer review only

Category	Domain	Subdomain	Measure	N
			Social media perpetration / victimisation survey$	1
			Composite Abuse Scale$	3
			Abusive Behaviour Inventory$	2
			Controlling Behaviour Inventory$	3
		**Number of abusive incidents or behaviours$**		**4**
			Mean number of abusive incidents per man$	1
			Mean number of abusive behaviours reported by participants$	1
			Number / percent of participants experiencing between 0 and 7 behaviours (reported for emotional, physical and sexual behaviours) $	1
			IMPACT monitoring toolkit (reported for emotional, physical, and sexual behaviours) $	1
		**Severity of abuse$**		**29**
			Severity of Abuse Grid - severity: Number / percent of participants reporting high / moderate / standard severity (reported for physical abuse, sexual abuse, harassment and stalking and jealous and controlling behaviour) $	8*,±
			Severity of Abuse Grid - reduction in severity: Number / percent of participants reporting a reduction of high / moderate / standard / unknown severity (reported for physical abuse, sexual abuse, harassment and stalking and jealous and controlling behaviour) $	1
			Severity of Abuse Grid – escalation in severity: Number / percent of participants reporting escalation in severity of abuse$	2
			Severity of Abuse Grid - severity: Number / percent of participants reporting any form of severe abuse$	2
			Severity of Abuse Grid - high severity: Number / percent of participants reporting severe abuse (reported for physical abuse, sexual abuse, harassment and stalking and jealous and controlling behaviour) $	1
			Severity of Abuse Grid - severity: Number / percent of participants reporting multiple types of abuse that are high severity$	6*
			Severity of Abuse Grid - severity: Number / percent of participants reporting at least one type of abuse that is high severity$	6*
			Cambridge Crime Harm Index$	3
		**Frequency of abuse$**		**14**

For peer review only

Category	Domain	Subdomain	Measure	N
			Severity of Abuse Grid - frequency: Number / percent of participants reporting high / moderate / standard / unknown frequency (reported for physical abuse, sexual abuse, harassment and stalking and jealous and controlling behaviour) $	5*
			Severity of Abuse Grid - frequency escalation: Number / percent of participants reporting an escalation in frequency$	6*
			Frequency of abusive behaviours (scale 1 = never -3 = often) $	1
			Frequency rating of past violence (none (1) -> frequent (10) $	1
			Frequency rating of current violence (none (1) -> frequent (10) $	1
		**Duration of abuse$**		**1**
			Number / percent of victims with incidents recorded over a period longer than 12 months$	1
		**Severity and frequency of abuse$**		**13**
			Severity of Abuse Grid - severity and frequency escalation: Number / percent of participants reporting any escalation in severity or frequency of abuse$	7*
			Severity of Abuse Grid - severity and frequency escalation: Number / percent of participants reporting at least one form of abuse that is high severity and escalating in frequency or severity$	6*
		**Risk$**		**18**
			Spousal Assault Risk Assessment$	2
			DASH risk (high/med/standard) $	2
			Drive-DASH (mean score) $	1
			DASH risk (number with 1-14 assessments who had a decrease in score) $	1
			Change in risk in perpetrators (measurement tool unclear) $	1
			Weighted change in risk in perpetrators (measurement tool unclear) $	1
			IDVA perception of change in risk (do not know / increased / limited or no reduction / moderate reduction / significant reduction) $	1
			IDVA rated reduction in risk (percentage reporting 'some reduction in risk') $	1
			IDVA perception of sustainability of change in risk (Do not know / short term / medium term / long term / risk is permanently eliminated) $	1
			Men's perception of risk of using violence again (never/not sure/possibly) $	1

For peer review only

Category	Domain	Subdomain	Measure	N
			Risk factors - Perpetrators' crimogenic behaviour and aggravating problems (frequency and percent of 7 factors) $	1
			Risk factors - Specific features of abuse (frequency and percent of 10 factors) $	1
			Risk factors - Victims' appraisals of threat (frequency and percent of 3 factors) $	1
			Risk factors - Factors relating to children (frequency and percent of 3 factors) $	1
			Risk factors - Factors relating to victim (frequency and percent of 4 factors) $	1
			Probability of none / standard / moderate / and high abuse (reported for physical abuse / sexual abuse / harassment and stalking / jealous and controlling behaviour) $	1
		**Police incidents$**		**39**
			Average / total number of police incidents$	1
			Average / total number of DV related police incidents$	2
			Average / total number of incidents recorded by the police per month$	1
			Average number of DV complaints per month$	1
			Average number of arrests made per month$	1
			Average number of victims refusing to make a complaint per month$	1
			Average number of mental health related DV complaints per month$	1
			Average number of alcohol related DV complaints per month$	1
			Average number of drug related DV complaints per month$	1
			Average number of concern for children reports per month$	1
			Number / percent of participants that committed DV related police incidents$	1
			Number / percent of participants that committed (unspecified) police incidents$	1
			Number / percent of participants with (unspecified) arrests$	1
			Number / percent of calls to domestic incidents$	1
			Number / percent of repeat calls to domestic incidents$	1
			Number / percent of repeat calls to domestic violence incidents (number with 2 incidents, 3 incidents, 4 incidents… 14 incidents) $	1
			Number / percent of DV incidents where the perpetrator was not present$	1
			Number / percent of DV incidents that were crimed$	1
			Number / percent of DV incidents that led to arrests$	2

For peer review only

Category	Domain	Subdomain	Measure	N
			Number / percent of incidents with an arrest where a statement is given$	1
			Number / percent of incidents with an arrest and a statement is given where it is then retracted$	1
			Number / percent of crimes where perpetrator was charged/summonsed$	1
			Number / percent of police incidents before and after a MARAC$	1
			Number / percent of high / medium / low severity police incidents before and after a MARAC$	1
			Number / percent of women experiencing no further post-MARAC incidents of violence or abuse$	1
			Number / percent of women experiencing no further post-MARAC police complaints or call outs$	2
			Number / percent of women experiencing no further post-MARAC police complaints$	2
			Number / percent of women experiencing no further post-MARAC police call outs$	2
			Number / percent of women experiencing either additional post-MARAC police complaints or call outs$	1
			Number / percent of women experiencing both additional post-MARAC police complaints and call outs$	1
			Percent change in total calls to domestic incidents$	1
			Percent change in total calls to domestic incidents (as proportion of all calls) $	1
			Frequency of police call outs (never / once / 2-5 times / 6-10 times) $	1
			Cost of police incidents$	1
	**Perpetration of DSVA**	**Reoffending**		**20**
			Number / percent with proven reoffending (DV, core violence or any offence)	1
			Number / percent who reoffended (police incidents)	1
			Number / percent who committed further sex offences	2
			Number / percent who reoffended (no further details)	1
			Number / percent with repeat prosecution for violence against partner	1
			Number / percent with reconviction for another sexual offence	1
			Number / percent with reconviction for another violence offence	1
			Number / percent with reconviction for any offence	2

For peer review only

Category	Domain	Subdomain	Measure	N
			Number / percent linked to another crime (suspect or charged)	1
			Number / percent linked to another DVA crime (suspect or charged)	1
			Frequency of re-arrests for DVA	1
			Prevalence of re-arrests for DVA	1
			Number / percent of crimes that that were DVA related	1
			Number of incidents of DV whilst on the programme	1
			Number of incidents of non-DV crime whilst on the programme	1
			Number / percent of partners reporting subsequent violence	1
			Number / percent of partners reporting 'frequent violence' at follow up	1
			Time to reoffend	1
		**Criminal justice outcomes**		**16**
			Average number of people charged per month	1
			Average number of cases proceeding through court per month	1
			Average number of days between arrest and case finalisation	1
			Number / percent of cases discontinued	2
			Number / percent of cases bound over / other / found not guilty or absolute discharge / convicted	1
			Number / percent of victims retracting	1
			Number / percent of trials that went ahead on scheduled date	1
			Number / percent of trials that received a conviction	1
			Number / percent of charges that received a conviction	1
			Number / percent of guilty and not guilty pleas	1
			Number / percent of defendants who had the following sentences: conditional discharge / financial penalty / custody / community punishment order / community rehabilitation order	1
			Number / percent of cases with the following convictions: custodial sentence / non-custodial sentence / conditional discharge	1
			Number / percent of evidence types in CPS files (victim statement / police statement / defendant interview / witness statement / medical statement / case exhibits / 999 tape / forensic)	1
			Improvements specific to the 'CPS Criteria for Success'	1

For peer review only

Category	Domain	Subdomain	Measure	N
			Average monetary penalties (fines and compensation)	1
		**Presence of abuse**$		**46**
			Severity of Abuse Grid - presence: Number / percent of people responding yes to the presence of abuse (reported for physical abuse, sexual abuse, harassment and stalking and jealous and controlling behaviour) $	10*
			Severity of Abuse Grid - cessation: Number / percent of people reporting no abuse$	11*
			Severity of Abuse Grid - reduction in presence: Number / percent of people reporting a reduction of abuse (reported for physical abuse, sexual abuse, harassment and stalking and jealous and controlling behaviour) $	1
			Severity of Abuse Grid - multiple types: Number / percent of people reporting multiple types of abuse$	7*
			Number / percent of participants experience concurrent domestic abuse as well as sexual abuse$	1
			Number / percent of participants who have been subjected to adult sex offences$	1
			Number / percent of men abusing each month$	1
			Space for Action Survey (% responding yes to 12 indicators) $	1
			Physical and Sexual Violence Survey (% responding yes to 7 indicators) $	1
			Harassment and other abusive acts survey (% responding yes to 6 indicators) $	1
			Fathering survey / using children against a partner (% responding yes to 5 indicators) $	2
			Social media perpetration / victimisation survey$	1
			Composite Abuse Scale$	3
			Abusive Behaviour Inventory$	2
			Controlling Behaviour Inventory$	3
		**Number of abusive incidents or behaviours**$		**4**
			Mean number of abusive incidents per man$	1
			Mean number of abusive behaviours reported by participants$	1
			Number / percent of participants experiencing between 0 and 7 behaviours (reported for emotional, physical and sexual behaviours) $	1
			IMPACT monitoring toolkit (reported for emotional, physical, and sexual behaviours) $	1
		**Severity of abuse**$		**29**

For peer review only

Category	Domain	Subdomain	Measure	N
			Severity of Abuse Grid - severity: Number / percent of participants reporting high / moderate / standard severity (reported for physical abuse, sexual abuse, harassment and stalking and jealous and controlling behaviour) $	8*,±
			Severity of Abuse Grid - reduction in severity: Number / percent of participants reporting a reduction of high / moderate / standard / unknown severity (reported for physical abuse, sexual abuse, harassment and stalking and jealous and controlling behaviour) $	1
			Severity of Abuse Grid – escalation in severity: Number / percent of participants reporting escalation in severity of abuse$	2
			Severity of Abuse Grid - severity: Number / percent of participants reporting any form of severe abuse$	2
			Severity of Abuse Grid - high severity: Number / percent of participants reporting severe abuse (reported for physical abuse, sexual abuse, harassment and stalking and jealous and controlling behaviour) $	1
			Severity of Abuse Grid - severity: Number / percent of participants reporting multiple types of abuse that are high severity$	6*
			Severity of Abuse Grid - severity: Number / percent of participants reporting at least one type of abuse that is high severity$	6*
			Cambridge Crime Harm Index$	3
		**Frequency of abuse$**		**14**
			Severity of Abuse Grid - frequency: Number / percent of participants reporting high / moderate / standard / unknown frequency (reported for physical abuse, sexual abuse, harassment and stalking and jealous and controlling behaviour) $	5*
			Severity of Abuse Grid - frequency escalation: Number / percent of participants reporting an escalation in frequency$	6*
			Frequency of abusive behaviours (scale 1 = never -3 = often) $	1
			Frequency rating of past violence (none (1) -> frequent (10) $	1
			Frequency rating of current violence (none (1) -> frequent (10) $	1
		**Duration of abuse$**		**1**
			Number / percent of victims with incidents recorded over a period longer than 12 months$	1
		**Severity and frequency of abuse$**		**13**
			Severity of Abuse Grid - severity and frequency escalation: Number / percent of participants reporting any escalation in severity or frequency of abuse$	7*
			Severity of Abuse Grid - severity and frequency escalation: Number / percent	6*

Category	Domain	Subdomain	Measure	N
			of participants reporting at least one form of abuse that is high severity and escalating in frequency or severity$	
		**Risk$**		**18**
			Spousal Assault Risk Assessment$	2
			DASH risk (high/med/standard) $	2
			Drive-DASH (mean score) $	1
			DASH risk (number with 1-14 assessments who had a decrease in score) $	1
			Change in risk in perpetrators (measurement tool unclear) $	1
			Weighted change in risk in perpetrators (measurement tool unclear) $	1
			IDVA perception of change in risk (do not know / increased / limited or no reduction / moderate reduction / significant reduction) $	1
			IDVA rated reduction in risk (percentage reporting 'some reduction in risk') $	1
			IDVA perception of sustainability of change in risk (Do not know / short term / medium term / long term / risk is permanently eliminated) $	1
			Men's perception of risk of using violence again (never/not sure/possibly) $	1
			Risk factors - Perpetrators' crimogenic behaviour and aggravating problems (frequency and percent of 7 factors) $	1
			Risk factors - Specific features of abuse (frequency and percent of 10 factors) $	1
			Risk factors - Victims' appraisals of threat (frequency and percent of 3 factors) $	1
			Risk factors - Factors relating to children (frequency and percent of 3 factors) $	1
			Risk factors - Factors relating to victim (frequency and percent of 4 factors) $	1
			Probability of none / standard / moderate / and high abuse (reported for physical abuse / sexual abuse / harassment and stalking / jealous and controlling behaviour) $	1
		**Police incidents$**		**39**
			Average / total number of police incidents$	1
			Average / total number of DV related police incidents$	2
			Average / total number of incidents recorded by the police per month$	1
			Average number of DV complaints per month$	1
			Average number of arrests made per month$	1

For peer review only

Category	Domain	Subdomain	Measure	N
			Average number of victims refusing to make a complaint per month$	1
			Average number of mental health related DV complaints per month$	1
			Average number of alcohol related DV complaints per month$	1
			Average number of drug related DV complaints per month$	1
			Average number of concern for children reports per month$	1
			Number / percent of participants that committed DV related police incidents$	1
			Number / percent of participants that committed (unspecified) police incidents$	1
			Number / percent of participants with (unspecified) arrests$	1
			Number / percent of calls to domestic incidents$	1
			Number / percent of repeat calls to domestic incidents$	1
			Number / percent of repeat calls to domestic violence incidents (number with 2 incidents, 3 incidents, 4 incidents… 14 incidents) $	1
			Number / percent of DV incidents where the perpetrator was not present$	1
			Number / percent of DV incidents that were crimed$	1
			Number / percent of DV incidents that led to arrests$	2
			Number / percent of incidents with an arrest where a statement is given$	1
			Number / percent of incidents with an arrest and a statement is given where it is then retracted$	1
			Number / percent of crimes where perpetrator was charged/summonsed$	1
			Number / percent of police incidents before and after a MARAC$	1
			Number / percent of high / medium / low severity police incidents before and after a MARAC$	1
			Number / percent of women experiencing no further post-MARAC incidents of violence or abuse$	1
			Number / percent of women experiencing no further post-MARAC police complaints or call outs$	2
			Number / percent of women experiencing no further post-MARAC police complaints$	2
			Number / percent of women experiencing no further post-MARAC police call outs$	2
			Number / percent of women experiencing either additional post-MARAC police complaints or call outs	1

For peer review only

Category	Domain	Subdomain	Measure	N
			Number / percent of women experiencing both additional post-MARAC police complaints and call outs	1
			Percent change in total calls to domestic incidents	1
			Percent change in total calls to domestic incidents (as proportion of all calls)	1
			Frequency of police call outs (never / once / 2-5 times / 6-10 times)	1
			Cost of police incidents	1

Note: Severity of Abuse Grid: there was a diverse approach to how it was used by different authors in the literature, , therefore we decided to split outcomes according to the various ways in which it was operationalised, rather than just reporting 'Severity of Abuse Grid' as an outcome.

* Note: Some studies do not explicitly specify using the Severity of Abuse Grid, but this has been presumed because the wording used when reporting outcomes matches exactly.

± Note: One study uses the outcome measure to assess perpetrators using abusive behaviours, rather than victim-survivor experiences of abuse.

$ Note: These outcomes and subdomains appear in more than one domain.

For peer review only

Figure 3: Temporal trends in outcome domains reported in DVSA evaluations, 1991 to 2022

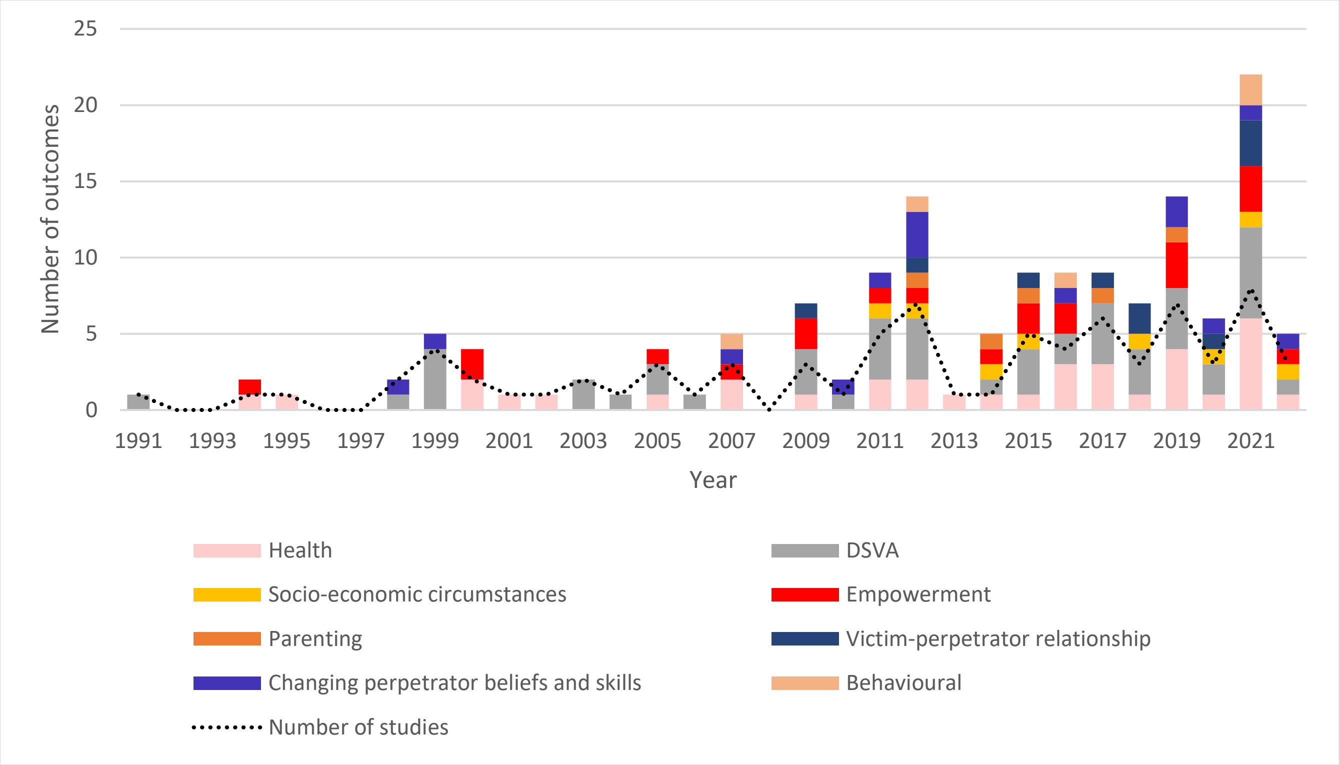

Note: For ease of interpretation, mental health, physical health and wellbeing domains have been combined into one health domain, and the experience of DSVA and perpetration of DSVA domains have been combined into a single 'DSVA' domain.
Note: Sixteen studies reported an outcome that crosses two or more domains therefore some outcomes are represented by multiple domains. Additionally, 38 studies reported an outcome that was categorised as both the experience of DSVA and perpetration of DSVA, because the specific outcome could represent either domain depending on who is completing the outcome measure.

BMJ Open

Trends in outcomes used to measure the effectiveness of UK-based support interventions and services targeted at adults with experience of domestic and sexual violence and abuse: A scoping review.

Journal:	*BMJ Open*
Manuscript ID	bmjopen-2023-074452.R2
Article Type:	Original research
Date Submitted by the Author:	19-Dec-2023
Complete List of Authors:	Carlisle, Sophie ; King's College London, Health Service and Population Research<br>Bunce, Annie; City University of London<br>Prina, Matthew; Newcastle University, Population Health Sciences Institute<br>Cook, Elizabeth; City University of London, Violence and Society Centre<br>Barbosa, Estela; University of Bristol, Population Health Sciences, Bristol Medical School; UKPRP VISION Consortium<br>McManus, Sally; City University of London; National Centre for Social Research<br>Feder, Gene; University of Bristol, Community based medicine<br>Lewis, Natalia; University of Bristol, Centre for Academic Primary Care, Population Health Sciences, Bristol Medical School; National Institute for Health Research Collaboration for Leadership in Applied Health Research and Care North Thames at Bart's Health NHS Trust, Queen Mary University of London, Centre for Primary Care and Public Health
**Primary Subject Heading**:	Public health
Secondary Subject Heading:	Public health
Keywords:	Systematic Review, PUBLIC HEALTH, MENTAL HEALTH

SCHOLARONE™
Manuscripts

I, the Submitting Author has the right to grant and does grant on behalf of all authors of the Work (as defined in the below author licence), an exclusive licence and/or a non-exclusive licence for contributions from authors who are: i) UK Crown employees; ii) where BMJ has agreed a CC-BY licence shall apply, and/or iii) in accordance with the terms applicable for US Federal Government officers or employees acting as part of their official duties; on a worldwide, perpetual, irrevocable, royalty-free basis to BMJ Publishing Group Ltd ("BMJ") its licensees and where the relevant Journal is co-owned by BMJ to the co-owners of the Journal, to publish the Work in this journal and any other BMJ products and to exploit all rights, as set out in our licence.

The Submitting Author accepts and understands that any supply made under these terms is made by BMJ to the Submitting Author unless you are acting as an employee on behalf of your employer or a postgraduate student of an affiliated institution which is paying any applicable article publishing charge ("APC") for Open Access articles. Where the Submitting Author wishes to make the Work available on an Open Access basis (and intends to pay the relevant APC), the terms of reuse of such Open Access shall be governed by a Creative Commons licence – details of these licences and which Creative Commons licence will apply to this Work are set out in our licence referred to above.

Other than as permitted in any relevant BMJ Author's Self Archiving Policies, I confirm this Work has not been accepted for publication elsewhere, is not being considered for publication elsewhere and does not duplicate material already published. I confirm all authors consent to publication of this Work and authorise the granting of this licence.

For peer review only

Trends in outcomes used to measure the effectiveness of UK-based support interventions and services targeted at adults with experience of domestic and sexual violence and abuse: A scoping review.

Sophie Carlisle[1], Annie Bunce[3], Matthew Prina[5], Elizabeth Cook[3], Estela C Barbosa[2], Sally McManus[3,4], Gene Feder[2], Natalia V Lewis[2]

Corresponding author

Sophie Carlisle: Department of Health Service and Population Research, Institute of Psychiatry, Psychology and Neuroscience, King's College London, De Crespigny Park, London, UK. Telephone/fax: N/A. Email: Sophie.r.carlisle@kcl.ac.uk

Author affiliations

1 Department of Health Service and Population Research, Institute of Psychiatry, Psychology and Neuroscience, King's College London, De Crespigny Park, London, UK

2 Bristol Medical School, University of Bristol, Bristol BS8 2PS, UK

3 Violence and Society Centre, City, University of London, London, UK

4 National Centre for Social Research, London, UK

5 Population Health Sciences Institute, Faculty of Medical Sciences, Newcastle University, Newcastle, NE4 5PL, UK

Key words

Scoping review, domestic abuse, sexual violence, perpetrator programme, outcomes

For peer review only - http://bmjopen.bmj.com/site/about/guidelines.xhtml

For peer review only

Author Contributions

All authors (SC, AB, MP, EC, SM, GF, NL) contributed to the conceptualisation and design of the review. SC carried out searches, screening, extraction, data charting, and analysis. AB was the second reviewer and contributed to the screening and extraction stages. SC produced the first draft of the manuscript and subsequent revised versions following valuable input and refinement from the co-authors (AB, MP, EC, SM, GF, NL). All authors approved the final version.

Funding statement

This research was supported by the UK Prevention Research Partnership (Violence, Health and Society; MR-VO49879/1), which is funded by the British Heart Foundation, Chief Scientist Office of the Scottish Government Health and Social Care Directorates, Engineering and Physical Sciences Research Council, Economic and Social Research Council, Health and Social Care Research and Development Division (Welsh Government), Medical Research Council, National Institute for Health and Care Research, Natural Environment Research Council, Public Health Agency (Northern Ireland), The Health Foundation, and Wellcome. The views expressed in this Article are those of the authors and not necessarily those of the UK Prevention Research Partnership or any other funder. The funder had no input in the design of the protocol or conduct of the review.

Competing interests statement

None declared.

Data availability statement

All data relevant to the study are included in the article and uploaded as supplementary information.

For peer review only - http://bmjopen.bmj.com/site/about/guidelines.xhtml

Word count: 5735

Abstract

Objectives: In the UK, a range of support services and interventions are available to people who have experienced or perpetrated domestic and sexual violence and abuse (DSVA). However, it is currently not clear which outcomes and outcome measures are used to assess their effectiveness. The objective of this review is to summarise, map and identify trends in outcome measures in evaluations and reports of DSVA services and interventions in the UK.

Design: Scoping review

Data sources: MEDLINE, EMBASE, PsycINFO, Social Policy and Practice, ASSIA, IBSS, Sociological abstracts and SSCI electronic databases were searched from inception until 21st June 2022. Grey literature sources were identified and searched.

Eligibility: We included randomised controlled trials, non-randomised comparative studies, pre-post studies and service evaluations, with at least one outcome relating to the effectiveness of the support intervention or service for people who have experienced and/or perpetrated DSVA. Outcomes had to be assessed at baseline and at least one more time-point, or compared to a comparison group.

Charting methods: Outcome measures were extracted, iteratively thematically grouped into categories, domains and subdomains, and trends were explored.

Results: 80 studies reporting 87 interventions or services were included. A total of 426 outcome measures were extracted, of which 200 were used more than once. The most commonly reported subdomain was DSVA perpetration. Cessation of abuse according to the Severity of Abuse Grid was the most common individual outcome. Analysis of temporal trends showed that the number of studies and outcomes used has increased since the 1990s.

For peer review only - http://bmjopen.bmj.com/site/about/guidelines.xhtml

Conclusions: Our findings highlight inconsistencies between studies in outcome measurement. The increase in the number of studies and variety of measures suggests that as evaluation of these services and interventions matures, there is an increased need for a core of common, reliable metrics to aid comparability.

Protocol registration: https://osf.io/frh2e

Article summary

Strengths and limitations of this study

- This scoping review adhered to PRISMA 2020 guidelines to robustly and systematically map effectiveness outcome measures used in evaluations of support interventions and services for people who have experienced and/or perpetrated DSVA.
- It included an extensive grey literature search to be comprehensive and inclusive.
- An advisory group consisting of representatives from six third sector organisations has been involved in the development of the review.
- The review has not included services or interventions for children who have experienced DSVA, interventions aimed at staff training, and is limited to the UK.
- Further research is urgently required to work towards agreement on which outcome measures should be prioritised and adopted, whilst also avoiding burden on systems and people experiencing DSVA.

For peer review only

Introduction

Domestic and sexual violence and abuse (DSVA; comprising a range of different behaviours and experiences, see Appendix 1) represents a significant international public health issue [1-3]. Approximately 1 in 3 women and 1 in 7 men in England and Wales have experienced domestic violence and abuse in their lifetime [4]. Globally, over 35% of women have experienced either physical and/or sexual intimate partner violence or non-partner sexual violence [5]. Poverty, homelessness, and insecure immigration status heighten DSVA vulnerability [6, 7], and the risk of experiencing DSVA is also higher for younger adults, lesbian, gay, bisexual and transgender (LGBT) people [8-11], and people with long term illness, disability or mental health problems [12, 13]. The UK does not currently routinely collect data on perpetrators, however studies have found that the prevalence of DSVA perpetration ranges from 11.6-80.0% depending on country, gender and definition of DSVA [14, 15]. Perpetrators are typically male [16] and are often serial perpetrators with more than one victim [17, 18]. The impacts of DSVA are profound and diverse. As well as visible and immediate consequences such as physical injury or death, DSVA has been associated with longer-term physical health [19-25] and mental health problems [26-29]. Other areas impacted by DSVA include homelessness [30], access to finances, employment and education [31], and increased demand on the criminal justice system [32], social services [33], and police [34].

Given the wide-ranging impacts of DSVA, the support options available to people who have experienced it are diverse. Interventions and services include refuges, outreach, counselling, financial or legal advice, helplines and advocates. In the UK, these are often provided by third sector organisations (organisations that are neither public nor private sector, including voluntary and community organisations; see Appendix 1), but can also be provided by services located in the public and private sectors. Some services are targeted at particular groups with heightened vulnerability to DSVA, such as LGBT+ and Black and Minority Ethnic

For peer review only - http://bmjopen.bmj.com/site/about/guidelines.xhtml

(BME) victim-survivors or victim-survivors with disability, however provision for these groups is stark and disproportionately underfunded [35-37]. Several theoretical frameworks have been developed to explain how such support services and interventions may work. For instance, the socio-ecological model of violence against women highlights key risk factors at the individual, relationship, community and societal levels, identifying potential intervention points for preventing and responding to violence [38]. One of the risk factors identified by Heise is social isolation, therefore one component of a support intervention may be to increase social support and build relationships. Sullivan's conceptual model of DV support services [39] builds on the Conservation of Resources theory, which suggests that psychological distress following trauma is influenced by the loss of economic, social and interpersonal resources central to well-being [40-42]. Based on this, Sullivan provides an exemplar model for support services which outlines eight common programme activities aimed at creating communities that value their members and promote well-being, though intrapersonal and interpersonal changes, which impact a range of intrapersonal and interpersonal social and emotional well-being outcomes.

Previous systematic reviews of the global evidence have found benefits of such interventions on a range of social and emotional well-being related outcomes, including housing interventions improving mental health, perceived safety and stress [43], economic interventions reducing levels of domestic violence and increasing empowerment [44], advocacy interventions improving quality of life and depression [45], and psychological therapies reducing depression and anxiety [46]. Service evaluations and annual reports produced by local DSVA organisations have also found associations between service use and improved perceptions of safety, quality of life, health and wellbeing, and confidence [47-51]. Finally, reviews of perpetrator programmes (i.e., interventions targeted at people who use violence) have provided some evidence of reductions in the perpetration and experience of abusive behaviours, although highlighted methodological issues [52, 53].

For peer review only

Despite existing reviews identifying potential benefits of specific types of support services and interventions, there is limited understanding of which outcomes and outcome measures are currently being used both within and across UK-based support services and interventions when assessing their effectiveness [54, 55]. The specific outcome measures that are being used appear to vary, making it difficult to compare and synthesise the overall evidence [56]. Previous research in the UK and US literature that has explored outcome measurement in the domestic violence field has noted the diversity of reported outcomes (e.g. [57, 58]) and highlighted various issues and difficulties surrounding outcome measurement, which contributes this diversity [59, 60]. These studies point to the differing priorities of funders and service providers, and the diversity of specific service goals and objectives, which are often multiple and complex, as key drivers behind the range of outcomes being utilised. This literature also includes discussions on what should be measured, with several pushing for the extension and diversification of outcomes and measurement strategies, potentially contributing to the diversity of outcomes being used. Identifying the most commonly utilised outcome measures will allow for meta-analysis, leading to more rigorous evaluations of the literature, and may also guide interventions and services on which outcome measures to use. Scoping reviews present an opportunity to map the evidence, and are ideal for summarising heterogenous evidence [61, 62]. This scoping review aims to summarise and map trends in the outcome measures being used to evaluate UK-based support interventions and services for DSVA.

Objectives

Specific objectives of this scoping review include:

1. Identify and thematically group outcome measures that have been used to assess the effectiveness of UK-based support interventions and services for adults who have experienced and/or perpetrated DSVA.
2. Identify which outcome measures have been used most frequently.

For peer review only - http://bmjopen.bmj.com/site/about/guidelines.xhtml

3. Explore whether the outcome measures differ by: the type of victim-survivor or perpetrator support; DSVA type; source of the study; study setting; and over time.

Review question

What outcomes relating to effectiveness have been measured in intervention studies and service evaluations of UK-based support programmes for adults who have experienced and/or perpetrated DSVA?

Methods

Protocol and registration

The protocol was developed according to the Preferred Reporting Items for Systematic Reviews and Meta-Analysis Extension for Scoping Reviews (PRISMA-ScR) [63] and published on Open Science Framework: https://osf.io/frh2e [64]. The PRISMA-ScR checklist is available in Appendix 2.

Eligibility criteria

We included any UK-based secondary or tertiary prevention support services and interventions aimed at adults who had experienced or perpetrated DSVA. Primary prevention services and interventions were excluded as they aim to prevent DSVA before it occurs, and therefore focus on individuals or populations who have not necessarily experienced violence. Interventions and services that were not specifically aimed at DSVA, such as general trauma interventions, were only included if the majority of participants (>50%) had experienced DSVA. Randomised controlled trials (RCTs), non-randomised comparative trials, pre-post studies and service evaluations that reported effectiveness outcomes at two or more time-points or made comparisons to another group were included, so that cause and effect could be inferred. Perpetrator programmes were considered for several reasons. Firstly, including them allows the measurement of DSVA without placing the burden on people who have

For peer review only - http://bmjopen.bmj.com/site/about/guidelines.xhtml

experienced DSVA to change someone else's behaviour. This ties in to the UK government's new perpetrator strategy [65], which intends to place the onus on perpetrators changing their behaviour, alongside the recovery of those who have experienced DSVA. Finally, perpetrator programmes provide support for perpetrators to change their behaviour, and many offer associated support to (ex)partners or referral to appropriate support. Only English language studies were eligible for inclusion. Given the focus on UK-based support services and interventions, we considered the impact of this restriction on the comprehensiveness of the review to be minimal, however this does mean that the results of this review cannot be generalised to contexts outside of the UK. Details of the full eligibility criteria can be found in Table 1.

Table 1: PICO Inclusion and Exclusion Criteria

	Inclusion	Exclusion
Population	• Adults (16 years or older) who have experienced DSVA.<br>• Adults (16 years or older) who have perpetrated DSVA.	• Children<br>• Adults who have not experienced and/or perpetrated DSVA
Intervention (and service)	• Any secondary or tertiary prevention intervention and/or service for DSVA e.g., housing (e.g., refuges, housing workers, resettlements), Advocacy (e.g., Independent Domestic Violence Advisers/Advocates (IDVAs), Independent Sexual Violence Advisers/Advocates (ISVAs)), outreach, open access (e.g., helplines, drop-ins, online chats), psychological support (e.g., support groups, counselling, befriending), legal support, financial support, multi-agency risk assessment conferences (MARACs), and police-based services.<br>• Perpetrator programmes (see Appendix 1).<br>• Entry to the intervention had to be determined by the either the experience of DSVA (for victim-survivor support interventions) or perpetrating DSVA (for perpetrator programmes).	• Pharmacological<br>• Primary prevention<br>• Not primarily aimed at people with experience of DSVA

For peer review only - http://bmjopen.bmj.com/site/about/guidelines.xhtml

	Inclusion	Exclusion
Comparison	• RCTs:<br>○ Another type of included intervention or service.<br>○ Usual care<br>○ No intervention or service<br>• Pre-post designs:<br>○ Before and after the intervention or service	• No comparison
Outcome	• Any outcomes used to measure effectiveness of DSVA support services and interventions.	• Those not focussed on the effectiveness of the intervention or service (e.g., process evaluation outcomes such as satisfaction, staff training, etc)
Setting	• UK based	• Not UK based
Study design	• RCT<br>• Nonrandomised comparative<br>• Pre-post<br>• Service evaluation	• Cross-sectional, case control, case study<br>• Qualitative<br>• Letters to the editor, think pieces, descriptive only
Dates	• 1982-present	• Pre-1982

Information sources and search strategy

MEDLINE, EMBASE, PsycINFO, Social Policy and Practice, ASSIA, IBSS, Sociological abstracts and SSCI electronic databases were searched from inception until 21st June 2022. Grey literature sources were also identified, including searching electronic grey literature databases (National Grey Literature Collection, EThOS, Social Care Online and the Violence Against Women Network), a call for evidence which was shared with research networks and UK-based DSVA services and organisations, and searches of relevant UK charity and organisation websites. Finally, backwards and forwards citation tracking of all included studies, as well as reference searching of identified systematic reviews was undertaken to identify further relevant studies. The search results were exported into EndNote and duplicates were removed. An example of the search strategy for the peer review search and the grey literature search can be found in Appendix 3.

For peer review only

For peer review only - http://bmjopen.bmj.com/site/about/guidelines.xhtml

Selection of sources of evidence

The de-duplicated citations were uploaded into Rayyan [66]. Titles and abstracts, followed by full-texts, were screened according to the inclusion and exclusion criteria. A 20% sample was independently screened by a second reviewer at each stage, and all disagreements were resolved by discussion, with a third reviewer where needed. Risk of bias assessments are not necessary for scoping reviews [61].

Data charting process and data items

Data charting took place using a piloted excel spreadsheet. Data items extracted included the study citation, study design, participant demographics, description of the service or intervention and comparison (if applicable), setting, and the outcome measures used. All outcomes that were used to assess the effectiveness of DSVA support interventions and services were extracted. Data extraction and charting was carried out by one reviewer, and checked for errors by a second reviewer. Where data was missing, corresponding authors were contacted and asked to supply said data. After two weeks, if there had been no response, corresponding authors were contacted a second time and given a minimum of an additional two weeks to respond.

Synthesis of results

A narrative synthesis was conducted to summarise the included interventions and services and the effectiveness outcomes they measured. The unit of analysis was the outcome measure(s) reported. Interventions and services were grouped by the type of support offered, based on the intervention and service types outlined in the protocol. Where studies reported more than one form of support as part of the intervention or service, they were classified as “multi-service”. Where the same intervention or service produced multiple reports (e.g., from different years), these were combined into one entry during data charting

For peer review only - http://bmjopen.bmj.com/site/about/guidelines.xhtml

so that all outcomes ever reported by that service were extracted, but were not double counted if multiple reports used the same outcome.

Effectiveness outcomes reported by studies were grouped thematically by type to create categories, domains and subdomains. The groupings and the names for the sub-domains, domains and categories were developed iteratively. All outcome measures were firstly listed in an excel spreadsheet. This included standardised and unstandardised questionnaires and single item measures. A column was added, describing what the outcome measured (e.g., the Beck Depression Inventory [67] measures depression). These sub-domains were then grouped into broader domains (e.g., depression is a mental health-related outcome). Where applicable, these were grouped into again even broader categories (e.g., mental health is a form of overall health).

A list of all relevant outcomes and domains, and how frequently they were reported was made. Comparisons were made between outcomes reported by studies in different sectors, over time, and by service user (i.e., perpetrator or victim-survivor) and support type (i.e., psychological support, housing, combinations), or violence type (i.e., domestic violence and abuse [DVA], sexual violence and abuse [SVA], childhood sexual abuse [CSA]).

Patient and public involvement

An advisory group was set up comprising representatives from six specialist DSVA organisations who are involved in the delivery, planning, funding or support of specialist DSVA support services in the UK. Specifically, the group included representatives from two second-tier (i.e., organisations that support front-line services but do not provide services themselves) domestic abuse organisations, one second-tier organisation for violence against BME women and girls, one domestic abuse organisation that provides a range of front-line services, one service focusing specifically on supporting male victims, working with

For peer review only - http://bmjopen.bmj.com/site/about/guidelines.xhtml

perpetrators of domestic violence, and working with young people using violence in close relationships, and one service focusing specifically on sexual violence and abuse. The group inputted to the design of the study protocol, interpretation of the data, and provided insight regarding the challenges in measuring the effectiveness of support services in the third sector. Their input resulted in several changes, including the broadening of the scope of this review to all effectiveness outcomes, rather than a narrower focus on only outcomes relating to reducing violence, to reflect the priorities of the sector.

Results

Selection of sources of evidence

After duplications were removed, a total of 12431 records and 869 reports were screened for inclusion. Eighty studies from 111 reports were included in this review (see Figure 1). Data extracted from the included studies are summarised in Appendix 4, and Figure 2 [51, 68-177].

Characteristics of sources of evidence

The 80 studies described a total of 87 interventions or services, with seven studies describing both a perpetrator programme and an associated support service for (ex)partners of participating perpetrators. All studies were conducted between 1991 and 2022.

Description of studies

Study design

Many of the included studies were mixed-method service evaluations; for these studies, only the methodology relevant to the included outcomes is reported as the other methods do not meet the review eligibility criteria (e.g., qualitative, case studies). The most common study design was pre-post single group designs (n=53), of these 14 included additional time-points at mid-intervention or follow-up. Eight studies were RCTs, and ten used a non-randomised

For peer review only - http://bmjopen.bmj.com/site/about/guidelines.xhtml

comparative design. For one study, the design could not be determined. Eight studies used secondary analysis of datasets, which collated data from multiple services. These were primarily publications produced by SafeLives, a UK-based charity dedicated to ending domestic abuse, using their Insights system, an outcome measurement tool which allows services across England and Wales to collect data according to various measurement indicators.

Perpetrator programmes

Perpetrator programmes were grouped together. They were typically described as behaviour change programmes based on a cognitive behavioural model, and whilst many included additional elements such as psychological support, these were often described in insufficient detail. Of the 80 included studies, 27 described a perpetrator programme.

Victim-survivor support interventions and services

Interventions and services providing support for those who had experienced DSVA were grouped by type of support provided, based on initial scoping of the literature. The most common were psychological support (n=21), advocacy (n=14) and multi-service (n=11). Three studies each reported on multi-agency risk assessment conferences (MARACs) and specialist domestic violence police teams. Two studies reported housing interventions or services. One study each reported on sexual violence services, a specialist domestic violence court, helpline services, outreach services, and adult health-based services. One study described a women's service linked to an associated perpetrator programme, but the specific type of support offered was unclear. Several studies evaluated the effectiveness of more than one intervention or service. Of the 60 victim-survivor support interventions and services, two were specifically targeted at male victim-survivors, one at BME women, one at victims of trafficking, and one at women with disabilities. There was no evidence for interventions or services targeted specifically at LGBT+ victim-survivors.

For peer review only - http://bmjopen.bmj.com/site/about/guidelines.xhtml

Type of DSVA

The studies were categorised as being primarily aimed at DVA, SVA, both DVA and SVA (DSVA), or CSA. Categorisations were based on how the study or service described itself, however it should be noted that the type of violence/abuse was often not clearly defined. Thus, these categorisations should be considered as having ambiguous boundaries, and interpreted with caution.

The majority (n=53; 66.3%) of the studies were aimed at DVA, whilst 24 (29.3%) were aimed specifically at SVA. Of these, nine studies were specifically targeted at adults who had experienced CSA. Three studies (3.8%) were aimed at both DVA and SVA, or DSVA.

Source of the studies

Of the 80 studies and 111 reports, 41 studies from 47 reports were located in the peer-reviewed literature, and 39 studies from 64 reports were located in the grey literature.

Setting

Just under half of studies were conducted in the third sector (n=39; 47.6%). Twenty-six studies were conducted in the public sector, and 12 spanned multiple sectors. One was conducted in a privately owned prison, and for one study the setting was unclear.

Types of outcomes

The 80 included studies measured a total of 426 outcomes. Of these 200 (47.0%) were used more than once. In total, there were 282 unique outcomes, 57 of which were used more than once, and 226 were used only once. The number of effectiveness outcomes measured by each individual study ranged from one to 19, with a mean average of 5.23.

For peer review only - http://bmjopen.bmj.com/site/about/guidelines.xhtml

Outcomes were grouped thematically into 11 domains and 49 subdomains. The 11 domains included mental health, physical health, general wellbeing (all of which fall under the broad category of ‘health’), the experience of DSVA and the perpetration of DSVA (which fall under the broad category of ‘DSVA’), empowerment, socio-economic circumstances, behavioural outcomes, the victim-perpetrator relationship, parenting, and changing perpetrator beliefs and skills (Appendix 5).

Many of the outcome measures reported spanned two or more domains (Figure 3). For instance, ‘health’ as an overarching category included three domains: mental health, physical health and general wellbeing. ‘Quality of life’ as a subdomain spanned physical health, mental health and general wellbeing domains, based on the identified outcome measures and their specific items. For example, the 12-item Short Form (SF-12) is a measure of quality of life and general health, which includes items relating to physical health, mental health, and general wellbeing. Similarly, the overarching category of DSVA contained two domains: the experience of DSVA and the perpetration of DSVA. However, many outcome measures could fall into either domain, depending on who the respondent was (i.e., the perpetrator or the victim-survivor). For example, the outcome ‘presence of abuse’ would fall into the ‘experience of DSVA’ domain if the respondent was the victim-survivor, and the ‘perpetration of DSVA’ domain if the respondent was the perpetrator. Thus, there was substantial overlap between these two subdomains. Many of the outcomes within the DSVA category, whilst defined slightly differently, were not distinct. For instance, there is significant overlap between the concept of ‘revictimization’ and the outcome ‘presence of abuse’ when measured using a pre-post design (if the victim-survivor indicates that they are still experiencing abuse post-intervention, then this indicates ‘revictimization’). There is similarly overlap between reoffending and revictimization (although we cannot presume a direct overlap, as a perpetrator may have multiple victims) and between ‘reoffending’ and ‘police incidents’ (although not all reoffending will be recorded by police therefore this subdomain may be broader).

For peer review only - http://bmjopen.bmj.com/site/about/guidelines.xhtml

For each of the 11 domains, the total number of outcomes, the total number of unique outcomes, and the number of studies reporting at least one outcome that fell within that domain, were explored. The domain ‘perpetration of abuse’ has the highest number of studies reporting at least one outcome, the most total outcome measures, and the most unique outcome measures (Table 2).

For peer review only - http://bmjopen.bmj.com/site/about/guidelines.xhtml

Table 2. Frequency of outcomes by outcome domain, and the number of studies reporting one or more outcomes per outcome domains by intervention type, source of study, violence type and sector

		Outcome measure domain										
		Mental health	Physical health	Wellbeing	Behavioural	Empowerment	Socio-economic circumstances	Changing perpetrator beliefs and skills	Victim-perpetrator relationship	Parenting	Experience of DSVA	Perpetration of DSVA
Number of studies reporting at least one outcome		29	11	19	5	21	9	14	10	5	42	46
Number of total outcome measures		85	22	25	11	32	17	33	12	7	189	201
Number of total unique outcome measures		52	16	21	11	23	17	28	7	5	106	119
**Type of intervention**	**Number of studies**											
Perpetrator programme	27	2	1	2	1	1	1	14	4	3	14	21
Victim support:												
Psychological support	21	17	1	7	2	10	2	0	0	1	1	0
Advocacy	14	5	4	4	0	4	2	0	2	1	12	11
Multi-service	11	4	4	4	1	5	1	0	0	0	8	5
Housing	2	0	0	0	0	0	1	0	1	0	1	1
MARAC	3	0	0	0	0	0	0	0	0	0	3	2
Specialist DV police teams	3	0	0	1	0	0	0	0	0	0	2	2
Specialist DV court	1	0	0	0	0	0	0	0	0	0	0	1
Sexual violence service	1	1	1	1	1	1	1	0	0	0	1	1
Helpline	1	0	0	0	0	0	0	0	1	0	1	1
Outreach	1	0	0	0	0	0	0	0	1	0	1	1

For peer review only - http://bmjopen.bmj.com/site/about/guidelines.xhtml

Health	1	0	0	0	0	0	1	0	1	0	1	1
Other / Unclear	1	0	0	0	0	0	0	0	0	0	1	1
**Source**												
Peer reviewed	41	22	6	10	3	11	3	9	1	3	13	19
Grey literature	39	7	5	9	2	10	6	5	9	2	29	27
**Type of violence**												
DVA	53	11	9	12	2	10	6	8	10	5	40	41
SVA	15	7	2	4	2	5	1	6	0	0	1	4
Both	3	2	0	0	0	1	0	0	0	0	1	1
CSA	9	9	0	3	1	5	2	0	0	0	0	0
**Sector**												
Third	39	12	7	14	3	12	7	5	9	4	27	23
Public	26	13	2	3	1	8	2	5	0	1	5	10
Mixed	12	3	2	1	1	0	0	2	1	0	9	11
Private	1	1	0	1	0	1	0	1	0	0	0	0
Unclear	2	0	0	0	0	0	0	1	0	0	1	2

Note: CSA: Childhood sexual abuse; DSVA: Domestic and sexual violence and abuse; DV: Domestic violence; DVA: domestic violence and abuse: MARAC: Multi-agency risk assessment conference; SVA: Sexual violence and abuse

Note: Sixteen studies reported an outcome that fits into multiple domains therefore some outcomes are represented by multiple domains. Additionally, 38 studies reported an outcome that was categorised as both the experience of DSVA and perpetration of DSVA, because the specific outcome could represent either domain depending on who is completing the outcome measure.

Note: Two studies have outcome domains that are represented in multiple intervention types (perpetrator programme, unclear/other and advocacy), because they report a perpetrator programme and an associated women's service, but it is unclear which intervention/service the outcomes were measured as part of.

Note: The number of studies for the different intervention/service types is more than the total number of studies (80) because 7 studies reported a perpetrator programme and associated women's service.

For peer review only - http://bmjopen.bmj.com/site/about/guidelines.xhtml

Of the three most common outcome measures across intervention types, all fell within the DSVA category (experience of DSVA / perpetration of DSVA; Table 3). Cessation of abuse according to the Severity of Abuse Grid (SAG) [178] was the most common outcome (n=11), followed by the presence of abuse (n=10) and the severity of abuse (n=8), both also measured by the SAG. The Rosenberg Self-Esteem Scale [179], and two more measures of DSVA from the SAG (multiple types of abuse; abuse that is escalating in severity or frequency) were each reported by seven studies.

Table 3: Most commonly reported outcome measures

Outcome	Subdomain	Domain	No. of studies
**Overall**			
1. Cessation of abuse measured by the SAG [178]	Presence of abuse	Experience of DSVA/ perpetration of DSVA	11
2. Presence of abuse measured by the SAG* [178]	Presence of abuse	Experience of DSVA/ perpetration of DSVA	10
3. Severity of abuse measured by the SAG* [178]	Severity of abuse	Experience of DSVA/ perpetration of DSVA	8
4. RSES [179]	Self-esteem	Empowerment	7
5. Multiple types of abuse measured by the SAG [178]	Presence of abuse	Experience of DSVA/ perpetration of DSVA	7
6. Abuse that is escalating in severity or frequency as measured by the SAG [178]	Severity and frequency of abuse	Experience of DSVA/ perpetration of DSVA	7
**Peer review vs grey literature**			
**Grey literature**			
1. Cessation of abuse measured by the SAG [178]	Presence of abuse	Experience of DSVA/ perpetration of DSVA	9
2. Presence of abuse measured by the SAG* [178]	Presence of abuse	Experience of DSVA/ perpetration of DSVA	9
3. Severity of abuse measured by the SAG* [178]	Severity of abuse	Experience of DSVA/ perpetration of DSVA	8
**Peer review**			
1. RSES [179]	Self-esteem	Empowerment	6
2. BDI [67]	Depression	Mental health	5
3. CORE-OM [180]	Psychological issues	Mental health	5
**Sector**			
**Third**			
1. Cessation of abuse measured by the SAG [178]	Presence of abuse	Experience of DSVA/ perpetration of DSVA	9
2. Presence of abuse measured by the SAG* [178]	Presence of abuse	Experience of DSVA/ perpetration of DSVA	9
3. Severity of abuse measured by the SAG* [178]	Severity of abuse	Experience of DSVA/ perpetration of DSVA	8
**Public**			

For peer review only - http://bmjopen.bmj.com/site/about/guidelines.xhtml

Outcome	Subdomain	Domain	No. of studies
1. BDI [67]	Depression	Mental health	5
2. RSES [179]	Self-esteem	Empowerment	4
3. SCL-90R [181]	Psychological issues	Mental health	4
**Mixed**			
1. Cases discontinued	Criminal justice outcomes	Perpetration of DSVA	2
2. PHQ [182]	Depression	Mental health	2
3. GAD-7 [183]	Anxiety	Mental health	2
4. Cambridge Crime Harm Index [184]	Severity of abuse	Experience of DSVA/ perpetration of DSVA	2
5. PC-PTSD [185]	Trauma	Mental health	2
6. No further post-MARAC police complaints or call outs	Police incidents	Experience of DSVA/ perpetration of DSVA	2
7. No further post-MARAC police complaints	Police incidents	Experience of DSVA/ perpetration of DSVA	2
8. No further post-MARAC police call outs	Police incidents	Experience of DSVA/ perpetration of DSVA	2

Note: BDI: Beck Depression Inventory; CORE-OM: Clinical Outcomes in Routine Evaluation Outcome Measure; DSVA: Domestic and sexual violence and abuse; GAD-7: Generalised Anxiety Disorder Questionnaire; MARAC: Multi-agency risk assessment conference; PHQ: Patient Health Questionnaire; PC-PTSD-5: Primary Care PTSD Screen; RSES: Rosenberg Self-Esteem Scale; SAG: Severity of Abuse Grid; SCL-90R: Symptom Checklist 90 revised.
**Reported separately for physical abuse, sexual abuse, harassment and stalking, and jealous and controlling behaviour*

Patterns in the use of outcome

Table 2 summarises the patterns in outcome domains according to perpetrator programmes, victim support type, DSVA type, source of literature and setting/sector.

Outcomes in perpetrator programmes

Of the 27 perpetrator programmes, 14 reported at least one outcome relating to changing perpetrator behaviour, and 21 reported at least one relating to the perpetration of DSVA.

Outcomes by victim support type

The most common outcome domains for psychological support interventions and services were mental health (n=17) and empowerment (n=10), whilst advocacy, multi-service interventions and studies of MARACs primarily reported on experience of DSVA (n=12, n=8 and n=3, respectively). Specialist DV police teams reported equally on the experience of and the perpetration of DSVA (n=2 each).

For peer review only - http://bmjopen.bmj.com/site/about/guidelines.xhtml

Outcomes by type of DSVA

Of the studies primarily focused on DVA, the majority reported at least one outcome relating to the experience of DSVA (n=40; 75.5%) and the perpetration of DSVA (n=41; 77.4%). Just under half of studies of adult SVA (n=7; 46.7%), and all studies of CSA, reported mental health outcomes (n=9; 100%).

Outcomes by source of studies

Whilst the split of included studies from the grey literature and peer reviewed literature was relatively even, studies located within the grey literature were more diverse. In total, grey literature studies reported 254 outcome measures, 178 of which were unique. In comparison, peer reviewed studies reported a total of 172 outcomes, 125 of which were unique. Peer reviewed studies had more of a tendency to report at least one outcome relating to mental health (n=22; 53.7%), whilst grey literature studies were more likely to report outcomes relating to the experience of DSVA (n=29; 74.4%).

Outcomes by setting

Two thirds of studies set in the third sector reported outcomes relating to the experience of DSVA subdomain (n=27; 69.2%). Perpetration of DSVA was the most common outcome subdomain for interventions and services with a mixed setting (n=11; 91.7%), whilst mental health was the most common subdomain for those in the public sector (n=13; 50%).

Outcomes 1991 to 2022

Evaluations of DSVA support services and interventions have become more frequent over time. Nearly half (48.8%) of included studies were published in 2015-2022, with over a quarter (26.3%) published in 2019-2022 (Appendix 6).

For peer review only - http://bmjopen.bmj.com/site/about/guidelines.xhtml

For peer review only

Discussion

This study used scoping review methodology to map which outcomes have been used in studies assessing the effectiveness of a range of support interventions and services for people who have experienced or perpetrated DSVA. A total of 426 outcome measures were used in 80 papers and reports, describing 87 interventions and services. Less than half of the measures were used more than once.

The most common outcome domain was the perpetration of DSVA, followed closely by the experience of DSVA, both of which included outcomes relating directly to abusive and violent behaviours. This demonstrates that the measurement of DSVA has been prioritised when assessing service and intervention effectiveness, and is consistent with the common goal of many DSVA support services and organisations of ending violence and abuse [186, 187]. However, 201 and 189 unique outcome measures were charted within these two domains respectively, showing the lack of consensus regarding the specific outcomes that should be measured. The SAG, which measures several different aspects of abusive behaviours (including presence of four types of abuse, frequency, severity, and cessation of abuse), was the most frequently used outcome measure. It was primarily used by studies drawing upon the SafeLives' Insights datasets, although it was employed by several additional studies too. Despite being the most frequently reported outcome, the SAG was still utilised by only 13 of the 80 studies, illustrating the inconsistencies and variation in measures being used by these evaluations and reports.

Of the 282 unique outcomes reported, 226 were used only once. Although many of these outcomes were similar, they often differed slightly according to the unit or method of measurement, meaning that combining them would be methodologically inappropriate. This further demonstrates the disparate and highly variable nature of outcome measures currently

For peer review only - http://bmjopen.bmj.com/site/about/guidelines.xhtml

in use. Whilst this may be partly explained by the fact that different sectors and types of support interventions have different priorities, such disparity is evident even *within* sectors and intervention types. For instance, of the 153 different outcome measures reported by third sector studies, only 24.8% were used more than once, with a similarly low proportion in mixed sector and public sector studies (both 30.7%). This indicates a lack of comparability surrounding the best way to assess the effectiveness of support services and interventions, and makes synthesis such as meta-analysis less feasible.

Mapping revealed differences between the types of outcome domains frequently reported in the peer reviewed and grey literature, with peer reviewed studies focusing more on mental health related outcomes, and grey literature studies focusing primarily on DSVA. This difference may be explained by the overlap between grey literature and third sector-based studies, and the fact that third sector organisations are heavily tied to the outcomes that funders and commissioners require them to measure. With the focus on wanting to establish and reduce the prevalence of DSVA itself, and the associated focus on the perpetration of violence, it might be that more funders and commissioners are requiring these to be measured, or are prioritising these measurements above the wider impacts of DSVA.

Mapping of trends over time showed that as well as a general increase in the number of studies being conducted, there appeared to be increased diversity in outcomes measured. The two most common outcome categories, DSVA and health, have been measured fairly consistently over the past thirty years, whilst domains such as empowerment, the victim-perpetrator relationship and parenting have become more common in the past decade. This is likely reflective of an increased knowledge of the wide-ranging impacts of DSVA and its ripple effects, increased recognition of the need for holistic support, and the move towards survivor-centred outcome measurement and consultation with those with lived experience [188].

For peer review only

Overall, this review has demonstrated that the types of outcomes measured when assessing the effectiveness of support interventions and services for people who have experienced DSVA vary widely. There are several potential reasons for this diversity in outcome measures. Firstly, as previously mentioned, DSVA has many impacts, including on physical health, mental health, housing, finances, relationships, and many more. Therefore, there are many avenues through which support services and interventions could have impact and improve outcomes. Indeed, in their review Sprague, McKay [57] noted that many services have multiple goals that cannot be assessed by a single outcome, thus multiple outcomes are required. Secondly, differences between the priorities of funders, services and service users, and researchers in terms of outcome measurement likely results in diversity in outcomes and differences in outcome measures utilised between sectors [58, 59]. Thirdly, there is currently a lack of consensus across the field regarding what outcomes should be measured and, importantly, how. For instance, while it may be agreed that 'safety' is an important outcome to assess, how this is operationalised and measured is not consistent.

Challenges in outcome measurement

There are two linked challenges facing UK-based DSVA services, especially those located in the third sector. Firstly, funding of DSVA services is precarious and diminishing [189-192]. Being awarded funding requires evidence of effectiveness. However, the second challenge is that carrying out service evaluations to provide evidence of effectiveness is costly, making it even more difficult for services to secure funding. This is reflected in take-up of the aforementioned SafeLives' Insights system, which captures rich data and has likely contributed to more consistent reporting of outcomes across England and Wales. However, individual services have to pay to use the system, and the numbers of services contributing to the dataset each year has reduced, likely due to reduced funding [consultation with stakeholder advisory group].

For peer review only - http://bmjopen.bmj.com/site/about/guidelines.xhtml

Limitations

One limitation of this review is the unavoidable potential for double counting. For instance, the services that are included in SafeLives' Insights datasets are anonymous. Thus, it is not possible to determine if any services included in the Insights datasets have also published an independent evaluation. A second limitation of this review is that despite a comprehensive search strategy, no evaluations of support services run by by-and-for organisations were identified (see Appendix 1). This is illustrative of a systemic, cyclical and enduring issue facing these smaller services in particular, who are disproportionally underfunded and lack the resources needed to conduct and publish evaluations, meaning they are underrepresented in the literature [36, 193-195]. Further, whilst the search strategy was comprehensive and designed in collaboration with the advisory group to ensure key search terms were included, the search was not exhaustive, and therefore it is possible that had we selected different databases or included additional terms, we may have identified additional relevant papers. Additionally, because of time and resource restrictions, only 20% of records were dual screened during the title/abstract and full text screening stages, which does increase the risk of relevant studies being incorrectly excluded. Further, we excluded process evaluation outcomes. Given that there may be causal links between process measures and distal outcomes, it is possible that some relevant outcomes were missed. Finally, given the primary objectives of the review were to map and chart outcome measures being used, we have not made any assessments regarding the validity or relevance of any particular measure. Therefore, whilst we have identified the most commonly used outcome measures, that is not to say that these are the most helpful, informative or important to people with lived experience or front-line service providers.

Implications and recommendations

The findings from this scoping review regarding outcomes most commonly measured to assess the effectiveness of DSVA interventions highlight how support services are working

For peer review only - http://bmjopen.bmj.com/site/about/guidelines.xhtml

to promote survivors' safety and wellbeing. However, the diversity of the specific outcomes and measurement tools used has implications for researchers, service providers, policymakers and funders .

Implications and recommendations for researchers

Increased consensus between researchers, service providers, policymakers and funders is needed. This will allow for more meaningful syntheses of the literature, as well as building a larger evidence base, so that a better understanding of the most effective means of support for people who have experienced DSVA can be reached. Thus, further research is needed to extend ongoing work on the development of shared outcome frameworks, to determine which outcomes and outcome measures are most appropriate, valid and relevant, in order to work towards a consensus and build a shared evidence base to enable future meta-analyses. These outcome frameworks should be underpinned by programme theories. We hope our findings will inspire further conversation and exploration of what to measure, when and with whom, and provide guidance to researchers, service providers and funders striving to strike the balance between reaching a shared consensus, and selecting outcomes according to important contextual factors such as the (often multiple and complex) goals of the intervention, the timing of evaluation, and the study population, for example. This work should be carried out as a collaboration between people who have experienced abuse and their families, service providers and, in the UK, commissioners, to ensure researchers and evaluators are measuring the outcomes that matter most to the people who matter most. Such approaches have previously been used to develop outcome frameworks in related areas [56]. This work should also consider whether the outcomes and outcome measures most appropriate and relevant vary by different characteristics or populations, such as male victim-survivors, BME populations, or those from the LGBT+ community. Any outcome frameworks that are developed should consider this and be adaptable to the different target populations of various services.

For peer review only - http://bmjopen.bmj.com/site/about/guidelines.xhtml

Implications and recommendations for service providers

Whilst we recognise that service providers working in the DSVA field are often over-burdened and under-resourced, and doing the best they can with what they have, we would encourage service providers to use the findings from this review to consider their current practices for outcome measurement and the how's and why's underlying them. If resource allows, organisations could assess whether the outcome measurement tools currently being used are fit for purpose and reflect recent shifts in definitions of and responses to domestic abuse, and explore opportunities for improvement and innovation. In the absence of additional resource, service providers should continue to record and monitor the various service- and individual-level outcomes they are required to measure as accurately and consistently as is possible whilst delivering frontline services.

Implications and recommendations for policymakers

Ultimately, the extent to which service providers can apply research findings to practice and use them to drive improvements to services depends heavily upon policymakers' decisions relating to the provision and funding of services, and monitoring and evaluation. Currently, patchwork and piecemeal funding [36, 196] and inconsistent commissioning frameworks contribute to the diversity of outcomes measured and underpin a competitive funding landscape that is not conducive to achieving consensus across the sector. Service providers working to end DSVA have been campaigning for greater consistency in the commissioning of services for many years, and their voices should be used by policymakers to enact meaningful change. Based on discussions with our advisory group throughout the review process, we would suggest that policymakers focus on ensuring any core outcomes framework is implementable on a practical level. Service providers in low resource settings likely have less scope for this than others, and it is crucial that any core outcome measurement tools are not too time-consuming for service providers to use and complete. Additionally, policymakers and commissioners should look beyond blunt, immediately measurable outcomes (e.g. immediate safety, prosecution and conviction rates) and

For peer review only - http://bmjopen.bmj.com/site/about/guidelines.xhtml

recognise the value of other outcomes (e.g. social and emotional wellbeing), which may take longer to measure but be a more accurate indication of real change. A suggested avenue for working towards this would be to run a national survivor consultation to draw out what is the most meaningful and/or has made the most difference for survivors. Key findings from this could then be mapped onto the outcomes prioritised by policymakers, service providers and funders, to identify where there are overlaps and where there are disparities that need addressing.

Conclusions

A broad range of outcome measures have been used to assess support interventions for people who have experienced or perpetrated DSVA. There is growing recognition across various sectors of the potential benefits of a core set of outcomes, which include increasing consistency and consensus [197, 198]. We need to take stock of the current state of the evidence and reconceptualise what a core set of outcomes should look like across and within sectors. This core outcome set needs to be co-produced by a range of stakeholders, including those with experience of DSVA and those providing front line support. For this to be feasible a trade-off needs to occur. Burden needs to be minimised by not requiring excessive data collection, but instead, collection of only the most relevant outcome measures according to the specific purpose of the intervention or service. At the same time, within-service consistency needs to be increased, as well as supporting comparability across sectors. Further research is needed to better understand which outcomes should be prioritised in order to strike this balance.

For peer review only

Figure legends:

Figure 1. PRISMA flow diagram

Figure 2. Bar chart to show the percentage of included studies with each categorisation type.

Figure 3. Venn diagram of outcome categories, domains and subdomains

Ethical approval statement: Not applicable as this was a scoping review.

For peer review only

For peer review only - http://bmjopen.bmj.com/site/about/guidelines.xhtml

References

1. UK Faculty of Public Health, *The role of public health in the prevention of violence*. 2016.
2. Savage-Borne, J. and A. Maitra. *Intimate partner violence as a public health problem*. 2021; Available from: https://blogs.bmj.com/bmj/2021/03/25/intimate-partner-violence-as-a-public-health-problem/.
3. World Health Organization. *Voilence against women*. 2021; Available from: https://www.who.int/news-room/fact-sheets/detail/violence-against-women.
4. Office for National Statistics (ONS), *Domestic abuse prevalence and victim characteristics Year ending March 2022*, Office for National Statistics (ONS), Editor. 2022.
5. World Health Organization, *Global and regional estimates of violence against women: prevalence and health effects of intimate partner violence and non-partner sexual violence*. 2013: World Health Organization.
6. Femi-Ajao, O., S. Kendal, and K. Lovell, *A qualitative systematic review of published work on disclosure and help-seeking for domestic violence and abuse among women from ethnic minority populations in the UK.* Ethnicity & Health, 2020. **25**(5): p. 732-746.
7. AVA, A., *Breaking down the barriers: findings of the national commission on domestic and sexual violence and multiple disadvantage*. 2020.
8. Donovan, C., et al., *Comparing domestic abuse in same sex and heterosexual relationships.* Unpublished manuscript, University of Sunderland, Sunderland, England, and University of Bristol, Bristol, England, 2006.
9. Roch, A., G. Ritchie, and J. Morton, *Transgender people's experiences of domestic abuse.* Scotland: LGBT Youth Scotland and the Equality Network, 2010.
10. Valentine, S.E., et al., *Disparities in exposure to intimate partner violence among transgender/gender nonconforming and sexual minority primary care patients.* LGBT health, 2017. **4**(4): p. 260-267.
11. Langenderfer-Magruder, L., et al., *Experiences of intimate partner violence and subsequent police reporting among lesbian, gay, bisexual, transgender, and queer adults in Colorado: Comparing rates of cisgender and transgender victimization.* Journal of interpersonal violence, 2016. **31**(5): p. 855-871.
12. Khalifeh, H., et al., *Violence against people with disability in England and Wales: findings from a national cross-sectional survey.* PloS one, 2013. **8**(2): p. e55952.
13. Trevillion, K., et al., *Experiences of domestic violence and mental disorders: a systematic review and meta-analysis.* PloS one, 2012. **7**(12): p. e51740.
14. Fulu, E., et al., *Prevalence of and factors associated with male perpetration of intimate partner violence: findings from the UN Multi-country Cross-sectional Study on Men and Violence in Asia and the Pacific.* The lancet global health, 2013. **1**(4): p. e187-e207.
15. Herbert, A., et al., *Risk factors for intimate partner violence and abuse among adolescents and young adults: findings from a UK population-based cohort.* Wellcome open research, 2020. **5**.
16. Hester, M., *Who does what to whom?: Gender and domestic violence perpetrators*. 2009: University of Bristol [in association with] Northern Rock Foundation Bristol.
17. Robinson, A.L., *Serial domestic abuse in Wales: an exploratory study into its definition, prevalence, correlates, and management.* Victims & Offenders, 2017. **12**(5): p. 643-662.
18. Gilchrist, G., et al., *The prevalence and factors associated with ever perpetrating intimate partner violence by men receiving substance use treatment in Brazil and England: A cross-cultural comparison.* Drug and Alcohol Review, 2017. **36**(1): p. 34-51.
19. Basile, K.C., et al., *National intimate partner and sexual violence survey: 2010 summary report.* 2011.

For peer review only - http://bmjopen.bmj.com/site/about/guidelines.xhtml

20. Breiding, M.J., M.C. Black, and G.W. Ryan, *Chronic disease and health risk behaviors associated with intimate partner violence—18 US states/territories, 2005.* Annals of epidemiology, 2008. **18**(7): p. 538-544.
21. Chandan, J.S., et al., *Risk of cardiometabolic disease and all-cause mortality in female survivors of domestic abuse.* Journal of the American Heart Association, 2020. **9**(4): p. e014580.
22. Crofford, L.J., *Violence, stress, and somatic syndromes.* Trauma, Violence, & Abuse, 2007. **8**(3): p. 299-313.
23. John, R., et al., *Domestic violence: prevalence and association with gynaecological symptoms.* BJOG: An International Journal of Obstetrics & Gynaecology, 2004. **111**(10): p. 1128-1132.
24. Leserman, J. and D.A. Drossman, *Relationship of abuse history to functional gastrointestinal disorders and symptoms: some possible mediating mechanisms.* Trauma, Violence, & Abuse, 2007. **8**(3): p. 331-343.
25. Tjaden, P. and N. Thoennes, *Prevalence and consequences of male-to-female and female-to-male intimate partner violence as measured by the National Violence Against Women Survey.* Violence against women, 2000. **6**(2): p. 142-161.
26. McManus, S., et al., *Receiving threatening or obscene messages from a partner and mental health, self-harm and suicidality: results from the Adult Psychiatric Morbidity Survey.* Social psychiatry and psychiatric epidemiology, 2021: p. 1-11.
27. Povey, D., et al., *Homicides, Firearm Offences and Intimate Violence 2006/07 (Supplementary Volume 2 to Crime in England and Wales 2006/07) Home Office Statistical Bulletin 03/08 [online]. London: Home Office Research Development and Statistics Directorate*. 2008.
28. McManus, S., et al., *Intimate Partner Violence, Suicidality, and Self-Harm: A Probability Sample Survey of the General Population in England.* Suicidality, and Self-Harm: A Probability Sample Survey of the General Population in England, 2022.
29. Jonas, S., et al., *Gender differences in intimate partner violence and psychiatric disorders in England: results from the 2007 adult psychiatric morbidity survey.* Epidemiology and psychiatric sciences, 2014. **23**(2): p. 189-199.
30. St. Mungo's, *Rebuilding Shattered Lives [online]*. 2014.
31. Women's Aid, *The Domestic Abuse Report 2019: The Economics of Abuse Report Summary*. 2019.
32. Crown Prosecution Service, *Violence against Women and Girls Report 10th Edition 2016-17*. 2017.
33. Office for National Statistics (ONS), *Domestic abuse and the criminal justice system*. 2021.
34. ONS, *Domestic abuse in England and Wales overview: November 2021. Figures on domestic abuse from police recorded crime and a number of different organisations.* 2021.
35. Thiara, R.K., G. Hague, and A. Mullender, *Losing out on both counts: disabled women and domestic violence.* Disability & Society, 2011. **26**(6): p. 757-771.
36. Imkaan, *From Survival to Sustainability: Critical Issues for the Specialist Black and 'Minority Ethnic'Ending Violence Against Women and Girls Sector in the UK*. 2018, Imkaan London.
37. Colgan, F., C. Hunter, and A. McKearney, *'Staying alive': The impact of 'austerity cuts' on the LGBT Voluntary and Community Sector (VCS) in England and Wales*. 2014, TUC.
38. Heise, L.L., *Violence against women: An integrated, ecological framework.* Violence against women, 1998. **4**(3): p. 262-290.
39. Sullivan, C.M., *Understanding how domestic violence support services promote survivor well-being: A conceptual model.* Journal of family violence, 2018. **33**(2): p. 123-131.
40. Hobfoll, S., *Stress[illegible]culture[illegible]and community: the psychology and philosophy of stress*. 1998, New York: Plenum Press[illegible].
41. Hobfoll, S.E., *Conservation of resources: A new attempt at conceptualizing stress.* American psychologist, 1989. **44**(3): p. 513.

For peer review only - http://bmjopen.bmj.com/site/about/guidelines.xhtml

42. Hobfoll, S.E., *The influence of culture, community, and the nested-self in the stress process: Advancing conservation of resources theory.* Applied psychology, 2001. **50**(3): p. 337-421.
43. Yakubovich, A.R., et al., *Housing interventions for women experiencing intimate partner violence: a systematic review.* The Lancet Public Health, 2021.
44. Kiani, Z., et al., *A systematic review: Empowerment interventions to reduce domestic violence?* Aggression and violent behavior, 2021. **58**: p. 101585.
45. Rivas, C., et al., *Advocacy interventions to reduce or eliminate violence and promote the physical and psychosocial well-being of women who experience intimate partner abuse: A systematic review.* Campbell Systematic Reviews, 2016. **12**(1): p. 1-202.
46. Hameed, M., et al., *Psychological therapies for women who experience intimate partner violence.* Cochrane database of systematic reviews, 2020(7).
47. Advance, *Annual Report and Accounts*. 2021.
48. Refuge, *Annual Report 2020–2021 and financial statements*. 2021.
49. Yellow Door, *Annual Review and Accounts 2020-2021*. 2021.
50. Oasis, *Impact*. 2019.
51. Holly Taylor-Dunn and Rosie Erol, *Evaluation of SaferPlaces' Independent Domestic Violence Advisor Services.* 2019.
52. Lilley-Walker, S.-J., M. Hester, and W. Turner, *Evaluation of European domestic violence perpetrator programmes: Toward a model for designing and reporting evaluations related to perpetrator treatment interventions.* International journal of offender therapy and comparative criminology, 2018. **62**(4): p. 868-884.
53. Akoensi, T.D., et al., *Domestic violence perpetrator programs in Europe, Part II: A systematic review of the state of evidence.* International journal of offender therapy and comparative criminology, 2013. **57**(10): p. 1206-1225.
54. National Institute for Health and Care Excellence. *Domestic violence and abuse: multi-agency working: Public health guideline [PH50]. Recommendations for research*. 2014; Available from: https://www.nice.org.uk/guidance/ph50/chapter/5-Recommendations-for-research.
55. James Lind Alliance. *Sexual Violence Top 10*. 2022; Available from: https://www.jla.nihr.ac.uk/priority-setting-partnerships/sexual-violence/top-10-priorities.htm.
56. Powell, C., et al., *Development of core outcome sets for family and child-focused interventions for child maltreatment and domestic violence*. 2021.
57. Sprague, S., et al., *Outcome measures for evaluating intimate partner violence programs within clinical settings: A systematic review of the literature.* Trauma, Violence, & Abuse, 2017. **18**(5): p. 508-522.
58. Clark, S.E., et al., *Engaging domestic abuse practitioners and survivors in a review of outcome tools–reflections on differing priorities.* Research for All, 2023. **7**(1).
59. Sullivan, C.M., *Evaluating domestic violence support service programs: Waste of time, necessary evil, or opportunity for growth?* Aggression and Violent Behavior, 2011. **16**(4): p. 354-360.
60. Grych, J. and S. Hamby, *Advancing the measurement of violence: Challenges and opportunities.* Psychology of Violence, 2014. **4**(4): p. 363.
61. Munn, Z., et al., *Systematic review or scoping review? Guidance for authors when choosing between a systematic or scoping review approach.* BMC medical research methodology, 2018. **18**: p. 1-7.
62. Peterson, J., et al., *Understanding scoping reviews: Definition, purpose, and process.* Journal of the American Association of Nurse Practitioners, 2017. **29**(1): p. 12-16.
63. Tricco, A.C., et al., *PRISMA extension for scoping reviews (PRISMA-ScR): checklist and explanation.* Annals of internal medicine, 2018. **169**(7): p. 467-473.

For peer review only - http://bmjopen.bmj.com/site/about/guidelines.xhtml

64. Carlisle, S., et al. *Measuring the effectiveness of UK-based support interventions and/or services targeted at adults who have experienced domestic and sexual violence and abuse (DSVA): A scoping review*. 2022; Available from: https://osf.io/frh2e.
65. Home Office, *Policy Paper: Tackling perpetrators*. 2022.
66. Ouzzani, M., et al., *Rayyan — a web and mobile app for systematic reviews.* Systematic Reviews, 2016. **5**(210).
67. Beck, A.T., R.A. Steer, and G.K. Brown, *Beck depression inventory*. 1987: Harcourt Brace Jovanovich New York:.
68. ADVA. and S.P. Associates., *REPAIR (Resolve to End the Perpetration of Abuse in Relationships): A Community- and Whole-family-based Intervention Programme Targeting Perpetrators of Domestic Violence and Abuse in Devon. An evaluation of a three-year Invest to Save (ISB) PROJECT*. 2009: Exeter.
69. Advance, *'A Place to go Like This': Breaking the cycle of harm for mothers involved in offending who are survivors of domestic abuse, and their children.* 2020.
70. Barber, M., R. Jenkins, and C. Jones, *A survivor's group for women who have a learning disability.* British Journal of Developmental Disabilities, 2000. **46**(90, Pt 1): p. 31-41.
71. Bloomfield, S. and L. Dixon, *An outcome evaluation of the integrated domestic abuse programme (IDAP) and community domestic violence programme (CDVP).* London: National Offender Management Service, 2015.
72. Bowen, E., *Evaluation of a community based domestic violence offender rehabilitation programme. Unpublished doctoral thesis*, in *Centre for Forensic and Family Psychology*. 2004, University of Birmingham.
73. Bowen, E., *Therapeutic Environment and Outcomes in a UK Domestic Violence Perpetrator Program.* Small Group Research, 2010. **41**(2): p. 198-220.
74. Bowen, E. and E. Gilchrist, *Predicting dropout of court-mandated treatment in a British sample of domestic violence offenders.* Psychology, Crime & Law, 2006. **12**(5): p. 573-587.
75. Bowen, E., E. Gilchrist, and A.R. Beech, *Change in treatment has no relationship with subsequent re-offending in UK domestic violence sample: A preliminary study.* International Journal of Offender Therapy and Comparative Criminology, 2008. **52**(5): p. 598-614.
76. Bowen, E., E.A. Gilchrist, and A.R. Beech, *An Examination of the Impact of Community-Based Rehabilitation on the Offending Behaviour of Male Domestic Violence Offenders and the Characteristics Associated with Recidivism.* Legal and Criminological Psychology, 2005. **10**(2): p. 189-209.
77. Brady, F., et al., *Narrative exposure therapy for survivors of human trafficking: feasibility randomised controlled trial.* BJPsych Open, 2021. **7**(6).
78. Burton, S., L. Regan, and L. Kelly, *Supporting women and challenging men: lessons from the Domestic Violence Intervention Project.* 1998.
79. Callaghan, J., et al., *Make a Change: An evaluation of the implementation of an early response intervention for those who have used abusive behaviours in their intimate relationships*. 2020.
80. Respect and Women's Aid Federation England, *Make a Change Executive Summary*.
81. Calvert, R., S. Kellett, and T. Hagan, *Group cognitive analytic therapy for female survivors of childhood sexual abuse.* British Journal of Clinical Psychology, 2015. **54**(4): p. 391-413.
82. Clarke, S. and S. Llewelyn, *Personal constructs of survivors of childhood sexual abuse receiving cognitive analytic therapy.* British Journal of Medical Psychology, 1994. **67(3)**: p. 273-289.
83. Clarke, S. and C. Pearson, *Personal constructs of male survivors of childhood sexual abuse receiving cognitive analytic therapy.* British Journal of Medical Psychology, 2000. **73(2)**: p. 169-177.
84. Cook, D.A., et al., *The Berkeley group: ten years' experience of a group for non-violent sex offenders.* The British Journal of Psychiatry, 1991. **158**(2): p. 238-243.

For peer review only - http://bmjopen.bmj.com/site/about/guidelines.xhtml

85. Co-Ordinated Action Against Domestic, A., *Insights into domestic abuse 1: a place of greater safety.* 2012.
86. Co-ordinated Action Against Domestic Abuse (CAADA), *Insights National Dataset 2011–12 Appendix to: A place of greater safety*. 2012.
87. Coy, M. and L. Kelly, *Islands in the stream: an evaluation of four London independent domestic violence advocacy schemes*. 2011.
88. Dheensa, S., G. Penny, and Safelink, *"In my way…" Evaluation Of Learning Disabilities Sexual Abuse Support Services*.
89. Dobash, R.P., et al., *A research evaluation of British programmes for violent men.* Journal of Social Policy, 1999. **28**(2): p. 205-233.
90. Ellis, F., *Rehabilitation programme for adult survivors of childhood sexual abuse.* Journal of public mental health, 2012. **11**(2): p. 88-92.
91. Farrell, G. and A. Buckley, *Evaluation of a UK police domestic violence unit using repeat victimisation as a performance indicator.* The Howard Journal of Criminal Justice, 1999. **38**(1): p. 42-53.
92. Ferrari, G., et al., *Psychological advocacy towards healing (PATH): A randomized controlled trial of a psychological intervention in a domestic violence service setting.* Plos One, 2018. **13**(11).
93. Friendship, C., R.E. Mann, and A.R. Beech, *Evaluation of a national prison-based treatment program for sexual offenders in England and Wales.* Journal of interpersonal violence, 2003. **18**(7): p. 744-759.
94. Garry, K. and P. Goodwin, *Adult Counselling Service Phase two evaluation report: year 1 2020-21*. 2021, Merida Associates.
95. Karen Garry and Ellie Munro, *Adult Counselling Service Interim evaluation | May 2019*. 2019.
96. Karen Garry and Ellie Munro, *Adult Counselling Service Evaluation report – phase one 2016-2020*. 2020.
97. Geoghegan-Fittall, S., C. Keeble, and D. Wunsch, *The Croydon Drive Project: A 2-year Evaluation Final Report*. 2020.
98. Gilchrist, E., et al., *Using the Behaviour Change Wheel to design an intervention for partner abusive men in drug and alcohol treatment.* Pilot and feasibility studies, 2021. **7**(1): p. 1-14.
99. Gilchrist, G., et al., *ADVANCE integrated group intervention to address both substance use and intimate partner abuse perpetration by men in substance use treatment: a feasibility randomised controlled trial.* BMC public health, 2021. **21**(1): p. 1-20.
100. Goosey, J., L. Sherman, and P. Neyroud, *Integrated case management of repeated intimate partner violence: A randomized, controlled trial.* Cambridge Journal of Evidence-Based Policing, 2017. **1**(2): p. 174-189.
101. Halliwell, G., et al., *Cry for health: a quantitative evaluation of a hospital-based advocacy intervention for domestic violence and abuse.* Bmc Health Services Research, 2019. **19**(1).
102. Hester, M., et al., *Evaluation of Year 2 of the Drive Project – A Pilot to Address High Risk Perpetrators of Domestic Abuse*. 2019.
103. Hester, M., et al., *Evaluation of the Drive Project – a pilot to address high-risk perpetrators of domestic abuse Year 1 Feasibility Study*. 2017.
104. Hester, M., et al., *Evaluation of the Drive Project – A Three-year Pilot to Address High-risk, High-harm Perpetrators of Domestic Abuse*. 2019.
105. Hester, M., et al., *Evaluation of the Drive Project – A Three-year Pilot to Address High-risk, High-harm Perpetrators of Domestic Abuse EXECUTIVE SUMMARY.* 2020.
106. Hester, M. and N. Westmarland, *Tackling domestic violence: effective interventions and approaches*. 2005: Home Office Research, Development and Statistics Directorate.
107. Howarth, E. and et al., *Safety in numbers: a multi-site evaluation of Independent Domestic Violence Advisor Services.* 2009.

For peer review only - http://bmjopen.bmj.com/site/about/guidelines.xhtml

108. Howarth, E., et al., *Safety in numbers: summary of findings and recommendations from a multi-site evaluation of Independent Domestic Violence Advisors.* 2009.
109. Howarth, E. and A. Robinson, *Responding Effectively to Women Experiencing Severe Abuse: Identifying Key Components of a British Advocacy Intervention.* Violence against Women, 2016. **22**(1): p. 41-63.
110. Karatzias, T., et al., *Group psychotherapy for female adult survivors of interpersonal psychological trauma: a preliminary study in Scotland.* Journal of Mental Health, 2016. **25**(6): p. 512-519.
111. Kelley, T.M., et al., *The Efficacy of Principle-Based Correctional Counseling for Improving the Self-Control and Mental Health of People Incarcerated for Sexual Violence.* Violence Against Women, 2022. **28**(2): p. 573-592.
112. Kelly, L., *Domestic Violence Matters: an evaluation of a development project*. 1999.
113. Kelly, L. and N. Westmarland, *DOMESTIC VIOLENCE PERPETRATOR PROGRAMMES STEPS TOWARDS CHANGE: Project Mirabal Final Report*. 2015.
114. Koppensteiner, M.F., J. Matheson, and R. Plugor, *Project 360: An intervention to address victim-police engagement in repeat domestic violence cases*. 2017.
115. Lindsay, W.R., et al., *Comparing offenders against women and offenders against children on treatment outcome in offenders with intellectual disability.* Journal of Applied Research in Intellectual Disabilities, 2011. **24**(4): p. 361-369.
116. Lindsay, W.R., et al., *The treatment of six men with a learning disability convicted of sex offences with children.* British Journal of Clinical Psychology, 1998. **37**: p. 83-98.
117. Lowe, M., et al., *CORE assessment of adult survivors abused as children: A NAPAC group therapy evaluation.* Counselling & Psychotherapy Research, 2017. **17**(1): p. 71-79.
118. McConnell, N., et al., *Caring dads safer children*. 2014.
119. McConnell, N., M. Barnard, and J. Taylor, *Caring Dads Safer Children: Families' Perspectives on an Intervention for Maltreating Fathers.* Psychology of Violence, 2017. **7**(3): p. 406-416.
120. McCracken, K. and T. Deave, *Evaluation of the Caring Dads Cymru programme. Final report.* 2012.
121. Michie, A.M. and W.R. Lindsay, *A treatment component designed to enhance empathy in sex offenders with an intellectual disability.* The British Journal of Forensic Practice, 2012. **14**(1): p. 40-48.
122. Morgan, S. and J. Parkes, *The Hampshire Domestic Abuse Prevention Partnership 2016-2018: process and outcomes evaluation.* 2018.
123. Morgan, S.A., B.M.S. McCausland, and J. Parkes, *Baseline characteristics and outcomes of the main perpetrator programme within the Hampshire Domestic Abuse Prevention Partnership, UK: A mixed methods study.* Plos One, 2019. **14**(7).
124. Morrison, A. and L. Treliving, *Evaluation of outcome in a dynamically orientated group for adult males who have been sexually abused in childhood.* British Journal of Psychotherapy, 2002. **19**: p. 59-75.
125. Murphy, G., et al., *Cognitive-behavioural treatment for men with intellectual disabilities and sexually abusive behaviour: A pilot study.* Journal of Intellectual Disability Research, 2007. **51(11)**: p. 902-912.
126. Oasis. *Impact report 2013-2014*. 2014; Available from: https://issuu.com/kristinagwynne/docs/oasis_annual_report_2013-2014-web/1.
127. Oasis, *Impact Report 2015-2016.* 2016.
128. Ormston, R., C. Mullholland, and L. Setterfield, *Caledonian system evaluation: analysis of a programme for tackling domestic abuse in Scotland*. 2016, Edinburgh: Scotland. Scottish Government Social Research. vi, 88.
129. Payne, A., H. Liebling-Kalifani, and S. Joseph, *Client-centred group therapy for survivors of interpersonal trauma: A pilot investigation.* Counselling & Psychotherapy Research, 2007. **7**(2): p. 100-105.

For peer review only - http://bmjopen.bmj.com/site/about/guidelines.xhtml

130. Peckham, N.G., S. Howlett, and A. Corbett, *Evaluating a survivors group pilot for a women with significant intellectual disabilities who have been sexually abused.* Journal of Applied Research in Intellectual Disabilities, 2007. **20**(4): p. 308-322.
131. Robinson, A.L., *The Cardiff Women's Safety Unit: A multi-agency approach to domestic violence.* Final Evaluation Report. Cardiff University: School of Social Sciences.(Available online at http://www. cf. ac. uk/socsi/whoswho/robinson. html), 2003.
132. Robinson, A.L., *Domestic Violence MARACs (Multi-Agency Risk Assesment Conferences) for Very High-risk Victims in Cardiff, Wales: A Process and Outcome Evaluation.* 2004.
133. Robinson, A.L., *Reducing repeat victimization among high-risk victims of domestic violence: The benefits of a coordinated community response in Cardiff, Wales.* Violence Against Women, 2006. **12(8)**: p. 761-788.
134. Robjant, K., J. Roberts, and C. Katona, *Treating posttraumatic stress disorder in female victims of trafficking using narrative exposure therapy: A retrospective audit.* Frontiers in Psychiatry, 2017. **8**.
135. Rose, J., et al., *A sex offender treatment group for men with intellectual disabilities in a community setting.* The British Journal of Forensic Practice, 2012.
136. Ross, J., J. Sebire, and H. Strang, *Tracking Repeat Victimisation After Domestic Abuse Cases Are Heard With and Without Independent Domestic Violence Advisors (IDVAs) in an English Magistrate's Court.* Cambridge Journal of Evidence-Based Policing, 2022: p. 1-15.
137. Ryan, M., et al., *A prospective study of the effectiveness of group and individual psychotherapy for women CSA survivors.* Psychology and Psychotherapy: Theory, Research and Practice, 2005. **78(4)**: p. 465-480.
138. SafeLives, *Insights refuge England and Wales dataset 2015-18 Adult refuge services*. 2018.
139. SafeLives, *Defining Marac Outcomes National Pilot*. 2012.
140. SafeLives, *Insights England and Wales dataset 2018-19 Sexual violence services* 2019.
141. SafeLives, *Insights SV dataset 2020-21 Sexual violence (SV) services*. 2021.
142. SafeLives, *Insights health England and Wales dataset 2015-2018 Adults health-based services*. 2018.
143. SafeLives, *Insights helpline England and Wales dataset 2014-17 Adult helpline services*. 2017.
144. SafeLives, *Insights Idva national dataset 2012–13 Adult independent domestic violence advisor (Idva) services*. 2015.
145. SafeLives, *Insights Idva national dataset 2013–14 Adult independent domestic violence advisor (Idva) services*. 2015.
146. SafeLives, *Insights Idva England and Wales dataset 2016-17 Adult independent domestic violence advisor (Idva) services*. 2017.
147. SafeLives, *Insights Idva England and Wales dataset 2018-19 Adult Independent domestic violence advisor (Idva) services*. 2019.
148. SafeLives, *Insights Idva dataset 2020-21 Adult Independent domestic violence advisor (Idva) services*. 2021.
149. SafeLives, *Insights outreach national dataset 2012–13 Adult outreach services*. 2015.
150. SafeLives, *Insights outreach national dataset 2013–14 Adult outreach services.* 2015.
151. SafeLives, *Insights outreach England and Wales dataset 2016-17 Adult outreach services*. 2017.
152. SafeLives, *Insights outreach England and Wales dataset 2018-19 Adult outreach services*. 2019.
153. SafeLives, *Insights outreach dataset 2020-21 Adult outreach services*. 2021.
154. Sharpe, J., et al., *Group analytic therapy for male survivors of childhood sexual abuse.* Group Analysis, 2001. **34**: p. 195-209.
155. Skyner, D.R. and J. Waters, *Working with perpetrators of domestic violence to protect women and children: a partnership between Cheshire Probation Service and the NSPCC.* Child

For peer review only - http://bmjopen.bmj.com/site/about/guidelines.xhtml

Abuse Review: Journal of the British Association for the Study and Prevention of Child Abuse and Neglect, 1999. **8**(1): p. 46-54.
156. Smith, D., et al., *Adults with a history of child sexual abuse: Evaluation of a pilot therapy service.* British Medical Journal, 1995. **310(6988)**: p. 1175-1178.
157. Smith, E., et al., *Strengthening the mother-child relationship following domestic abuse: Service evaluation.* Child Abuse Review, 2015. **24**(4): p. 261-273.
158. Stanley, N., et al., *An evaluation of a new initiative for male perpetrators of domestic violence* 2011.
159. Stanley, N. and a. et, *Roadmap evaluation: final report - VOICES*. 2021, Bristol: Women's Aid SafeLives. 279.
160. Stanley, N. and a. et, *Roadmap evaluation: final report - SLCDPs*. 2021, Bristol: Women's Aid SafeLives. 279.
161. Strang, H., et al., *Reducing the harm of intimate partner violence: Randomized controlled trial of the Hampshire Constabulary CARA Experiment.* Cambridge Journal of Evidence-Based Policing, 2017. **1**(2): p. 160-173.
162. Taylor-Dunn, H. and R. Erol, *Improving the safety and well-being of domestic abuse survivors: The role of a specialist organisation in supporting the work of Independent Domestic Violence Advisors.* Crime Prevention and Community Safety, 2021. **23**(2): p. 115-136.
163. Eve; The Institute for Public Safety, C.J., *2019*. Evaluation of SHE.
164. Trevillion, K., et al., *Linking abuse and recovery through advocacy: An observational study.* Epidemiology and Psychiatric Sciences, 2014. **23**(1): p. 99-113.
165. Vallely, C., *Evaluation of domestic violence pilot sites at Caerphilly (Gwent) and Croydon 2004/05.* 2005.
166. Webster, R., *Kent and Medway Independent Domestic Violence Advisor Service Impact Evaluation FINAL REPORT*. 2015.
167. Westmarland, N. and S. Alderson, *The health, mental health, and well-being benefits of rape crisis counseling.* Journal of Interpersonal Violence, 2013. **28**(17): p. 3265-3282.
168. Williamson, D. and A. Boyle, *Results of the domestic violence advocacy program at addenbrookes hospital.* Academic Emergency Medicine, 2012. **19**: p. 741.
169. Williamson, E. and M. Hester, *Evaluation of the South Tyneside Domestic Abuse Perpetrator Programme (STDAPP) 2006-2008.* University of Bristol, 2009.
170. Miles, C. and K. Smith, *Nowhere to turn, 2018: findings from the second year of the No Woman Turned away project*. 2018, Bristol: Women's Aid. 48.
171. Women's Aid, *Nowhere To Turn, 2022: Findings from the sixth year of the No Woman Turned Away project*. 2022.
172. Women's Aid, *NOWHERE TO TURN Findings from the first year of the No Woman Turned Away project*. 2017.
173. Women's Aid, *NOWHERE TO TURN Findings from the third year of the No Woman Turned Away project*. 2019.
174. Women's Aid, *NOWHERE TO TURN Findings from the fourth year of the No Woman Turned Away project*. 2020, Women's Aid Federation of England.
175. Women's Aid, *NOWHERE TO TURN 2021 Findings from the fifth year of the No Woman Turned Away project*. 2021.
176. Women's Resource, C., *Women and Girls Network: the WGN empowerment model - redressing negative individual and social impacts of gendered violence through holistic counselling.* 2011.
177. Women's Resource, C., *Ashiana Network: journey towards safety.* 2011.
178. Campbell, J.C. and K.L. Soeken, *Women's responses to battering over time: an analysis of change.* Journal of Interpersonal violence, 1999. **14**(1): p. 21-40.
179. Rosenberg, M., *Rosenberg self-esteem scale.* Journal of Religion and Health, 1965.

For peer review only - http://bmjopen.bmj.com/site/about/guidelines.xhtml

180. Evans, C., et al., *Towards a standardised brief outcome measure: Psychometric properties and utility of the CORE–OM.* The British Journal of Psychiatry, 2002. **180**(1): p. 51-60.
181. Derogatis, L.R., *SCL-90-R: Administration, scoring & procedures manual-II for the (revised) version and other instruments of the psychopathology rating scale series.* Clinical Psychometric Research., 1992: p. 1-16.
182. Kroenke, K., R.L. Spitzer, and J.B. Williams, *The PHQ-9: validity of a brief depression severity measure.* Journal of general internal medicine, 2001. **16**(9): p. 606-613.
183. Spitzer, R.L., et al., *A brief measure for assessing generalized anxiety disorder: the GAD-7.* Archives of internal medicine, 2006. **166**(10): p. 1092-1097.
184. Sherman, L., P.W. Neyroud, and E. Neyroud, *The Cambridge crime harm index: Measuring total harm from crime based on sentencing guidelines.* Policing: a journal of policy and practice, 2016. **10**(3): p. 171-183.
185. Prins, A., et al., *The primary care PTSD screen for DSM-5 (PC-PTSD-5).[measurement instrument].* Universität Bielefeld: Bielefeld, Germany, 2015.
186. Women Against Violence Europe, *WAVE Report 2015: On the Role of Specialist Women's Support Services in Europe*. 2016.
187. Women's Aid. *What we believe: Our values*. 2022; Available from: https://www.womensaid.org.uk/what-we-believe/.
188. Robotham, D., A. Sweeney, and C. Perôt, *Violence, abuse and mental health network: Survivors' priority themes and questions for research—Consultation report, 2019.* Retrieved June, 2019. **26**: p. 2020.
189. Macy, R.J., et al., *Domestic violence and sexual assault services: Historical concerns and contemporary challenges.* Journal of Interpersonal Violence, 2010. **25**(1): p. 3-32.
190. SafeLives, *We only do bones here': Why London needs a whole-health approach to domestic abuse*. 2021, SafeLives: London.
191. Wiley, K., *Implementing domestic violence policy: When accountability trumps mission.* Affilia, 2020. **35**(4): p. 533-551.
192. Women's Aid, *Fragile funding landscape: The extent of local authority commissioning in the domestic abuse refuge sector in England 2020*. 2021, Women's Aid: Bristol.
193. Towers, J. and S. Walby, *Measuring the impact of cuts in public expenditure on the provision of services to prevent violence against women and girls.* 2012.
194. Corry, D., *Hearing women's voices: Why women 2018.* . 2018.
195. Heady, L., A. Kail, and C. Yeowart, *Understanding the stability and sustainability of the violence against women voluntary sector.* 2011.
196. Domestic Abuse Commissioner, *Early Findings from the Domestic Abuse Commissioner's Mapping of Domestic Abuse Services across England & Wales*. 2022.
197. Sullivan, R. and E. Sharples, *The CSA Centre's Evaluation Fund: A Reflection*. 2018, Barkingside: Centre of expertise on child sexual abuse.
198. Home Office, *Violence against women and girls services: commissioning toolkit*. 2022, Home Office: London.

For peer review only - http://bmjopen.bmj.com/site/about/guidelines.xhtml

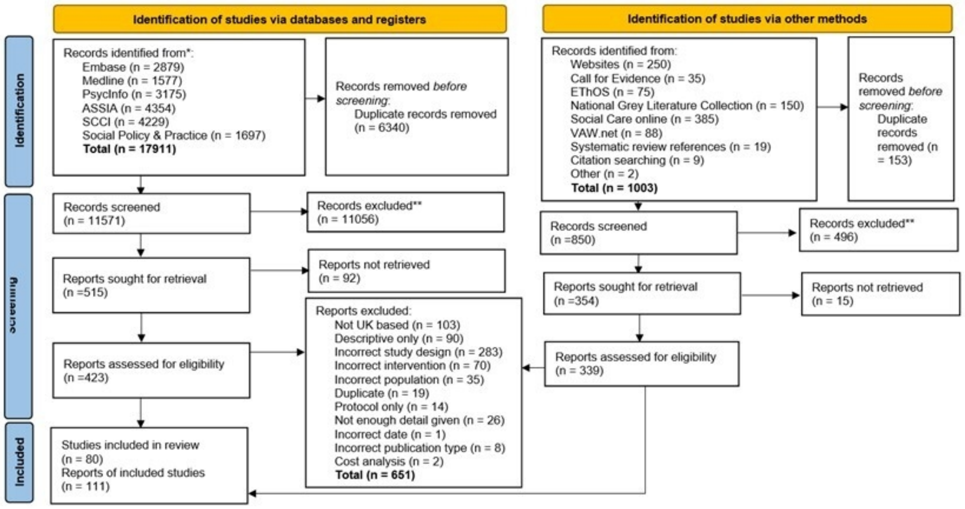

PRISMA flow diagram

35x18mm (600 x 600 DPI)

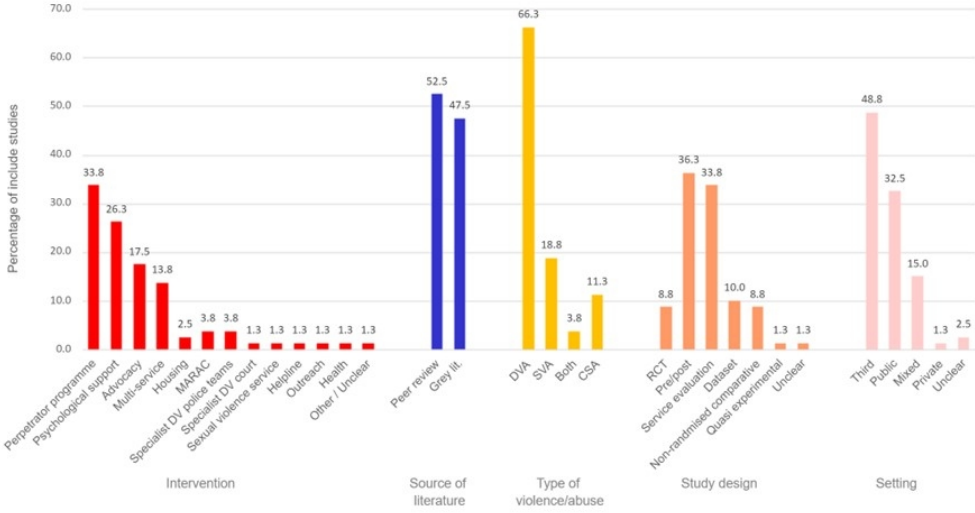

Bar chart to show the percentage of included studies with each categorisation type.

235x129mm (96 x 96 DPI)

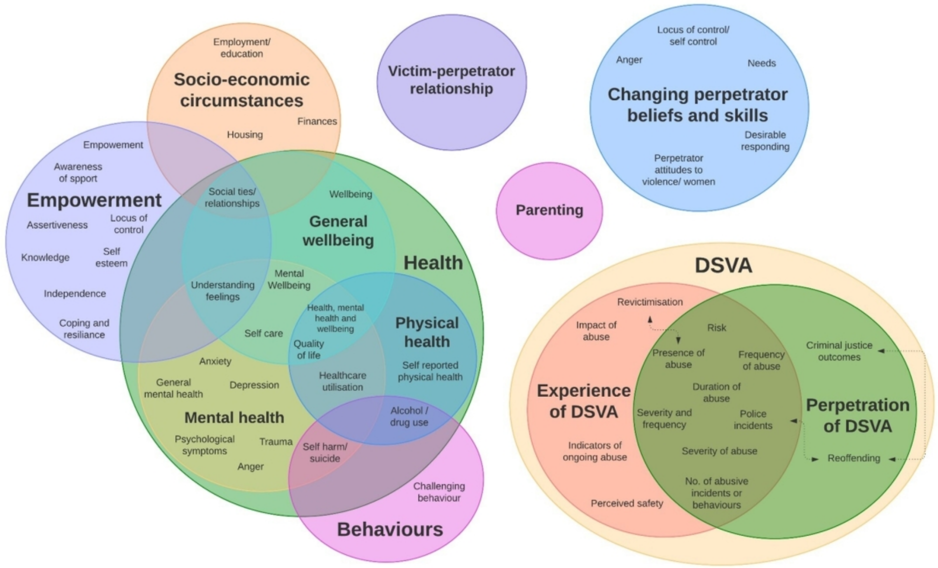

Venn diagram of outcome categories, domains and subdomains

167x99mm (300 x 300 DPI)

Appendix 1: Glossary

Advocacy: Adapted from the National Institute for Health and Clinical Excellence (NICE) Domestic Violence and Abuse: Multi-agency Working public health guideline (PH50)[1]. In general, advocacy for people who have experienced DSVA includes: legal, housing and financial advice: access to and use of community resources such as refuges, emergency housing and psychological interventions; and safety planning advice. The activities may differ according to the level of risk facing the person. Crisis advocacy involves working with the person for a limited period of time (they may then be referred on to more specialist agencies). Practitioners providing advocacy can also provide ongoing support and informal counselling. The intensity of the advocacy provided may vary. It may last for a year – or longer, if the person is particularly vulnerable.

By-and-for: By-and-for services are lived experience organisation, where all of the staff, senior managers, Board of Trustees, and women and girls using services are Black and minoritised women. Such services have a Black feminist ethos, locating themselves as anti-racist, social justice and intersectional organisations.

Domestic and sexual violence and abuse (DSVA): This review works to the UK government definition of domestic violence and abuse[2], and The Istanbul Convention[3], The World Health Organisation[4], and The Rome Statute of the International Criminal Court's (ICC) Elements of Crimes definitions of sexual violence and abuse[5]. In brief, domestic violence and abuse is defined as behaviour within an intimate relationship or family which includes acts of physical violence, sexual violence, emotional or psychological abuse, and controlling behaviours. This also includes 'honour'-based violence and FGM. Sexual violence and abuse is defined as any non-consensual act of sexual nature, extending to attempts to obtain a sexual act, unwanted sexual comments or advances, or acts to traffic, using force, threat of force or coercion.

Independent domestic violence advisers (IDVAs): Adapted from the NICE PH50 guideline. Also known as independent domestic violence advocates, IDVAs work primarily with people at high risk of domestic violence and abuse, independently of any one agency, to secure their safety and the safety of their children. Serving as the primary point of contact, IDVAs normally work with their clients from the point of crisis to assess the level of risk, discuss the options and develop plans that address their immediate safety, as well as longer-term solutions. In many areas they are funded by the local community safety partnership, in some areas they are funded by the police or local authorities.

Intervention: Adapted from the NICE Glossary. A treatment, service, project, or programme.

Intimate partner violence (IPV): Adapted from the World Health Organisation's definition. Intimate partner violence refers to behaviour within an intimate relationship that causes physical, sexual or psychological harm, including acts of physical aggression, sexual coercion, psychological abuse and controlling behaviours. This definition covers violence by both current and former spouses and partners.

Organisation: In the context of this review, organisations refer exclusively to those that carry out activities relating to DSVA. Organisations may include both specialist service providers, and organisations that do not directly provider services (such as women's rights organisations that may focus on activities such as campaigning, research, supporting specialist services, training, and education, for issues relating to DSVA, for example).

For peer review only

People who have experienced DSVA: Throughout this review, people who experience DSVA refers to those who are victims or survivors of DSVA.

Perpetrator programmes: Any group or individual intervention for people who have perpetrated DSVA, with the aim of reducing the incidence of violence by changing attitudes, behaviours and/or beliefs of perpetrators.

Primary, secondary, and tertiary prevention: Primary prevention refers to any action, strategy or policy that works to stop domestic violence from occurring in the first place. Primary prevention seeks to reduce the overall likelihood that anyone will become a victim or perpetrator of domestic violence by creating conditions that make violence less likely to occur. Secondary prevention refers to intervening and responding to violence that has already occurred in order to stop violence from happening again. Secondary prevention activities can include refuge, counselling, and safety planning. Tertiary prevention refers to ongoing support to victims and ongoing accountability for abusers. Tertiary prevention activities address the long-term consequences of domestic violence. They can include support groups and other resources for survivors to help them heal so that they do not experience abuse again. They can also include perpetrator programs for abusers to prevent them from continuing to perpetrate violence in the future.

Refuge or shelter: Adapted from the NICE PH50 guideline. Residential service – a safe house – provided for adults (usually women) and children who are experiencing domestic violence and abuse.

Service provider: Primary, community, including third sector, secondary and tertiary care providers of health and social care services, including prison health services and criminal justice agencies. In the context of DSVA, the service providers are predominantly third sector providers. Third sector service providers may be specialist DSVA providers (i.e., they only provide services relating to DSVA) or non-specialist-DSVA providers (i.e., they provide specialist support services for DSVA as well as support services for other issues). Outside of the third sector, service providers such as the NHS, police and courts may provide some DSVA specific services.

Third sector organisations: In this review the definition of third sector services provided by the National Audit Office has been adopted. 'Third sector organisations' is a term used to describe the range of organisations that are neither public sector nor private sector. It includes voluntary and community organisations (both registered charities and other organisations such as associations, self-help groups and community groups), social enterprises, mutuals and co-operatives. Third sector organisations generally: are independent of government. This is also an important part of the history and culture of the sector; are 'value-driven'. This means they are motivated by the desire to achieve social goals (for example, improving public welfare, the environment or economic well-being) rather than the desire to distribute profit; and reinvest any surpluses generated in the pursuit of their goals. For this reason they are sometimes called 'not-for-profit organisations'. A better term is 'not-for-personal-profit'. In many cases, third sector organisations need to make surpluses (or 'profits') to be financially sustainable.

For peer review only

References

1. National Institute for Health and Care Excellence. *Domestic violence and abuse: multi-agency working: Public health guideline [PH50]. Recommendations for research*. 2014; Available from: https://www.nice.org.uk/guidance/ph50/chapter/5-Recommendations-for-research.
2. Home Office. *Circular 003/2013: new government domestic violence and abuse definition*. 2013; Available from: https://www.gov.uk/government/publications/new-government-domestic-violence-and-abuse-definition/circular-0032013-new-government-domestic-violence-and-abuse-definition.
3. Council of Europe, *Council of Europe Convention on preventing and combating violence against women and domestic violence (CETS No. 210)*. 2011.
4. World Health Organization, *World report on violence and health*. 2002.
5. International Criminal Court, *Elements of Crime*. 2013.

For peer review only

Appendix 2: Preferred Reporting Items for Systematic reviews and Meta-Analyses extension for Scoping Reviews (PRISMA-ScR) Checklist

SECTION	ITEM	PRISMA-ScR CHECKLIST ITEM	REPORTED ON PAGE #
**TITLE**			
Title	1	Identify the report as a scoping review.	1
**ABSTRACT**			
Structured summary	2	Provide a structured summary that includes (as applicable): background, objectives, eligibility criteria, sources of evidence, charting methods, results, and conclusions that relate to the review questions and objectives.	3-4
**INTRODUCTION**			
Rationale	3	Describe the rationale for the review in the context of what is already known. Explain why the review questions/objectives lend themselves to a scoping review approach.	5-6
Objectives	4	Provide an explicit statement of the questions and objectives being addressed with reference to their key elements (e.g., population or participants, concepts, and context) or other relevant key elements used to conceptualize the review questions and/or objectives.	6-7
**METHODS**			
Protocol and registration	5	Indicate whether a review protocol exists; state if and where it can be accessed (e.g., a Web address); and if available, provide registration information, including the registration number.	7
Eligibility criteria	6	Specify characteristics of the sources of evidence used as eligibility criteria (e.g., years considered, language, and publication status), and provide a rationale.	7-8 and Table 1
Information sources*	7	Describe all information sources in the search (e.g., databases with dates of coverage and contact with authors to identify additional sources), as well as the date the most recent search was executed.	9
Search	8	Present the full electronic search strategy for at least 1 database, including any limits used, such that it could be repeated.	Appendix 2
Selection of sources of evidence†	9	State the process for selecting sources of evidence (i.e., screening and eligibility) included in the scoping review.	10
Data charting process‡	10	Describe the methods of charting data from the included sources of evidence (e.g., calibrated forms or forms that have been tested by the team before their use, and whether data charting was done independently or in duplicate) and any processes for obtaining and confirming data from investigators.	10
Data items	11	List and define all variables for which data were sought and any assumptions and simplifications made.	10
Critical appraisal of individual sources of evidence§	12	If done, provide a rationale for conducting a critical appraisal of included sources of evidence; describe the methods used and how this information was used in any data synthesis (if appropriate).	N/A

For peer review only

SECTION	ITEM	PRISMA-ScR CHECKLIST ITEM	REPORTED ON PAGE #
Synthesis of results	13	Describe the methods of handling and summarizing the data that were charted.	10-11
**RESULTS**			
Selection of sources of evidence	14	Give numbers of sources of evidence screened, assessed for eligibility, and included in the review, with reasons for exclusions at each stage, ideally using a flow diagram.	12 and Figure 1
Characteristics of sources of evidence	15	For each source of evidence, present characteristics for which data were charted and provide the citations.	12-16
Critical appraisal within sources of evidence	16	If done, present data on critical appraisal of included sources of evidence (see item 12).	N/A
Results of individual sources of evidence	17	For each included source of evidence, present the relevant data that were charted that relate to the review questions and objectives.	Appendix 4
Synthesis of results	18	Summarize and/or present the charting results as they relate to the review questions and objectives.	17-21
**DISCUSSION**			
Summary of evidence	19	Summarize the main results (including an overview of concepts, themes, and types of evidence available), link to the review questions and objectives, and consider the relevance to key groups.	22-23
Limitations	20	Discuss the limitations of the scoping review process.	24-25
Conclusions	21	Provide a general interpretation of the results with respect to the review questions and objectives, as well as potential implications and/or next steps.	26
**FUNDING**			
Funding	22	Describe sources of funding for the included sources of evidence, as well as sources of funding for the scoping review. Describe the role of the funders of the scoping review.	3-4

JBI = Joanna Briggs Institute; PRISMA-ScR = Preferred Reporting Items for Systematic reviews and Meta-Analyses extension for Scoping Reviews.
* Where *sources of evidence* (see second footnote) are compiled from, such as bibliographic databases, social media platforms, and Web sites.
† A more inclusive/heterogeneous term used to account for the different types of evidence or data sources (e.g., quantitative and/or qualitative research, expert opinion, and policy documents) that may be eligible in a scoping review as opposed to only studies. This is not to be confused with *information sources* (see first footnote).
‡ The frameworks by Arksey and O'Malley (6) and Levac and colleagues (7) and the JBI guidance (4, 5) refer to the process of data extraction in a scoping review as data charting.
§ The process of systematically examining research evidence to assess its validity, results, and relevance before using it to inform a decision. This term is used for items 12 and 19 instead of "risk of bias" (which is more applicable to systematic reviews of interventions) to include and acknowledge the various sources of evidence that may be used in a scoping review (e.g., quantitative and/or qualitative research, expert opinion, and policy document).

From: Tricco AC, Lillie E, Zarin W, O'Brien KK, Colquhoun H, Levac D, et al. PRISMA Extension for Scoping Reviews (PRISMAScR): Checklist and Explanation. Ann Intern Med. 2018;169:467–473. doi: 10.7326/M18-0850.

Appendix 3: Example search strategies

Search strategy development process

Search terms were identified during initial scoping and protocol development stages. An initial list of search terms was drafted based on terms found in the literature and based on search terms used in systematic reviews in related areas. This initial list of terms was shared with the review team and with the Patient and Public Involvement advisory group, who added any additional terms thought to be relevant to the review's research question.

Peer review example: Medline

1. exp United kingdom/
2. (national health service* or nhs*).ti,ab,in.
3. (english not ((published or publication* or translat* or written or language* or speak* or literature or citation*) adj5 english)).ti,ab.
4. (gb or "g.b." or britain* or (british* not "british columbia") or uk or "u.k." or united kingdom* or (england* not "new england") or northern ireland* or northern irish* or scotland* or scottish* or ((wales or "south wales") not "new south wales") or welsh*).ti,ab,jw,in.
5. (bangor or "bangor's" or cardiff or "cardiff's" or newport or "newport's" or st asaph or "st asaph's" or st davids or swansea or "swansea's").ti,ab,in,cq.
6. (bath or "bath's" or ((birmingham not alabama*) or ("birmingham's" not alabama*) or bradford or "bradford's" or brighton or "brighton's" or bristol or "bristol's" or carlisle* or "carlisle's" or (cambridge not (massachusetts* or boston* or harvard*)) or ("cambridge's" not (massachusetts* or boston* or harvard*)) or (canterbury not zealand*) or ("canterbury's" not zealand*) or chelmsford or "chelmsford's" or chester or "chester's" or chichester or "chichester's" or coventry or "coventry's" or derby or "derby's" or (durham not (carolina* or nc)) or ("durham's" not (carolina* or nc)) or ely or "ely's" or exeter or "exeter's" or gloucester or "gloucester's" or hereford or "hereford's" or hull or "hull's" or lancaster or "lancaster's" or leeds* or leicester or "leicester's" or (lincoln not nebraska*) or ("lincoln's" not nebraska*) or (liverpool not (new south wales* or nsw)) or ("liverpool's" not (new south wales* or nsw)) or ((london not (ontario* or ont or toronto*)) or ("london's" not (ontario* or ont or toronto*)) or manchester or "manchester's" or (newcastle not (new south wales* or nsw)) or ("newcastle's" not (new south wales* or nsw)) or norwich or "norwich's" or nottingham or "nottingham's" or oxford or "oxford's" or peterborough or "peterborough's" or plymouth or "plymouth's" or portsmouth or "portsmouth's" or preston or "preston's" or ripon or "ripon's" or salford or "salford's" or salisbury or "salisbury's" or sheffield or "sheffield's" or southampton or "southampton's" or st albans or stoke or "stoke's" or sunderland or "sunderland's" or truro or "truro's" or wakefield or "wakefield's" or wells or westminster or "westminster's" or winchester or "winchester's" or wolverhampton or "wolverhampton's" or (worcester not (massachusetts* or boston* or harvard*)) or ("worcester's" not (massachusetts* or boston* or harvard*)) or (york not ("new york*" or ny or ontario* or ont or toronto*)) or ("york's" not ("new york*" or ny or ontario* or ont or toronto*))))).ti,ab,in.
7. (aberdeen or "aberdeen's" or dundee or "dundee's" or edinburgh or "edinburgh's" or glasgow or "glasgow's" or inverness or (perth not australia*) or ("perth's" not australia*) or stirling or "stirling's").ti,ab,in.
8. (armagh or "armagh's" or belfast or "belfast's" or lisburn or "lisburn's" or londonderry or "londonderry's" or derry or "derry's" or newry or "newry's").ti,ab,in.
9. (exp africa/ or exp americas/ or exp antarctic regions/ or exp arctic regions/ or exp asia/ or exp australia/ or exp oceania/) not (exp United Kingdom/ or europe/)

10. (child adj3 maltreat*).ti,ab,kw.
11. (sexual adj3 harass*).ti,ab,kw.
12. (batter* adj3 (wom#n or wife or wives)).ti,ab,kw.
13. rape*.ti,ab,kw.
14. (beat* adj3 (partner* or spous* or wom#n or wife or wives or m#n or husband*)).ti,ab,kw.
15. (sexual adj3 coercion).ti,ab,kw.
16. female genital mutilation.ti,ab,kw.
17. forced marriage.ti,ab,kw.
18. sex traffic*.ti,ab,kw.
19. revenge porn.ti,ab,kw.
20. controlling behaviour.ti,ab,kw.
21. prostitut*.ti,ab,kw.
22. sex work*.ti,ab,kw.
23. stalking.ti,ab,kw.
24. (drink adj3 spik*).ti,ab,kw.

25. domestic abuse service*.ti,ab,kw.
26. women's project*.ti,ab,kw.
27. women's resource centre.ti,ab,kw.
28. safety unit*.ti,ab,kw.
29. domestic abuse support service*.ti,ab,kw.
30. domestic abuse team*.ti,ab,kw.
31. domestic violence intervention*.ti,ab,kw.
32. women's service*.ti,ab,kw.
33. women's rights organi#ation*.ti,ab,kw.
34. domestic abuse advice and support.ti,ab,kw.
35. women's centre*.ti,ab,kw.
36. domestic abuse project*.ti,ab,kw.
37. domestic and sexual abuse project*.ti,ab,kw.
38. victim care service*.ti,ab,kw.
39. support centre*.ti,ab,kw.
40. women's welfare.ti,ab,kw.
41. speciali* service*.ti,ab,kw.
42. speciali* sexual violence service*.ti,ab,kw.
43. speciali* domestic violence service*.ti,ab,kw.
44. speciali* support.ti,ab,kw.
45. speciali* violence and abuse service*.ti,ab,kw.
46. speciali* women's voluntary sector service*.ti,ab,kw.
47. service* for survivors.ti,ab,kw.
48. victim service*.ti,ab,kw.
49. victim support*.ti,ab,kw.
50. violence against women and girls service*.ti,ab,kw.
51. domestic violence support service*.ti,ab,kw.
52. community based service*.ti,ab,kw.
53. sexual assault referral centre*.ti,ab,kw.
54. rape crisis.ti,ab,kw.
55. third sector.ti,ab,kw.
56. second tier.ti,ab,kw.
57. crisis intervention.ti,ab,kw.
58. independent domestic violence advoca*.ti,ab,kw.
59. IDVA.ti,ab,kw.
60. independent sexual violence advoca*.ti,ab,kw.
61. ISVA.ti,ab,kw.
62. shelter*.ti,ab,kw.
63. refuge.ti,ab,kw.

For peer review only

64. refuges.ti,ab,kw.
65. advoca*.ti,ab,kw.
66. counsel?ing.ti,ab,kw.
67. outreach.ti,ab,kw.
68. helpline*.ti,ab,kw.
69. emergency shelter*.ti,ab,kw.
70. supportive hous*.ti,ab,kw.
71. violence against women service*.ti,ab,kw.
72. recovery service*.ti,ab,kw.
73. speciali* victim service*.ti,ab,kw.
74. independent domestic violence advisor*.ti,ab,kw.
75. independent sexual violence advisor*.ti,ab,kw.
76. resettlement.ti,ab,kw.
77. floating support.ti,ab,kw.
78. drop-in.ti,ab,kw.
79. online chat.ti,ab,kw.
80. support group*.ti,ab,kw.
81. violence advocacy project*.ti,ab,kw.
82. group work program*.ti,ab,kw.
83. information and advice.ti,ab,kw.
84. (dedicated adj4 service*).ti,ab,kw.
85. charit*.ti,ab,kw.
86. independent.ti,ab,kw.
87. voluntary.ti,ab,kw.
88. public sector.ti,ab,kw.
89. specialist.ti,ab,kw.
90. speciali?ed.ti,ab,kw.
91. non-governmental organi#ation*.ti,ab,kw.
92. nonprofit organi#ation*.ti,ab,kw.
93. not-for-profit organi#ation*.ti,ab,kw.
94. by and for service*.ti,ab,kw.
95. by and for organi#ation*.ti,ab,kw.
96. therapy.ti,ab,kw.
97. empowerment.ti,ab,kw.
98. case management.ti,ab,kw.
99. stress management.ti,ab,kw.
100. safety behaviour*.ti,ab,kw.
101. safety strateg*.ti,ab,kw.
102. legal advice.ti,ab,kw.
103. mindfulness based stress reduction.ti,ab,kw.
104. home visit*.ti,ab,kw.
105. sanctuar*.ti,ab,kw.
106. safe house*.ti,ab,kw.
107. flexible fund*.ti,ab,kw.
108. exp battered woman/
109. exp domestic violence/ or exp child abuse/ or exp spouse abuse/ or exp gender-based violence/ or exp intimate partner violence/ or exp physical abuse/ or exp rape/
110. exp partner violence/
111. exp family violence/
112. exp coercion/
113. exp emotional abuse/
114. exp sexual violence/ or exp sexual abuse/ or exp sexual assault/
115. ("City of London" or Bedfordshire or Berkshire or Bristol or Buckinghamshire or Cambridgeshire or Cheshire or Cornwall or Cumbria or Derbyshire or Devon or Dorset or Durham or Yorkshire or Essex or Gloucestershire or "greater london" or "greater manchester" or "isle of wight" or "west midlands" or sussex or Hampshire or

For peer review only

Herefordshire or Hertfordshire or Kent or Lancashire Leicestershire or Lincolnshire or Merseyside or Norfolk or Northamptonshire or Northumberland or Nottinghamshire or Oxfordshire or Rutland or Shropshire or Somerset or Staffordshire or Suffolk or Surrey or "Tyne and Wear" or Warwickshire or Wiltshire or Worcestershire or Aberdeenshire or Angus or Forfarshire or Argyll* or Ayrshire or "County of Ayr" or "County of Banff" or "County of Berwick" or "County of Bute" or "County of Clackmannan" or "East Lothian" or "Ross and Cromarty" or "West Lothian" or Banffshire or Berwickshire or Buteshire or Caithness or Clackmannanshire or Dumfriesshire or Dunbartonshire or Fife or Invernessshire or Kincardineshire or Kinrossshire or Kirkcudbrightshire or Lanarkshire or Midlothian or Moray or Nairnshire or Orkney or Peebleshire or Perthshire or Renfrewshire or Roxburghshire or Selkirkshire or Shetland or Stirlingshire or Sutherland or Wigtownshire or "Blaenau Gwent" or "Isle of Anglesey" or "Merthyr Tydfil" or "Neath Port Talbot" or "Rhondda Cynon Taff" or "Vale of Glamorgan" or Bridgend or Caerphilly or Cardiff or Carmarthenshire or Ceredigion or Conwy or Denbighshire or Flintshire or Gwynedd or Wrexham or Antrim or Armagh or Down or Fermanagh or Londonderry or derry or Swansea or Torfaen or Newport or Pembrokeshire or Powys or Monmouthshire or Tyrone).ti,ab,in.

116. 1 or 2 or 3 or 4 or 5 or 6 or 7 or 8 or 115
117. 116 not 9
118. tenant support.ti,ab,kw.
119. perpetrator programme*.ti,ab,kw.
120. (violen* adj3 (sexual or domestic or child or partner* or spous* or couple* or family or interpersonal or date or dating or gender based or coercive or conjugal or victim* or survivor* or perpetrat* or technology-facilitated or wom#n or image based or honor based or obstetric)).ti,ab,kw.
121. (abus* adj3 (sexual or domestic or child or partner* or spous* or family or couple* or gender based or wom#n or wife or wives or victim* or survivor* or perpetrat* or financ* or economic or image-based or technology-facilitated)).ti,ab,kw.
122. (assault* adj3 (sexual or partner* or spous* or victim* or survivor* or perpetrat* or couple*)).ti,ab,kw.
123. 25 or 26 or 27 or 28 or 29 or 30 or 31 or 32 or 33 or 34 or 35 or 36 or 37 or 38 or 39 or 40 or 41 or 42 or 43 or 44 or 45 or 46 or 47 or 48 or 49 or 50 or 51 or 52 or 53 or 54 or 55 or 56 or 57 or 58 or 59 or 60 or 61 or 62 or 63 or 64 or 65 or 66 or 67 or 68 or 69 or 70 or 71 or 72 or 73 or 74 or 75 or 76 or 77 or 78 or 79 or 80 or 81 or 82 or 83 or 84 or 85 or 86 or 87 or 88 or 89 or 90 or 91 or 92 or 93 or 94 or 95 or 96 or 97 or 98 or 99 or 100 or 101 or 102 or 103 or 104 or 105 or 106 or 107 or 118 or 119
124. 10 or 11 or 12 or 13 or 14 or 15 or 16 or 17 or 18 or 19 or 20 or 21 or 22 or 23 or 24 or 108 or 109 or 110 or 111 or 112 or 113 or 114 or 120 or 121 or 122
125. 117 and 123 and 124
126. limit 125 to (english language and humans)

For peer review only

Grey literature example: Social care online

(combined v4:
UK terms [
 - Location:"'england"' including broader terms
 - OR Location:"'wales"' including this term only
 - OR Location:"'scotland"' including this term only
 - OR Location:"'northern ireland"' including this term only
 - OR Location:"'united kingdom"' including this term only
]
AND
service terms - all fields [
 - AllFields:"'specialised service"'
 - OR AllFields:"'specialist service"'
 - OR AllFields:"'support service"'
 - OR AllFields:"'abuse service"'
 - OR AllFields:"'violence service"'
]
AND
violence terms v2 [
 - SubjectTerms:"'domestic violence"' including related terms
 - OR SubjectTerms:"'sexual abuse"' including related terms
 - OR AllFields:"'sexual violence"'
 - OR AllFields:"'domestic abuse"'
 - OR AllFields:"'domestic violence"'
 - OR AllFields:"'sexual abuse"'
]
)

For peer review only

Appendix 4: Details of included studies

Study	Peer review or grey lit	Study design	Setting/ sector	Type of intervention	Type of abuse*	No. of outcomes
ADVA (2009)[1]	Grey lit.	Service evaluation	Third	*"Repair"*<br>Perpetrator programme<br>• Comprising individual and group work sessions. Primarily cognitive behavioural, focusing on motivation, responsibility, safety and acknowledgment<br>Women's service<br>• Supportive befriending, information about legal and support services, expectation management, group work, telephone contact, feedback on partners' attendance, safety planning and monitoring and awareness raising	DVA	7
Advance (2020)[2]	Grey lit.	Service evaluation	Third	*"Minerva"*<br>Multi-service<br>• Advocacy for access to other community services<br>• One-to-one and group work support on a range of issues including emotional wellbeing, physical and mental health, violence and abuse, problematic substance use<br>• Benefits and debt advice, housing	DVA	1
Barber et al (2000)[3]	Peer review	Pre-post	Public: Learning disability hospital	-<br>Psychological support<br>• Group therapy using a person-centred therapeutic model, which aimed to run interactive and structured educative sessions, supplemented with supportive and non-confrontational discussions	SVA	4

For peer review only

Study	Peer review or grey lit	Study design	Setting/ sector	Type of intervention	Type of abuse*	No. of outcomes
Bloomfield & Dixon (2015)[4]	Grey lit.	Non-randomised comparative	Public: National Probation Service	*"Integrated Domestic Abuse Programme (IDAP) and Community Domestic Violence Programme (CDVP)"*<br>Perpetrator programme<br>• Cognitive behavioural programme, which challenged offenders' attitudes and beliefs to change their behaviour	DVA	2
Bowen (2010)[5-9]	Peer review	Quasi experimental	Public: local probation service	*"Domestic Violence Perpetrator Programme"*<br>Perpetrator programme<br>• Psychoeducational treatment programme in a group setting, focusing on definitions of DV, socialisation, impact of DV on partners and children, empathy, sexual respect and accountability and communication	DVA	9
Brady et al (2021)[10]	Peer review	RCT	Mixed: Third (human rights charity); unclear ('clinical setting')	-<br>Psychological support<br>• Narrative exposure therapy, involving co-construction of traumatic experiences and a therapy plan, to process traumatic memories. Psychoeducation was also delivered to all participants	SVA	5
Burton et al (1998)[11]	Grey lit.	Service evaluation	Third	*"Violence Prevention Programme (VPP)"*<br>Perpetrator programme<br>• Structured group sessions designed to assist men in understanding why they abuse, how they can change it, and constructing respectful relationships with women<br>Multi-service<br>• Telephone advice and support; making contact with, and regular follow-up of, partners of men on VPP; one-to-one support sessions; two weekly support groups;	DVA	2

Study	Peer review or grey lit	Study design	Setting/ sector	Type of intervention	Type of abuse*	No. of outcomes
				advocacy for women users; weekly liaison with the VPP; interagency work, including workshops and training; administration; and training for new volunteers. These also involve safety planning, housing / financial / legal advice and education (understanding domestic violence)		
Callaghan et al (2020)[12, 13]	Grey lit.	Pre-post	Third: DV charities / organisations	*"Make a Change"*<br>Perpetrator programme<br>• Group-based intervention for perpetrators<br>Psychological support<br>• Individual support for (ex)partners	DVA	13
Calvert et al (2015)[14]	Peer review	Pre-post	Public: Tertiary psychotherapy service	-<br>Psychological support<br>• Group cognitive analytic therapy	SVA (CSA)	6
Clarke & Llewelyn (1994)[15]	Peer review	Pre-post	Public: NHS	-<br>Psychological support<br>• Cognitive analytic therapy	SVA (CSA)	4
Clarke & Pearson (2000)[16]	Peer review	Pre-post	Public: NHS	-<br>Psychological support<br>• Cognitive analytic therapy	SVA (CSA)	4
Cook et al (1991)[17]	Peer review	Pre-post	Public	-<br>Perpetrator programme<br>• Group treatment for sex offenders	SVA	1
Co-ordinated Action Against Domestic	Grey lit.	Data-set / service evaluation	Third	-<br>Advocacy<br>• 14 IDVA services, with types of support	DVA	11

For peer review only

Study	Peer review or grey lit	Study design	Setting/ sector	Type of intervention	Type of abuse*	No. of outcomes
Abuse (2012)[18, 19]				including safety planning, support with MARAC, liaison and support with police, support with criminal court cases, liaison and support with probation, support with civil orders, support with housing, financial benefits advice and support, support with immigration, health and wellbeing advice and support, and support with children		
Coy and Kelly (2011)[20]	Grey lit.	Service evaluation	Third	-<br>Advocacy<br>• Four IDVAs in different settings (police station, hospital A&E department, a community based DV project, and a women only VAW organisation)	DVA	2
Dheensa & Penny (2019)[21]	Grey lit.	Service evaluation	Third	-<br>Advocacy<br>• Learning disability ISVA	SVA	1
Dobash et al (1999)[22]	Peer review	Non-randomised comparative	Public	*"IDVPP"* and *"CHANGE"*<br>Perpetrator programme<br>• Two perpetrator programmes, both focused on challenging group work	DVA	3
Ellis (2012)[23]	Peer review	Pre-post	Third?	*"The Butterfly Programme"*<br>Psychosocial support<br>• Group therapy	SVA (CSA)	6
Farrell & Buckley (1999)[24]	Peer review	Pre-post	Public	*"The Domestic Violence Unit"*<br>Specialist DV police teams<br>• Police officers dedicated to dealing with DV cases with specialist training.	DVA	5
Ferrari et al (2018)[25]	Peer review	Randomised controlled trial	Third	*"PATH"*<br>Advocacy<br>• Specialist psychological advocates providing	DVA	6

For peer review only

Study	Peer review or grey lit	Study design	Setting/ sector	Type of intervention	Type of abuse*	No. of outcomes
				1:1 support on top of regular advocacy provision		
Friendship et al. (2003)[26]	Peer review	Pre-post with retrospectively selected comparison group	Public: Prisons	*"SOTP"*<br>Perpetrator programme<br>• Two programmes based on the cognitive behavioural model, using group sessions	DVA	3
Garry & Munro (2020)[27-29]	Grey lit.	Service evaluation	Third	*"The Rape and Sexual Violence Project (RSVP) adult counselling service"*<br>Psychological support<br>• Face-to-face counselling. Clients can also access RSVPs other support services	SVA	1
Geoghegan-Fittall et al (2020)[30]	Grey lit.	Service evaluation	Third	*"The Croydon Drive Project"*<br>Perpetrator programme<br>• Direct and indirect interventions and activity disruption. Interventions may be singular or concurrent	DVA	3
Gilchrist et al (2021)[31, 32]	Peer review	Randomised controlled trial	Mixed: NHS and voluntary sector	*"ADVANCE"*<br>Perpetrator programme<br>• Manualised evidence-informed tailored intervention focusing on developing healthy relationships	DVA	14
Goosey et al (2017)[33]	Peer review	Randomised controlled trial	Mixed: community safety team, police, drug and alcohol service, Thames Valley Probation, Youth Offending	-<br>Multi-service<br>• Integrated cased management using the Domestic Abuse Service Coordination model, including support for victims, counselling for perpetrators, and follow up	DVA	1

Study	Peer review or grey lit	Study design	Setting/ sector	Type of intervention	Type of abuse*	No. of outcomes
			Service, Domestic Abuse Perpetrator Service, Berkshire Women's Aid, Bracknell Forest Homes	police visits		
Halliwell et al (2019)[34]	Peer review	Non-randomised comparative	Mixed: Public and Third	-<br>Advocacy<br>• A hospital based IDVA who provided support and advice, risk assessment and safety planning, referral, partnership work and training hospital staff	DVA	8
Hester et al (2019)[35-38]	Grey lit	Service evaluation using RCT design	Third	*"Drive"*<br>Perpetrator programme<br>• Intensive case management and multi-agency response. Uses disruption, support and behaviour change interventions alongside protective work<br>Advocacy<br>• One-to-one IDVA support for the associated victims-survivors	DVA	10
Hester & Westmarland (2005)[39]	Grey lit.	Service evaluation	Mixed: Third, public	-<br>Multiple / multi-service<br>• 27 domestic violence projects split in to seven packages (criminal and civil justice, protection and prevention, Black and other ethnic minorities, health, multi-service, education and rural work). A sub selection of the 27 that report relevant outcomes and use the included methodology were extracted, including: Bradford Staying Put, Hammersmith and Fulham Standing Together, Northampton Sunflower Centre,	DVA	17

For peer review only

Study	Peer review or grey lit	Study design	Setting/ sector	Type of intervention	Type of abuse*	No. of outcomes
				Cheshire and Taunton – use of photographic evidence, Croydon Domestic Violence Advocate Service, Northampton Sunflower Centre		
Howarth et al (2009)[98, 99]	Grey lit	Service evaluation	Third	-<br>Advocacy<br>• Seven IDVA services in a range of rurality's, sizes and age of service	DVA	13
Howarth and Robinson (2016)[100]	Peer review	Pre-post	Unclear	-<br>Advocacy<br>• Seven IDVA services. Support provided included safety planning, housing and accommodation, child-related support, access to justice systems, health and wellbeing, and welfare and immigration support	DVA	4
Karatzias et al (2016)[101]	Peer review	Pre-post	Public: NHS	*"Trauma Recovery and Empowerment Model"*<br>Psychological support<br>• Group psychotherapy using cognitive behavioural and skills training techniques	DVA<br>SVA<br>(CSA)	5
Kelley et al (2022)[40]	Peer review	Non-randomised comparative	Private: Privately owned prison	*"Principle-based correctional counselling"*<br>Perpetrator programme<br>• Classes including modules on building rapport, exploration of reality, exploration of thought, exploration of feelings/behaviour.<br>• All participants also participated in the required CBT based Sexual offender Treatment programme	SVA	6
Kelly et al	Grey lit.	Service	Public	*"Domestic Violence Matters"*	DVA	5

Study	Peer review or grey lit	Study design	Setting/ sector	Type of intervention	Type of abuse*	No. of outcomes
(1999)[41]		evaluation		Specialist domestic violence police teams<br>• Five support workers who worked alongside the police to support women where an arrest of a perpetrator occurred.		
Kelly & Westmarland (2015)[42]	Grey lit.	Service evaluation	Third	*"DVPP"*<br>Perpetrator programme<br>• Twelve Respect accredited DVPPs. All have manuals and involved group work.<br>Unclear – Women's support service<br>• Each perpetrator programme had an integrated women's support service, however no further details are provided.	DVA	7
Koppensteiner et al (2019)[43]	Grey lit.	Randomised controlled trial	Public: Police	*"Project 360"*<br>Specialist domestic violence police teams<br>• Engagement workers who contact victims and offer further assistance, including information on legal options, referrals to support services, and safety planning	DVA	1
Lindsay et al (2011)[44]	Peer review	Pre-post	Unclear	-<br>Perpetrator programme<br>• A sex offender treatment programme, including modules on disclosure, pathways into offending, cognitive distortions, childhood abuse, victim awareness, relapse prevention, and adaptive attachment	SVA	2
Lindsay et al. (1998)[45]	Peer review	Pre-post	Public: Psychology / psychiatry department	-<br>Perpetrator programme<br>• Group sessions focused on sex offending and cognitive change, including taking responsibility, victim awareness and behaviours consistent with offending	SVA	1

For peer review only - http://bmjopen.bmj.com/site/about/guidelines.xhtml

Study	Peer review or grey lit	Study design	Setting/ sector	Type of intervention	Type of abuse*	No. of outcomes
Lowe et al (2017)[46]	Peer review	Pre-post	Third	-<br>Psychological support<br>• Group therapy. Sessions used a person centred, psychoeducational approach with elements of cognitive behavioural therapy	SVA (CSA)	3
McConnell et al (2017)[47, 48]	Peer review	Pre-post	Third	*"Caring Dads Safer Children"*<br>Perpetrator programme<br>• Includes seven treatment targets: anger/hostility; family cohesion/ coparenting/domestic violence; perceptions of the child as a problem; use of corporal punishment and other aversive behaviours; positive and involved parent-child relationship; self-centeredness; and misuse of substances	DVA	4
McCracken & Deave (2012)[49]	Grey lit.	Service evaluation	Third	*"Caring Dads Cymru"*<br>Perpetrator programme<br>• Group work programme based on cognitive behavioural therapy and motivational models of intervention	DVA	4
Michie & Lindsay (2012)[50]	Peer review	Non-randomised comparative	Public: NHS	-<br>Perpetrator programme<br>• Cognitive behavioural therapy with an empathy component	SVA	1
Morgan et al (2019)[51, 52]	Peer review	Pre-post	Third	*"RADAR/ADAPT"*<br>Perpetrator programme<br>• A multiagency initiative that provides a tiered needs-led approach. It provides a programme for perpetrators and support for victims	DVA	4
Morrison &	Peer review	Pre-post	Public: Local	-	SVA	1

For peer review only

Study	Peer review or grey lit	Study design	Setting/ sector	Type of intervention	Type of abuse*	No. of outcomes
Treliving (2002)[53]			psychotherapy service	Psychological support<br>• Group therapy aiming to provide men with a safe space to explore their difficulties and the impact of childhood sexual abuse	(CSA)	
Murphy et al (2007)[54]	Peer review	Pre-post	Public: Intellectual disability services	-<br>Perpetrator programme<br>• Group cognitive behavioural therapy for sexually abusive men	SVA	4
Oasis (2016)[55, 56]	Grey lit	Service evaluation	Third	-<br>Multi-service<br>• Counselling (6 sessions plus more if required)<br>• Refuge: a 12-bed refuge providing temporary emergency accommodation. Staff provide emotional and practical support	DVA	3
Ormston et al (2016)[57]	Grey lit.	Service evaluation	Third	*"The Caledonian System"*<br>Perpetrator programme<br>• Perpetrator programme covering lifelong change, responsibility for and to self, relationships, sexual respect, men and women, and children and fathering.<br>Multi-service<br>• Women's service including safety planning, information, advice, and emotional support to (ex)partners	DVA	6
Payne et al (2007)[58]	Peer review	Pre-post	Public (assumed as authors are clinical psychologists)	-<br>Psychological support<br>• Client centred group therapy	DVA<br>SVA<br>(CSA)	3
Peckham et al (2007)[59]	Peer review	Pre-post	Public: local community intellectual disability service	-<br>Psychological support<br>• Survivors group, involving educating clients and carers about sexual abuse, and weekly	SVA	6

For peer review only

Study	Peer review or grey lit	Study design	Setting/ sector	Type of intervention	Type of abuse*	No. of outcomes
				sessions using drawings, videos, dolls and pictures		
Robinson (2003)[60]	Grey lit.	Service evaluation	Mixed: Public (police, criminal justice) and third sector	*"Women's Safety Unit"*<br>Multi-service<br>• Advocacy, specialist counselling, legal services, housing services, refuge provision, target hardening and collecting evidence	DVA<br>SVA	11
Robinson (2004)[61]	Grey lit.	Service evaluation	Mixed: Public (police, probation, health, WSU, social services, housing, health) and third sector	-<br>MARAC<br>• Multiagency meeting which delegates actions to workers. Agencies include police, social services, probation, health, and education (where relevant)	DVA	6
Robinson (2006)[62]	Peer review	Pre-post	Mixed: Police, probation, health, WSU, social services, housing, health, third sector	-<br>MARAC<br>• Monthly multiagency meetings to discuss high risk victims, to share information and take actions to reduce future harm.	DVA	6
Robjant et al (2017)[63]	Peer review	Pre-post	Third	-<br>Psychological support<br>• Narrative exposure therapy, a therapy specifically designed for victims of trauma	SVA	2
Rose et al (2012)[64]	Peer review	Pre-post	Public: Community Learning Disability Services	-<br>Perpetrator programme<br>• Group programme with topics including sex education, emotion recognition, cognitive distortions, motivation for offences, empathy and relapse prevention	SVA	4
Ross et al.	Peer review	Non-	Public: Magistrate's	-	DVA	4

For peer review only

Study	Peer review or grey lit	Study design	Setting/ sector	Type of intervention	Type of abuse*	No. of outcomes
(2022)[65]		randomised comparison	Court	Advocacy<br>• An IDVA service based at a Specialist Domestic Abuse Court		
Ryan et al (2005)[66]	Peer review	Randomised controlled trial	Public: NHS psychology departments	-<br>Psychological support<br>• Individual and group therapy, focused on experiences and consequences of CSA	SVA (CSA)	4
SafeLives[67]	Grey lit.	Dataset / service evaluation	Third	-<br>Refuge<br>• No information	DVA	12
SafeLives[68]	Grey lit.	Dataset / service evaluation	Third	-<br>MARAC<br>• No information	DVA	3
SafeLives[69, 70]	Grey lit.	Dataset / service evaluation	Third	-<br>Sexual violence services<br>• No information	SVA	19
SafeLives[71]	Grey lit.	Dataset / service evaluation	Third	-<br>Health services<br>• No information	DVA	12
Safelives[72]	Grey lit.	Dataset / service evaluation	Third	-<br>Helpline<br>• No information	DVA	11
SafeLives[73-77]	Grey lit.	Dataset / service evaluation	Third	-<br>IDVA<br>• No information	DVA	12
SafeLives[78-82]	Grey lit.	Dataset / service evaluation	Third	-<br>Outreach<br>• No information	DVA	12

For peer review only

Study	Peer review or grey lit	Study design	Setting/ sector	Type of intervention	Type of abuse*	No. of outcomes
Sharpe et al (2001)[83]	Peer review	Pre-post	Public: Psychological therapies service	*"The Male Childhood Sexual Abuse Group"*<br>Psychological support<br>• Group therapy, no further information	SVA (CSA)	2
Skyner & Waters (1999)[84]	Peer review	NR	Mixed: Public (probation service) and third sector (NSPCC)	*"The Domestic Violence Prevention Programme"*<br>Perpetrator programme<br>• A cognitive behavioural programme with a variety of learning styles. A parallel support package is provided to (ex)partners and their children throughout	DVA	4
Smith et al (1995)[85]	Peer review	Pre-post	Public: Specialised therapy unit	*"Breakfree"*<br>Psychological support<br>• Individual therapy, daytime drop in facility, telephone contact, out-of-hours paging service, limited befriending service and a 'time out' facility where a client can stay in a safe environment for 1-2 nights	SVA (CSA)	3
Smith et al (2015)[86]	Peer review	Pre-post	Third	*"Domestic Abuse Recovering Together (DART)"*<br>Psychological support<br>• Mother and child sessions, designed to strengthen their relationship and support recovery. Followed by separate peer group sessions	DVA	3
Stanley et al (2011)[87]	Grey lit.	Service evaluation	Mixed: Third and Public (NHS, City Council)	*"Strength to change"*<br>Perpetrator programme<br>• Individual and group sessions over 40 weeks. Advocacy and advice are offered where appropriate. Sessions include mindfulness techniques, cognitive approaches, counselling, behavioural techniques.<br>• A women's service is available for	DVA	4

For peer review only

Study	Peer review or grey lit	Study design	Setting/ sector	Type of intervention	Type of abuse*	No. of outcomes
				(ex)partners offering practical and emotional support		
Stanley et al (2021)[88]	Grey lit	Service evaluation	Third	*"VOICES"*<br>Multi-service<br>• Trauma informed approach. Practitioners were given a new assessment framework, training and planning tools. No information on the specific types of activities or interventions is reported	DVA	6
Stanley et al (2021)[89]	Grey lit	Service evaluation	Third	*"SafeLives Co-Designed Pilots (SLCDPs)"*<br>Multi-service<br>• A suite of interventions for survivors, their children and perpetrators, using a whole family approach. This included community IDVA support, complex needs IDVA support, step down and recovery groups, children and young peoples work and 'Engage', which worked with the whole family	DVA	5
Strang et al (2017)[90]	Peer review	Randomised controlled trial	Mixed: Public (police), third sector	*"CARA"*<br>Perpetrator programme<br>• A two-day offender workshop, focusing on raising awareness, and moving offenders from denial and minimisation to acceptance and responsibility of harm, and conflict resolution	DVA	3
Taylor-Dunn & Erol (2019)[91, 92]	Grey lit	Service evaluation	Third	-<br>Advocacy<br>• Intensive one-to-one IDVA support within an immediate to medium term time-frame, implementing individual support plans and risk assessments to reduce risk and encourage recovery support	DVA	5

For peer review only

Study	Peer review or grey lit	Study design	Setting/ sector	Type of intervention	Type of abuse*	No. of outcomes
The Institute for Public Safety, Crime & Justice, University of Northampton (2019)[93]	Grey lit.	Service evaluation	Third	*"SHE"*<br>Psychological support<br>• A 'minimum support service (MSS)' with regular telephone check-ins, with an emphasis on safety and wellbeing, and an 'integrated support service (ISS+)' offering a face-to-face person-centred programme<br>Perpetrator programme<br>• 'Evolve', a programme domestic violence perpetrator programme working towards Respect accreditation	DVA	5
Trevillon et al (2014)[94]	Peer review	Non-randomised comparative	Public: Community mental health team	-<br>Advocacy<br>• Domestic violence training for clinicians, domestic violence manual for clinicians, mental illness training for domestic violence advisors, direct referral pathway to domestic violence advocacy, provision of a domestic violence advocacy service, comprising emotional and practical support, domestic violence education, support groups, safety planning and legal/housing support	DVA	11
Vallely et al (2005)[95]	Grey lit.	Service evaluation	Mixed: Public (Crown Prosecution Service, Police, Magistrates' Court, Probation service, local authorities) and Third sector	-<br>Specialist domestic violence court<br>• The service included a dedicated sitting for pre-trial matters and sentencing, allocated weekly; specialist magistrates with domestic violence training, specially trained CPS lawyers, and an independent advocacy service	DVA	5
Webster (2015)[96]	Grey lit.	Service evaluation	Third	*"Kent and Medway IDVA"*<br>Advocacy	DVA	2

Study	Peer review or grey lit	Study design	Setting/ sector	Type of intervention	Type of abuse*	No. of outcomes
				• Up to 21 IDVAs working with domestic abuse victims. No further information.		
Westmarland & Alderson (2013)[97]	Peer review	Pre-post	Third	-<br>Psychological support<br>• Face-to-face counselling, following a woman-centred, empowerment model, with elements of cognitive behavioural therapy, goal setting and rational emotive behaviour therapy	SVA	1
Williamson & Boyle (2012)[98]	Peer review	Pre-post	Public: NHS	*"IDVA Advocacy Intervention Programme"*<br>Advocacy<br>• No details reported	DVA	2
Williamson & Hester (2009)[99]	Grey lit.	Service evaluation	Third	*"South Tyneside Domestic Abuse Perpetrator Programme"*<br>Perpetrator programme<br>• A 'pro-feminist broadly cognitive behavioural model combined with gender analysis' approach. Group and one-to-one sessions.	DVA	2
Women's Aid (2022)[100-105]	Grey lit.	Service evaluation	Third	*"No Woman Turned Away"*<br>Housing (specialist caseworkers)<br>• Caseworkers provide telephone and email support to women looking for a refuge space	DVA	1
Women's Resource Centre (2011b)[106]	Grey lit.	Service evaluation	Third	*"Women and Girls Network"*<br>Psychological support<br>Counselling using trauma therapy, cognitive behavioural therapy, and integrated person-centred and feminist counselling practice. The organisation also offers a telephone helpline, body therapies, group programmes and specialist support projects	DVA	1
Women's Resource	Grey lit.	Service evaluation	Third	*"Ashiana"*<br>Multi-service	DVA	11

For peer review only

Study	Peer review or grey lit	Study design	Setting/ sector	Type of intervention	Type of abuse*	No. of outcomes
Centre (2011a)[107]				• Refuge: Safe accommodation across three schemes for South Asian, Turkish and Iranian women. Residents can stay for up to 1 year.<br>• Outreach: To provide support and reduce repeat victimisation. Ashiana also runs a DV support group for women in the wider community, as well as an end of therapy support group.		

For peer review only

1. ADVA. and S.P. Associates., *REPAIR (Resolve to End the Perpetration of Abuse in Relationships): A Community- and Whole-family-based Intervention Programme Targeting Perpetrators of Domestic Violence and Abuse in Devon. An evaluation of a three-year Invest to Save (ISB) PROJECT*. 2009: Exeter.
2. Advance, *'A Place to go Like This': Breaking the cycle of harm for mothers involved in offending who are survivors of domestic abuse, and their children.* 2020.
3. Barber, M., R. Jenkins, and C. Jones, *A survivor's group for women who have a learning disability.* British Journal of Developmental Disabilities, 2000. **46**(90, Pt 1): p. 31-41.
4. Bloomfield, S. and L. Dixon, *An outcome evaluation of the integrated domestic abuse programme (IDAP) and community domestic violence programme (CDVP).* London: National Offender Management Service, 2015.
5. Bowen, E., *Evaluation of a community based domestic violence offender rehabilitation programme. Unpublished doctoral thesis*, in *Centre for Forensic and Family Psychology*. 2004, University of Birmingham.
6. Bowen, E., *Therapeutic Environment and Outcomes in a UK Domestic Violence Perpetrator Program.* Small Group Research, 2010. **41**(2): p. 198-220.
7. Bowen, E. and E. Gilchrist, *Predicting dropout of court-mandated treatment in a British sample of domestic violence offenders.* Psychology, Crime & Law, 2006. **12**(5): p. 573-587.
8. Bowen, E., E. Gilchrist, and A.R. Beech, *Change in treatment has no relationship with subsequent re-offending in UK domestic violence sample: A preliminary study.* International Journal of Offender Therapy and Comparative Criminology, 2008. **52**(5): p. 598-614.
9. Bowen, E., E.A. Gilchrist, and A.R. Beech, *An Examination of the Impact of Community-Based Rehabilitation on the Offending Behaviour of Male Domestic Violence Offenders and the Characteristics Associated with Recidivism.* Legal and Criminological Psychology, 2005. **10**(2): p. 189-209.
10. Brady, F., et al., *Narrative exposure therapy for survivors of human trafficking: feasibility randomised controlled trial.* BJPsych Open, 2021. **7**(6).
11. Burton, S., L. Regan, and L. Kelly, *Supporting women and challenging men: lessons from the Domestic Violence Intervention Project.* 1998.
12. Callaghan, J., et al., *Make a Change: An evaluation of the implementation of an early response intervention for those who have used abusive behaviours in their intimate relationships*. 2020.
13. Respect and Women's Aid Federation England, *Make a Change Executive Summary*.
14. Calvert, R., S. Kellett, and T. Hagan, *Group cognitive analytic therapy for female survivors of childhood sexual abuse.* British Journal of Clinical Psychology, 2015. **54**(4): p. 391-413.
15. Clarke, S. and S. Llewelyn, *Personal constructs of survivors of childhood sexual abuse receiving cognitive analytic therapy.* British Journal of Medical Psychology, 1994. **67(3)**: p. 273-289.
16. Clarke, S. and C. Pearson, *Personal constructs of male survivors of childhood sexual abuse receiving cognitive analytic therapy.* British Journal of Medical Psychology, 2000. **73(2)**: p. 169-177.
17. Cook, D.A., et al., *The Berkeley group: ten years' experience of a group for non-violent sex offenders.* The British Journal of Psychiatry, 1991. **158**(2): p. 238-243.

For peer review only

18. Co-Ordinated Action Against Domestic, A., *Insights into domestic abuse 1: a place of greater safety.* 2012.
19. Co-ordinated Action Against Domestic Abuse (CAADA), *Insights National Dataset 2011–12 Appendix to: A place of greater safety*. 2012.
20. Coy, M. and L. Kelly, *Islands in the stream: an evaluation of four London independent domestic violence advocacy schemes*. 2011.
21. Dheensa, S., G. Penny, and Safelink, *"In my way…" Evaluation Of Learning Disabilities Sexual Abuse Support Services*.
22. Dobash, R.P., et al., *A research evaluation of British programmes for violent men.* Journal of Social Policy, 1999. **28**(2): p. 205-233.
23. Ellis, F., *Rehabilitation programme for adult survivors of childhood sexual abuse.* Journal of public mental health, 2012. **11**(2): p. 88-92.
24. Farrell, G. and A. Buckley, *Evaluation of a UK police domestic violence unit using repeat victimisation as a performance indicator.* The Howard Journal of Criminal Justice, 1999. **38**(1): p. 42-53.
25. Ferrari, G., et al., *Psychological advocacy towards healing (PATH): A randomized controlled trial of a psychological intervention in a domestic violence service setting.* Plos One, 2018. **13**(11).
26. Friendship, C., R.E. Mann, and A.R. Beech, *Evaluation of a national prison-based treatment program for sexual offenders in England and Wales.* Journal of interpersonal violence, 2003. **18**(7): p. 744-759.
27. Garry, K. and P. Goodwin, *Adult Counselling Service Phase two evaluation report: year 1 2020-21*. 2021, Merida Associates.
28. Karen Garry and Ellie Munro, *Adult Counselling Service Interim evaluation | May 2019*. 2019.
29. Karen Garry and Ellie Munro, *Adult Counselling Service Evaluation report – phase one 2016-2020*. 2020.
30. Geoghegan-Fittall, S., C. Keeble, and D. Wunsch, *The Croydon Drive Project: A 2-year Evaluation Final Report*. 2020.
31. Gilchrist, E., et al., *Using the Behaviour Change Wheel to design an intervention for partner abusive men in drug and alcohol treatment.* Pilot and feasibility studies, 2021. **7**(1): p. 1-14.
32. Gilchrist, G., et al., *ADVANCE integrated group intervention to address both substance use and intimate partner abuse perpetration by men in substance use treatment: a feasibility randomised controlled trial.* BMC public health, 2021. **21**(1): p. 1-20.
33. Goosey, J., L. Sherman, and P. Neyroud, *Integrated case management of repeated intimate partner violence: A randomized, controlled trial.* Cambridge Journal of Evidence-Based Policing, 2017. **1**(2): p. 174-189.
34. Halliwell, G., et al., *Cry for health: a quantitative evaluation of a hospital-based advocacy intervention for domestic violence and abuse.* Bmc Health Services Research, 2019. **19**(1).
35. Hester, M., et al., *Evaluation of Year 2 of the Drive Project – A Pilot to Address High Risk Perpetrators of Domestic Abuse*. 2019.
36. Hester, M., et al., *Evaluation of the Drive Project – a pilot to address high-risk perpetrators of domestic abuse Year 1 Feasibility Study*. 2017.
37. Hester, M., et al., *Evaluation of the Drive Project – A Three-year Pilot to Address High-risk, High-harm Perpetrators of Domestic Abuse*. 2019.
38. Hester, M., et al., *Evaluation of the Drive Project – A Three-year Pilot to Address High-risk, High-harm Perpetrators of Domestic Abuse EXECUTIVE SUMMARY.* 2020.
39. Hester, M. and N. Westmarland, *Tackling domestic violence: effective interventions and approaches*. 2005: Home Office Research, Development and Statistics Directorate.

For peer review only

40. Kelley, T.M., et al., *The Efficacy of Principle-Based Correctional Counseling for Improving the Self-Control and Mental Health of People Incarcerated for Sexual Violence.* Violence Against Women, 2022. **28**(2): p. 573-592.
41. Kelly, L., *Domestic Violence Matters: an evaluation of a development project*. 1999.
42. Kelly, L. and N. Westmarland, *DOMESTIC VIOLENCE PERPETRATOR PROGRAMMES STEPS TOWARDS CHANGE: Project Mirabal Final Report*. 2015.
43. Koppensteiner, M.F., J. Matheson, and R. Plugor, *Project 360: An intervention to address victim-police engagement in repeat domestic violence cases*. 2017.
44. Lindsay, W.R., et al., *Comparing offenders against women and offenders against children on treatment outcome in offenders with intellectual disability.* Journal of Applied Research in Intellectual Disabilities, 2011. **24**(4): p. 361-369.
45. Lindsay, W.R., et al., *The treatment of six men with a learning disability convicted of sex offences with children.* British Journal of Clinical Psychology, 1998. **37**: p. 83-98.
46. Lowe, M., et al., *CORE assessment of adult survivors abused as children: A NAPAC group therapy evaluation.* Counselling & Psychotherapy Research, 2017. **17**(1): p. 71-79.
47. McConnell, N., et al., *Caring dads safer children*. 2014.
48. McConnell, N., M. Barnard, and J. Taylor, *Caring Dads Safer Children: Families' Perspectives on an Intervention for Maltreating Fathers.* Psychology of Violence, 2017. **7**(3): p. 406-416.
49. McCracken, K. and T. Deave, *Evaluation of the Caring Dads Cymru programme. Final report.* 2012.
50. Michie, A.M. and W.R. Lindsay, *A treatment component designed to enhance empathy in sex offenders with an intellectual disability.* The British Journal of Forensic Practice, 2012. **14**(1): p. 40-48.
51. Morgan, S. and J. Parkes, *The Hampshire Domestic Abuse Prevention Partnership 2016-2018: process and outcomes evaluation.* 2018.
52. Morgan, S.A., B.M.S. McCausland, and J. Parkes, *Baseline characteristics and outcomes of the main perpetrator programme within the Hampshire Domestic Abuse Prevention Partnership, UK: A mixed methods study.* Plos One, 2019. **14**(7).
53. Morrison, A. and L. Treliving, *Evaluation of outcome in a dynamically orientated group for adult males who have been sexually abused in childhood.* British Journal of Psychotherapy, 2002. **19**: p. 59-75.
54. Murphy, G., et al., *Cognitive-behavioural treatment for men with intellectual disabilities and sexually abusive behaviour: A pilot study.* Journal of Intellectual Disability Research, 2007. **51(11)**: p. 902-912.
55. Oasis. *Impact report 2013-2014*. 2014; Available from: https://issuu.com/kristinagwynne/docs/oasis_annual_report_2013-2014-web/1.
56. Oasis, *Impact Report 2015-2016.* 2016.
57. Ormston, R., C. Mullholland, and L. Setterfield, *Caledonian system evaluation: analysis of a programme for tackling domestic abuse in Scotland*. 2016, Edinburgh: Scotland. Scottish Government Social Research. vi, 88.
58. Payne, A., H. Liebling-Kalifani, and S. Joseph, *Client-centred group therapy for survivors of interpersonal trauma: A pilot investigation.* Counselling & Psychotherapy Research, 2007. **7**(2): p. 100-105.
59. Peckham, N.G., S. Howlett, and A. Corbett, *Evaluating a survivors group pilot for a women with significant intellectual disabilities who have been sexually abused.* Journal of Applied Research in Intellectual Disabilities, 2007. **20**(4): p. 308-322.

For peer review only

60. Robinson, A.L., *The Cardiff Women's Safety Unit: A multi-agency approach to domestic violence.* Final Evaluation Report. Cardiff University: School of Social Sciences.(Available online at http://www. cf. ac. uk/socsi/whoswho/robinson. html), 2003.
61. Robinson, A.L., *Domestic Violence MARACs (Multi-Agency Risk Assesment Conferences) for Very High-risk Victims in Cardiff, Wales: A Process and Outcome Evaluation.* 2004.
62. Robinson, A.L., *Reducing repeat victimization among high-risk victims of domestic violence: The benefits of a coordinated community response in Cardiff, Wales.* Violence Against Women, 2006. **12(8)**: p. 761-788.
63. Robjant, K., J. Roberts, and C. Katona, *Treating posttraumatic stress disorder in female victims of trafficking using narrative exposure therapy: A retrospective audit.* Frontiers in Psychiatry, 2017. **8**.
64. Rose, J., et al., *A sex offender treatment group for men with intellectual disabilities in a community setting.* The British Journal of Forensic Practice, 2012.
65. Ross, J., J. Sebire, and H. Strang, *Tracking Repeat Victimisation After Domestic Abuse Cases Are Heard With and Without Independent Domestic Violence Advisors (IDVAs) in an English Magistrate's Court.* Cambridge Journal of Evidence-Based Policing, 2022: p. 1-15.
66. Ryan, M., et al., *A prospective study of the effectiveness of group and individual psychotherapy for women CSA survivors.* Psychology and Psychotherapy: Theory, Research and Practice, 2005. **78(4)**: p. 465-480.
67. SafeLives, *Insights refuge England and Wales dataset 2015-18 Adult refuge services*. 2018.
68. SafeLives, *Defining Marac Outcomes National Pilot*. 2012.
69. SafeLives, *Insights England and Wales dataset 2018-19 Sexual violence services* 2019.
70. SafeLives, *Insights SV dataset 2020-21 Sexual violence (SV) services*. 2021.
71. SafeLives, *Insights health England and Wales dataset 2015-2018 Adults health-based services*. 2018.
72. SafeLives, *Insights helpline England and Wales dataset 2014-17 Adult helpline services*. 2017.
73. SafeLives, *Insights Idva national dataset 2012–13 Adult independent domestic violence advisor (Idva) services*. 2015.
74. SafeLives, *Insights Idva national dataset 2013–14 Adult independent domestic violence advisor (Idva) services*. 2015.
75. SafeLives, *Insights Idva England and Wales dataset 2016-17 Adult independent domestic violence advisor (Idva) services*. 2017.
76. SafeLives, *Insights Idva England and Wales dataset 2018-19 Adult Independent domestic violence advisor (Idva) services*. 2019.
77. SafeLives, *Insights Idva dataset 2020-21 Adult Independent domestic violence advisor (Idva) services*. 2021.
78. SafeLives, *Insights outreach national dataset 2012–13 Adult outreach services*. 2015.
79. SafeLives, *Insights outreach national dataset 2013–14 Adult outreach services.* 2015.
80. SafeLives, *Insights outreach England and Wales dataset 2016-17 Adult outreach services*. 2017.
81. SafeLives, *Insights outreach England and Wales dataset 2018-19 Adult outreach services*. 2019.
82. SafeLives, *Insights outreach dataset 2020-21 Adult outreach services*. 2021.
83. Sharpe, J., et al., *Group analytic therapy for male survivors of childhood sexual abuse.* Group Analysis, 2001. **34**: p. 195-209.
84. Skyner, D.R. and J. Waters, *Working with perpetrators of domestic violence to protect women and children: a partnership between Cheshire Probation Service and the NSPCC.* Child Abuse Review: Journal of the British Association for the Study and Prevention of Child Abuse and Neglect, 1999. **8**(1): p. 46-54.

For peer review only

85. Smith, D., et al., *Adults with a history of child sexual abuse: Evaluation of a pilot therapy service.* British Medical Journal, 1995. **310(6988)**: p. 1175-1178.
86. Smith, E., et al., *Strengthening the mother-child relationship following domestic abuse: Service evaluation.* Child Abuse Review, 2015. **24**(4): p. 261-273.
87. Stanley, N., et al., *An evaluation of a new initiative for male perpetrators of domestic violence* 2011.
88. Stanley, N. and a. et, *Roadmap evaluation: final report - VOICES*. 2021, Bristol: Women's Aid
SafeLives. 279.
89. Stanley, N. and a. et, *Roadmap evaluation: final report - SLCDPs*. 2021, Bristol: Women's Aid
SafeLives. 279.
90. Strang, H., et al., *Reducing the harm of intimate partner violence: Randomized controlled trial of the Hampshire Constabulary CARA Experiment.* Cambridge Journal of Evidence-Based Policing, 2017. **1**(2): p. 160-173.
91. Holly Taylor-Dunn and Rosie Erol, *Evaluation of SaferPlaces' Independent Domestic Violence Advisor Services.* 2019.
92. Taylor-Dunn, H. and R. Erol, *Improving the safety and well-being of domestic abuse survivors: The role of a specialist organisation in supporting the work of Independent Domestic Violence Advisors.* Crime Prevention and Community Safety, 2021. **23**(2): p. 115-136.
93. Eve; The Institute for Public Safety, C.J., *2019*. Evaluation of SHE.
94. Trevillion, K., et al., *Linking abuse and recovery through advocacy: An observational study.* Epidemiology and Psychiatric Sciences, 2014. **23**(1): p. 99-113.
95. Vallely, C., *Evaluation of domestic violence pilot sites at Caerphilly (Gwent) and Croydon 2004/05.* 2005.
96. Webster, R., *Kent and Medway Independent Domestic Violence Advisor Service Impact Evaluation FINAL REPORT*. 2015.
97. Westmarland, N. and S. Alderson, *The health, mental health, and well-being benefits of rape crisis counseling.* Journal of Interpersonal Violence, 2013. **28**(17): p. 3265-3282.
98. Williamson, D. and A. Boyle, *Results of the domestic violence advocacy program at addenbrookes hospital.* Academic Emergency Medicine, 2012. **19**: p. 741.
99. Williamson, E. and M. Hester, *Evaluation of the South Tyneside Domestic Abuse Perpetrator Programme (STDAPP) 2006-2008.* University of Bristol, 2009.
100. Women's Aid, *Nowhere To Turn, 2022: Findings from the sixth year of the No Woman Turned Away project*. 2022.
101. Miles, C. and K. Smith, *Nowhere to turn, 2018: findings from the second year of the No Woman Turned away project*. 2018, Bristol: Women's Aid. 48.
102. Women's Aid, *NOWHERE TO TURN Findings from the first year of the No Woman Turned Away project*. 2017.
103. Women's Aid, *NOWHERE TO TURN Findings from the third year of the No Woman Turned Away project*. 2019.
104. Women's Aid, *NOWHERE TO TURN Findings from the fourth year of the No Woman Turned Away project*. 2020, Women's Aid Federation of England.
105. Women's Aid, *NOWHERE TO TURN 2021 Findings from the fifth year of the No Woman Turned Away project*. 2021.
106. Women's Resource, C., *Women and Girls Network: the WGN empowerment model - redressing negative individual and social impacts of gendered violence through holistic counselling.* 2011.

For peer review only

107. Women's Resource, C., *Ashiana Network: journey towards safety.* 2011.

For peer review only

Appendix 5: Outcome measures by domain and subdomain

Category	Domain	Subdomain	Measure	N
**Health**	**Mental health**	**Depression**		**16**
			Beck Depression Inventory	6
			Hospital Anxiety and Depression Scale - Depression	4
			Patient Health Questionnaire	3
			PROMIS depression Short Form	1
			Single item – "Is the client experiencing depression?"	1
			Single item – reduction in feeling depressed	1
		**Anxiety**		**10**
			Hospital Anxiety and Depression Scale - Anxiety	4
			Generalized Anxiety Disorder Scale	3
			PROMIS Anxiety Short Form	1
			Spiel State Anxiety	1
			Single item – "Is the client experiencing anxiety?"	1
		**Trauma**		**12**
			Primary care PTSD screen	2
			Impact of Event Scale	2
			PTSD Checklist for DSM-5 (PCL-5)	1
			PTSD Symptom Scale	1
			Posttraumatic Diagnostic Scale	1
			Post-traumatic Stress Disorder Scale	1
			Changes in Outlook Questionnaire	1
			The Clinician Administered PTSD Scale	1
			PTSD Checklist (PCL-C)	1
			Single item – "I have "flashbacks" about what happened"	1
		**General mental health**		**4**
			General Health Questionnaire (GHQ-28)	2
			Single item - "Current mental health rating"	1

For peer review only

Category	Domain	Subdomain	Measure	N
			No information – reported outcome is "Improved mental health"	1
		**Psychological symptoms**		**19**
			The Clinical Outcomes in Routine Evaluation - Outcome Measure (CORE-OM)	5
			The Symptom Checklist 90 revised (SCL-90R)	4
			Brief Symptom Inventory	2
			Jehu Beliefs Inventory	2
			The Shutdown Dissociation Scale (Shu-Dis)	1
			Dissociative Experiences Scale (DES)	1
			Belief Inventory	1
			Social Activity and Distress Scale	1
			Delusions, Symptoms and States Inventory	1
			Single item – “Is the client experiencing emotional instability?”	1
		**Anger**		**2**
			The Novaco Anger Scale	1
			PROMIS Anger Short Form	1
		**Understanding feelings**$		2
			Three Principles Inventory$	1
			Single item - "Understanding feelings better"$	1
		**Self-Harm / Suicide**$		**5**
			Single item – reduction in experiencing suicidal thoughts or feelings$	1
			Single item – reduction in self harm$	1
			Single item – “Is the client experiencing self-harm?” $	1
			Single item – “Is the client experiencing suicidal thoughts?” $	1
			Single item – “Is the client experiencing suicidal behaviours?” $	1
		**Healthcare utilisation / resource use**$		**5**
			Number / percent of participants using healthcare resources (e.g., A&E, GP, psychiatrist)$	3
			Number / percent of participants using community health and social service resources (e.g., health visitor, drug worker) $	1
			Days of admission$	1
		**Mental wellbeing**$		**4**

For peer review only

Category	Domain	Subdomain	Measure	N
			Short Warwick–Edinburgh Mental Wellbeing Scale (SWEMWBS) $	4
				2
		**Self-care**$	Single item - "Is the client experiencing trouble sleeping?" $	1
			Single item - "Is the client experiencing problems with eating?" $	1
				**3**
		**Quality of life**$	Short Form 12 (SF12) $	2
			Manchester Short Assessment of Quality of Life$	1
		**Health, mental health and wellbeing**$		**1**
			Taking Back Control Tool$	1
	**Physical health**	**Self-reported physical health**		**8**
			EQ-5D	3
			Visual analogue scale for physical health	2
			Single item – "Current physical health (poor/fair/good/very good/excellent)"	1
			No information – reported outcome is "Improved physical health (DV specific)"	1
			No information – reported outcome is "Improved physical health (not DV specific)"	1
		**Alcohol and drug use**		**5**
			Alcohol Use Disorders Identification Test (AUDIT) $	1
			Single item – "Current drug use (excessive/high/moderate/low/none)" $	1
			Single item – "Current alcohol use (excessive/high/moderate/low/none)" $	1
			Single item – "Alcohol problem (yes/no)" $	1
			Single item – "Drug problem (yes/no)" $	1
				**5**
		**Healthcare utilisation / resource use**$	Number / percent of participants using healthcare resources (e.g., A&E, GP, psychiatrist)$	3
			Number / percent of participants using community health and social service resources (e.g., health visitor, drug worker) $	1
			Days of admission$	1
		**Quality of life**$		**3**
			Short Form 12 (SF12) $	2

For peer review only

Category	Domain	Subdomain	Measure	N
			Manchester Short Assessment of Quality of Life[$]	1
		**Health, mental health and wellbeing**[$]		**1**
			Taking Back Control Tool[$]	1
	**Wellbeing**	**Wellbeing**		**7**
			Outcome Star	1
			Adult wellbeing scale	1
			Benefits of Therapy scale	1
			Personal Outcomes and Wellbeing Record (POWeR)	1
			Victim survey (perceived wellbeing - safety and wellbeing)	1
			Wellbeing survey (9 domains)	1
			CORE monitoring form	1
			Perceived Severity of Problems/Concerns Questionnaire	1
		**Social ties/ relationships**		**5**
			Social Inclusion Scale[$]	1
			Inventory of interpersonal problems 32 (IIP-32) [$]	1
			Single item - "Current social and community support (no social contact / negative social contacts / some positive contacts / majority positive contacts / all positive)" [$]	1
			No information – reported outcome is "Improved relationship with family"[$]	1
			No information – reported outcome is "Improved social support"[$]	1
		**Understanding feelings**		**2**
			Three Principles Inventory[$]	1
			Single item - "Understanding feelings better"[$]	1
		**Mental wellbeing**[$]		**4**
			Short Warwick–Edinburgh Mental Wellbeing Scale (SWEMWBS) [$]	4
		**Self-care**[$]		**2**

For peer review only

Category	Domain	Subdomain	Measure	N
			Single item - "Is the client experiencing trouble sleeping?" $	1
			Single item - "Is the client experiencing problems with eating?" $	1
				**3**
		**Quality of life$**	Short Form 12 (SF12) $	2
			Manchester Short Assessment of Quality of Life$	1
		**Health, mental health and wellbeing$**		**1**
			Taking Back Control Tool$	1
**Behavioural**	**Behavioural**	**Challenging behaviour**		**1**
			Challenging Behaviour Inventory	1
		**Self-Harm / Suicide$**		**5**
			Single item – reduction in experiencing suicidal thoughts or feelings$	1
			Single item – reduction in self harm$	1
			Single item – "Is the client experiencing self-harm?" $	1
			Single item – "Is the client experiencing suicidal thoughts?" $	1
			Single item – "Is the client experiencing suicidal behaviours?" $	1
		**Alcohol and drug use$**		**5**
			Alcohol Use Disorders Identification Test (AUDIT) $	1
			Single item – "Current drug use (excessive/high/moderate/low/none)" $	1
			Single item – "Current alcohol use (excessive/high/moderate/low/none)" $	1
			Single item – "Alcohol problem (yes/no)" $	1
			Single item – "Drug problem (yes/no)" $	1
**Empowerment**	**Empowerment**	**Self-esteem**		**12**
			Rosenberg's self-esteem scale	7
			Culture Free Self Esteem Inventory	2
			The Self Concept Questionnaire	1
			Self-esteem Assessment Tool	1
			No information – reported outcome is "Improved self-esteem"	1
		**Coping and resilience**		**3**
			Resilience Research Centre Adult Resilience Measure (RRC-ARM)	1

For peer review only

Category	Domain	Subdomain	Measure	N
			Cope and Recover Questionnaire	1
			No information – reported outcome is "Improved coping"	1
		**Locus of control**		**1**
			Review of Personal Effectiveness with Locus of Control	
		**Empowerment**		**5**
			The Empowerment Star	2
			Measure of Victim Empowerment Related to Safety Scale (MOVERS)	1
			Coping and confidence outcome measure	2
		**Assertiveness**		**1**
			Assertiveness Questionnaire	1
		**Knowledge**		**1**
			Goal Attainment Scale	1
		**Social ties/ relationships**[$]		**5**
			Social Inclusion Scale[$]	1
			Inventory of interpersonal problems 32 (IIP-32) [$]	1
			Single item - "Current social and community support (no social contact / negative social contacts / some positive contacts / majority positive contacts / all positive)" [$]	1
			No information – reported outcome is "Improved relationship with family"[$]	1
			No information – reported outcome is "Improved social support"[$]	1
		**Understanding feelings**[$]		**2**
			Three Principles Inventory[$]	1
			Single item - "Understanding feelings better"[$]	1
				2
			No information – reported outcome "Independence"	1
		**Independence**		
			No information – reported outcome "Increased likelihood of employment "	1
		**Awareness of support**		1

For peer review only

Category	Domain	Subdomain	Measure	N
			Number of criminal justice contacts	1
**Socio-economic circumstances**	**Socio-economic circumstances**	**Employment/ education**		**5**
			Single item - "Employment status (FT employed/PT employed/unemployed/caring for children/family)"	1
			Single item - "Employment status (paid employment/voluntary work/education/retired)"	1
			Single item - "Returned to/begun employment or education"	1
			Single item - "Education, employment and training (none / occasional / some sustained / sustained / sustained and fulfilling)"	1
			No information – reported outcome "Increased likelihood of employment"$	1
		**Finances**		**3**
			No information – reported outcome "Financial savings"	1
			Single item - "Perception of income (high/comfortable/managing with treats/managing with occasional treats/essentials only/struggling)"	1
			Single item - "Current financial situation (significant financial problems/being reliant on others/unable to pay for essentials/managing essentials but nothing left over/comfortably managing and no financial concerns)"	1
		**Housing**		**4**
			No information – reported outcome "Access to housing"	1
			Single item – "Current housing (homeless / transitory / unsuitable / temporary / secure)"	1
			Single item – "Number of women accommodated in suitable refuge spaces"	1
			Number / percent of participants using supported accommodation resources (e.g., weeks spent in refuge / staffed accommodation / B&B, boarding house or hotel / homeless and living with friends or family)	1
				**1**
				5
		**Social ties/ relationships$**	Social Inclusion Scale$	1
			Inventory of interpersonal problems 32 (IIP-32) $	1
			Single item - "Current social and community support (no social contact /	1

For peer review only

Category	Domain	Subdomain	Measure	N
			negative social contacts / some positive contacts / majority positive contacts / all positive)" $	
			No information – reported outcome is "Improved relationship with family"$	1
			No information – reported outcome is "Improved social support"$	1
**Changing perpetrator behaviour**	**Changing perpetrator behaviour**	**Anger**		**1**
			The Novaco Anger Scale	1
		**Perpetrator needs**		**1**
			Distribution of needs	1
		**Attitudes to women / violence**		**20**
			Inventory of Beliefs About Wife Beating	1
			Questionnaire on Attitudes Consistent with Sexual Offending (QACSO)	3
			Socio-Sexual Knowledge and Attitudes Assessment – Revised (SSKAAT-R)	1
			The Intimate Partner Violence Responsibility Attribution Scale (IPVRAS)	1
			URICA-DV Readiness to change	1
			Propensity for Abusiveness Scale (PAS)	2
			Paulhus Deception Scale (PDS)	1
			Interpersonal Reactivity Index (IRI)	1
			Sexual Attitudes and Knowledge Scale	1
			Victim Empathy Scale-Adapted	1
			Sexual Offenders Self Appraisal Scale	1
			Treatment motivation Questionnaire	1
			Interpersonal Dependency Inventory	1
			Attitudes Toward Exhibitionism Questionnaire	1
			No information - "Attitudes to the offence"	1
			No information - "Attitudes towards women"	1
			No information - "Acceptance of responsibility"	1
		**Desirable responding**		**3**
			Balanced Inventory of Desirable Reporting	3
		**Locus of control / self-control**		**6**
			Multidimensional Locus of Control Scale	1
			Rotter's Locus of Control Scale	1
			Brief self-control scale (BSCS)	1
			Low self-control scale (LSCS)	1

For peer review only

Category	Domain	Subdomain	Measure	N
			Levenson Locus of Control	1
			The Nowicki-Strickland Locus of Control Scale	1
		**Reasons for violence**		**2**
			Number of reasons for violence given	1
			Number of participants reporting each of 7 potential reasons for abuse	1
**Victim-perpetrator relationship**	**Victim-perpetrator relationship**	**Victim-perpetrator relationship**		**12**
			The Communication Patterns Questionnaire-Short Form (CPQ-SF)	1
			Respectful communication	1
			Awareness of self and others	1
			Attitude to partner (self-partner correlation)	1
			Single item - "Relationship status"	1
			Single item - "Hopes for the relationship"	1
			Single item - "Living circumstances (with perpetrator / not living together / intermittently living together)	6
**Parenting**	**Parenting**	**Parenting**		**7**
			Parent Empowerment and Efficacy Measure (PEEM)	1
			Camberwell Assessment of Need for Mothers (Short version)	1
			Parenting Stress Index	2
			Parental Acceptance-Rejection Questionnaire (PARQ)	2
			The Parental Locus of Control Scale	1
**DSVA**	**Experience of DSVA**	**Impact of abuse**		**2**
			Number of participants experiencing each of 18 potential impacts of abuse	1
			Mean number of impacts of abuse	
		**Indicators of ongoing abuse**		**5**
			Additional MARACs/Reappearance at MARAC	3
			Other evidence of abuse (e.g. harassing texts)	1
			Cases reopened	1
		**Perceived safety**		**7**
			Safety Behaviour Checklist	1
			Single item - "Partner fearful (1= never to 4=often)"	1

For peer review only

Category	Domain	Subdomain	Measure	N
			Single item - "Feeling of safety (much safer / somewhat safer / no change / less safe)"	1
			Safety (6 items)	2
			Single item - "How safe do you feel? (not safe / a little unsafe / somewhat safe / very safe)	1
			No information - reported outcome is "increased safety"	1
			No information - reported outcome is "avoidance of forced marriage"	1
		**Revictimization**		**4**
			Average number of repeat victims per month	1
			Number / percent of repeat victims	1
			Number / percent of women reporting repeat victimisation (for IPA)	1
			Number / percent of women disclosing further incidents of violence to IDVA	1
		**Presence of abuse**$		**46**
			Severity of Abuse Grid - presence: Number / percent of people responding yes to the presence of abuse (reported for physical abuse, sexual abuse, harassment and stalking and jealous and controlling behaviour) $	10*
			Severity of Abuse Grid - cessation: Number / percent of people reporting no abuse$	11*
			Severity of Abuse Grid - reduction in presence: Number / percent of people reporting a reduction of abuse (reported for physical abuse, sexual abuse, harassment and stalking and jealous and controlling behaviour) $	1
			Severity of Abuse Grid - multiple types: Number / percent of people reporting multiple types of abuse$	7*
			Number / percent of participants experience concurrent domestic abuse as well as sexual abuse$	1
			Number / percent of participants who have been subjected to adult sex offences$	1
			Number / percent of men abusing each month$	1
			Space for Action Survey (% responding yes to 12 indicators) $	1
			Physical and Sexual Violence Survey (% responding yes to 7 indicators) $	1
			Harassment and other abusive acts survey (% responding yes to 6 indicators) $	1
			Fathering survey / using children against a partner (% responding yes to 5 indicators) $	2

For peer review only

Category	Domain	Subdomain	Measure	N
			Social media perpetration / victimisation survey$	1
			Composite Abuse Scale$	3
			Abusive Behaviour Inventory$	2
			Controlling Behaviour Inventory$	3
		**Number of abusive incidents or behaviours$**		**4**
			Mean number of abusive incidents per man$	1
			Mean number of abusive behaviours reported by participants$	1
			Number / percent of participants experiencing between 0 and 7 behaviours (reported for emotional, physical and sexual behaviours) $	1
			IMPACT monitoring toolkit (reported for emotional, physical, and sexual behaviours) $	1
		**Severity of abuse$**		**29**
			Severity of Abuse Grid - severity: Number / percent of participants reporting high / moderate / standard severity (reported for physical abuse, sexual abuse, harassment and stalking and jealous and controlling behaviour) $	8*,±
			Severity of Abuse Grid - reduction in severity: Number / percent of participants reporting a reduction of high / moderate / standard / unknown severity (reported for physical abuse, sexual abuse, harassment and stalking and jealous and controlling behaviour) $	1
			Severity of Abuse Grid – escalation in severity: Number / percent of participants reporting escalation in severity of abuse$	2
			Severity of Abuse Grid - severity: Number / percent of participants reporting any form of severe abuse$	2
			Severity of Abuse Grid - high severity: Number / percent of participants reporting severe abuse (reported for physical abuse, sexual abuse, harassment and stalking and jealous and controlling behaviour) $	1
			Severity of Abuse Grid - severity: Number / percent of participants reporting multiple types of abuse that are high severity$	6*
			Severity of Abuse Grid - severity: Number / percent of participants reporting at least one type of abuse that is high severity$	6*
			Cambridge Crime Harm Index$	3
		**Frequency of abuse$**		**14**

For peer review only

Category	Domain	Subdomain	Measure	N
			Severity of Abuse Grid - frequency: Number / percent of participants reporting high / moderate / standard / unknown frequency (reported for physical abuse, sexual abuse, harassment and stalking and jealous and controlling behaviour) [$]	5*
			Severity of Abuse Grid - frequency escalation: Number / percent of participants reporting an escalation in frequency[$]	6*
			Frequency of abusive behaviours (scale 1 = never -3 = often) [$]	1
			Frequency rating of past violence (none (1) -> frequent (10) [$]	1
			Frequency rating of current violence (none (1) -> frequent (10) [$]	1
		**Duration of abuse[$]**		**1**
			Number / percent of victims with incidents recorded over a period longer than 12 months[$]	1
		**Severity and frequency of abuse[$]**		**13**
			Severity of Abuse Grid - severity and frequency escalation: Number / percent of participants reporting any escalation in severity or frequency of abuse[$]	7*
			Severity of Abuse Grid - severity and frequency escalation: Number / percent of participants reporting at least one form of abuse that is high severity and escalating in frequency or severity[$]	6*
		**Risk[$]**		**18**
			Spousal Assault Risk Assessment[$]	2
			DASH risk (high/med/standard) [$]	2
			Drive-DASH (mean score) [$]	1
			DASH risk (number with 1-14 assessments who had a decrease in score) [$]	1
			Change in risk in perpetrators (measurement tool unclear) [$]	1
			Weighted change in risk in perpetrators (measurement tool unclear) [$]	1
			IDVA perception of change in risk (do not know / increased / limited or no reduction / moderate reduction / significant reduction) [$]	1
			IDVA rated reduction in risk (percentage reporting 'some reduction in risk') [$]	1
			IDVA perception of sustainability of change in risk (Do not know / short term / medium term / long term / risk is permanently eliminated) [$]	1
			Men's perception of risk of using violence again (never/not sure/possibly) [$]	1

For peer review only

Category	Domain	Subdomain	Measure	N
			Risk factors - Perpetrators' crimogenic behaviour and aggravating problems (frequency and percent of 7 factors) [$]	1
			Risk factors - Specific features of abuse (frequency and percent of 10 factors) [$]	1
			Risk factors - Victims' appraisals of threat (frequency and percent of 3 factors) [$]	1
			Risk factors - Factors relating to children (frequency and percent of 3 factors) [$]	1
			Risk factors - Factors relating to victim (frequency and percent of 4 factors) [$]	1
			Probability of none / standard / moderate / and high abuse (reported for physical abuse / sexual abuse / harassment and stalking / jealous and controlling behaviour) [$]	1
		**Police incidents**[$]		**39**
			Average / total number of police incidents[$]	1
			Average / total number of DV related police incidents[$]	2
			Average / total number of incidents recorded by the police per month[$]	1
			Average number of DV complaints per month[$]	1
			Average number of arrests made per month[$]	1
			Average number of victims refusing to make a complaint per month[$]	1
			Average number of mental health related DV complaints per month[$]	1
			Average number of alcohol related DV complaints per month[$]	1
			Average number of drug related DV complaints per month[$]	1
			Average number of concern for children reports per month[$]	1
			Number / percent of participants that committed DV related police incidents[$]	1
			Number / percent of participants that committed (unspecified) police incidents[$]	1
			Number / percent of participants with (unspecified) arrests[$]	1
			Number / percent of calls to domestic incidents[$]	1
			Number / percent of repeat calls to domestic incidents[$]	1
			Number / percent of repeat calls to domestic violence incidents (number with 2 incidents, 3 incidents, 4 incidents… 14 incidents) [$]	1
			Number / percent of DV incidents where the perpetrator was not present[$]	1
			Number / percent of DV incidents that were crimed[$]	1
			Number / percent of DV incidents that led to arrests[$]	2

For peer review only

Category	Domain	Subdomain	Measure	N
			Number / percent of incidents with an arrest where a statement is given$	1
			Number / percent of incidents with an arrest and a statement is given where it is then retracted$	1
			Number / percent of crimes where perpetrator was charged/summonsed$	1
			Number / percent of police incidents before and after a MARAC$	1
			Number / percent of high / medium / low severity police incidents before and after a MARAC$	1
			Number / percent of women experiencing no further post-MARAC incidents of violence or abuse$	1
			Number / percent of women experiencing no further post-MARAC police complaints or call outs$	2
			Number / percent of women experiencing no further post-MARAC police complaints$	2
			Number / percent of women experiencing no further post-MARAC police call outs$	2
			Number / percent of women experiencing either additional post-MARAC police complaints or call outs$	1
			Number / percent of women experiencing both additional post-MARAC police complaints and call outs$	1
			Percent change in total calls to domestic incidents$	1
			Percent change in total calls to domestic incidents (as proportion of all calls) $	1
			Frequency of police call outs (never / once / 2-5 times / 6-10 times) $	1
			Cost of police incidents$	1
	**Perpetration of DSVA**	**Reoffending**		**20**
			Number / percent with proven reoffending (DV, core violence or any offence)	1
			Number / percent who reoffended (police incidents)	1
			Number / percent who committed further sex offences	2
			Number / percent who reoffended (no further details)	1
			Number / percent with repeat prosecution for violence against partner	1
			Number / percent with reconviction for another sexual offence	1
			Number / percent with reconviction for another violence offence	1
			Number / percent with reconviction for any offence	2

For peer review only

Category	Domain	Subdomain	Measure	N
			Number / percent linked to another crime (suspect or charged)	1
			Number / percent linked to another DVA crime (suspect or charged)	1
			Frequency of re-arrests for DVA	1
			Prevalence of re-arrests for DVA	1
			Number / percent of crimes that that were DVA related	1
			Number of incidents of DV whilst on the programme	1
			Number of incidents of non-DV crime whilst on the programme	1
			Number / percent of partners reporting subsequent violence	1
			Number / percent of partners reporting 'frequent violence' at follow up	1
			Time to reoffend	1
		**Criminal justice outcomes**		**16**
			Average number of people charged per month	1
			Average number of cases proceeding through court per month	1
			Average number of days between arrest and case finalisation	1
			Number / percent of cases discontinued	2
			Number / percent of cases bound over / other / found not guilty or absolute discharge / convicted	1
			Number / percent of victims retracting	1
			Number / percent of trials that went ahead on scheduled date	1
			Number / percent of trials that received a conviction	1
			Number / percent of charges that received a conviction	1
			Number / percent of guilty and not guilty pleas	1
			Number / percent of defendants who had the following sentences: conditional discharge / financial penalty / custody / community punishment order / community rehabilitation order	1
			Number / percent of cases with the following convictions: custodial sentence / non-custodial sentence / conditional discharge	1
			Number / percent of evidence types in CPS files (victim statement / police statement / defendant interview / witness statement / medical statement / case exhibits / 999 tape / forensic)	1
			Improvements specific to the 'CPS Criteria for Success'	1

For peer review only

Category	Domain	Subdomain	Measure	N
			Average monetary penalties (fines and compensation)	1
		**Presence of abuse$**		**46**
			Severity of Abuse Grid - presence: Number / percent of people responding yes to the presence of abuse (reported for physical abuse, sexual abuse, harassment and stalking and jealous and controlling behaviour) $	10*
			Severity of Abuse Grid - cessation: Number / percent of people reporting no abuse$	11*
			Severity of Abuse Grid - reduction in presence: Number / percent of people reporting a reduction of abuse (reported for physical abuse, sexual abuse, harassment and stalking and jealous and controlling behaviour) $	1
			Severity of Abuse Grid - multiple types: Number / percent of people reporting multiple types of abuse$	7*
			Number / percent of participants experience concurrent domestic abuse as well as sexual abuse$	1
			Number / percent of participants who have been subjected to adult sex offences$	1
			Number / percent of men abusing each month$	1
			Space for Action Survey (% responding yes to 12 indicators) $	1
			Physical and Sexual Violence Survey (% responding yes to 7 indicators) $	1
			Harassment and other abusive acts survey (% responding yes to 6 indicators) $	1
			Fathering survey / using children against a partner (% responding yes to 5 indicators) $	2
			Social media perpetration / victimisation survey$	1
			Composite Abuse Scale$	3
			Abusive Behaviour Inventory$	2
			Controlling Behaviour Inventory$	3
		**Number of abusive incidents or behaviours$**		**4**
			Mean number of abusive incidents per man$	1
			Mean number of abusive behaviours reported by participants$	1
			Number / percent of participants experiencing between 0 and 7 behaviours (reported for emotional, physical and sexual behaviours) $	1
			IMPACT monitoring toolkit (reported for emotional, physical, and sexual behaviours) $	1
		**Severity of abuse$**		**29**

For peer review only

Category	Domain	Subdomain	Measure	N
			Severity of Abuse Grid - severity: Number / percent of participants reporting high / moderate / standard severity (reported for physical abuse, sexual abuse, harassment and stalking and jealous and controlling behaviour) $	8*,±
			Severity of Abuse Grid - reduction in severity: Number / percent of participants reporting a reduction of high / moderate / standard / unknown severity (reported for physical abuse, sexual abuse, harassment and stalking and jealous and controlling behaviour) $	1
			Severity of Abuse Grid – escalation in severity: Number / percent of participants reporting escalation in severity of abuse$	2
			Severity of Abuse Grid - severity: Number / percent of participants reporting any form of severe abuse$	2
			Severity of Abuse Grid - high severity: Number / percent of participants reporting severe abuse (reported for physical abuse, sexual abuse, harassment and stalking and jealous and controlling behaviour) $	1
			Severity of Abuse Grid - severity: Number / percent of participants reporting multiple types of abuse that are high severity$	6*
			Severity of Abuse Grid - severity: Number / percent of participants reporting at least one type of abuse that is high severity$	6*
			Cambridge Crime Harm Index$	3
		**Frequency of abuse$**		**14**
			Severity of Abuse Grid - frequency: Number / percent of participants reporting high / moderate / standard / unknown frequency (reported for physical abuse, sexual abuse, harassment and stalking and jealous and controlling behaviour) $	5*
			Severity of Abuse Grid - frequency escalation: Number / percent of participants reporting an escalation in frequency$	6*
			Frequency of abusive behaviours (scale 1 = never -3 = often) $	1
			Frequency rating of past violence (none (1) -> frequent (10) $	1
			Frequency rating of current violence (none (1) -> frequent (10) $	1
		**Duration of abuse$**		**1**
			Number / percent of victims with incidents recorded over a period longer than 12 months$	1
		**Severity and frequency of abuse$**		**13**
			Severity of Abuse Grid - severity and frequency escalation: Number / percent of participants reporting any escalation in severity or frequency of abuse$	7*
			Severity of Abuse Grid - severity and frequency escalation: Number / percent	6*

Category	Domain	Subdomain	Measure	N
			of participants reporting at least one form of abuse that is high severity and escalating in frequency or severity$	
		**Risk$**		**18**
			Spousal Assault Risk Assessment$	2
			DASH risk (high/med/standard) $	2
			Drive-DASH (mean score) $	1
			DASH risk (number with 1-14 assessments who had a decrease in score) $	1
			Change in risk in perpetrators (measurement tool unclear) $	1
			Weighted change in risk in perpetrators (measurement tool unclear) $	1
			IDVA perception of change in risk (do not know / increased / limited or no reduction / moderate reduction / significant reduction) $	1
			IDVA rated reduction in risk (percentage reporting 'some reduction in risk') $	1
			IDVA perception of sustainability of change in risk (Do not know / short term / medium term / long term / risk is permanently eliminated) $	1
			Men's perception of risk of using violence again (never/not sure/possibly) $	1
			Risk factors - Perpetrators' crimogenic behaviour and aggravating problems (frequency and percent of 7 factors) $	1
			Risk factors - Specific features of abuse (frequency and percent of 10 factors) $	1
			Risk factors - Victims' appraisals of threat (frequency and percent of 3 factors) $	1
			Risk factors - Factors relating to children (frequency and percent of 3 factors) $	1
			Risk factors - Factors relating to victim (frequency and percent of 4 factors) $	1
			Probability of none / standard / moderate / and high abuse (reported for physical abuse / sexual abuse / harassment and stalking / jealous and controlling behaviour) $	1
		**Police incidents$**		**39**
			Average / total number of police incidents$	1
			Average / total number of DV related police incidents$	2
			Average / total number of incidents recorded by the police per month$	1
			Average number of DV complaints per month$	1
			Average number of arrests made per month$	1

For peer review only

Category	Domain	Subdomain	Measure	N
			Average number of victims refusing to make a complaint per month$	1
			Average number of mental health related DV complaints per month$	1
			Average number of alcohol related DV complaints per month$	1
			Average number of drug related DV complaints per month$	1
			Average number of concern for children reports per month$	1
			Number / percent of participants that committed DV related police incidents$	1
			Number / percent of participants that committed (unspecified) police incidents$	1
			Number / percent of participants with (unspecified) arrests$	1
			Number / percent of calls to domestic incidents$	1
			Number / percent of repeat calls to domestic incidents$	1
			Number / percent of repeat calls to domestic violence incidents (number with 2 incidents, 3 incidents, 4 incidents… 14 incidents) $	1
			Number / percent of DV incidents where the perpetrator was not present$	1
			Number / percent of DV incidents that were crimed$	1
			Number / percent of DV incidents that led to arrests$	2
			Number / percent of incidents with an arrest where a statement is given$	1
			Number / percent of incidents with an arrest and a statement is given where it is then retracted$	1
			Number / percent of crimes where perpetrator was charged/summonsed$	1
			Number / percent of police incidents before and after a MARAC$	1
			Number / percent of high / medium / low severity police incidents before and after a MARAC$	1
			Number / percent of women experiencing no further post-MARAC incidents of violence or abuse$	1
			Number / percent of women experiencing no further post-MARAC police complaints or call outs$	2
			Number / percent of women experiencing no further post-MARAC police complaints$	2
			Number / percent of women experiencing no further post-MARAC police call outs$	2
			Number / percent of women experiencing either additional post-MARAC police complaints or call outs	1

For peer review only

Category	Domain	Subdomain	Measure	N
			Number / percent of women experiencing both additional post-MARAC police complaints and call outs	1
			Percent change in total calls to domestic incidents	1
			Percent change in total calls to domestic incidents (as proportion of all calls)	1
			Frequency of police call outs (never / once / 2-5 times / 6-10 times)	1
			Cost of police incidents	1

Note: Severity of Abuse Grid: there was a diverse approach to how it was used by different authors in the literature, , therefore we decided to split outcomes according to the various ways in which it was operationalised, rather than just reporting ‘Severity of Abuse Grid’ as an outcome.

* Note: Some studies do not explicitly specify using the Severity of Abuse Grid, but this has been presumed because the wording used when reporting outcomes matches exactly.

± Note: One study uses the outcome measure to assess perpetrators using abusive behaviours, rather than victim-survivor experiences of abuse.

$ Note: These outcomes and subdomains appear in more than one domain.

For peer review only

Figure 4: Temporal trends in outcome domains reported in DVSA evaluations, 1991 to 2022

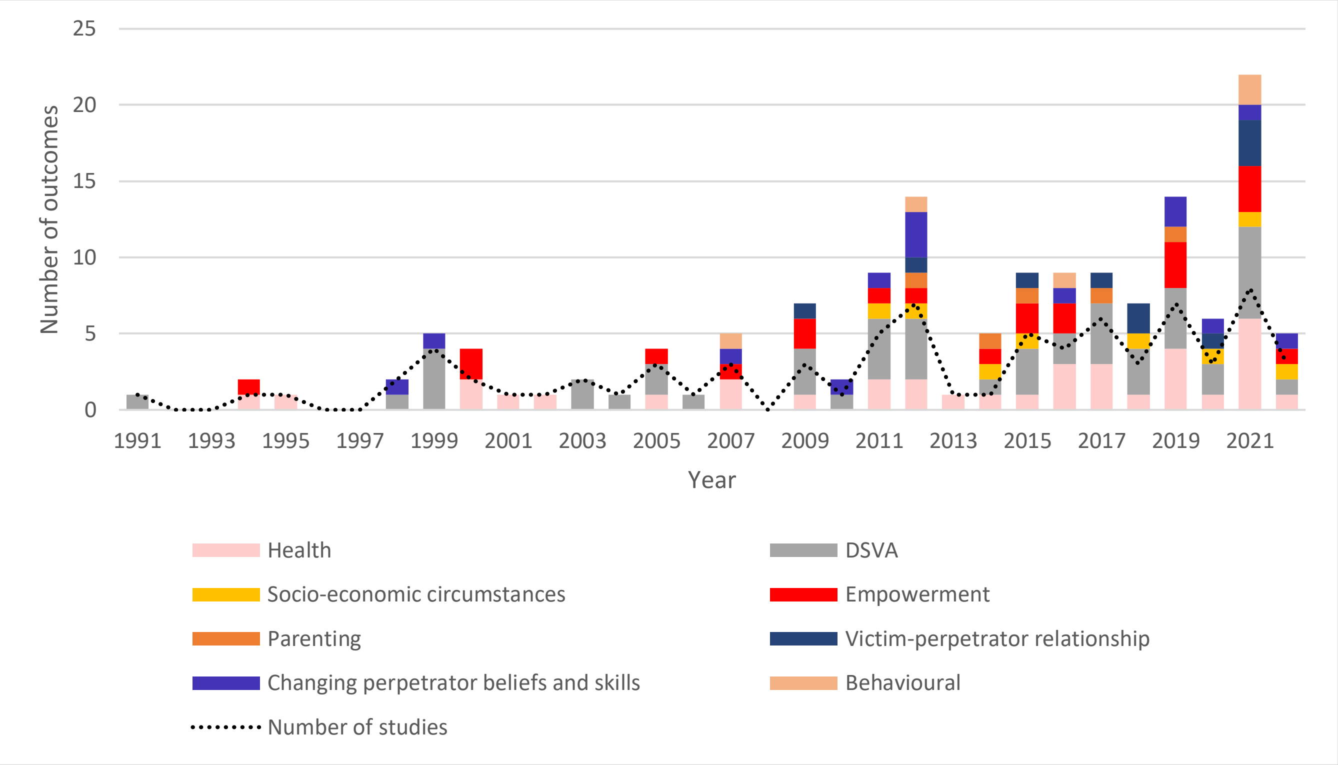

Note: For ease of interpretation, mental health, physical health and wellbeing domains have been combined into one health domain, and the experience of DSVA and perpetration of DSVA domains have been combined into a single 'DSVA' domain.
Note: Sixteen studies reported an outcome that crosses two or more domains therefore some outcomes are represented by multiple domains. Additionally, 38 studies reported an outcome that was categorised as both the experience of DSVA and perpetration of DSVA, because the specific outcome could represent either domain depending on who is completing the outcome measure.

BMJ Open

Trends in outcomes used to measure the effectiveness of UK-based support interventions and services targeted at adults with experience of domestic and sexual violence and abuse: A scoping review.

Journal:	*BMJ Open*
Manuscript ID	bmjopen-2023-074452.R3
Article Type:	Original research
Date Submitted by the Author:	18-Mar-2024
Complete List of Authors:	Carlisle, Sophie ; King's College London, Health Service and Population Research<br>Bunce, Annie; City University of London<br>Prina, Matthew; Newcastle University, Population Health Sciences Institute<br>Cook, Elizabeth; City University of London, Violence and Society Centre<br>Barbosa, Estela; University of Bristol, Population Health Sciences, Bristol Medical School; UKPRP VISION Consortium<br>McManus, Sally; City University of London; National Centre for Social Research<br>Feder, Gene; University of Bristol, Community based medicine<br>Lewis, Natalia; University of Bristol, Centre for Academic Primary Care, Population Health Sciences, Bristol Medical School; National Institute for Health Research Collaboration for Leadership in Applied Health Research and Care North Thames at Bart’s Health NHS Trust, Queen Mary University of London, Centre for Primary Care and Public Health
**Primary Subject Heading**:	Public health
Secondary Subject Heading:	Public health
Keywords:	Systematic Review, PUBLIC HEALTH, MENTAL HEALTH

SCHOLARONE™ Manuscripts

I, the Submitting Author has the right to grant and does grant on behalf of all authors of the Work (as defined in the below author licence), an exclusive licence and/or a non-exclusive licence for contributions from authors who are: i) UK Crown employees; ii) where BMJ has agreed a CC-BY licence shall apply, and/or iii) in accordance with the terms applicable for US Federal Government officers or employees acting as part of their official duties; on a worldwide, perpetual, irrevocable, royalty-free basis to BMJ Publishing Group Ltd ("BMJ") its licensees and where the relevant Journal is co-owned by BMJ to the co-owners of the Journal, to publish the Work in this journal and any other BMJ products and to exploit all rights, as set out in our licence.

The Submitting Author accepts and understands that any supply made under these terms is made by BMJ to the Submitting Author unless you are acting as an employee on behalf of your employer or a postgraduate student of an affiliated institution which is paying any applicable article publishing charge ("APC") for Open Access articles. Where the Submitting Author wishes to make the Work available on an Open Access basis (and intends to pay the relevant APC), the terms of reuse of such Open Access shall be governed by a Creative Commons licence – details of these licences and which Creative Commons licence will apply to this Work are set out in our licence referred to above.

Other than as permitted in any relevant BMJ Author's Self Archiving Policies, I confirm this Work has not been accepted for publication elsewhere, is not being considered for publication elsewhere and does not duplicate material already published. I confirm all authors consent to publication of this Work and authorise the granting of this licence.

For peer review only

Trends in outcomes used to measure the effectiveness of UK-based support interventions and services targeted at adults with experience of domestic and sexual violence and abuse: A scoping review.

Sophie Carlisle[1], Annie Bunce[3], Matthew Prina[5], Elizabeth Cook[3], Estela C Barbosa[2], Sally McManus[3,4], Gene Feder[2], Natalia V Lewis[2]

Corresponding author

Sophie Carlisle: Department of Health Service and Population Research, Institute of Psychiatry, Psychology and Neuroscience, King's College London, De Crespigny Park, London, UK. Telephone/fax: N/A. Email: Sophie.r.carlisle@kcl.ac.uk

Author affiliations

1 Department of Health Service and Population Research, Institute of Psychiatry, Psychology and Neuroscience, King's College London, De Crespigny Park, London, UK

2 Bristol Medical School, University of Bristol, Bristol BS8 2PS, UK

3 Violence and Society Centre, City, University of London, London, UK

4 National Centre for Social Research, London, UK

5 Population Health Sciences Institute, Faculty of Medical Sciences, Newcastle University, Newcastle, NE4 5PL, UK

Key words

Scoping review, domestic abuse, sexual violence, perpetrator programme, outcomes

For peer review only - http://bmjopen.bmj.com/site/about/guidelines.xhtml

Author Contributions

All authors (SC, AB, MP, EC, ECB, SM, GF, NVL) contributed to the conceptualisation and design of the review. SC carried out searches, screening, extraction, data charting, and analysis. AB was the second reviewer and contributed to the screening and extraction stages. SC produced the first draft of the manuscript and subsequent revised versions following valuable input and refinement from the co-authors (AB, MP, EC, ECB, SM, GF, NVL). All authors approved the final version.

Acknowledgements

We thank the representatives from SafeLives, Women's Aid Federation England, Respect, Rape Crisis England and Wales, Refuge and Imkaan who made up our patient and public advisory group, for their contribution to this project.

Funding statement

This research was supported by the UK Prevention Research Partnership (Violence, Health and Society; MR-VO49879/1), which is funded by the British Heart Foundation, Chief Scientist Office of the Scottish Government Health and Social Care Directorates, Engineering and Physical Sciences Research Council, Economic and Social Research Council, Health and Social Care Research and Development Division (Welsh Government), Medical Research Council, National Institute for Health and Care Research, Natural Environment Research Council, Public Health Agency (Northern Ireland), The Health Foundation, and Wellcome. The views expressed in this Article are those of the authors and not necessarily those of the UK Prevention Research Partnership or any other funder. The funder had no input in the design of the protocol or conduct of the review.

Competing interests statement

None declared.

For peer review only - http://bmjopen.bmj.com/site/about/guidelines.xhtml

Data availability statement

All data relevant to the study are included in the article and uploaded as supplementary information.

Word count: 5872

For peer review only

Abstract

Objectives: In the UK, a range of support services and interventions are available to people who have experienced or perpetrated domestic and sexual violence and abuse (DSVA). However, it is currently not clear which outcomes and outcome measures are used to assess their effectiveness. The objective of this review is to summarise, map and identify trends in outcome measures in evaluations of DSVA services and interventions in the UK.

Design: Scoping review.

Data sources: MEDLINE, EMBASE, PsycINFO, Social Policy and Practice, ASSIA, IBSS, Sociological abstracts and SSCI electronic databases were searched from inception until 21st June 2022. Grey literature sources were identified and searched.

Eligibility: We included randomised controlled trials, non-randomised comparative studies, pre-post studies and service evaluations, with at least one outcome relating to the effectiveness of the support intervention or service for people who have experienced and/or perpetrated DSVA. Outcomes had to be assessed at baseline and at least one more time-point, or compared to a comparison group.

Charting methods: Outcome measures were extracted, iteratively thematically grouped into categories, domains and subdomains, and trends were explored.

Results: 80 studies reporting 87 DSVA interventions or services were included. A total of 426 outcome measures were extracted, of which 200 were used more than once. The most commonly reported outcome subdomain was DSVA perpetration. Cessation of abuse

according to the Severity of Abuse Grid was the most common individual outcome. Analysis of temporal trends showed that the number of studies and outcomes used has increased since the 1990s.

Conclusions: Our findings highlight inconsistencies between studies in outcome measurement. The increase in the number of studies and variety of measures suggests that as evaluation of DSVA services and interventions matures, there is an increased need for a core of common, reliable metrics to aid comparability.

Protocol registration: https://osf.io/frh2e

Article summary

Strengths and limitations of this study

- This scoping review adhered to PRISMA 2020 guidelines to robustly and systematically map effectiveness outcome measures used in evaluations of support interventions and services for people who have experienced and/or perpetrated DSVA.
- It included an extensive grey literature search to be comprehensive and inclusive.
- An advisory group consisting of representatives from six third sector organisations has been involved in the development of the review.
- The review has not included services or interventions for children who have experienced DSVA, interventions aimed at staff training, and is limited to the UK.
- Further research is urgently required to work towards agreement on which outcome measures should be prioritised and adopted, whilst also avoiding burden on systems and people experiencing DSVA.

For peer review only

Introduction

Domestic and sexual violence and abuse (DSVA; comprising a range of different behaviours and experiences, see Appendix 1) represents a significant international public health issue [1-3]. Approximately 1 in 3 women and 1 in 7 men in England and Wales have experienced domestic violence and abuse in their lifetime [4]. Globally, over 35% of women have experienced either physical and/or sexual intimate partner violence or non-partner sexual violence [5]. Poverty, homelessness, and insecure immigration status heighten DSVA vulnerability [6, 7], and the risk of experiencing DSVA is also higher for younger adults, lesbian, gay, bisexual and transgender (LGBT) people [8-11], and people with long term illness, disability or mental health problems [12, 13]. The UK does not currently routinely collect data on perpetrators, however studies have found that the prevalence of DSVA perpetration ranges from 11.6-80.0% depending on country, gender and definition of DSVA [14, 15]. Perpetrators are typically male [16] and are often serial perpetrators with more than one victim [17, 18]. The impacts of DSVA are profound and diverse. As well as visible and immediate consequences such as physical injury or death, DSVA has been associated with longer-term physical health [19-25] and mental health problems [26-29]. Other areas impacted by DSVA include homelessness [30], access to finances, employment and education [31], and increased demand on the criminal justice system [32], social services [33], and police [34].

Given the wide-ranging impacts of DSVA, the support options available to people who have experienced it are diverse. Interventions and services include refuges, outreach, counselling, financial or legal advice, helplines and advocates. In the UK, these are often provided by third sector organisations (organisations that are neither public nor private sector, including voluntary and community organisations; see Appendix 1), but can also be provided by services located in the public and private sectors. Some services are targeted at particular groups with heightened vulnerability to DSVA, such as LGBT+ and Black and Minority Ethnic

For peer review only

(BME) victim-survivors or victim-survivors with disability, however provision for these groups is stark and disproportionately underfunded [35-37]. Several theoretical frameworks have been developed to explain how such support services and interventions may work. For instance, the socio-ecological model of violence against women highlights key risk factors at the individual, relationship, community and societal levels, identifying potential intervention points for preventing and responding to violence [38]. One of the risk factors identified by Heise is social isolation, therefore one component of a support intervention may be to increase social support and build relationships. Sullivan's conceptual model of DV support services [39] builds on the Conservation of Resources theory, which suggests that psychological distress following trauma is influenced by the loss of economic, social and interpersonal resources central to well-being [40-42]. Based on this, Sullivan provides an exemplar model for support services which outlines eight common programme activities aimed at creating communities that value their members and promote well-being, though intrapersonal and interpersonal changes, which impact a range of intrapersonal and interpersonal social and emotional well-being outcomes.

Previous systematic reviews of the global evidence have found benefits of such interventions on a range of social and emotional well-being related outcomes, including housing interventions improving mental health, perceived safety and stress [43], economic interventions reducing levels of domestic violence and increasing empowerment [44], advocacy interventions improving quality of life and depression [45], and psychological therapies reducing depression and anxiety [46]. Service evaluations and annual reports produced by local DSVA organisations have also found associations between service use and improved perceptions of safety, quality of life, health and wellbeing, and confidence [47-51]. Finally, reviews of perpetrator programmes (i.e., interventions targeted at people who use DSVA) have provided some evidence of reductions in the perpetration and experience of abusive behaviours, although highlighted methodological issues [52, 53].

For peer review only - http://bmjopen.bmj.com/site/about/guidelines.xhtml

Despite existing reviews identifying potential benefits of specific types of support services and interventions, there is limited understanding of which outcomes and outcome measures are currently being used both within and across UK-based DSVA support services and interventions when assessing their effectiveness [54, 55]. The specific outcome measures that are being used appear to vary, making it difficult to compare and synthesise the overall evidence [56]. Previous research in the UK and US literature that has explored outcome measurement in the DSVA field has noted the diversity of reported outcomes (e.g. [57, 58]) and highlighted various issues and difficulties surrounding outcome measurement, which contributes this diversity [59, 60]. These studies point to the differing priorities of funders and service providers, and the diversity of specific DSVA service goals and objectives, which are often multiple and complex, as key drivers behind the range of outcomes being utilised. This literature also includes discussions on what should be measured, with several pushing for the extension and diversification of outcomes and measurement strategies, potentially contributing to the diversity of outcomes being used. Identifying the most commonly utilised outcome measures will allow for meta-analysis, leading to more rigorous evaluations of the literature, and may also guide interventions and services on which outcome measures to use. Scoping reviews present an opportunity to map the evidence, and are ideal for summarising heterogenous evidence [61, 62]. This scoping review aims to summarise and map trends in the outcome measures being used to evaluate UK-based support interventions and services for DSVA.

Objectives

Specific objectives of this scoping review include:

1. Identify and thematically group outcome measures that have been used to assess the effectiveness of UK-based support interventions and services for adults who have experienced and/or perpetrated DSVA.
2. Identify which outcome measures have been used most frequently.

For peer review only - http://bmjopen.bmj.com/site/about/guidelines.xhtml

3. Explore whether the outcome measures differ by: the type of victim-survivor or perpetrator support; DSVA type; source of the study; study setting; and over time.

Review question

What outcomes relating to effectiveness have been measured in intervention studies and service evaluations of UK-based support programmes for adults who have experienced and/or perpetrated DSVA?

Methods

Protocol and registration

The protocol was developed according to the Preferred Reporting Items for Systematic Reviews and Meta-Analysis Extension for Scoping Reviews (PRISMA-ScR) [63] and published on Open Science Framework: https://osf.io/frh2e [64]. The PRISMA-ScR checklist is available in Appendix 2.

Eligibility criteria

We included any UK-based secondary or tertiary prevention support services and interventions aimed at adults who had experienced or perpetrated DSVA. Primary prevention services and interventions were excluded as they aim to prevent DSVA before it occurs, and therefore focus on individuals or populations who have not necessarily experienced violence. Interventions and services that were not specifically aimed at DSVA, such as general trauma interventions, were only included if the majority of participants (>50%) had experienced DSVA. Randomised controlled trials (RCTs), non-randomised comparative trials, pre-post studies and service evaluations that reported effectiveness outcomes at two or more time-points or made comparisons to another group were included, so that cause and effect could be inferred. Perpetrator programmes were considered for several reasons. Firstly, including them allows the measurement of DSVA without placing the burden on people who have

For peer review only - http://bmjopen.bmj.com/site/about/guidelines.xhtml

For peer review only

experienced DSVA to change someone else's behaviour. This ties in to the UK government's new perpetrator strategy [65], which intends to place the onus on perpetrators changing their behaviour, alongside the recovery of those who have experienced DSVA. Finally, perpetrator programmes provide support for DSVA perpetrators to change their behaviour, and many offer associated support to (ex)partners or referral to appropriate support. Only English language studies were eligible for inclusion. Given the focus on UK-based support services and interventions, we considered the impact of this restriction on the comprehensiveness of the review to be minimal, however this does mean that the results of this review cannot be generalised to contexts outside of the UK. Details of the full eligibility criteria can be found in Table 1.

Table 1: PICO Inclusion and Exclusion Criteria

	Inclusion	Exclusion
Population	• Adults (16 years or older) who have experienced DSVA.<br>• Adults (16 years or older) who have perpetrated DSVA.	• Children<br>• Adults who have not experienced and/or perpetrated DSVA
Intervention (and service)	• Any secondary or tertiary prevention intervention and/or service for DSVA including but not limited to: housing (e.g., refuges, housing workers, resettlements), Advocacy (e.g., Independent Domestic Violence Advisers/Advocates (IDVAs), Independent Sexual Violence Advisers/Advocates (ISVAs)), outreach, open access (e.g., helplines, drop-ins, online chats), psychological support (e.g., support groups, counselling, befriending), legal support, financial support, multi-agency risk assessment conferences (MARACs), and police-based services.<br>• Perpetrator programmes (see Appendix 1).<br>• Entry to the intervention had to be determined by the either the experience of DSVA (for victim-survivor support interventions) or perpetrating DSVA (for perpetrator programmes).	• Pharmacological<br>• Primary prevention<br>• Not primarily aimed at people with experience of DSVA

For peer review only - http://bmjopen.bmj.com/site/about/guidelines.xhtml

	Inclusion	Exclusion
Comparison	• RCTs:<br>○ Another type of included intervention or service.<br>○ Usual care<br>○ No intervention or service<br>• Pre-post designs:<br>○ Before and after the intervention or service	• No comparison
Outcome	• Any outcomes used to measure effectiveness of DSVA support services and interventions.	• Those not focussed on the effectiveness of the intervention or service (e.g., process evaluation outcomes such as satisfaction, staff training, etc)
Setting	• UK based	• Not UK based
Study design	• RCT<br>• Nonrandomised comparative<br>• Pre-post<br>• Service evaluation	• Cross-sectional, case control, case study<br>• Qualitative<br>• Letters to the editor, think pieces, descriptive only
Dates	• 1982-present	• Pre-1982

Information sources and search strategy

MEDLINE, EMBASE, PsycINFO, Social Policy and Practice, ASSIA, IBSS, Sociological abstracts and SSCI electronic databases were searched from inception until 21st June 2022. Grey literature sources were also identified, including searching electronic grey literature databases (National Grey Literature Collection, EThOS, Social Care Online and the Violence Against Women Network), a call for evidence which was shared with research networks and UK-based DSVA services and organisations, and searches of relevant UK charity and organisation websites. Finally, backwards and forwards citation tracking of all included studies, as well as reference searching of identified systematic reviews was undertaken to identify further relevant studies. The search results were exported into EndNote and duplicates were removed. An example of the search strategy for the peer review search and the grey literature search can be found in Appendix 3.

For peer review only

For peer review only - http://bmjopen.bmj.com/site/about/guidelines.xhtml

Selection of sources of evidence

The de-duplicated citations were uploaded into Rayyan [66]. Titles and abstracts, followed by full-texts, were screened according to the inclusion and exclusion criteria. A 20% sample was independently screened by a second reviewer at each stage, and all disagreements were resolved by discussion, with a third reviewer where needed. Risk of bias assessments are not necessary for scoping reviews [61].

Data charting process and data items

Data charting took place using a piloted excel spreadsheet. Data items extracted included the study citation, study design, participant demographics, description of the service or intervention and comparison (if applicable), setting, and the outcome measures used. All outcomes that were used to assess the effectiveness of DSVA support interventions and services were extracted. Data extraction and charting was carried out by one reviewer, and checked for errors by a second reviewer. Where data was missing, corresponding authors were contacted and asked to supply said data. After two weeks, if there had been no response, corresponding authors were contacted a second time and given a minimum of an additional two weeks to respond.

Synthesis of results

A narrative synthesis was conducted to summarise the included DSVA interventions and services and the effectiveness outcomes they measured. The unit of analysis was the outcome measure(s) reported. DSVA interventions and services were grouped by the type of support offered, based on the intervention and service types outlined in the protocol. Where studies reported more than one form of support as part of the intervention or service, they were classified as “multi-service”. Where the same intervention or service produced multiple reports (e.g., from different years), these were combined into one entry during data charting

For peer review only - http://bmjopen.bmj.com/site/about/guidelines.xhtml

so that all outcomes ever reported by that service were extracted, but were not double counted if multiple reports used the same outcome.

Effectiveness outcomes reported by studies were grouped thematically by type to create categories, domains and subdomains. The groupings and the names for the sub-domains, domains and categories were developed iteratively. All outcome measures were firstly listed in an excel spreadsheet. This included standardised and unstandardised questionnaires and single item measures. A column was added, describing what the outcome measured (e.g., the Beck Depression Inventory [67] measures depression). These sub-domains were then grouped into broader domains (e.g., depression is a mental health-related outcome). Where applicable, these were grouped into again even broader categories (e.g., mental health is a form of overall health).

A list of all relevant outcomes and domains, and how frequently they were reported was made. Comparisons were made between outcomes reported by studies in different sectors, over time, and by service user (i.e., perpetrator or victim-survivor) and support type (i.e., psychological support, housing, combinations), or violence type (i.e., domestic violence and abuse [DVA], sexual violence and abuse [SVA], childhood sexual abuse [CSA]).

Patient and public involvement

An advisory group was set up comprising representatives from six specialist DSVA organisations who are involved in the delivery, planning, funding or support of specialist DSVA support services in the UK. Specifically, the group included representatives from two second-tier (i.e., organisations that support front-line services but do not provide services themselves) domestic abuse organisations, one second-tier organisation for violence against BME women and girls, one domestic abuse organisation that provides a range of front-line services, one service focusing specifically on supporting male victims, working with

For peer review only - http://bmjopen.bmj.com/site/about/guidelines.xhtml

perpetrators of domestic violence, and working with young people using violence in close relationships, and one service focusing specifically on sexual violence and abuse. The group inputted to the design of the study protocol including refinement of the review question and review aims, based on their experiences of service delivery and priorities in terms of addressing knowledge gaps. The group also provided valuable contextual insight regarding the challenges in measuring the effectiveness of support services in the third sector. Their input resulted in several changes, including the broadening of the scope of this review to all effectiveness outcomes, rather than a narrower focus on only outcomes relating to reducing violence, to reflect the priorities of the sector. Provisional findings of the review were shared with the group, and their thoughts regarding the interpretation of the results were discussed, which fed into the manuscript write-up. The finalised review will be disseminated to the advisory group.

Results

Selection of sources of evidence

After duplications were removed, a total of 12431 records and 869 reports were screened for inclusion. Eighty studies from 111 reports were included in this review (see Figure 1). Data extracted from the included studies are summarised in Appendix 4, and Figure 2 [51, 68-177].

Characteristics of sources of evidence

The 80 studies described a total of 87 DSVA interventions or services, with seven studies describing both a perpetrator programme and an associated support service for (ex)partners of participating perpetrators. All studies were conducted between 1991 and 2022.

For peer review only

Description of studies

Study design

Many of the included studies were mixed-method service evaluations; for these studies, only the methodology relevant to the included outcomes is reported as the other methods do not meet the review eligibility criteria (e.g., qualitative, case studies). The most common study design was pre-post single group designs (n=53), of these 14 included additional time-points at mid-intervention or follow-up. Eight studies were RCTs, and ten used a non-randomised comparative design. For one study, the design could not be determined. Eight studies used secondary analysis of datasets, which collated data from multiple services. These were primarily publications produced by SafeLives, a UK-based charity dedicated to ending DSVA, using their Insights system, an outcome measurement tool which allows services across England and Wales to collect data according to various measurement indicators.

Perpetrator programmes

Perpetrator programmes were grouped together. They were typically described as behaviour change programmes based on a cognitive behavioural model, and whilst many included additional elements such as psychological support, these were often described in insufficient detail. Of the 80 included studies, 27 described a perpetrator programme.

Victim-survivor support interventions and services

Interventions and services providing support for those who had experienced DSVA were grouped by type of support provided, based on initial scoping of the literature. The most common were psychological support (n=21), advocacy (n=14) and multi-service (n=11). Three studies each reported on multi-agency risk assessment conferences (MARACs) and specialist domestic violence police teams. Two studies reported housing interventions or services. One study each reported on sexual violence services, a specialist domestic violence court, helpline services, outreach services, and adult health-based services. One

For peer review only

study described a women's service linked to an associated perpetrator programme, but the specific type of support offered was unclear. Several studies evaluated the effectiveness of more than one intervention or service. Of the 60 victim-survivor support interventions and services, two were specifically targeted at male victim-survivors, one at BME women, one at victims of trafficking, and one at women with disabilities. There was no evidence for interventions or services targeted specifically at LGBT+ victim-survivors.

Type of DSVA

The studies were categorised as being primarily aimed at DVA, SVA, both DVA and SVA (DSVA), or CSA. Categorisations were based on how the study or service described itself, however it should be noted that the type of violence/abuse was often not clearly defined. Thus, these categorisations should be considered as having ambiguous boundaries, and interpreted with caution.

The majority (n=53; 66.3%) of the studies were aimed at DVA, whilst 24 (29.3%) were aimed specifically at SVA. Of these, nine studies were specifically targeted at adults who had experienced CSA. Three studies (3.8%) were aimed at DSVA.

Source of the studies

Of the 80 studies and 111 reports, 41 studies from 47 reports were located in the peer-reviewed literature, and 39 studies from 64 reports were located in the grey literature.

Setting

Just under half of studies were conducted in the third sector (n=39; 47.6%). Twenty-six studies were conducted in the public sector, and 12 spanned multiple sectors. One was conducted in a privately owned prison, and for one study the setting was unclear.

For peer review only - http://bmjopen.bmj.com/site/about/guidelines.xhtml

Types of outcomes

The 80 included studies measured a total of 426 outcomes. Of these 200 (47.0%) were used more than once. In total, there were 282 unique outcomes, 57 of which were used more than once, and 226 were used only once. The number of effectiveness outcomes measured by each individual study ranged from one to 19, with a mean average of 5.23.

Outcomes were grouped thematically into 11 domains and 49 subdomains. The 11 domains included mental health, physical health, general wellbeing (all of which fall under the broad category of 'health'), the experience of DSVA and the perpetration of DSVA (which fall under the broad category of 'DSVA'), empowerment, socio-economic circumstances, behavioural outcomes, the victim-perpetrator relationship, parenting, and changing perpetrator beliefs and skills (Appendix 5).

Many of the outcome measures reported spanned two or more domains (Figure 3). For instance, 'health' as an overarching category included three domains: mental health, physical health and general wellbeing. 'Quality of life' as a subdomain spanned physical health, mental health and general wellbeing domains, based on the identified outcome measures and their specific items. For example, the 12-item Short Form (SF-12) is a measure of quality of life and general health, which includes items relating to physical health, mental health, and general wellbeing. Similarly, the overarching category of DSVA contained two domains: the experience of DSVA and the perpetration of DSVA. However, many outcome measures could fall into either domain, depending on who the respondent was (i.e., the perpetrator or the victim-survivor). For example, the outcome 'presence of abuse' would fall into the 'experience of DSVA' domain if the respondent was the victim-survivor, and the 'perpetration of DSVA' domain if the respondent was the perpetrator. Thus, there was substantial overlap between these two subdomains. Many of the outcomes within the DSVA category, whilst defined slightly differently, were not distinct. For instance, there is significant overlap between the concept of 'revictimization' and the outcome 'presence of abuse' when

For peer review only - http://bmjopen.bmj.com/site/about/guidelines.xhtml

measured using a pre-post design (if the victim-survivor indicates that they are still experiencing abuse post-intervention, then this indicates ‘revictimization’). There is similarly overlap between reoffending and revictimization (although we cannot presume a direct overlap, as a perpetrator may have multiple victims) and between ‘reoffending’ and ‘police incidents’ (although not all reoffending will be recorded by police therefore this subdomain may be broader).

For each of the 11 domains, the total number of outcomes, the total number of unique outcomes, and the number of studies reporting at least one outcome that fell within that domain, were explored. The domain ‘perpetration of abuse’ has the highest number of studies reporting at least one outcome, the most total outcome measures, and the most unique outcome measures (Appendix 6).

Of the three most common outcome measures across intervention types, all fell within the DSVA category (experience of DSVA / perpetration of DSVA; Table 2). Cessation of abuse according to the Severity of Abuse Grid (SAG) [178] was the most common outcome (n=11), followed by the presence of abuse (n=10) and the severity of abuse (n=8), both also measured by the SAG. The Rosenberg Self-Esteem Scale [179], and two more measures of DSVA from the SAG (multiple types of abuse; abuse that is escalating in severity or frequency) were each reported by seven studies.

Table 2: Most commonly reported outcome measures

Outcome	Subdomain	Domain	No. of studies
**Overall**			
1. Cessation of abuse measured by the SAG [178]	Presence of abuse	Experience of DSVA/ perpetration of DSVA	11
2. Presence of abuse measured by the SAG* [178]	Presence of abuse	Experience of DSVA/ perpetration of DSVA	10
3. Severity of abuse measured by the SAG* [178]	Severity of abuse	Experience of DSVA/ perpetration of DSVA	8
4. RSES [179]	Self-esteem	Empowerment	7
5. Multiple types of abuse measured by the SAG [178]	Presence of abuse	Experience of DSVA/ perpetration of DSVA	7

For peer review only - http://bmjopen.bmj.com/site/about/guidelines.xhtml

Outcome	Subdomain	Domain	No. of studies
6. Abuse that is escalating in severity or frequency as measured by the SAG [178]	Severity and frequency of abuse	Experience of DSVA/ perpetration of DSVA	7
**Peer review vs grey literature**			
**Grey literature**			
1. Cessation of abuse measured by the SAG [178]	Presence of abuse	Experience of DSVA/ perpetration of DSVA	9
2. Presence of abuse measured by the SAG* [178]	Presence of abuse	Experience of DSVA/ perpetration of DSVA	9
3. Severity of abuse measured by the SAG* [178]	Severity of abuse	Experience of DSVA/ perpetration of DSVA	8
**Peer review**			
1. RSES [179]	Self-esteem	Empowerment	6
2. BDI [67]	Depression	Mental health	5
3. CORE-OM [180]	Psychological issues	Mental health	5
**Sector**			
**Third**			
1. Cessation of abuse measured by the SAG [178]	Presence of abuse	Experience of DSVA/ perpetration of DSVA	9
2. Presence of abuse measured by the SAG* [178]	Presence of abuse	Experience of DSVA/ perpetration of DSVA	9
3. Severity of abuse measured by the SAG* [178]	Severity of abuse	Experience of DSVA/ perpetration of DSVA	8
**Public**			
1. BDI [67]	Depression	Mental health	5
2. RSES [179]	Self-esteem	Empowerment	4
3. SCL-90R [181]	Psychological issues	Mental health	4
**Mixed**			
1. Cases discontinued	Criminal justice outcomes	Perpetration of DSVA	2
2. PHQ [182]	Depression	Mental health	2
3. GAD-7 [183]	Anxiety	Mental health	2
4. Cambridge Crime Harm Index [184]	Severity of abuse	Experience of DSVA/ perpetration of DSVA	2
5. PC-PTSD [185]	Trauma	Mental health	2
6. No further post-MARAC police complaints or call outs	Police incidents	Experience of DSVA/ perpetration of DSVA	2
7. No further post-MARAC police complaints	Police incidents	Experience of DSVA/ perpetration of DSVA	2
8. No further post-MARAC police call outs	Police incidents	Experience of DSVA/ perpetration of DSVA	2

Note: BDI: Beck Depression Inventory; CORE-OM: Clinical Outcomes in Routine Evaluation Outcome Measure; DSVA: Domestic and sexual violence and abuse; GAD-7: Generalised Anxiety Disorder Questionnaire; MARAC: Multi-agency risk assessment conference; PHQ: Patient Health Questionnaire; PC-PTSD-5: Primary Care PTSD Screen; RSES: Rosenberg Self-Esteem Scale; SAG: Severity of Abuse Grid; SCL-90R: Symptom Checklist 90 revised.
**Reported separately for physical abuse, sexual abuse, harassment and stalking, and jealous and controlling behaviour*

For peer review only - http://bmjopen.bmj.com/site/about/guidelines.xhtml

Patterns in the use of outcome

The table in Appendix 6 summarises the patterns in outcome domains according to perpetrator programmes, victim support type, DSVA type, source of literature and setting/sector.

Outcomes in perpetrator programmes

Of the 27 perpetrator programmes, 14 reported at least one outcome relating to changing perpetrator behaviour, and 21 reported at least one relating to the perpetration of DSVA.

Outcomes by victim support type

The most common outcome domains for psychological support interventions and services were mental health (n=17) and empowerment (n=10), whilst advocacy, multi-service interventions and studies of MARACs primarily reported on experience of DSVA (n=12, n=8 and n=3, respectively). Specialist DV police teams reported equally on the experience of and the perpetration of DSVA (n=2 each).

Outcomes by type of DSVA

Of the studies primarily focused on DSVA, the majority reported at least one outcome relating to the experience of DSVA (n=40; 75.5%) and the perpetration of DSVA (n=41; 77.4%). Just under half of studies of adult SVA (n=7; 46.7%), and all studies of CSA, reported mental health outcomes (n=9; 100%).

Outcomes by source of studies

Whilst the split of included studies from the grey literature and peer reviewed literature was relatively even, studies located within the grey literature were more diverse. In total, grey literature studies reported 254 outcome measures, 178 of which were unique. In comparison, peer reviewed studies reported a total of 172 outcomes, 125 of which were

For peer review only - http://bmjopen.bmj.com/site/about/guidelines.xhtml

For peer review only

unique. Peer reviewed studies had more of a tendency to report at least one outcome relating to mental health (n=22; 53.7%), whilst grey literature studies were more likely to report outcomes relating to the experience of DSVA (n=29; 74.4%).

Outcomes by setting

Two thirds of studies set in the third sector reported outcomes relating to the experience of DSVA subdomain (n=27; 69.2%). Perpetration of DSVA was the most common outcome subdomain for interventions and services with a mixed setting (n=11; 91.7%), whilst mental health was the most common subdomain for those in the public sector (n=13; 50%).

Outcomes 1991 to 2022

Evaluations of DSVA support services and interventions have become more frequent over time. Nearly half (48.8%) of included studies were published in 2015-2022, with over a quarter (26.3%) published in 2019-2022 (Appendix 7).

Discussion

This study used scoping review methodology to map which outcomes have been used in studies assessing the effectiveness of a range of support interventions and services for people who have experienced or perpetrated DSVA. A total of 426 outcome measures were used in 80 papers and reports, describing 87 DSVA interventions and services. Less than half of the measures were used more than once.

The most common outcome domain was the perpetration of DSVA, followed closely by the experience of DSVA, both of which included outcomes relating directly to abusive and violent behaviours. This demonstrates that the measurement of DSVA has been prioritised when assessing service and intervention effectiveness, and is consistent with the common goal of

For peer review only - http://bmjopen.bmj.com/site/about/guidelines.xhtml

many DSVA support services and organisations of ending violence and abuse [186, 187]. However, 201 and 189 unique outcome measures were charted within these two domains respectively, showing the lack of consensus regarding the specific outcomes that should be measured. The SAG, which measures several different aspects of abusive behaviours (including presence of four types of abuse, frequency, severity, and cessation of abuse), was the most frequently used outcome measure. It was primarily used by studies drawing upon the SafeLives' Insights datasets, although it was employed by several additional studies too. Despite being the most frequently reported outcome, the SAG was still utilised by only 13 of the 80 studies, illustrating the inconsistencies and variation in measures being used by these evaluations and reports.

Of the 282 unique outcomes reported, 226 were used only once. Although many of these outcomes were similar, they often differed slightly according to the unit or method of measurement, meaning that combining them would be methodologically inappropriate. This further demonstrates the disparate and highly variable nature of outcome measures currently in use. Whilst this may be partly explained by the fact that different sectors and types of support interventions have different priorities, such disparity is evident even *within* sectors and intervention types. For instance, of the 153 different outcome measures reported by third sector studies, only 24.8% were used more than once, with a similarly low proportion in mixed sector and public sector studies (both 30.7%). This indicates a lack of comparability surrounding the best way to assess the effectiveness of support services and interventions, and makes synthesis such as meta-analysis less feasible.

Mapping revealed differences between the types of outcome domains frequently reported in the peer reviewed and grey literature, with peer reviewed studies focusing more on mental health related outcomes, and grey literature studies focusing primarily on DSVA. This difference may be explained by the overlap between grey literature and third sector-based studies, and the fact that third sector organisations are heavily tied to the outcomes that

For peer review only - http://bmjopen.bmj.com/site/about/guidelines.xhtml

funders and commissioners require them to measure. With the focus on wanting to establish and reduce the prevalence of DSVA itself, and the associated focus on the perpetration of violence, it might be that more funders and commissioners are requiring these to be measured, or are prioritising these measurements above the wider impacts of DSVA.

Mapping of trends over time showed that as well as a general increase in the number of studies being conducted, there appeared to be increased diversity in outcomes measured. The two most common outcome categories, DSVA and health, have been measured fairly consistently over the past thirty years, whilst domains such as empowerment, the victim-perpetrator relationship and parenting have become more common in the past decade. This is likely reflective of an increased knowledge of the wide-ranging impacts of DSVA and its ripple effects, increased recognition of the need for holistic support, and the move towards survivor-centred outcome measurement and consultation with those with lived experience [188].

Overall, this review has demonstrated that the types of outcomes measured when assessing the effectiveness of support interventions and services for people who have experienced DSVA vary widely. There are several potential reasons for this diversity in outcome measures. Firstly, as previously mentioned, DSVA has many impacts, including on physical health, mental health, housing, finances, relationships, and many more. Therefore, there are many avenues through which support services and interventions could have impact and improve outcomes. Indeed, in their review Sprague, McKay [57] noted that many DSVA services have multiple goals that cannot be assessed by a single outcome, thus multiple outcomes are required. Secondly, differences between the priorities of funders, services and service users, and researchers in terms of outcome measurement likely results in diversity in outcomes and differences in outcome measures utilised between sectors [58, 59]. Thirdly, there is currently a lack of consensus across the field regarding what outcomes should be

For peer review only - http://bmjopen.bmj.com/site/about/guidelines.xhtml

measured and, importantly, how. For instance, while it may be agreed that 'safety' is an important outcome to assess, how this is operationalised and measured is not consistent.

Challenges in outcome measurement

There are two linked challenges facing UK-based DSVA services, especially those located in the third sector. Firstly, funding of DSVA services is precarious and diminishing [189-192]. Being awarded funding requires evidence of effectiveness. However, the second challenge is that carrying out service evaluations to provide evidence of effectiveness is costly, making it even more difficult for services to secure funding. This is reflected in take-up of the aforementioned SafeLives' Insights system, which captures rich data and has likely contributed to more consistent reporting of outcomes across England and Wales. However, individual services have to pay to use the system, and the numbers of services contributing to the dataset each year has reduced, likely due to reduced funding [consultation with stakeholder advisory group].

Limitations

There are limitations of this review associated with the scoping review methodology. For instance, scoping reviews prioritise breadth over depth. The primary objectives of this review were to map and chart outcome measures being used (i.e., the breadth of outcome literature), and so we have not made any assessments regarding the validity or relevance of any particular measure. Therefore, whilst we have identified the most commonly used outcome measures, that is not to say that these are the most helpful, informative or important to people with lived experience or to front-line service providers. Further, in line with scoping review methodology, we have not completed risk of bias assessments for any of the included studies, therefore we cannot comment on the quality of these studies. A further limitation of this review is the unavoidable potential for double counting. For instance, the services that are included in SafeLives' Insights datasets are anonymous. Thus, it is not

For peer review only - http://bmjopen.bmj.com/site/about/guidelines.xhtml

possible to determine if any services included in the Insights datasets have also published an independent evaluation. Another limitation of this review is that despite a comprehensive search strategy, no evaluations of support services run by by-and-for organisations were identified (see Appendix 1). This is illustrative of a systemic, cyclical and enduring issue facing these smaller services in particular, who are disproportionally underfunded and lack the resources needed to conduct and publish evaluations, meaning they are underrepresented in the literature [36, 193-195]. Further, despite the comprehensive search strategy which was designed in collaboration with the advisory group to ensure key search terms were included, the search was not exhaustive, and therefore it is possible that had we selected different databases or included additional terms, we may have identified additional relevant papers. Additionally, because of time and resource restrictions, only 20% of records were dual screened during the title/abstract and full text screening stages, which does increase the risk of potential bias in the selection of studies and the risk of relevant studies being incorrectly excluded. Finally, we excluded process evaluation outcomes. Given that there may be causal links between process measures and distal outcomes, it is possible that some relevant outcomes were missed.

Implications and recommendations

The findings from this scoping review regarding outcomes most commonly measured to assess the effectiveness of DSVA interventions highlight how support services are working to promote survivors' safety and wellbeing. However, the diversity of the specific outcomes and measurement tools used has implications for researchers, service providers, policymakers and funders.

Implications and recommendations for researchers

Increased consensus between researchers, service providers, policymakers and funders is needed. This will allow for more meaningful syntheses of the literature, as well as building a larger evidence base, so that a better understanding of the most effective means of support

For peer review only - http://bmjopen.bmj.com/site/about/guidelines.xhtml

for people who have experienced DSVA can be reached. Thus, further research is needed to extend ongoing work on the development of shared outcome frameworks, to determine which outcomes and outcome measures are most appropriate, valid and relevant, in order to work towards a consensus and build a shared evidence base to enable future meta-analyses. These outcome frameworks should be underpinned by programme theories. We hope our findings will inspire further conversation and exploration of what to measure, when and with whom, and provide guidance to researchers, service providers and funders striving to strike the balance between reaching a shared consensus, and selecting outcomes according to important contextual factors such as the (often multiple and complex) goals of the intervention, the timing of evaluation, and the study population, for example. This work should be carried out as a collaboration between people who have experienced abuse and their families, service providers and, in the UK, commissioners, to ensure researchers and evaluators are measuring the outcomes that matter most to the people who matter most. Such approaches have previously been used to develop outcome frameworks in related areas [56]. This work should also consider whether the outcomes and outcome measures most appropriate and relevant vary by different characteristics or populations, such as male victim-survivors, BME populations, or those from the LGBT+ community. Any outcome frameworks that are developed should consider this and be adaptable to the different target populations of various services.

Implications and recommendations for service providers

Whilst we recognise that service providers working in the DSVA field are often over-burdened and under-resourced, and doing the best they can with what they have, we would encourage service providers to use the findings from this review to consider their current practices for outcome measurement and the how’s and why’s underlying them. If resource allows, organisations could assess whether the outcome measurement tools currently being used are fit for purpose and reflect recent shifts in definitions of and responses to domestic abuse, and explore opportunities for improvement and innovation. In the absence of

For peer review only - http://bmjopen.bmj.com/site/about/guidelines.xhtml

additional resource, service providers should continue to record and monitor the various service- and individual-level outcomes they are required to measure as accurately and consistently as is possible whilst delivering frontline services.

Implications and recommendations for policymakers

Ultimately, the extent to which service providers can apply research findings to practice and use them to drive improvements to services depends heavily upon policymakers' decisions relating to the provision and funding of services, and monitoring and evaluation. Currently, patchwork and piecemeal funding [36, 196] and inconsistent commissioning frameworks contribute to the diversity of outcomes measured and underpin a competitive funding landscape that is not conducive to achieving consensus across the sector. Service providers working to end DSVA have been campaigning for greater consistency in the commissioning of services for many years, and their voices should be used by policymakers to enact meaningful change. Based on discussions with our advisory group throughout the review process, we would suggest that policymakers focus on ensuring any core outcomes framework is implementable on a practical level. Service providers in low resource settings likely have less scope for this than others, and it is crucial that any core outcome measurement tools are not too time-consuming for service providers to use and complete. Additionally, policymakers and commissioners should look beyond blunt, immediately measurable outcomes (e.g. immediate safety, prosecution and conviction rates) and recognise the value of other outcomes (e.g. social and emotional wellbeing), which may take longer to measure but be a more accurate indication of real change. A suggested avenue for working towards this would be to run a national survivor consultation to draw out what is the most meaningful and/or has made the most difference for survivors. Key findings from this could then be mapped onto the outcomes prioritised by policymakers, service providers and funders, to identify where there are overlaps and where there are disparities that need addressing.

For peer review only - http://bmjopen.bmj.com/site/about/guidelines.xhtml

Conclusions

A broad range of outcome measures have been used to assess the effectiveness of support interventions for people who have experienced or perpetrated DSVA. There is growing recognition across various sectors of the potential benefits of a core set of outcomes, which include increasing consistency and consensus [197, 198]. We need to take stock of the current state of the evidence and reconceptualise what a core set of outcomes should look like across and within sectors. This core outcome set needs to be co-produced by a range of stakeholders, including those with experience of DSVA and those providing front line support. For this to be feasible a trade-off needs to occur. Burden needs to be minimised by not requiring excessive data collection, but instead, collection of only the most relevant outcome measures according to the specific purpose of the intervention or service. At the same time, within-service consistency needs to be increased, as well as supporting comparability across sectors. Further research is needed to better understand which outcomes should be prioritised in order to strike this balance.

For peer review only

Figure legends:

Figure 1. PRISMA flow diagram

Figure 2. Bar chart to show the percentage of included studies with each categorisation type.

Figure 3. Venn diagram of outcome categories, domains and subdomains

Ethical approval statement: Not applicable as this was a scoping review.

For peer review only

For peer review only - http://bmjopen.bmj.com/site/about/guidelines.xhtml

References

1. UK Faculty of Public Health, *The role of public health in the prevention of violence*. 2016.
2. Savage-Borne, J. and A. Maitra. *Intimate partner violence as a public health problem*. 2021; Available from: https://blogs.bmj.com/bmj/2021/03/25/intimate-partner-violence-as-a-public-health-problem/.
3. World Health Organization. *Voilence against women*. 2021; Available from: https://www.who.int/news-room/fact-sheets/detail/violence-against-women.
4. Office for National Statistics (ONS), *Domestic abuse prevalence and victim characteristics Year ending March 2022*, Office for National Statistics (ONS), Editor. 2022.
5. World Health Organization, *Global and regional estimates of violence against women: prevalence and health effects of intimate partner violence and non-partner sexual violence*. 2013: World Health Organization.
6. Femi-Ajao, O., S. Kendal, and K. Lovell, *A qualitative systematic review of published work on disclosure and help-seeking for domestic violence and abuse among women from ethnic minority populations in the UK.* Ethnicity & Health, 2020. **25**(5): p. 732-746.
7. AVA, A., *Breaking down the barriers: findings of the national commission on domestic and sexual violence and multiple disadvantage*. 2020.
8. Donovan, C., et al., *Comparing domestic abuse in same sex and heterosexual relationships.* Unpublished manuscript, University of Sunderland, Sunderland, England, and University of Bristol, Bristol, England, 2006.
9. Roch, A., G. Ritchie, and J. Morton, *Transgender people's experiences of domestic abuse.* Scotland: LGBT Youth Scotland and the Equality Network, 2010.
10. Valentine, S.E., et al., *Disparities in exposure to intimate partner violence among transgender/gender nonconforming and sexual minority primary care patients.* LGBT health, 2017. **4**(4): p. 260-267.
11. Langenderfer-Magruder, L., et al., *Experiences of intimate partner violence and subsequent police reporting among lesbian, gay, bisexual, transgender, and queer adults in Colorado: Comparing rates of cisgender and transgender victimization.* Journal of interpersonal violence, 2016. **31**(5): p. 855-871.
12. Khalifeh, H., et al., *Violence against people with disability in England and Wales: findings from a national cross-sectional survey.* PloS one, 2013. **8**(2): p. e55952.
13. Trevillion, K., et al., *Experiences of domestic violence and mental disorders: a systematic review and meta-analysis.* PloS one, 2012. **7**(12): p. e51740.
14. Fulu, E., et al., *Prevalence of and factors associated with male perpetration of intimate partner violence: findings from the UN Multi-country Cross-sectional Study on Men and Violence in Asia and the Pacific.* The lancet global health, 2013. **1**(4): p. e187-e207.
15. Herbert, A., et al., *Risk factors for intimate partner violence and abuse among adolescents and young adults: findings from a UK population-based cohort.* Wellcome open research, 2020. **5**.
16. Hester, M., *Who does what to whom?: Gender and domestic violence perpetrators*. 2009: University of Bristol [in association with] Northern Rock Foundation Bristol.
17. Robinson, A.L., *Serial domestic abuse in Wales: an exploratory study into its definition, prevalence, correlates, and management.* Victims & Offenders, 2017. **12**(5): p. 643-662.
18. Gilchrist, G., et al., *The prevalence and factors associated with ever perpetrating intimate partner violence by men receiving substance use treatment in Brazil and England: A cross-cultural comparison.* Drug and Alcohol Review, 2017. **36**(1): p. 34-51.
19. Basile, K.C., et al., *National intimate partner and sexual violence survey: 2010 summary report.* 2011.

For peer review only - http://bmjopen.bmj.com/site/about/guidelines.xhtml

20. Breiding, M.J., M.C. Black, and G.W. Ryan, *Chronic disease and health risk behaviors associated with intimate partner violence—18 US states/territories, 2005.* Annals of epidemiology, 2008. **18**(7): p. 538-544.
21. Chandan, J.S., et al., *Risk of cardiometabolic disease and all-cause mortality in female survivors of domestic abuse.* Journal of the American Heart Association, 2020. **9**(4): p. e014580.
22. Crofford, L.J., *Violence, stress, and somatic syndromes.* Trauma, Violence, & Abuse, 2007. **8**(3): p. 299-313.
23. John, R., et al., *Domestic violence: prevalence and association with gynaecological symptoms.* BJOG: An International Journal of Obstetrics & Gynaecology, 2004. **111**(10): p. 1128-1132.
24. Leserman, J. and D.A. Drossman, *Relationship of abuse history to functional gastrointestinal disorders and symptoms: some possible mediating mechanisms.* Trauma, Violence, & Abuse, 2007. **8**(3): p. 331-343.
25. Tjaden, P. and N. Thoennes, *Prevalence and consequences of male-to-female and female-to-male intimate partner violence as measured by the National Violence Against Women Survey.* Violence against women, 2000. **6**(2): p. 142-161.
26. McManus, S., et al., *Receiving threatening or obscene messages from a partner and mental health, self-harm and suicidality: results from the Adult Psychiatric Morbidity Survey.* Social psychiatry and psychiatric epidemiology, 2021: p. 1-11.
27. Povey, D., et al., *Homicides, Firearm Offences and Intimate Violence 2006/07 (Supplementary Volume 2 to Crime in England and Wales 2006/07) Home Office Statistical Bulletin 03/08 [online]. London: Home Office Research Development and Statistics Directorate*. 2008.
28. McManus, S., et al., *Intimate Partner Violence, Suicidality, and Self-Harm: A Probability Sample Survey of the General Population in England.* Suicidality, and Self-Harm: A Probability Sample Survey of the General Population in England, 2022.
29. Jonas, S., et al., *Gender differences in intimate partner violence and psychiatric disorders in England: results from the 2007 adult psychiatric morbidity survey.* Epidemiology and psychiatric sciences, 2014. **23**(2): p. 189-199.
30. St. Mungo's, *Rebuilding Shattered Lives [online]*. 2014.
31. Women's Aid, *The Domestic Abuse Report 2019: The Economics of Abuse Report Summary*. 2019.
32. Crown Prosecution Service, *Violence against Women and Girls Report 10th Edition 2016-17*. 2017.
33. Office for National Statistics (ONS), *Domestic abuse and the criminal justice system*. 2021.
34. ONS, *Domestic abuse in England and Wales overview: November 2021. Figures on domestic abuse from police recorded crime and a number of different organisations.* 2021.
35. Thiara, R.K., G. Hague, and A. Mullender, *Losing out on both counts: disabled women and domestic violence.* Disability & Society, 2011. **26**(6): p. 757-771.
36. Imkaan, *From Survival to Sustainability: Critical Issues for the Specialist Black and 'Minority Ethnic'Ending Violence Against Women and Girls Sector in the UK*. 2018, Imkaan London.
37. Colgan, F., C. Hunter, and A. McKearney, *'Staying alive': The impact of 'austerity cuts' on the LGBT Voluntary and Community Sector (VCS) in England and Wales*. 2014, TUC.
38. Heise, L.L., *Violence against women: An integrated, ecological framework.* Violence against women, 1998. **4**(3): p. 262-290.
39. Sullivan, C.M., *Understanding how domestic violence support services promote survivor well-being: A conceptual model.* Journal of family violence, 2018. **33**(2): p. 123-131.
40. Hobfoll, S., *Stress[illegible]culture[illegible]and community: the psychology and philosophy of stress*. 1998, New York: Plenum Press[illegible].
41. Hobfoll, S.E., *Conservation of resources: A new attempt at conceptualizing stress.* American psychologist, 1989. **44**(3): p. 513.

For peer review only - http://bmjopen.bmj.com/site/about/guidelines.xhtml

42. Hobfoll, S.E., *The influence of culture, community, and the nested-self in the stress process: Advancing conservation of resources theory.* Applied psychology, 2001. **50**(3): p. 337-421.
43. Yakubovich, A.R., et al., *Housing interventions for women experiencing intimate partner violence: a systematic review.* The Lancet Public Health, 2021.
44. Kiani, Z., et al., *A systematic review: Empowerment interventions to reduce domestic violence?* Aggression and violent behavior, 2021. **58**: p. 101585.
45. Rivas, C., et al., *Advocacy interventions to reduce or eliminate violence and promote the physical and psychosocial well-being of women who experience intimate partner abuse: A systematic review.* Campbell Systematic Reviews, 2016. **12**(1): p. 1-202.
46. Hameed, M., et al., *Psychological therapies for women who experience intimate partner violence.* Cochrane database of systematic reviews, 2020(7).
47. Advance, *Annual Report and Accounts*. 2021.
48. Refuge, *Annual Report 2020–2021 and financial statements*. 2021.
49. Yellow Door, *Annual Review and Accounts 2020-2021*. 2021.
50. Oasis, *Impact*. 2019.
51. Holly Taylor-Dunn and Rosie Erol, *Evaluation of SaferPlaces' Independent Domestic Violence Advisor Services.* 2019.
52. Lilley-Walker, S.-J., M. Hester, and W. Turner, *Evaluation of European domestic violence perpetrator programmes: Toward a model for designing and reporting evaluations related to perpetrator treatment interventions.* International journal of offender therapy and comparative criminology, 2018. **62**(4): p. 868-884.
53. Akoensi, T.D., et al., *Domestic violence perpetrator programs in Europe, Part II: A systematic review of the state of evidence.* International journal of offender therapy and comparative criminology, 2013. **57**(10): p. 1206-1225.
54. National Institute for Health and Care Excellence. *Domestic violence and abuse: multi-agency working: Public health guideline [PH50]. Recommendations for research*. 2014; Available from: https://www.nice.org.uk/guidance/ph50/chapter/5-Recommendations-for-research.
55. James Lind Alliance. *Sexual Violence Top 10*. 2022; Available from: https://www.jla.nihr.ac.uk/priority-setting-partnerships/sexual-violence/top-10-priorities.htm.
56. Powell, C., et al., *Development of core outcome sets for family and child-focused interventions for child maltreatment and domestic violence*. 2021.
57. Sprague, S., et al., *Outcome measures for evaluating intimate partner violence programs within clinical settings: A systematic review of the literature.* Trauma, Violence, & Abuse, 2017. **18**(5): p. 508-522.
58. Clark, S.E., et al., *Engaging domestic abuse practitioners and survivors in a review of outcome tools–reflections on differing priorities.* Research for All, 2023. **7**(1).
59. Sullivan, C.M., *Evaluating domestic violence support service programs: Waste of time, necessary evil, or opportunity for growth?* Aggression and Violent Behavior, 2011. **16**(4): p. 354-360.
60. Grych, J. and S. Hamby, *Advancing the measurement of violence: Challenges and opportunities.* Psychology of Violence, 2014. **4**(4): p. 363.
61. Munn, Z., et al., *Systematic review or scoping review? Guidance for authors when choosing between a systematic or scoping review approach.* BMC medical research methodology, 2018. **18**: p. 1-7.
62. Peterson, J., et al., *Understanding scoping reviews: Definition, purpose, and process.* Journal of the American Association of Nurse Practitioners, 2017. **29**(1): p. 12-16.
63. Tricco, A.C., et al., *PRISMA extension for scoping reviews (PRISMA-ScR): checklist and explanation.* Annals of internal medicine, 2018. **169**(7): p. 467-473.

For peer review only - http://bmjopen.bmj.com/site/about/guidelines.xhtml

64. Carlisle, S., et al. *Measuring the effectiveness of UK-based support interventions and/or services targeted at adults who have experienced domestic and sexual violence and abuse (DSVA): A scoping review*. 2022; Available from: https://osf.io/frh2e.
65. Home Office, *Policy Paper: Tackling perpetrators*. 2022.
66. Ouzzani, M., et al., *Rayyan — a web and mobile app for systematic reviews.* Systematic Reviews, 2016. **5**(210).
67. Beck, A.T., R.A. Steer, and G.K. Brown, *Beck depression inventory*. 1987: Harcourt Brace Jovanovich New York:.
68. ADVA. and S.P. Associates., *REPAIR (Resolve to End the Perpetration of Abuse in Relationships): A Community- and Whole-family-based Intervention Programme Targeting Perpetrators of Domestic Violence and Abuse in Devon. An evaluation of a three-year Invest to Save (ISB) PROJECT*. 2009: Exeter.
69. Advance, *'A Place to go Like This': Breaking the cycle of harm for mothers involved in offending who are survivors of domestic abuse, and their children.* 2020.
70. Barber, M., R. Jenkins, and C. Jones, *A survivor's group for women who have a learning disability.* British Journal of Developmental Disabilities, 2000. **46**(90, Pt 1): p. 31-41.
71. Bloomfield, S. and L. Dixon, *An outcome evaluation of the integrated domestic abuse programme (IDAP) and community domestic violence programme (CDVP).* London: National Offender Management Service, 2015.
72. Bowen, E., *Evaluation of a community based domestic violence offender rehabilitation programme. Unpublished doctoral thesis*, in *Centre for Forensic and Family Psychology*. 2004, University of Birmingham.
73. Bowen, E., *Therapeutic Environment and Outcomes in a UK Domestic Violence Perpetrator Program.* Small Group Research, 2010. **41**(2): p. 198-220.
74. Bowen, E. and E. Gilchrist, *Predicting dropout of court-mandated treatment in a British sample of domestic violence offenders.* Psychology, Crime & Law, 2006. **12**(5): p. 573-587.
75. Bowen, E., E. Gilchrist, and A.R. Beech, *Change in treatment has no relationship with subsequent re-offending in UK domestic violence sample: A preliminary study.* International Journal of Offender Therapy and Comparative Criminology, 2008. **52**(5): p. 598-614.
76. Bowen, E., E.A. Gilchrist, and A.R. Beech, *An Examination of the Impact of Community-Based Rehabilitation on the Offending Behaviour of Male Domestic Violence Offenders and the Characteristics Associated with Recidivism.* Legal and Criminological Psychology, 2005. **10**(2): p. 189-209.
77. Brady, F., et al., *Narrative exposure therapy for survivors of human trafficking: feasibility randomised controlled trial.* BJPsych Open, 2021. **7**(6).
78. Burton, S., L. Regan, and L. Kelly, *Supporting women and challenging men: lessons from the Domestic Violence Intervention Project.* 1998.
79. Callaghan, J., et al., *Make a Change: An evaluation of the implementation of an early response intervention for those who have used abusive behaviours in their intimate relationships*. 2020.
80. Respect and Women's Aid Federation England, *Make a Change Executive Summary*.
81. Calvert, R., S. Kellett, and T. Hagan, *Group cognitive analytic therapy for female survivors of childhood sexual abuse.* British Journal of Clinical Psychology, 2015. **54**(4): p. 391-413.
82. Clarke, S. and S. Llewelyn, *Personal constructs of survivors of childhood sexual abuse receiving cognitive analytic therapy.* British Journal of Medical Psychology, 1994. **67(3)**: p. 273-289.
83. Clarke, S. and C. Pearson, *Personal constructs of male survivors of childhood sexual abuse receiving cognitive analytic therapy.* British Journal of Medical Psychology, 2000. **73(2)**: p. 169-177.
84. Cook, D.A., et al., *The Berkeley group: ten years' experience of a group for non-violent sex offenders.* The British Journal of Psychiatry, 1991. **158**(2): p. 238-243.

For peer review only - http://bmjopen.bmj.com/site/about/guidelines.xhtml

85. Co-Ordinated Action Against Domestic, A., *Insights into domestic abuse 1: a place of greater safety.* 2012.
86. Co-ordinated Action Against Domestic Abuse (CAADA), *Insights National Dataset 2011–12 Appendix to: A place of greater safety*. 2012.
87. Coy, M. and L. Kelly, *Islands in the stream: an evaluation of four London independent domestic violence advocacy schemes*. 2011.
88. Dheensa, S., G. Penny, and Safelink, *"In my way…" Evaluation Of Learning Disabilities Sexual Abuse Support Services*.
89. Dobash, R.P., et al., *A research evaluation of British programmes for violent men.* Journal of Social Policy, 1999. **28**(2): p. 205-233.
90. Ellis, F., *Rehabilitation programme for adult survivors of childhood sexual abuse.* Journal of public mental health, 2012. **11**(2): p. 88-92.
91. Farrell, G. and A. Buckley, *Evaluation of a UK police domestic violence unit using repeat victimisation as a performance indicator.* The Howard Journal of Criminal Justice, 1999. **38**(1): p. 42-53.
92. Ferrari, G., et al., *Psychological advocacy towards healing (PATH): A randomized controlled trial of a psychological intervention in a domestic violence service setting.* Plos One, 2018. **13**(11).
93. Friendship, C., R.E. Mann, and A.R. Beech, *Evaluation of a national prison-based treatment program for sexual offenders in England and Wales.* Journal of interpersonal violence, 2003. **18**(7): p. 744-759.
94. Garry, K. and P. Goodwin, *Adult Counselling Service Phase two evaluation report: year 1 2020-21*. 2021, Merida Associates.
95. Karen Garry and Ellie Munro, *Adult Counselling Service Interim evaluation | May 2019*. 2019.
96. Karen Garry and Ellie Munro, *Adult Counselling Service Evaluation report – phase one 2016-2020*. 2020.
97. Geoghegan-Fittall, S., C. Keeble, and D. Wunsch, *The Croydon Drive Project: A 2-year Evaluation Final Report*. 2020.
98. Gilchrist, E., et al., *Using the Behaviour Change Wheel to design an intervention for partner abusive men in drug and alcohol treatment.* Pilot and feasibility studies, 2021. **7**(1): p. 1-14.
99. Gilchrist, G., et al., *ADVANCE integrated group intervention to address both substance use and intimate partner abuse perpetration by men in substance use treatment: a feasibility randomised controlled trial.* BMC public health, 2021. **21**(1): p. 1-20.
100. Goosey, J., L. Sherman, and P. Neyroud, *Integrated case management of repeated intimate partner violence: A randomized, controlled trial.* Cambridge Journal of Evidence-Based Policing, 2017. **1**(2): p. 174-189.
101. Halliwell, G., et al., *Cry for health: a quantitative evaluation of a hospital-based advocacy intervention for domestic violence and abuse.* Bmc Health Services Research, 2019. **19**(1).
102. Hester, M., et al., *Evaluation of Year 2 of the Drive Project – A Pilot to Address High Risk Perpetrators of Domestic Abuse*. 2019.
103. Hester, M., et al., *Evaluation of the Drive Project – a pilot to address high-risk perpetrators of domestic abuse Year 1 Feasibility Study*. 2017.
104. Hester, M., et al., *Evaluation of the Drive Project – A Three-year Pilot to Address High-risk, High-harm Perpetrators of Domestic Abuse*. 2019.
105. Hester, M., et al., *Evaluation of the Drive Project – A Three-year Pilot to Address High-risk, High-harm Perpetrators of Domestic Abuse EXECUTIVE SUMMARY.* 2020.
106. Hester, M. and N. Westmarland, *Tackling domestic violence: effective interventions and approaches*. 2005: Home Office Research, Development and Statistics Directorate.
107. Howarth, E. and et al., *Safety in numbers: a multi-site evaluation of Independent Domestic Violence Advisor Services.* 2009.

For peer review only - http://bmjopen.bmj.com/site/about/guidelines.xhtml

108. Howarth, E., et al., *Safety in numbers: summary of findings and recommendations from a multi-site evaluation of Independent Domestic Violence Advisors.* 2009.
109. Howarth, E. and A. Robinson, *Responding Effectively to Women Experiencing Severe Abuse: Identifying Key Components of a British Advocacy Intervention.* Violence against Women, 2016. **22**(1): p. 41-63.
110. Karatzias, T., et al., *Group psychotherapy for female adult survivors of interpersonal psychological trauma: a preliminary study in Scotland.* Journal of Mental Health, 2016. **25**(6): p. 512-519.
111. Kelley, T.M., et al., *The Efficacy of Principle-Based Correctional Counseling for Improving the Self-Control and Mental Health of People Incarcerated for Sexual Violence.* Violence Against Women, 2022. **28**(2): p. 573-592.
112. Kelly, L., *Domestic Violence Matters: an evaluation of a development project*. 1999.
113. Kelly, L. and N. Westmarland, *DOMESTIC VIOLENCE PERPETRATOR PROGRAMMES STEPS TOWARDS CHANGE: Project Mirabal Final Report*. 2015.
114. Koppensteiner, M.F., J. Matheson, and R. Plugor, *Project 360: An intervention to address victim-police engagement in repeat domestic violence cases*. 2017.
115. Lindsay, W.R., et al., *Comparing offenders against women and offenders against children on treatment outcome in offenders with intellectual disability.* Journal of Applied Research in Intellectual Disabilities, 2011. **24**(4): p. 361-369.
116. Lindsay, W.R., et al., *The treatment of six men with a learning disability convicted of sex offences with children.* British Journal of Clinical Psychology, 1998. **37**: p. 83-98.
117. Lowe, M., et al., *CORE assessment of adult survivors abused as children: A NAPAC group therapy evaluation.* Counselling & Psychotherapy Research, 2017. **17**(1): p. 71-79.
118. McConnell, N., et al., *Caring dads safer children*. 2014.
119. McConnell, N., M. Barnard, and J. Taylor, *Caring Dads Safer Children: Families' Perspectives on an Intervention for Maltreating Fathers.* Psychology of Violence, 2017. **7**(3): p. 406-416.
120. McCracken, K. and T. Deave, *Evaluation of the Caring Dads Cymru programme. Final report.* 2012.
121. Michie, A.M. and W.R. Lindsay, *A treatment component designed to enhance empathy in sex offenders with an intellectual disability.* The British Journal of Forensic Practice, 2012. **14**(1): p. 40-48.
122. Morgan, S. and J. Parkes, *The Hampshire Domestic Abuse Prevention Partnership 2016-2018: process and outcomes evaluation.* 2018.
123. Morgan, S.A., B.M.S. McCausland, and J. Parkes, *Baseline characteristics and outcomes of the main perpetrator programme within the Hampshire Domestic Abuse Prevention Partnership, UK: A mixed methods study.* Plos One, 2019. **14**(7).
124. Morrison, A. and L. Treliving, *Evaluation of outcome in a dynamically orientated group for adult males who have been sexually abused in childhood.* British Journal of Psychotherapy, 2002. **19**: p. 59-75.
125. Murphy, G., et al., *Cognitive-behavioural treatment for men with intellectual disabilities and sexually abusive behaviour: A pilot study.* Journal of Intellectual Disability Research, 2007. **51(11)**: p. 902-912.
126. Oasis. *Impact report 2013-2014*. 2014; Available from: https://issuu.com/kristinagwynne/docs/oasis_annual_report_2013-2014-web/1.
127. Oasis, *Impact Report 2015-2016.* 2016.
128. Ormston, R., C. Mullholland, and L. Setterfield, *Caledonian system evaluation: analysis of a programme for tackling domestic abuse in Scotland*. 2016, Edinburgh: Scotland. Scottish Government Social Research. vi, 88.
129. Payne, A., H. Liebling-Kalifani, and S. Joseph, *Client-centred group therapy for survivors of interpersonal trauma: A pilot investigation.* Counselling & Psychotherapy Research, 2007. **7**(2): p. 100-105.

For peer review only - http://bmjopen.bmj.com/site/about/guidelines.xhtml

130. Peckham, N.G., S. Howlett, and A. Corbett, *Evaluating a survivors group pilot for a women with significant intellectual disabilities who have been sexually abused.* Journal of Applied Research in Intellectual Disabilities, 2007. **20**(4): p. 308-322.
131. Robinson, A.L., *The Cardiff Women's Safety Unit: A multi-agency approach to domestic violence.* Final Evaluation Report. Cardiff University: School of Social Sciences.(Available online at http://www. cf. ac. uk/socsi/whoswho/robinson. html), 2003.
132. Robinson, A.L., *Domestic Violence MARACs (Multi-Agency Risk Assesment Conferences) for Very High-risk Victims in Cardiff, Wales: A Process and Outcome Evaluation.* 2004.
133. Robinson, A.L., *Reducing repeat victimization among high-risk victims of domestic violence: The benefits of a coordinated community response in Cardiff, Wales.* Violence Against Women, 2006. **12(8)**: p. 761-788.
134. Robjant, K., J. Roberts, and C. Katona, *Treating posttraumatic stress disorder in female victims of trafficking using narrative exposure therapy: A retrospective audit.* Frontiers in Psychiatry, 2017. **8**.
135. Rose, J., et al., *A sex offender treatment group for men with intellectual disabilities in a community setting.* The British Journal of Forensic Practice, 2012.
136. Ross, J., J. Sebire, and H. Strang, *Tracking Repeat Victimisation After Domestic Abuse Cases Are Heard With and Without Independent Domestic Violence Advisors (IDVAs) in an English Magistrate's Court.* Cambridge Journal of Evidence-Based Policing, 2022: p. 1-15.
137. Ryan, M., et al., *A prospective study of the effectiveness of group and individual psychotherapy for women CSA survivors.* Psychology and Psychotherapy: Theory, Research and Practice, 2005. **78(4)**: p. 465-480.
138. SafeLives, *Insights refuge England and Wales dataset 2015-18 Adult refuge services*. 2018.
139. SafeLives, *Defining Marac Outcomes National Pilot*. 2012.
140. SafeLives, *Insights England and Wales dataset 2018-19 Sexual violence services* 2019.
141. SafeLives, *Insights SV dataset 2020-21 Sexual violence (SV) services*. 2021.
142. SafeLives, *Insights health England and Wales dataset 2015-2018 Adults health-based services*. 2018.
143. SafeLives, *Insights helpline England and Wales dataset 2014-17 Adult helpline services*. 2017.
144. SafeLives, *Insights Idva national dataset 2012–13 Adult independent domestic violence advisor (Idva) services*. 2015.
145. SafeLives, *Insights Idva national dataset 2013–14 Adult independent domestic violence advisor (Idva) services*. 2015.
146. SafeLives, *Insights Idva England and Wales dataset 2016-17 Adult independent domestic violence advisor (Idva) services*. 2017.
147. SafeLives, *Insights Idva England and Wales dataset 2018-19 Adult Independent domestic violence advisor (Idva) services*. 2019.
148. SafeLives, *Insights Idva dataset 2020-21 Adult Independent domestic violence advisor (Idva) services*. 2021.
149. SafeLives, *Insights outreach national dataset 2012–13 Adult outreach services*. 2015.
150. SafeLives, *Insights outreach national dataset 2013–14 Adult outreach services.* 2015.
151. SafeLives, *Insights outreach England and Wales dataset 2016-17 Adult outreach services*. 2017.
152. SafeLives, *Insights outreach England and Wales dataset 2018-19 Adult outreach services*. 2019.
153. SafeLives, *Insights outreach dataset 2020-21 Adult outreach services*. 2021.
154. Sharpe, J., et al., *Group analytic therapy for male survivors of childhood sexual abuse.* Group Analysis, 2001. **34**: p. 195-209.
155. Skyner, D.R. and J. Waters, *Working with perpetrators of domestic violence to protect women and children: a partnership between Cheshire Probation Service and the NSPCC.* Child

For peer review only - http://bmjopen.bmj.com/site/about/guidelines.xhtml

Abuse Review: Journal of the British Association for the Study and Prevention of Child Abuse and Neglect, 1999. **8**(1): p. 46-54.
156. Smith, D., et al., *Adults with a history of child sexual abuse: Evaluation of a pilot therapy service.* British Medical Journal, 1995. **310(6988)**: p. 1175-1178.
157. Smith, E., et al., *Strengthening the mother-child relationship following domestic abuse: Service evaluation.* Child Abuse Review, 2015. **24**(4): p. 261-273.
158. Stanley, N., et al., *An evaluation of a new initiative for male perpetrators of domestic violence* 2011.
159. Stanley, N. and a. et, *Roadmap evaluation: final report - VOICES*. 2021, Bristol: Women's Aid SafeLives. 279.
160. Stanley, N. and a. et, *Roadmap evaluation: final report - SLCDPs*. 2021, Bristol: Women's Aid SafeLives. 279.
161. Strang, H., et al., *Reducing the harm of intimate partner violence: Randomized controlled trial of the Hampshire Constabulary CARA Experiment.* Cambridge Journal of Evidence-Based Policing, 2017. **1**(2): p. 160-173.
162. Taylor-Dunn, H. and R. Erol, *Improving the safety and well-being of domestic abuse survivors: The role of a specialist organisation in supporting the work of Independent Domestic Violence Advisors.* Crime Prevention and Community Safety, 2021. **23**(2): p. 115-136.
163. Eve; The Institute for Public Safety, C.J., *2019*. Evaluation of SHE.
164. Trevillion, K., et al., *Linking abuse and recovery through advocacy: An observational study.* Epidemiology and Psychiatric Sciences, 2014. **23**(1): p. 99-113.
165. Vallely, C., *Evaluation of domestic violence pilot sites at Caerphilly (Gwent) and Croydon 2004/05.* 2005.
166. Webster, R., *Kent and Medway Independent Domestic Violence Advisor Service Impact Evaluation FINAL REPORT*. 2015.
167. Westmarland, N. and S. Alderson, *The health, mental health, and well-being benefits of rape crisis counseling.* Journal of Interpersonal Violence, 2013. **28**(17): p. 3265-3282.
168. Williamson, D. and A. Boyle, *Results of the domestic violence advocacy program at addenbrookes hospital.* Academic Emergency Medicine, 2012. **19**: p. 741.
169. Williamson, E. and M. Hester, *Evaluation of the South Tyneside Domestic Abuse Perpetrator Programme (STDAPP) 2006-2008.* University of Bristol, 2009.
170. Miles, C. and K. Smith, *Nowhere to turn, 2018: findings from the second year of the No Woman Turned away project*. 2018, Bristol: Women's Aid. 48.
171. Women's Aid, *Nowhere To Turn, 2022: Findings from the sixth year of the No Woman Turned Away project*. 2022.
172. Women's Aid, *NOWHERE TO TURN Findings from the first year of the No Woman Turned Away project*. 2017.
173. Women's Aid, *NOWHERE TO TURN Findings from the third year of the No Woman Turned Away project*. 2019.
174. Women's Aid, *NOWHERE TO TURN Findings from the fourth year of the No Woman Turned Away project*. 2020, Women's Aid Federation of England.
175. Women's Aid, *NOWHERE TO TURN 2021 Findings from the fifth year of the No Woman Turned Away project*. 2021.
176. Women's Resource, C., *Women and Girls Network: the WGN empowerment model - redressing negative individual and social impacts of gendered violence through holistic counselling.* 2011.
177. Women's Resource, C., *Ashiana Network: journey towards safety.* 2011.
178. Campbell, J.C. and K.L. Soeken, *Women's responses to battering over time: an analysis of change.* Journal of Interpersonal violence, 1999. **14**(1): p. 21-40.
179. Rosenberg, M., *Rosenberg self-esteem scale.* Journal of Religion and Health, 1965.

For peer review only - http://bmjopen.bmj.com/site/about/guidelines.xhtml

180. Evans, C., et al., *Towards a standardised brief outcome measure: Psychometric properties and utility of the CORE–OM.* The British Journal of Psychiatry, 2002. **180**(1): p. 51-60.
181. Derogatis, L.R., *SCL-90-R: Administration, scoring & procedures manual-II for the (revised) version and other instruments of the psychopathology rating scale series.* Clinical Psychometric Research., 1992: p. 1-16.
182. Kroenke, K., R.L. Spitzer, and J.B. Williams, *The PHQ-9: validity of a brief depression severity measure.* Journal of general internal medicine, 2001. **16**(9): p. 606-613.
183. Spitzer, R.L., et al., *A brief measure for assessing generalized anxiety disorder: the GAD-7.* Archives of internal medicine, 2006. **166**(10): p. 1092-1097.
184. Sherman, L., P.W. Neyroud, and E. Neyroud, *The Cambridge crime harm index: Measuring total harm from crime based on sentencing guidelines.* Policing: a journal of policy and practice, 2016. **10**(3): p. 171-183.
185. Prins, A., et al., *The primary care PTSD screen for DSM-5 (PC-PTSD-5).[measurement instrument].* Universität Bielefeld: Bielefeld, Germany, 2015.
186. Women Against Violence Europe, *WAVE Report 2015: On the Role of Specialist Women's Support Services in Europe*. 2016.
187. Women's Aid. *What we believe: Our values*. 2022; Available from: https://www.womensaid.org.uk/what-we-believe/.
188. Robotham, D., A. Sweeney, and C. Perôt, *Violence, abuse and mental health network: Survivors' priority themes and questions for research—Consultation report, 2019.* Retrieved June, 2019. **26**: p. 2020.
189. Macy, R.J., et al., *Domestic violence and sexual assault services: Historical concerns and contemporary challenges.* Journal of Interpersonal Violence, 2010. **25**(1): p. 3-32.
190. SafeLives, *We only do bones here': Why London needs a whole-health approach to domestic abuse*. 2021, SafeLives: London.
191. Wiley, K., *Implementing domestic violence policy: When accountability trumps mission.* Affilia, 2020. **35**(4): p. 533-551.
192. Women's Aid, *Fragile funding landscape: The extent of local authority commissioning in the domestic abuse refuge sector in England 2020*. 2021, Women's Aid: Bristol.
193. Towers, J. and S. Walby, *Measuring the impact of cuts in public expenditure on the provision of services to prevent violence against women and girls.* 2012.
194. Corry, D., *Hearing women's voices: Why women 2018.* . 2018.
195. Heady, L., A. Kail, and C. Yeowart, *Understanding the stability and sustainability of the violence against women voluntary sector.* 2011.
196. Domestic Abuse Commissioner, *Early Findings from the Domestic Abuse Commissioner's Mapping of Domestic Abuse Services across England & Wales*. 2022.
197. Sullivan, R. and E. Sharples, *The CSA Centre's Evaluation Fund: A Reflection*. 2018, Barkingside: Centre of expertise on child sexual abuse.
198. Home Office, *Violence against women and girls services: commissioning toolkit*. 2022, Home Office: London.

For peer review only - http://bmjopen.bmj.com/site/about/guidelines.xhtml

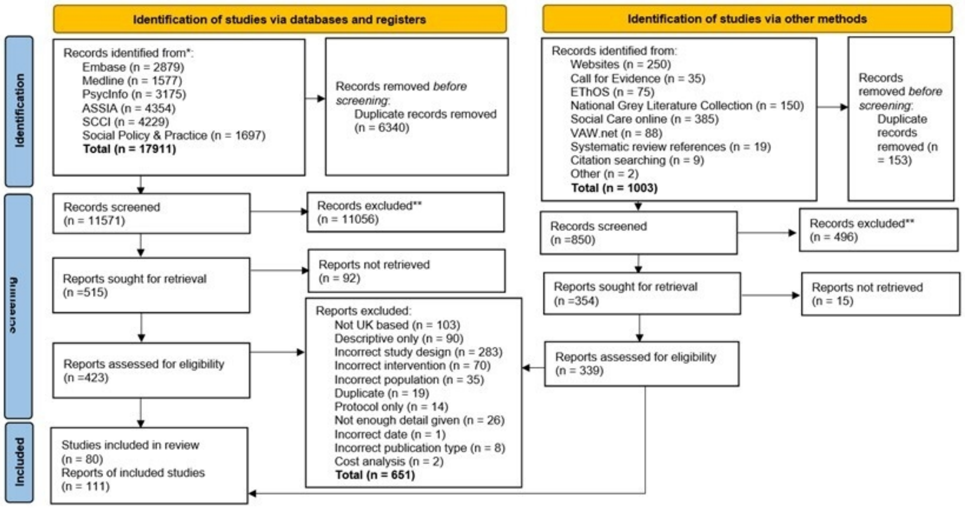

PRISMA flow diagram

35x18mm (600 x 600 DPI)

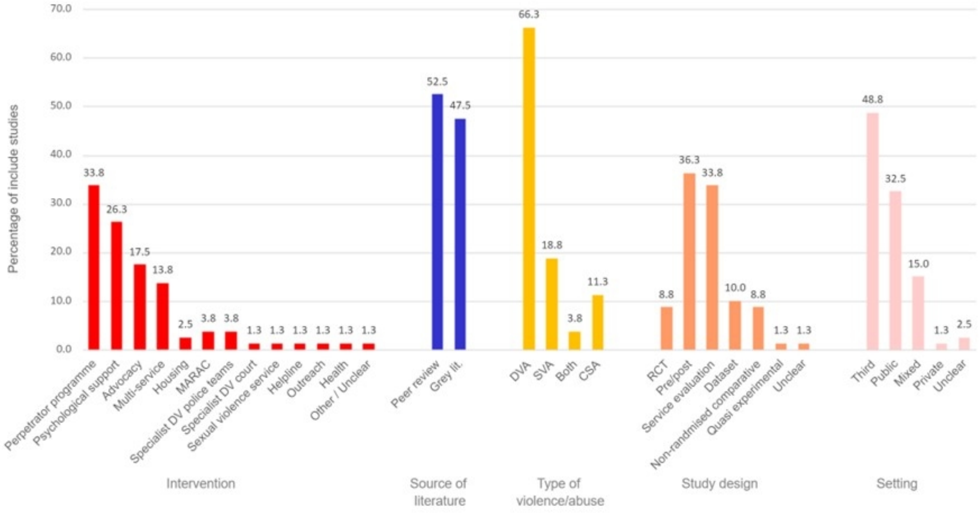

Bar chart to show the percentage of included studies with each categorisation type

75x41mm (300 x 300 DPI)

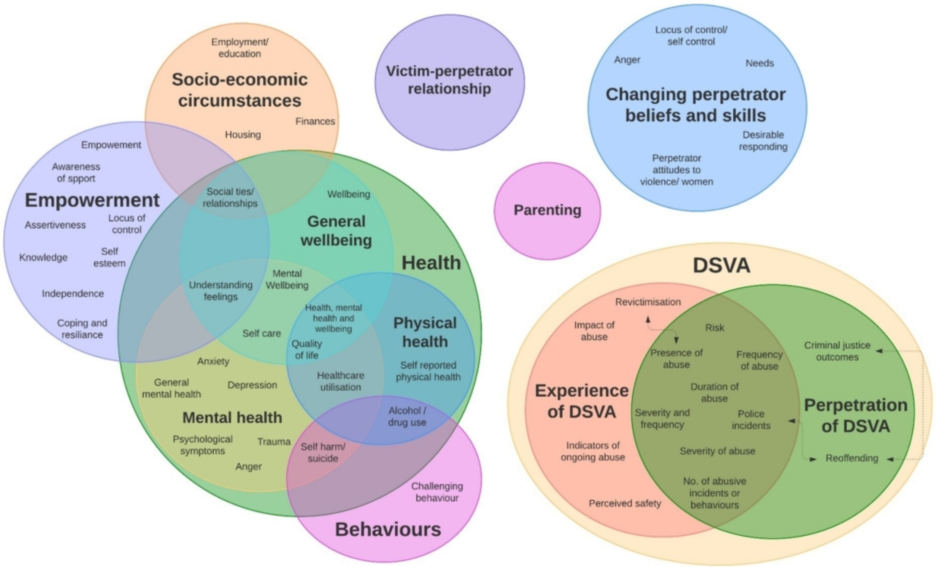

Venn diagram of outcome categories, domains and subdomains

167x99mm (300 x 300 DPI)

Appendix 1: Glossary

Advocacy: Adapted from the National Institute for Health and Clinical Excellence (NICE) Domestic Violence and Abuse: Multi-agency Working public health guideline (PH50)[1]. In general, advocacy for people who have experienced DSVA includes: legal, housing and financial advice: access to and use of community resources such as refuges, emergency housing and psychological interventions; and safety planning advice. The activities may differ according to the level of risk facing the person. Crisis advocacy involves working with the person for a limited period of time (they may then be referred on to more specialist agencies). Practitioners providing advocacy can also provide ongoing support and informal counselling. The intensity of the advocacy provided may vary. It may last for a year – or longer, if the person is particularly vulnerable.

By-and-for: By-and-for services are lived experience organisation, where all of the staff, senior managers, Board of Trustees, and women and girls using services are Black and minoritised women. Such services have a Black feminist ethos, locating themselves as anti-racist, social justice and intersectional organisations.

Domestic and sexual violence and abuse (DSVA): This review works to the UK government definition of domestic violence and abuse[2], and The Istanbul Convention[3], The World Health Organisation[4], and The Rome Statute of the International Criminal Court's (ICC) Elements of Crimes definitions of sexual violence and abuse[5]. In brief, domestic violence and abuse is defined as behaviour within an intimate relationship or family which includes acts of physical violence, sexual violence, emotional or psychological abuse, and controlling behaviours. This also includes 'honour'-based violence and FGM. Sexual violence and abuse is defined as any non-consensual act of sexual nature, extending to attempts to obtain a sexual act, unwanted sexual comments or advances, or acts to traffic, using force, threat of force or coercion.

Independent domestic violence advisers (IDVAs): Adapted from the NICE PH50 guideline. Also known as independent domestic violence advocates, IDVAs work primarily with people at high risk of domestic violence and abuse, independently of any one agency, to secure their safety and the safety of their children. Serving as the primary point of contact, IDVAs normally work with their clients from the point of crisis to assess the level of risk, discuss the options and develop plans that address their immediate safety, as well as longer-term solutions. In many areas they are funded by the local community safety partnership, in some areas they are funded by the police or local authorities.

Intervention: Adapted from the NICE Glossary. A treatment, service, project, or programme.

Intimate partner violence (IPV): Adapted from the World Health Organisation's definition. Intimate partner violence refers to behaviour within an intimate relationship that causes physical, sexual or psychological harm, including acts of physical aggression, sexual coercion, psychological abuse and controlling behaviours. This definition covers violence by both current and former spouses and partners.

Organisation: In the context of this review, organisations refer exclusively to those that carry out activities relating to DSVA. Organisations may include both specialist service providers, and organisations that do not directly provider services (such as women's rights organisations that may focus on activities such as campaigning, research, supporting specialist services, training, and education, for issues relating to DSVA, for example).

For peer review only

People who have experienced DSVA: Throughout this review, people who experience DSVA refers to those who are victims or survivors of DSVA.

Perpetrator programmes: Any group or individual intervention for people who have perpetrated DSVA, with the aim of reducing the incidence of violence by changing attitudes, behaviours and/or beliefs of perpetrators.

Primary, secondary, and tertiary prevention: Primary prevention refers to any action, strategy or policy that works to stop domestic violence from occurring in the first place. Primary prevention seeks to reduce the overall likelihood that anyone will become a victim or perpetrator of domestic violence by creating conditions that make violence less likely to occur. Secondary prevention refers to intervening and responding to violence that has already occurred in order to stop violence from happening again. Secondary prevention activities can include refuge, counselling, and safety planning. Tertiary prevention refers to ongoing support to victims and ongoing accountability for abusers. Tertiary prevention activities address the long-term consequences of domestic violence. They can include support groups and other resources for survivors to help them heal so that they do not experience abuse again. They can also include perpetrator programs for abusers to prevent them from continuing to perpetrate violence in the future.

Refuge or shelter: Adapted from the NICE PH50 guideline. Residential service – a safe house – provided for adults (usually women) and children who are experiencing domestic violence and abuse.

Service provider: Primary, community, including third sector, secondary and tertiary care providers of health and social care services, including prison health services and criminal justice agencies. In the context of DSVA, the service providers are predominantly third sector providers. Third sector service providers may be specialist DSVA providers (i.e., they only provide services relating to DSVA) or non-specialist-DSVA providers (i.e., they provide specialist support services for DSVA as well as support services for other issues). Outside of the third sector, service providers such as the NHS, police and courts may provide some DSVA specific services.

Third sector organisations: In this review the definition of third sector services provided by the National Audit Office has been adopted. 'Third sector organisations' is a term used to describe the range of organisations that are neither public sector nor private sector. It includes voluntary and community organisations (both registered charities and other organisations such as associations, self-help groups and community groups), social enterprises, mutuals and co-operatives. Third sector organisations generally: are independent of government. This is also an important part of the history and culture of the sector; are 'value-driven'. This means they are motivated by the desire to achieve social goals (for example, improving public welfare, the environment or economic well-being) rather than the desire to distribute profit; and reinvest any surpluses generated in the pursuit of their goals. For this reason they are sometimes called 'not-for-profit organisations'. A better term is 'not-for-personal-profit'. In many cases, third sector organisations need to make surpluses (or 'profits') to be financially sustainable.

For peer review only

References

1. National Institute for Health and Care Excellence. *Domestic violence and abuse: multi-agency working: Public health guideline [PH50]. Recommendations for research*. 2014; Available from: https://www.nice.org.uk/guidance/ph50/chapter/5-Recommendations-for-research.
2. Home Office. *Circular 003/2013: new government domestic violence and abuse definition*. 2013; Available from: https://www.gov.uk/government/publications/new-government-domestic-violence-and-abuse-definition/circular-0032013-new-government-domestic-violence-and-abuse-definition.
3. Council of Europe, *Council of Europe Convention on preventing and combating violence against women and domestic violence (CETS No. 210)*. 2011.
4. World Health Organization, *World report on violence and health*. 2002.
5. International Criminal Court, *Elements of Crime*. 2013.

For peer review only

Appendix 2: Preferred Reporting Items for Systematic reviews and Meta-Analyses extension for Scoping Reviews (PRISMA-ScR) Checklist

SECTION	ITEM	PRISMA-ScR CHECKLIST ITEM	REPORTED ON PAGE #
**TITLE**			
Title	1	Identify the report as a scoping review.	1
**ABSTRACT**			
Structured summary	2	Provide a structured summary that includes (as applicable): background, objectives, eligibility criteria, sources of evidence, charting methods, results, and conclusions that relate to the review questions and objectives.	3-4
**INTRODUCTION**			
Rationale	3	Describe the rationale for the review in the context of what is already known. Explain why the review questions/objectives lend themselves to a scoping review approach.	5-6
Objectives	4	Provide an explicit statement of the questions and objectives being addressed with reference to their key elements (e.g., population or participants, concepts, and context) or other relevant key elements used to conceptualize the review questions and/or objectives.	6-7
**METHODS**			
Protocol and registration	5	Indicate whether a review protocol exists; state if and where it can be accessed (e.g., a Web address); and if available, provide registration information, including the registration number.	7
Eligibility criteria	6	Specify characteristics of the sources of evidence used as eligibility criteria (e.g., years considered, language, and publication status), and provide a rationale.	7-8 and Table 1
Information sources*	7	Describe all information sources in the search (e.g., databases with dates of coverage and contact with authors to identify additional sources), as well as the date the most recent search was executed.	9
Search	8	Present the full electronic search strategy for at least 1 database, including any limits used, such that it could be repeated.	Appendix 2
Selection of sources of evidence†	9	State the process for selecting sources of evidence (i.e., screening and eligibility) included in the scoping review.	10
Data charting process‡	10	Describe the methods of charting data from the included sources of evidence (e.g., calibrated forms or forms that have been tested by the team before their use, and whether data charting was done independently or in duplicate) and any processes for obtaining and confirming data from investigators.	10
Data items	11	List and define all variables for which data were sought and any assumptions and simplifications made.	10
Critical appraisal of individual sources of evidence§	12	If done, provide a rationale for conducting a critical appraisal of included sources of evidence; describe the methods used and how this information was used in any data synthesis (if appropriate).	N/A

For peer review only

SECTION	ITEM	PRISMA-ScR CHECKLIST ITEM	REPORTED ON PAGE #
Synthesis of results	13	Describe the methods of handling and summarizing the data that were charted.	10-11
**RESULTS**			
Selection of sources of evidence	14	Give numbers of sources of evidence screened, assessed for eligibility, and included in the review, with reasons for exclusions at each stage, ideally using a flow diagram.	12 and Figure 1
Characteristics of sources of evidence	15	For each source of evidence, present characteristics for which data were charted and provide the citations.	12-16
Critical appraisal within sources of evidence	16	If done, present data on critical appraisal of included sources of evidence (see item 12).	N/A
Results of individual sources of evidence	17	For each included source of evidence, present the relevant data that were charted that relate to the review questions and objectives.	Appendix 4
Synthesis of results	18	Summarize and/or present the charting results as they relate to the review questions and objectives.	17-21
**DISCUSSION**			
Summary of evidence	19	Summarize the main results (including an overview of concepts, themes, and types of evidence available), link to the review questions and objectives, and consider the relevance to key groups.	22-23
Limitations	20	Discuss the limitations of the scoping review process.	24-25
Conclusions	21	Provide a general interpretation of the results with respect to the review questions and objectives, as well as potential implications and/or next steps.	26
**FUNDING**			
Funding	22	Describe sources of funding for the included sources of evidence, as well as sources of funding for the scoping review. Describe the role of the funders of the scoping review.	3-4

JBI = Joanna Briggs Institute; PRISMA-ScR = Preferred Reporting Items for Systematic reviews and Meta-Analyses extension for Scoping Reviews.
* Where *sources of evidence* (see second footnote) are compiled from, such as bibliographic databases, social media platforms, and Web sites.
† A more inclusive/heterogeneous term used to account for the different types of evidence or data sources (e.g., quantitative and/or qualitative research, expert opinion, and policy documents) that may be eligible in a scoping review as opposed to only studies. This is not to be confused with *information sources* (see first footnote).
‡ The frameworks by Arksey and O'Malley (6) and Levac and colleagues (7) and the JBI guidance (4, 5) refer to the process of data extraction in a scoping review as data charting.
§ The process of systematically examining research evidence to assess its validity, results, and relevance before using it to inform a decision. This term is used for items 12 and 19 instead of "risk of bias" (which is more applicable to systematic reviews of interventions) to include and acknowledge the various sources of evidence that may be used in a scoping review (e.g., quantitative and/or qualitative research, expert opinion, and policy document).

From: Tricco AC, Lillie E, Zarin W, O'Brien KK, Colquhoun H, Levac D, et al. PRISMA Extension for Scoping Reviews (PRISMAScR): Checklist and Explanation. Ann Intern Med. 2018;169:467–473. doi: 10.7326/M18-0850.

Appendix 3: Example search strategies

Search strategy development process

Search terms were identified during initial scoping and protocol development stages. An initial list of search terms was drafted based on terms found in the literature and based on search terms used in systematic reviews in related areas. This initial list of terms was shared with the review team and with the Patient and Public Involvement advisory group, who added any additional terms thought to be relevant to the review's research question.

Peer review example: Medline

1. exp United kingdom/
2. (national health service* or nhs*).ti,ab,in.
3. (english not ((published or publication* or translat* or written or language* or speak* or literature or citation*) adj5 english)).ti,ab.
4. (gb or "g.b." or britain* or (british* not "british columbia") or uk or "u.k." or united kingdom* or (england* not "new england") or northern ireland* or northern irish* or scotland* or scottish* or ((wales or "south wales") not "new south wales") or welsh*).ti,ab,jw,in.
5. (bangor or "bangor's" or cardiff or "cardiff's" or newport or "newport's" or st asaph or "st asaph's" or st davids or swansea or "swansea's").ti,ab,in,cq.
6. (bath or "bath's" or ((birmingham not alabama*) or ("birmingham's" not alabama*) or bradford or "bradford's" or brighton or "brighton's" or bristol or "bristol's" or carlisle* or "carlisle's" or (cambridge not (massachusetts* or boston* or harvard*)) or ("cambridge's" not (massachusetts* or boston* or harvard*)) or (canterbury not zealand*) or ("canterbury's" not zealand*) or chelmsford or "chelmsford's" or chester or "chester's" or chichester or "chichester's" or coventry or "coventry's" or derby or "derby's" or (durham not (carolina* or nc)) or ("durham's" not (carolina* or nc)) or ely or "ely's" or exeter or "exeter's" or gloucester or "gloucester's" or hereford or "hereford's" or hull or "hull's" or lancaster or "lancaster's" or leeds* or leicester or "leicester's" or (lincoln not nebraska*) or ("lincoln's" not nebraska*) or (liverpool not (new south wales* or nsw)) or ("liverpool's" not (new south wales* or nsw)) or ((london not (ontario* or ont or toronto*)) or ("london's" not (ontario* or ont or toronto*)) or manchester or "manchester's" or (newcastle not (new south wales* or nsw)) or ("newcastle's" not (new south wales* or nsw)) or norwich or "norwich's" or nottingham or "nottingham's" or oxford or "oxford's" or peterborough or "peterborough's" or plymouth or "plymouth's" or portsmouth or "portsmouth's" or preston or "preston's" or ripon or "ripon's" or salford or "salford's" or salisbury or "salisbury's" or sheffield or "sheffield's" or southampton or "southampton's" or st albans or stoke or "stoke's" or sunderland or "sunderland's" or truro or "truro's" or wakefield or "wakefield's" or wells or westminster or "westminster's" or winchester or "winchester's" or wolverhampton or "wolverhampton's" or (worcester not (massachusetts* or boston* or harvard*)) or ("worcester's" not (massachusetts* or boston* or harvard*)) or (york not ("new york*" or ny or ontario* or ont or toronto*)) or ("york's" not ("new york*" or ny or ontario* or ont or toronto*))))).ti,ab,in.
7. (aberdeen or "aberdeen's" or dundee or "dundee's" or edinburgh or "edinburgh's" or glasgow or "glasgow's" or inverness or (perth not australia*) or ("perth's" not australia*) or stirling or "stirling's").ti,ab,in.
8. (armagh or "armagh's" or belfast or "belfast's" or lisburn or "lisburn's" or londonderry or "londonderry's" or derry or "derry's" or newry or "newry's").ti,ab,in.
9. (exp africa/ or exp americas/ or exp antarctic regions/ or exp arctic regions/ or exp asia/ or exp australia/ or exp oceania/) not (exp United Kingdom/ or europe/)

10. (child adj3 maltreat*).ti,ab,kw.
11. (sexual adj3 harass*).ti,ab,kw.
12. (batter* adj3 (wom#n or wife or wives)).ti,ab,kw.
13. rape*.ti,ab,kw.
14. (beat* adj3 (partner* or spous* or wom#n or wife or wives or m#n or husband*)).ti,ab,kw.
15. (sexual adj3 coercion).ti,ab,kw.
16. female genital mutilation.ti,ab,kw.
17. forced marriage.ti,ab,kw.
18. sex traffic*.ti,ab,kw.
19. revenge porn.ti,ab,kw.
20. controlling behaviour.ti,ab,kw.
21. prostitut*.ti,ab,kw.
22. sex work*.ti,ab,kw.
23. stalking.ti,ab,kw.
24. (drink adj3 spik*).ti,ab,kw.

25. domestic abuse service*.ti,ab,kw.
26. women's project*.ti,ab,kw.
27. women's resource centre.ti,ab,kw.
28. safety unit*.ti,ab,kw.
29. domestic abuse support service*.ti,ab,kw.
30. domestic abuse team*.ti,ab,kw.
31. domestic violence intervention*.ti,ab,kw.
32. women's service*.ti,ab,kw.
33. women's rights organi#ation*.ti,ab,kw.
34. domestic abuse advice and support.ti,ab,kw.
35. women's centre*.ti,ab,kw.
36. domestic abuse project*.ti,ab,kw.
37. domestic and sexual abuse project*.ti,ab,kw.
38. victim care service*.ti,ab,kw.
39. support centre*.ti,ab,kw.
40. women's welfare.ti,ab,kw.
41. speciali* service*.ti,ab,kw.
42. speciali* sexual violence service*.ti,ab,kw.
43. speciali* domestic violence service*.ti,ab,kw.
44. speciali* support.ti,ab,kw.
45. speciali* violence and abuse service*.ti,ab,kw.
46. speciali* women's voluntary sector service*.ti,ab,kw.
47. service* for survivors.ti,ab,kw.
48. victim service*.ti,ab,kw.
49. victim support*.ti,ab,kw.
50. violence against women and girls service*.ti,ab,kw.
51. domestic violence support service*.ti,ab,kw.
52. community based service*.ti,ab,kw.
53. sexual assault referral centre*.ti,ab,kw.
54. rape crisis.ti,ab,kw.
55. third sector.ti,ab,kw.
56. second tier.ti,ab,kw.
57. crisis intervention.ti,ab,kw.
58. independent domestic violence advoca*.ti,ab,kw.
59. IDVA.ti,ab,kw.
60. independent sexual violence advoca*.ti,ab,kw.
61. ISVA.ti,ab,kw.
62. shelter*.ti,ab,kw.
63. refuge.ti,ab,kw.

For peer review only

64. refuges.ti,ab,kw.
65. advoca*.ti,ab,kw.
66. counsel?ing.ti,ab,kw.
67. outreach.ti,ab,kw.
68. helpline*.ti,ab,kw.
69. emergency shelter*.ti,ab,kw.
70. supportive hous*.ti,ab,kw.
71. violence against women service*.ti,ab,kw.
72. recovery service*.ti,ab,kw.
73. speciali* victim service*.ti,ab,kw.
74. independent domestic violence advisor*.ti,ab,kw.
75. independent sexual violence advisor*.ti,ab,kw.
76. resettlement.ti,ab,kw.
77. floating support.ti,ab,kw.
78. drop-in.ti,ab,kw.
79. online chat.ti,ab,kw.
80. support group*.ti,ab,kw.
81. violence advocacy project*.ti,ab,kw.
82. group work program*.ti,ab,kw.
83. information and advice.ti,ab,kw.
84. (dedicated adj4 service*).ti,ab,kw.
85. charit*.ti,ab,kw.
86. independent.ti,ab,kw.
87. voluntary.ti,ab,kw.
88. public sector.ti,ab,kw.
89. specialist.ti,ab,kw.
90. speciali?ed.ti,ab,kw.
91. non-governmental organi#ation*.ti,ab,kw.
92. nonprofit organi#ation*.ti,ab,kw.
93. not-for-profit organi#ation*.ti,ab,kw.
94. by and for service*.ti,ab,kw.
95. by and for organi#ation*.ti,ab,kw.
96. therapy.ti,ab,kw.
97. empowerment.ti,ab,kw.
98. case management.ti,ab,kw.
99. stress management.ti,ab,kw.
100. safety behaviour*.ti,ab,kw.
101. safety strateg*.ti,ab,kw.
102. legal advice.ti,ab,kw.
103. mindfulness based stress reduction.ti,ab,kw.
104. home visit*.ti,ab,kw.
105. sanctuar*.ti,ab,kw.
106. safe house*.ti,ab,kw.
107. flexible fund*.ti,ab,kw.
108. exp battered woman/
109. exp domestic violence/ or exp child abuse/ or exp spouse abuse/ or exp gender-based violence/ or exp intimate partner violence/ or exp physical abuse/ or exp rape/
110. exp partner violence/
111. exp family violence/
112. exp coercion/
113. exp emotional abuse/
114. exp sexual violence/ or exp sexual abuse/ or exp sexual assault/
115. ("City of London" or Bedfordshire or Berkshire or Bristol or Buckinghamshire or Cambridgeshire or Cheshire or Cornwall or Cumbria or Derbyshire or Devon or Dorset or Durham or Yorkshire or Essex or Gloucestershire or "greater london" or "greater manchester" or "isle of wight" or "west midlands" or sussex or Hampshire or

For peer review only

Herefordshire or Hertfordshire or Kent or Lancashire Leicestershire or Lincolnshire or Merseyside or Norfolk or Northamptonshire or Northumberland or Nottinghamshire or Oxfordshire or Rutland or Shropshire or Somerset or Staffordshire or Suffolk or Surrey or "Tyne and Wear" or Warwickshire or Wiltshire or Worcestershire or Aberdeenshire or Angus or Forfarshire or Argyll* or Ayrshire or "County of Ayr" or "County of Banff" or "County of Berwick" or "County of Bute" or "County of Clackmannan" or "East Lothian" or "Ross and Cromarty" or "West Lothian" or Banffshire or Berwickshire or Buteshire or Caithness or Clackmannanshire or Dumfriesshire or Dunbartonshire or Fife or Invernessshire or Kincardineshire or Kinrossshire or Kirkcudbrightshire or Lanarkshire or Midlothian or Moray or Nairnshire or Orkney or Peebleshire or Perthshire or Renfrewshire or Roxburghshire or Selkirkshire or Shetland or Stirlingshire or Sutherland or Wigtownshire or "Blaenau Gwent" or "Isle of Anglesey" or "Merthyr Tydfil" or "Neath Port Talbot" or "Rhondda Cynon Taff" or "Vale of Glamorgan" or Bridgend or Caerphilly or Cardiff or Carmarthenshire or Ceredigion or Conwy or Denbighshire or Flintshire or Gwynedd or Wrexham or Antrim or Armagh or Down or Fermanagh or Londonderry or derry or Swansea or Torfaen or Newport or Pembrokeshire or Powys or Monmouthshire or Tyrone).ti,ab,in.

116. 1 or 2 or 3 or 4 or 5 or 6 or 7 or 8 or 115
117. 116 not 9
118. tenant support.ti,ab,kw.
119. perpetrator programme*.ti,ab,kw.
120. (violen* adj3 (sexual or domestic or child or partner* or spous* or couple* or family or interpersonal or date or dating or gender based or coercive or conjugal or victim* or survivor* or perpetrat* or technology-facilitated or wom#n or image based or honor based or obstetric)).ti,ab,kw.
121. (abus* adj3 (sexual or domestic or child or partner* or spous* or family or couple* or gender based or wom#n or wife or wives or victim* or survivor* or perpetrat* or financ* or economic or image-based or technology-facilitated)).ti,ab,kw.
122. (assault* adj3 (sexual or partner* or spous* or victim* or survivor* or perpetrat* or couple*)).ti,ab,kw.
123. 25 or 26 or 27 or 28 or 29 or 30 or 31 or 32 or 33 or 34 or 35 or 36 or 37 or 38 or 39 or 40 or 41 or 42 or 43 or 44 or 45 or 46 or 47 or 48 or 49 or 50 or 51 or 52 or 53 or 54 or 55 or 56 or 57 or 58 or 59 or 60 or 61 or 62 or 63 or 64 or 65 or 66 or 67 or 68 or 69 or 70 or 71 or 72 or 73 or 74 or 75 or 76 or 77 or 78 or 79 or 80 or 81 or 82 or 83 or 84 or 85 or 86 or 87 or 88 or 89 or 90 or 91 or 92 or 93 or 94 or 95 or 96 or 97 or 98 or 99 or 100 or 101 or 102 or 103 or 104 or 105 or 106 or 107 or 118 or 119
124. 10 or 11 or 12 or 13 or 14 or 15 or 16 or 17 or 18 or 19 or 20 or 21 or 22 or 23 or 24 or 108 or 109 or 110 or 111 or 112 or 113 or 114 or 120 or 121 or 122
125. 117 and 123 and 124
126. limit 125 to (english language and humans)

For peer review only

Grey literature example: Social care online

(combined v4:
UK terms [
- Location:"'england"' including broader terms
- OR Location:"'wales"' including this term only
- OR Location:"'scotland"' including this term only
- OR Location:"'northern ireland"' including this term only
- OR Location:"'united kingdom"' including this term only
]
AND
service terms - all fields [
- AllFields:"'specialised service"'
- OR AllFields:"'specialist service"'
- OR AllFields:"'support service"'
- OR AllFields:"'abuse service"'
- OR AllFields:"'violence service"'
]
AND
violence terms v2 [
- SubjectTerms:"'domestic violence"' including related terms
- OR SubjectTerms:"'sexual abuse"' including related terms
- OR AllFields:"'sexual violence"'
- OR AllFields:"'domestic abuse"'
- OR AllFields:"'domestic violence"'
- OR AllFields:"'sexual abuse"'
]
)

For peer review only

Appendix 4: Details of included studies

Study	Peer review or grey lit	Study design	Setting/ sector	Type of intervention	Type of abuse*	No. of outcomes
ADVA (2009)[1]	Grey lit.	Service evaluation	Third	*"Repair"*<br>Perpetrator programme<br>• Comprising individual and group work sessions. Primarily cognitive behavioural, focusing on motivation, responsibility, safety and acknowledgment<br>Women's service<br>• Supportive befriending, information about legal and support services, expectation management, group work, telephone contact, feedback on partners' attendance, safety planning and monitoring and awareness raising	DVA	7
Advance (2020)[2]	Grey lit.	Service evaluation	Third	*"Minerva"*<br>Multi-service<br>• Advocacy for access to other community services<br>• One-to-one and group work support on a range of issues including emotional wellbeing, physical and mental health, violence and abuse, problematic substance use<br>• Benefits and debt advice, housing	DVA	1
Barber et al (2000)[3]	Peer review	Pre-post	Public: Learning disability hospital	-<br>Psychological support<br>• Group therapy using a person-centred therapeutic model, which aimed to run interactive and structured educative sessions, supplemented with supportive and non-confrontational discussions	SVA	4

For peer review only

Study	Peer review or grey lit	Study design	Setting/ sector	Type of intervention	Type of abuse*	No. of outcomes
Bloomfield & Dixon (2015)[4]	Grey lit.	Non-randomised comparative	Public: National Probation Service	*"Integrated Domestic Abuse Programme (IDAP) and Community Domestic Violence Programme (CDVP)"*<br>Perpetrator programme<br>• Cognitive behavioural programme, which challenged offenders' attitudes and beliefs to change their behaviour	DVA	2
Bowen (2010)[5-9]	Peer review	Quasi experimental	Public: local probation service	*"Domestic Violence Perpetrator Programme"*<br>Perpetrator programme<br>• Psychoeducational treatment programme in a group setting, focusing on definitions of DV, socialisation, impact of DV on partners and children, empathy, sexual respect and accountability and communication	DVA	9
Brady et al (2021)[10]	Peer review	RCT	Mixed: Third (human rights charity); unclear ('clinical setting')	-<br>Psychological support<br>• Narrative exposure therapy, involving co-construction of traumatic experiences and a therapy plan, to process traumatic memories. Psychoeducation was also delivered to all participants	SVA	5
Burton et al (1998)[11]	Grey lit.	Service evaluation	Third	*"Violence Prevention Programme (VPP)"*<br>Perpetrator programme<br>• Structured group sessions designed to assist men in understanding why they abuse, how they can change it, and constructing respectful relationships with women<br>Multi-service<br>• Telephone advice and support; making contact with, and regular follow-up of, partners of men on VPP; one-to-one support sessions; two weekly support groups;	DVA	2

For peer review only

Study	Peer review or grey lit	Study design	Setting/ sector	Type of intervention	Type of abuse*	No. of outcomes
				advocacy for women users; weekly liaison with the VPP; interagency work, including workshops and training; administration; and training for new volunteers. These also involve safety planning, housing / financial / legal advice and education (understanding domestic violence)		
Callaghan et al (2020)[12, 13]	Grey lit.	Pre-post	Third: DV charities / organisations	*"Make a Change"*<br>Perpetrator programme<br>• Group-based intervention for perpetrators<br>Psychological support<br>• Individual support for (ex)partners	DVA	13
Calvert et al (2015)[14]	Peer review	Pre-post	Public: Tertiary psychotherapy service	-<br>Psychological support<br>• Group cognitive analytic therapy	SVA (CSA)	6
Clarke & Llewelyn (1994)[15]	Peer review	Pre-post	Public: NHS	-<br>Psychological support<br>• Cognitive analytic therapy	SVA (CSA)	4
Clarke & Pearson (2000)[16]	Peer review	Pre-post	Public: NHS	-<br>Psychological support<br>• Cognitive analytic therapy	SVA (CSA)	4
Cook et al (1991)[17]	Peer review	Pre-post	Public	-<br>Perpetrator programme<br>• Group treatment for sex offenders	SVA	1
Co-ordinated Action Against Domestic	Grey lit.	Data-set / service evaluation	Third	-<br>Advocacy<br>• 14 IDVA services, with types of support	DVA	11

For peer review only

Study	Peer review or grey lit	Study design	Setting/ sector	Type of intervention	Type of abuse*	No. of outcomes
Abuse (2012)[18, 19]				including safety planning, support with MARAC, liaison and support with police, support with criminal court cases, liaison and support with probation, support with civil orders, support with housing, financial benefits advice and support, support with immigration, health and wellbeing advice and support, and support with children		
Coy and Kelly (2011)[20]	Grey lit.	Service evaluation	Third	- Advocacy<br>• Four IDVAs in different settings (police station, hospital A&E department, a community based DV project, and a women only VAW organisation)	DVA	2
Dheensa & Penny (2019)[21]	Grey lit.	Service evaluation	Third	- Advocacy<br>• Learning disability ISVA	SVA	1
Dobash et al (1999)[22]	Peer review	Non-randomised comparative	Public	*"IDVPP"* and *"CHANGE"* Perpetrator programme<br>• Two perpetrator programmes, both focused on challenging group work	DVA	3
Ellis (2012)[23]	Peer review	Pre-post	Third?	*"The Butterfly Programme"* Psychosocial support<br>• Group therapy	SVA (CSA)	6
Farrell & Buckley (1999)[24]	Peer review	Pre-post	Public	*"The Domestic Violence Unit"* Specialist DV police teams<br>• Police officers dedicated to dealing with DV cases with specialist training.	DVA	5
Ferrari et al (2018)[25]	Peer review	Randomised controlled trial	Third	*"PATH"* Advocacy<br>• Specialist psychological advocates providing	DVA	6

For peer review only

Study	Peer review or grey lit	Study design	Setting/ sector	Type of intervention	Type of abuse*	No. of outcomes
				1:1 support on top of regular advocacy provision		
Friendship et al. (2003)[26]	Peer review	Pre-post with retrospectively selected comparison group	Public: Prisons	*"SOTP"*<br>Perpetrator programme<br>• Two programmes based on the cognitive behavioural model, using group sessions	DVA	3
Garry & Munro (2020)[27-29]	Grey lit.	Service evaluation	Third	*"The Rape and Sexual Violence Project (RSVP) adult counselling service"*<br>Psychological support<br>• Face-to-face counselling. Clients can also access RSVPs other support services	SVA	1
Geoghegan-Fittall et al (2020)[30]	Grey lit.	Service evaluation	Third	*"The Croydon Drive Project"*<br>Perpetrator programme<br>• Direct and indirect interventions and activity disruption. Interventions may be singular or concurrent	DVA	3
Gilchrist et al (2021)[31, 32]	Peer review	Randomised controlled trial	Mixed: NHS and voluntary sector	*"ADVANCE"*<br>Perpetrator programme<br>• Manualised evidence-informed tailored intervention focusing on developing healthy relationships	DVA	14
Goosey et al (2017)[33]	Peer review	Randomised controlled trial	Mixed: community safety team, police, drug and alcohol service, Thames Valley Probation, Youth Offending	-<br>Multi-service<br>• Integrated cased management using the Domestic Abuse Service Coordination model, including support for victims, counselling for perpetrators, and follow up	DVA	1

For peer review only

Study	Peer review or grey lit	Study design	Setting/ sector	Type of intervention	Type of abuse*	No. of outcomes
			Service, Domestic Abuse Perpetrator Service, Berkshire Women's Aid, Bracknell Forest Homes	police visits		
Halliwell et al (2019)[34]	Peer review	Non-randomised comparative	Mixed: Public and Third	-<br>Advocacy<br>• A hospital based IDVA who provided support and advice, risk assessment and safety planning, referral, partnership work and training hospital staff	DVA	8
Hester et al (2019)[35-38]	Grey lit	Service evaluation using RCT design	Third	*"Drive"*<br>Perpetrator programme<br>• Intensive case management and multi-agency response. Uses disruption, support and behaviour change interventions alongside protective work<br>Advocacy<br>• One-to-one IDVA support for the associated victims-survivors	DVA	10
Hester & Westmarland (2005)[39]	Grey lit.	Service evaluation	Mixed: Third, public	-<br>Multiple / multi-service<br>• 27 domestic violence projects split in to seven packages (criminal and civil justice, protection and prevention, Black and other ethnic minorities, health, multi-service, education and rural work). A sub selection of the 27 that report relevant outcomes and use the included methodology were extracted, including: Bradford Staying Put, Hammersmith and Fulham Standing Together, Northampton Sunflower Centre,	DVA	17

Study	Peer review or grey lit	Study design	Setting/ sector	Type of intervention	Type of abuse*	No. of outcomes
				Cheshire and Taunton – use of photographic evidence, Croydon Domestic Violence Advocate Service, Northampton Sunflower Centre		
Howarth et al (2009)[98, 99]	Grey lit	Service evaluation	Third	-<br>Advocacy<br>• Seven IDVA services in a range of rurality's, sizes and age of service	DVA	13
Howarth and Robinson (2016)[100]	Peer review	Pre-post	Unclear	-<br>Advocacy<br>• Seven IDVA services. Support provided included safety planning, housing and accommodation, child-related support, access to justice systems, health and wellbeing, and welfare and immigration support	DVA	4
Karatzias et al (2016)[101]	Peer review	Pre-post	Public: NHS	*"Trauma Recovery and Empowerment Model"*<br>Psychological support<br>• Group psychotherapy using cognitive behavioural and skills training techniques	DVA<br>SVA<br>(CSA)	5
Kelley et al (2022)[40]	Peer review	Non-randomised comparative	Private: Privately owned prison	*"Principle-based correctional counselling"*<br>Perpetrator programme<br>• Classes including modules on building rapport, exploration of reality, exploration of thought, exploration of feelings/behaviour.<br>• All participants also participated in the required CBT based Sexual offender Treatment programme	SVA	6
Kelly et al	Grey lit.	Service	Public	*"Domestic Violence Matters"*	DVA	5

For peer review only

Study	Peer review or grey lit	Study design	Setting/ sector	Type of intervention	Type of abuse*	No. of outcomes
(1999)[41]		evaluation		Specialist domestic violence police teams<br>• Five support workers who worked alongside the police to support women where an arrest of a perpetrator occurred.		
Kelly & Westmarland (2015)[42]	Grey lit.	Service evaluation	Third	*"DVPP"*<br>Perpetrator programme<br>• Twelve Respect accredited DVPPs. All have manuals and involved group work.<br>Unclear – Women's support service<br>• Each perpetrator programme had an integrated women's support service, however no further details are provided.	DVA	7
Koppensteiner et al (2019)[43]	Grey lit.	Randomised controlled trial	Public: Police	*"Project 360"*<br>Specialist domestic violence police teams<br>• Engagement workers who contact victims and offer further assistance, including information on legal options, referrals to support services, and safety planning	DVA	1
Lindsay et al (2011)[44]	Peer review	Pre-post	Unclear	-<br>Perpetrator programme<br>• A sex offender treatment programme, including modules on disclosure, pathways into offending, cognitive distortions, childhood abuse, victim awareness, relapse prevention, and adaptive attachment	SVA	2
Lindsay et al. (1998)[45]	Peer review	Pre-post	Public: Psychology / psychiatry department	-<br>Perpetrator programme<br>• Group sessions focused on sex offending and cognitive change, including taking responsibility, victim awareness and behaviours consistent with offending	SVA	1

Study	Peer review or grey lit	Study design	Setting/ sector	Type of intervention	Type of abuse*	No. of outcomes
Lowe et al (2017)[46]	Peer review	Pre-post	Third	-<br>Psychological support<br>• Group therapy. Sessions used a person centred, psychoeducational approach with elements of cognitive behavioural therapy	SVA (CSA)	3
McConnell et al (2017)[47, 48]	Peer review	Pre-post	Third	*"Caring Dads Safer Children"*<br>Perpetrator programme<br>• Includes seven treatment targets: anger/hostility; family cohesion/ coparenting/domestic violence; perceptions of the child as a problem; use of corporal punishment and other aversive behaviours; positive and involved parent-child relationship; self-centeredness; and misuse of substances	DVA	4
McCracken & Deave (2012)[49]	Grey lit.	Service evaluation	Third	*"Caring Dads Cymru"*<br>Perpetrator programme<br>• Group work programme based on cognitive behavioural therapy and motivational models of intervention	DVA	4
Michie & Lindsay (2012)[50]	Peer review	Non-randomised comparative	Public: NHS	-<br>Perpetrator programme<br>• Cognitive behavioural therapy with an empathy component	SVA	1
Morgan et al (2019)[51, 52]	Peer review	Pre-post	Third	*"RADAR/ADAPT"*<br>Perpetrator programme<br>• A multiagency initiative that provides a tiered needs-led approach. It provides a programme for perpetrators and support for victims	DVA	4
Morrison &	Peer review	Pre-post	Public: Local	-	SVA	1

For peer review only

Study	Peer review or grey lit	Study design	Setting/ sector	Type of intervention	Type of abuse*	No. of outcomes
Treliving (2002)[53]			psychotherapy service	Psychological support<br>• Group therapy aiming to provide men with a safe space to explore their difficulties and the impact of childhood sexual abuse	(CSA)	
Murphy et al (2007)[54]	Peer review	Pre-post	Public: Intellectual disability services	-<br>Perpetrator programme<br>• Group cognitive behavioural therapy for sexually abusive men	SVA	4
Oasis (2016)[55, 56]	Grey lit	Service evaluation	Third	-<br>Multi-service<br>• Counselling (6 sessions plus more if required)<br>• Refuge: a 12-bed refuge providing temporary emergency accommodation. Staff provide emotional and practical support	DVA	3
Ormston et al (2016)[57]	Grey lit.	Service evaluation	Third	*"The Caledonian System"*<br>Perpetrator programme<br>• Perpetrator programme covering lifelong change, responsibility for and to self, relationships, sexual respect, men and women, and children and fathering.<br>Multi-service<br>• Women's service including safety planning, information, advice, and emotional support to (ex)partners	DVA	6
Payne et al (2007)[58]	Peer review	Pre-post	Public (assumed as authors are clinical psychologists)	-<br>Psychological support<br>• Client centred group therapy	DVA<br>SVA<br>(CSA)	3
Peckham et al (2007)[59]	Peer review	Pre-post	Public: local community intellectual disability service	-<br>Psychological support<br>• Survivors group, involving educating clients and carers about sexual abuse, and weekly	SVA	6

For peer review only

Study	Peer review or grey lit	Study design	Setting/ sector	Type of intervention	Type of abuse*	No. of outcomes
				sessions using drawings, videos, dolls and pictures		
Robinson (2003)[60]	Grey lit.	Service evaluation	Mixed: Public (police, criminal justice) and third sector	*"Women's Safety Unit"*<br>Multi-service<br>• Advocacy, specialist counselling, legal services, housing services, refuge provision, target hardening and collecting evidence	DVA<br>SVA	11
Robinson (2004)[61]	Grey lit.	Service evaluation	Mixed: Public (police, probation, health, WSU, social services, housing, health) and third sector	-<br>MARAC<br>• Multiagency meeting which delegates actions to workers. Agencies include police, social services, probation, health, and education (where relevant)	DVA	6
Robinson (2006)[62]	Peer review	Pre-post	Mixed: Police, probation, health, WSU, social services, housing, health, third sector	-<br>MARAC<br>• Monthly multiagency meetings to discuss high risk victims, to share information and take actions to reduce future harm.	DVA	6
Robjant et al (2017)[63]	Peer review	Pre-post	Third	-<br>Psychological support<br>• Narrative exposure therapy, a therapy specifically designed for victims of trauma	SVA	2
Rose et al (2012)[64]	Peer review	Pre-post	Public: Community Learning Disability Services	-<br>Perpetrator programme<br>• Group programme with topics including sex education, emotion recognition, cognitive distortions, motivation for offences, empathy and relapse prevention	SVA	4
Ross et al.	Peer review	Non-	Public: Magistrate's	-	DVA	4

For peer review only

Study	Peer review or grey lit	Study design	Setting/ sector	Type of intervention	Type of abuse*	No. of outcomes
(2022)[65]		randomised comparison	Court	Advocacy<br>• An IDVA service based at a Specialist Domestic Abuse Court		
Ryan et al (2005)[66]	Peer review	Randomised controlled trial	Public: NHS psychology departments	-<br>Psychological support<br>• Individual and group therapy, focused on experiences and consequences of CSA	SVA (CSA)	4
SafeLives[67]	Grey lit.	Dataset / service evaluation	Third	-<br>Refuge<br>• No information	DVA	12
SafeLives[68]	Grey lit.	Dataset / service evaluation	Third	-<br>MARAC<br>• No information	DVA	3
SafeLives[69, 70]	Grey lit.	Dataset / service evaluation	Third	-<br>Sexual violence services<br>• No information	SVA	19
SafeLives[71]	Grey lit.	Dataset / service evaluation	Third	-<br>Health services<br>• No information	DVA	12
Safelives[72]	Grey lit.	Dataset / service evaluation	Third	-<br>Helpline<br>• No information	DVA	11
SafeLives[73-77]	Grey lit.	Dataset / service evaluation	Third	-<br>IDVA<br>• No information	DVA	12
SafeLives[78-82]	Grey lit.	Dataset / service evaluation	Third	-<br>Outreach<br>• No information	DVA	12

For peer review only

Study	Peer review or grey lit	Study design	Setting/ sector	Type of intervention	Type of abuse*	No. of outcomes
Sharpe et al (2001)[83]	Peer review	Pre-post	Public: Psychological therapies service	*"The Male Childhood Sexual Abuse Group"*<br>Psychological support<br>• Group therapy, no further information	SVA (CSA)	2
Skyner & Waters (1999)[84]	Peer review	NR	Mixed: Public (probation service) and third sector (NSPCC)	*"The Domestic Violence Prevention Programme"*<br>Perpetrator programme<br>• A cognitive behavioural programme with a variety of learning styles. A parallel support package is provided to (ex)partners and their children throughout	DVA	4
Smith et al (1995)[85]	Peer review	Pre-post	Public: Specialised therapy unit	*"Breakfree"*<br>Psychological support<br>• Individual therapy, daytime drop in facility, telephone contact, out-of-hours paging service, limited befriending service and a 'time out' facility where a client can stay in a safe environment for 1-2 nights	SVA (CSA)	3
Smith et al (2015)[86]	Peer review	Pre-post	Third	*"Domestic Abuse Recovering Together (DART)"*<br>Psychological support<br>• Mother and child sessions, designed to strengthen their relationship and support recovery. Followed by separate peer group sessions	DVA	3
Stanley et al (2011)[87]	Grey lit.	Service evaluation	Mixed: Third and Public (NHS, City Council)	*"Strength to change"*<br>Perpetrator programme<br>• Individual and group sessions over 40 weeks. Advocacy and advice are offered where appropriate. Sessions include mindfulness techniques, cognitive approaches, counselling, behavioural techniques.<br>• A women's service is available for	DVA	4

For peer review only

Study	Peer review or grey lit	Study design	Setting/ sector	Type of intervention	Type of abuse*	No. of outcomes
				(ex)partners offering practical and emotional support		
Stanley et al (2021)[88]	Grey lit	Service evaluation	Third	*"VOICES"*<br>Multi-service<br>• Trauma informed approach. Practitioners were given a new assessment framework, training and planning tools. No information on the specific types of activities or interventions is reported	DVA	6
Stanley et al (2021)[89]	Grey lit	Service evaluation	Third	*"SafeLives Co-Designed Pilots (SLCDPs)"*<br>Multi-service<br>• A suite of interventions for survivors, their children and perpetrators, using a whole family approach. This included community IDVA support, complex needs IDVA support, step down and recovery groups, children and young peoples work and 'Engage', which worked with the whole family	DVA	5
Strang et al (2017)[90]	Peer review	Randomised controlled trial	Mixed: Public (police), third sector	*"CARA"*<br>Perpetrator programme<br>• A two-day offender workshop, focusing on raising awareness, and moving offenders from denial and minimisation to acceptance and responsibility of harm, and conflict resolution	DVA	3
Taylor-Dunn & Erol (2019)[91, 92]	Grey lit	Service evaluation	Third	-<br>Advocacy<br>• Intensive one-to-one IDVA support within an immediate to medium term time-frame, implementing individual support plans and risk assessments to reduce risk and encourage recovery support	DVA	5

Study	Peer review or grey lit	Study design	Setting/ sector	Type of intervention	Type of abuse*	No. of outcomes
The Institute for Public Safety, Crime & Justice, University of Northampton (2019)[93]	Grey lit.	Service evaluation	Third	*"SHE"*<br>Psychological support<br>• A 'minimum support service (MSS)' with regular telephone check-ins, with an emphasis on safety and wellbeing, and an 'integrated support service (ISS+)' offering a face-to-face person-centred programme<br>Perpetrator programme<br>• 'Evolve', a programme domestic violence perpetrator programme working towards Respect accreditation	DVA	5
Trevillon et al (2014)[94]	Peer review	Non-randomised comparative	Public: Community mental health team	-<br>Advocacy<br>• Domestic violence training for clinicians, domestic violence manual for clinicians, mental illness training for domestic violence advisors, direct referral pathway to domestic violence advocacy, provision of a domestic violence advocacy service, comprising emotional and practical support, domestic violence education, support groups, safety planning and legal/housing support	DVA	11
Vallely et al (2005)[95]	Grey lit.	Service evaluation	Mixed: Public (Crown Prosecution Service, Police, Magistrates' Court, Probation service, local authorities) and Third sector	-<br>Specialist domestic violence court<br>• The service included a dedicated sitting for pre-trial matters and sentencing, allocated weekly; specialist magistrates with domestic violence training, specially trained CPS lawyers, and an independent advocacy service	DVA	5
Webster (2015)[96]	Grey lit.	Service evaluation	Third	*"Kent and Medway IDVA"*<br>Advocacy	DVA	2

For peer review only

Study	Peer review or grey lit	Study design	Setting/ sector	Type of intervention	Type of abuse*	No. of outcomes
				• Up to 21 IDVAs working with domestic abuse victims. No further information.		
Westmarland & Alderson (2013)[97]	Peer review	Pre-post	Third	-<br>Psychological support<br>• Face-to-face counselling, following a woman-centred, empowerment model, with elements of cognitive behavioural therapy, goal setting and rational emotive behaviour therapy	SVA	1
Williamson & Boyle (2012)[98]	Peer review	Pre-post	Public: NHS	*"IDVA Advocacy Intervention Programme"*<br>Advocacy<br>• No details reported	DVA	2
Williamson & Hester (2009)[99]	Grey lit.	Service evaluation	Third	*"South Tyneside Domestic Abuse Perpetrator Programme"*<br>Perpetrator programme<br>• A 'pro-feminist broadly cognitive behavioural model combined with gender analysis' approach. Group and one-to-one sessions.	DVA	2
Women's Aid (2022)[100-105]	Grey lit.	Service evaluation	Third	*"No Woman Turned Away"*<br>Housing (specialist caseworkers)<br>• Caseworkers provide telephone and email support to women looking for a refuge space	DVA	1
Women's Resource Centre (2011b)[106]	Grey lit.	Service evaluation	Third	*"Women and Girls Network"*<br>Psychological support<br>Counselling using trauma therapy, cognitive behavioural therapy, and integrated person-centred and feminist counselling practice. The organisation also offers a telephone helpline, body therapies, group programmes and specialist support projects	DVA	1
Women's Resource	Grey lit.	Service evaluation	Third	*"Ashiana"*<br>Multi-service	DVA	11

For peer review only

Study	Peer review or grey lit	Study design	Setting/ sector	Type of intervention	Type of abuse*	No. of outcomes
Centre (2011a)[107]				• Refuge: Safe accommodation across three schemes for South Asian, Turkish and Iranian women. Residents can stay for up to 1 year.<br>• Outreach: To provide support and reduce repeat victimisation. Ashiana also runs a DV support group for women in the wider community, as well as an end of therapy support group.		

For peer review only

1. ADVA. and S.P. Associates., *REPAIR (Resolve to End the Perpetration of Abuse in Relationships): A Community- and Whole-family-based Intervention Programme Targeting Perpetrators of Domestic Violence and Abuse in Devon. An evaluation of a three-year Invest to Save (ISB) PROJECT*. 2009: Exeter.
2. Advance, *'A Place to go Like This': Breaking the cycle of harm for mothers involved in offending who are survivors of domestic abuse, and their children.* 2020.
3. Barber, M., R. Jenkins, and C. Jones, *A survivor's group for women who have a learning disability.* British Journal of Developmental Disabilities, 2000. **46**(90, Pt 1): p. 31-41.
4. Bloomfield, S. and L. Dixon, *An outcome evaluation of the integrated domestic abuse programme (IDAP) and community domestic violence programme (CDVP).* London: National Offender Management Service, 2015.
5. Bowen, E., *Evaluation of a community based domestic violence offender rehabilitation programme. Unpublished doctoral thesis*, in *Centre for Forensic and Family Psychology*. 2004, University of Birmingham.
6. Bowen, E., *Therapeutic Environment and Outcomes in a UK Domestic Violence Perpetrator Program.* Small Group Research, 2010. **41**(2): p. 198-220.
7. Bowen, E. and E. Gilchrist, *Predicting dropout of court-mandated treatment in a British sample of domestic violence offenders.* Psychology, Crime & Law, 2006. **12**(5): p. 573-587.
8. Bowen, E., E. Gilchrist, and A.R. Beech, *Change in treatment has no relationship with subsequent re-offending in UK domestic violence sample: A preliminary study.* International Journal of Offender Therapy and Comparative Criminology, 2008. **52**(5): p. 598-614.
9. Bowen, E., E.A. Gilchrist, and A.R. Beech, *An Examination of the Impact of Community-Based Rehabilitation on the Offending Behaviour of Male Domestic Violence Offenders and the Characteristics Associated with Recidivism.* Legal and Criminological Psychology, 2005. **10**(2): p. 189-209.
10. Brady, F., et al., *Narrative exposure therapy for survivors of human trafficking: feasibility randomised controlled trial.* BJPsych Open, 2021. **7**(6).
11. Burton, S., L. Regan, and L. Kelly, *Supporting women and challenging men: lessons from the Domestic Violence Intervention Project.* 1998.
12. Callaghan, J., et al., *Make a Change: An evaluation of the implementation of an early response intervention for those who have used abusive behaviours in their intimate relationships*. 2020.
13. Respect and Women's Aid Federation England, *Make a Change Executive Summary*.
14. Calvert, R., S. Kellett, and T. Hagan, *Group cognitive analytic therapy for female survivors of childhood sexual abuse.* British Journal of Clinical Psychology, 2015. **54**(4): p. 391-413.
15. Clarke, S. and S. Llewelyn, *Personal constructs of survivors of childhood sexual abuse receiving cognitive analytic therapy.* British Journal of Medical Psychology, 1994. **67(3)**: p. 273-289.
16. Clarke, S. and C. Pearson, *Personal constructs of male survivors of childhood sexual abuse receiving cognitive analytic therapy.* British Journal of Medical Psychology, 2000. **73(2)**: p. 169-177.
17. Cook, D.A., et al., *The Berkeley group: ten years' experience of a group for non-violent sex offenders.* The British Journal of Psychiatry, 1991. **158**(2): p. 238-243.

For peer review only

18. Co-Ordinated Action Against Domestic, A., *Insights into domestic abuse 1: a place of greater safety.* 2012.
19. Co-ordinated Action Against Domestic Abuse (CAADA), *Insights National Dataset 2011–12 Appendix to: A place of greater safety*. 2012.
20. Coy, M. and L. Kelly, *Islands in the stream: an evaluation of four London independent domestic violence advocacy schemes*. 2011.
21. Dheensa, S., G. Penny, and Safelink, *"In my way…" Evaluation Of Learning Disabilities Sexual Abuse Support Services*.
22. Dobash, R.P., et al., *A research evaluation of British programmes for violent men.* Journal of Social Policy, 1999. **28**(2): p. 205-233.
23. Ellis, F., *Rehabilitation programme for adult survivors of childhood sexual abuse.* Journal of public mental health, 2012. **11**(2): p. 88-92.
24. Farrell, G. and A. Buckley, *Evaluation of a UK police domestic violence unit using repeat victimisation as a performance indicator.* The Howard Journal of Criminal Justice, 1999. **38**(1): p. 42-53.
25. Ferrari, G., et al., *Psychological advocacy towards healing (PATH): A randomized controlled trial of a psychological intervention in a domestic violence service setting.* Plos One, 2018. **13**(11).
26. Friendship, C., R.E. Mann, and A.R. Beech, *Evaluation of a national prison-based treatment program for sexual offenders in England and Wales.* Journal of interpersonal violence, 2003. **18**(7): p. 744-759.
27. Garry, K. and P. Goodwin, *Adult Counselling Service Phase two evaluation report: year 1 2020-21*. 2021, Merida Associates.
28. Karen Garry and Ellie Munro, *Adult Counselling Service Interim evaluation | May 2019*. 2019.
29. Karen Garry and Ellie Munro, *Adult Counselling Service Evaluation report – phase one 2016-2020*. 2020.
30. Geoghegan-Fittall, S., C. Keeble, and D. Wunsch, *The Croydon Drive Project: A 2-year Evaluation Final Report*. 2020.
31. Gilchrist, E., et al., *Using the Behaviour Change Wheel to design an intervention for partner abusive men in drug and alcohol treatment.* Pilot and feasibility studies, 2021. **7**(1): p. 1-14.
32. Gilchrist, G., et al., *ADVANCE integrated group intervention to address both substance use and intimate partner abuse perpetration by men in substance use treatment: a feasibility randomised controlled trial.* BMC public health, 2021. **21**(1): p. 1-20.
33. Goosey, J., L. Sherman, and P. Neyroud, *Integrated case management of repeated intimate partner violence: A randomized, controlled trial.* Cambridge Journal of Evidence-Based Policing, 2017. **1**(2): p. 174-189.
34. Halliwell, G., et al., *Cry for health: a quantitative evaluation of a hospital-based advocacy intervention for domestic violence and abuse.* Bmc Health Services Research, 2019. **19**(1).
35. Hester, M., et al., *Evaluation of Year 2 of the Drive Project – A Pilot to Address High Risk Perpetrators of Domestic Abuse*. 2019.
36. Hester, M., et al., *Evaluation of the Drive Project – a pilot to address high-risk perpetrators of domestic abuse Year 1 Feasibility Study*. 2017.
37. Hester, M., et al., *Evaluation of the Drive Project – A Three-year Pilot to Address High-risk, High-harm Perpetrators of Domestic Abuse*. 2019.
38. Hester, M., et al., *Evaluation of the Drive Project – A Three-year Pilot to Address High-risk, High-harm Perpetrators of Domestic Abuse EXECUTIVE SUMMARY.* 2020.
39. Hester, M. and N. Westmarland, *Tackling domestic violence: effective interventions and approaches*. 2005: Home Office Research, Development and Statistics Directorate.

For peer review only

40. Kelley, T.M., et al., *The Efficacy of Principle-Based Correctional Counseling for Improving the Self-Control and Mental Health of People Incarcerated for Sexual Violence.* Violence Against Women, 2022. **28**(2): p. 573-592.
41. Kelly, L., *Domestic Violence Matters: an evaluation of a development project*. 1999.
42. Kelly, L. and N. Westmarland, *DOMESTIC VIOLENCE PERPETRATOR PROGRAMMES STEPS TOWARDS CHANGE: Project Mirabal Final Report*. 2015.
43. Koppensteiner, M.F., J. Matheson, and R. Plugor, *Project 360: An intervention to address victim-police engagement in repeat domestic violence cases*. 2017.
44. Lindsay, W.R., et al., *Comparing offenders against women and offenders against children on treatment outcome in offenders with intellectual disability.* Journal of Applied Research in Intellectual Disabilities, 2011. **24**(4): p. 361-369.
45. Lindsay, W.R., et al., *The treatment of six men with a learning disability convicted of sex offences with children.* British Journal of Clinical Psychology, 1998. **37**: p. 83-98.
46. Lowe, M., et al., *CORE assessment of adult survivors abused as children: A NAPAC group therapy evaluation.* Counselling & Psychotherapy Research, 2017. **17**(1): p. 71-79.
47. McConnell, N., et al., *Caring dads safer children*. 2014.
48. McConnell, N., M. Barnard, and J. Taylor, *Caring Dads Safer Children: Families' Perspectives on an Intervention for Maltreating Fathers.* Psychology of Violence, 2017. **7**(3): p. 406-416.
49. McCracken, K. and T. Deave, *Evaluation of the Caring Dads Cymru programme. Final report.* 2012.
50. Michie, A.M. and W.R. Lindsay, *A treatment component designed to enhance empathy in sex offenders with an intellectual disability.* The British Journal of Forensic Practice, 2012. **14**(1): p. 40-48.
51. Morgan, S. and J. Parkes, *The Hampshire Domestic Abuse Prevention Partnership 2016-2018: process and outcomes evaluation.* 2018.
52. Morgan, S.A., B.M.S. McCausland, and J. Parkes, *Baseline characteristics and outcomes of the main perpetrator programme within the Hampshire Domestic Abuse Prevention Partnership, UK: A mixed methods study.* Plos One, 2019. **14**(7).
53. Morrison, A. and L. Treliving, *Evaluation of outcome in a dynamically orientated group for adult males who have been sexually abused in childhood.* British Journal of Psychotherapy, 2002. **19**: p. 59-75.
54. Murphy, G., et al., *Cognitive-behavioural treatment for men with intellectual disabilities and sexually abusive behaviour: A pilot study.* Journal of Intellectual Disability Research, 2007. **51(11)**: p. 902-912.
55. Oasis. *Impact report 2013-2014*. 2014; Available from: https://issuu.com/kristinagwynne/docs/oasis_annual_report_2013-2014-web/1.
56. Oasis, *Impact Report 2015-2016.* 2016.
57. Ormston, R., C. Mullholland, and L. Setterfield, *Caledonian system evaluation: analysis of a programme for tackling domestic abuse in Scotland*. 2016, Edinburgh: Scotland. Scottish Government Social Research. vi, 88.
58. Payne, A., H. Liebling-Kalifani, and S. Joseph, *Client-centred group therapy for survivors of interpersonal trauma: A pilot investigation.* Counselling & Psychotherapy Research, 2007. **7**(2): p. 100-105.
59. Peckham, N.G., S. Howlett, and A. Corbett, *Evaluating a survivors group pilot for a women with significant intellectual disabilities who have been sexually abused.* Journal of Applied Research in Intellectual Disabilities, 2007. **20**(4): p. 308-322.

For peer review only

60. Robinson, A.L., *The Cardiff Women's Safety Unit: A multi-agency approach to domestic violence.* Final Evaluation Report. Cardiff University: School of Social Sciences.(Available online at http://www. cf. ac. uk/socsi/whoswho/robinson. html), 2003.
61. Robinson, A.L., *Domestic Violence MARACs (Multi-Agency Risk Assesment Conferences) for Very High-risk Victims in Cardiff, Wales: A Process and Outcome Evaluation.* 2004.
62. Robinson, A.L., *Reducing repeat victimization among high-risk victims of domestic violence: The benefits of a coordinated community response in Cardiff, Wales.* Violence Against Women, 2006. **12(8)**: p. 761-788.
63. Robjant, K., J. Roberts, and C. Katona, *Treating posttraumatic stress disorder in female victims of trafficking using narrative exposure therapy: A retrospective audit.* Frontiers in Psychiatry, 2017. **8**.
64. Rose, J., et al., *A sex offender treatment group for men with intellectual disabilities in a community setting.* The British Journal of Forensic Practice, 2012.
65. Ross, J., J. Sebire, and H. Strang, *Tracking Repeat Victimisation After Domestic Abuse Cases Are Heard With and Without Independent Domestic Violence Advisors (IDVAs) in an English Magistrate's Court.* Cambridge Journal of Evidence-Based Policing, 2022: p. 1-15.
66. Ryan, M., et al., *A prospective study of the effectiveness of group and individual psychotherapy for women CSA survivors.* Psychology and Psychotherapy: Theory, Research and Practice, 2005. **78(4)**: p. 465-480.
67. SafeLives, *Insights refuge England and Wales dataset 2015-18 Adult refuge services*. 2018.
68. SafeLives, *Defining Marac Outcomes National Pilot*. 2012.
69. SafeLives, *Insights England and Wales dataset 2018-19 Sexual violence services* 2019.
70. SafeLives, *Insights SV dataset 2020-21 Sexual violence (SV) services*. 2021.
71. SafeLives, *Insights health England and Wales dataset 2015-2018 Adults health-based services*. 2018.
72. SafeLives, *Insights helpline England and Wales dataset 2014-17 Adult helpline services*. 2017.
73. SafeLives, *Insights Idva national dataset 2012–13 Adult independent domestic violence advisor (Idva) services*. 2015.
74. SafeLives, *Insights Idva national dataset 2013–14 Adult independent domestic violence advisor (Idva) services*. 2015.
75. SafeLives, *Insights Idva England and Wales dataset 2016-17 Adult independent domestic violence advisor (Idva) services*. 2017.
76. SafeLives, *Insights Idva England and Wales dataset 2018-19 Adult Independent domestic violence advisor (Idva) services*. 2019.
77. SafeLives, *Insights Idva dataset 2020-21 Adult Independent domestic violence advisor (Idva) services*. 2021.
78. SafeLives, *Insights outreach national dataset 2012–13 Adult outreach services*. 2015.
79. SafeLives, *Insights outreach national dataset 2013–14 Adult outreach services.* 2015.
80. SafeLives, *Insights outreach England and Wales dataset 2016-17 Adult outreach services*. 2017.
81. SafeLives, *Insights outreach England and Wales dataset 2018-19 Adult outreach services*. 2019.
82. SafeLives, *Insights outreach dataset 2020-21 Adult outreach services*. 2021.
83. Sharpe, J., et al., *Group analytic therapy for male survivors of childhood sexual abuse.* Group Analysis, 2001. **34**: p. 195-209.
84. Skyner, D.R. and J. Waters, *Working with perpetrators of domestic violence to protect women and children: a partnership between Cheshire Probation Service and the NSPCC.* Child Abuse Review: Journal of the British Association for the Study and Prevention of Child Abuse and Neglect, 1999. **8**(1): p. 46-54.

For peer review only

85. Smith, D., et al., *Adults with a history of child sexual abuse: Evaluation of a pilot therapy service.* British Medical Journal, 1995. **310(6988)**: p. 1175-1178.

86. Smith, E., et al., *Strengthening the mother-child relationship following domestic abuse: Service evaluation.* Child Abuse Review, 2015. **24**(4): p. 261-273.

87. Stanley, N., et al., *An evaluation of a new initiative for male perpetrators of domestic violence* 2011.

88. Stanley, N. and a. et, *Roadmap evaluation: final report - VOICES*. 2021, Bristol: Women's Aid
SafeLives. 279.

89. Stanley, N. and a. et, *Roadmap evaluation: final report - SLCDPs*. 2021, Bristol: Women's Aid
SafeLives. 279.

90. Strang, H., et al., *Reducing the harm of intimate partner violence: Randomized controlled trial of the Hampshire Constabulary CARA Experiment.* Cambridge Journal of Evidence-Based Policing, 2017. **1**(2): p. 160-173.

91. Holly Taylor-Dunn and Rosie Erol, *Evaluation of SaferPlaces' Independent Domestic Violence Advisor Services.* 2019.

92. Taylor-Dunn, H. and R. Erol, *Improving the safety and well-being of domestic abuse survivors: The role of a specialist organisation in supporting the work of Independent Domestic Violence Advisors.* Crime Prevention and Community Safety, 2021. **23**(2): p. 115-136.

93. Eve; The Institute for Public Safety, C.J., *2019*. Evaluation of SHE.

94. Trevillion, K., et al., *Linking abuse and recovery through advocacy: An observational study.* Epidemiology and Psychiatric Sciences, 2014. **23**(1): p. 99-113.

95. Vallely, C., *Evaluation of domestic violence pilot sites at Caerphilly (Gwent) and Croydon 2004/05.* 2005.

96. Webster, R., *Kent and Medway Independent Domestic Violence Advisor Service Impact Evaluation FINAL REPORT*. 2015.

97. Westmarland, N. and S. Alderson, *The health, mental health, and well-being benefits of rape crisis counseling.* Journal of Interpersonal Violence, 2013. **28**(17): p. 3265-3282.

98. Williamson, D. and A. Boyle, *Results of the domestic violence advocacy program at addenbrookes hospital.* Academic Emergency Medicine, 2012. **19**: p. 741.

99. Williamson, E. and M. Hester, *Evaluation of the South Tyneside Domestic Abuse Perpetrator Programme (STDAPP) 2006-2008.* University of Bristol, 2009.

100. Women's Aid, *Nowhere To Turn, 2022: Findings from the sixth year of the No Woman Turned Away project*. 2022.

101. Miles, C. and K. Smith, *Nowhere to turn, 2018: findings from the second year of the No Woman Turned away project*. 2018, Bristol: Women's Aid. 48.

102. Women's Aid, *NOWHERE TO TURN Findings from the first year of the No Woman Turned Away project*. 2017.

103. Women's Aid, *NOWHERE TO TURN Findings from the third year of the No Woman Turned Away project*. 2019.

104. Women's Aid, *NOWHERE TO TURN Findings from the fourth year of the No Woman Turned Away project*. 2020, Women's Aid Federation of England.

105. Women's Aid, *NOWHERE TO TURN 2021 Findings from the fifth year of the No Woman Turned Away project*. 2021.

106. Women's Resource, C., *Women and Girls Network: the WGN empowerment model - redressing negative individual and social impacts of gendered violence through holistic counselling.* 2011.

For peer review only

107. Women's Resource, C., *Ashiana Network: journey towards safety.* 2011.

For peer review only

Appendix 5: Outcome measures by domain and subdomain

Category	Domain	Subdomain	Measure	N
**Health**	**Mental health**	**Depression**		**16**
			Beck Depression Inventory	6
			Hospital Anxiety and Depression Scale - Depression	4
			Patient Health Questionnaire	3
			PROMIS depression Short Form	1
			Single item – "Is the client experiencing depression?"	1
			Single item – reduction in feeling depressed	1
		**Anxiety**		**10**
			Hospital Anxiety and Depression Scale - Anxiety	4
			Generalized Anxiety Disorder Scale	3
			PROMIS Anxiety Short Form	1
			Spiel State Anxiety	1
			Single item – "Is the client experiencing anxiety?"	1
		**Trauma**		**12**
			Primary care PTSD screen	2
			Impact of Event Scale	2
			PTSD Checklist for DSM-5 (PCL-5)	1
			PTSD Symptom Scale	1
			Posttraumatic Diagnostic Scale	1
			Post-traumatic Stress Disorder Scale	1
			Changes in Outlook Questionnaire	1
			The Clinician Administered PTSD Scale	1
			PTSD Checklist (PCL-C)	1
			Single item – "I have "flashbacks" about what happened"	1
		**General mental health**		**4**
			General Health Questionnaire (GHQ-28)	2
			Single item - "Current mental health rating"	1

For peer review only

Category	Domain	Subdomain	Measure	N
			No information – reported outcome is "Improved mental health"	1
		**Psychological symptoms**		**19**
			The Clinical Outcomes in Routine Evaluation - Outcome Measure (CORE-OM)	5
			The Symptom Checklist 90 revised (SCL-90R)	4
			Brief Symptom Inventory	2
			Jehu Beliefs Inventory	2
			The Shutdown Dissociation Scale (Shu-Dis)	1
			Dissociative Experiences Scale (DES)	1
			Belief Inventory	1
			Social Activity and Distress Scale	1
			Delusions, Symptoms and States Inventory	1
			Single item – “Is the client experiencing emotional instability?”	1
		**Anger**		**2**
			The Novaco Anger Scale	1
			PROMIS Anger Short Form	1
		**Understanding feelings$**		2
			Three Principles Inventory$	1
			Single item - "Understanding feelings better"$	1
		**Self-Harm / Suicide$**		**5**
			Single item – reduction in experiencing suicidal thoughts or feelings$	1
			Single item – reduction in self harm$	1
			Single item – “Is the client experiencing self-harm?” $	1
			Single item – “Is the client experiencing suicidal thoughts?” $	1
			Single item – “Is the client experiencing suicidal behaviours?” $	1
		**Healthcare utilisation / resource use$**		**5**
			Number / percent of participants using healthcare resources (e.g., A&E, GP, psychiatrist)$	3
			Number / percent of participants using community health and social service resources (e.g., health visitor, drug worker) $	1
			Days of admission$	1
		**Mental wellbeing$**		**4**

Category	Domain	Subdomain	Measure	N
			Short Warwick–Edinburgh Mental Wellbeing Scale (SWEMWBS) $	4
				2
		**Self-care$**	Single item - "Is the client experiencing trouble sleeping?" $	1
			Single item - "Is the client experiencing problems with eating?" $	1
				**3**
		**Quality of life$**	Short Form 12 (SF12) $	2
			Manchester Short Assessment of Quality of Life$	1
		**Health, mental health and wellbeing$**		**1**
			Taking Back Control Tool$	1
	**Physical health**	**Self-reported physical health**		**8**
			EQ-5D	3
			Visual analogue scale for physical health	2
			Single item – "Current physical health (poor/fair/good/very good/excellent)"	1
			No information – reported outcome is "Improved physical health (DV specific)"	1
			No information – reported outcome is "Improved physical health (not DV specific)"	1
		**Alcohol and drug use**		**5**
			Alcohol Use Disorders Identification Test (AUDIT) $	1
			Single item – "Current drug use (excessive/high/moderate/low/none)" $	1
			Single item – "Current alcohol use (excessive/high/moderate/low/none)" $	1
			Single item – "Alcohol problem (yes/no)" $	1
			Single item – "Drug problem (yes/no)" $	1
				**5**
		**Healthcare utilisation / resource use$**	Number / percent of participants using healthcare resources (e.g., A&E, GP, psychiatrist)$	3
			Number / percent of participants using community health and social service resources (e.g., health visitor, drug worker) $	1
			Days of admission$	1
		**Quality of life$**		**3**
			Short Form 12 (SF12) $	2

For peer review only

Category	Domain	Subdomain	Measure	N
			Manchester Short Assessment of Quality of Life[$]	1
		**Health, mental health and wellbeing[$]**		**1**
			Taking Back Control Tool[$]	1
	**Wellbeing**	**Wellbeing**		**7**
			Outcome Star	1
			Adult wellbeing scale	1
			Benefits of Therapy scale	1
			Personal Outcomes and Wellbeing Record (POWeR)	1
			Victim survey (perceived wellbeing - safety and wellbeing)	1
			Wellbeing survey (9 domains)	1
			CORE monitoring form	1
			Perceived Severity of Problems/Concerns Questionnaire	1
				**5**
			Social Inclusion Scale[$]	1
			Inventory of interpersonal problems 32 (IIP-32) [$]	1
		**Social ties/ relationships**	Single item - "Current social and community support (no social contact / negative social contacts / some positive contacts / majority positive contacts / all positive)" [$]	1
			No information – reported outcome is "Improved relationship with family"[$]	1
			No information – reported outcome is "Improved social support"[$]	1
				**2**
		**Understanding feelings**	Three Principles Inventory[$]	1
			Single item - "Understanding feelings better"[$]	1
				**4**
		**Mental wellbeing[$]**		
			Short Warwick–Edinburgh Mental Wellbeing Scale (SWEMWBS) [$]	4
		**Self-care[$]**		**2**

For peer review only

Category	Domain	Subdomain	Measure	N
			Single item - "Is the client experiencing trouble sleeping?" $	1
			Single item - "Is the client experiencing problems with eating?" $	1
				**3**
		**Quality of life$**	Short Form 12 (SF12) $	2
			Manchester Short Assessment of Quality of Life$	1
		**Health, mental health and wellbeing$**		**1**
			Taking Back Control Tool$	1
**Behavioural**	**Behavioural**	**Challenging behaviour**		**1**
			Challenging Behaviour Inventory	1
		**Self-Harm / Suicide$**		**5**
			Single item – reduction in experiencing suicidal thoughts or feelings$	1
			Single item – reduction in self harm$	1
			Single item – "Is the client experiencing self-harm?" $	1
			Single item – "Is the client experiencing suicidal thoughts?" $	1
			Single item – "Is the client experiencing suicidal behaviours?" $	1
		**Alcohol and drug use$**		**5**
			Alcohol Use Disorders Identification Test (AUDIT) $	1
			Single item – "Current drug use (excessive/high/moderate/low/none)" $	1
			Single item – "Current alcohol use (excessive/high/moderate/low/none)" $	1
			Single item – "Alcohol problem (yes/no)" $	1
			Single item – "Drug problem (yes/no)" $	1
**Empowerment**	**Empowerment**	**Self-esteem**		**12**
			Rosenberg's self-esteem scale	7
			Culture Free Self Esteem Inventory	2
			The Self Concept Questionnaire	1
			Self-esteem Assessment Tool	1
			No information – reported outcome is "Improved self-esteem"	1
		**Coping and resilience**		**3**
			Resilience Research Centre Adult Resilience Measure (RRC-ARM)	1

For peer review only

Category	Domain	Subdomain	Measure	N
			Cope and Recover Questionnaire	1
			No information – reported outcome is "Improved coping"	1
		**Locus of control**		**1**
			Review of Personal Effectiveness with Locus of Control	
		**Empowerment**		**5**
			The Empowerment Star	2
			Measure of Victim Empowerment Related to Safety Scale (MOVERS)	1
			Coping and confidence outcome measure	2
		**Assertiveness**		**1**
			Assertiveness Questionnaire	1
		**Knowledge**		**1**
			Goal Attainment Scale	1
		**Social ties/ relationships**[$]		**5**
			Social Inclusion Scale[$]	1
			Inventory of interpersonal problems 32 (IIP-32) [$]	1
			Single item - "Current social and community support (no social contact / negative social contacts / some positive contacts / majority positive contacts / all positive)" [$]	1
			No information – reported outcome is "Improved relationship with family"[$]	1
			No information – reported outcome is "Improved social support"[$]	1
		**Understanding feelings**[$]		**2**
			Three Principles Inventory[$]	1
			Single item - "Understanding feelings better"[$]	1
				2
			No information – reported outcome "Independence"	1
		**Independence**		
			No information – reported outcome "Increased likelihood of employment "	1
		**Awareness of support**		1

For peer review only

Category	Domain	Subdomain	Measure	N
			Number of criminal justice contacts	1
**Socio-economic circumstances**	**Socio-economic circumstances**	**Employment/ education**		**5**
			Single item - "Employment status (FT employed/PT employed/unemployed/caring for children/family)"	1
			Single item - "Employment status (paid employment/voluntary work/education/retired)"	1
			Single item - "Returned to/begun employment or education"	1
			Single item - "Education, employment and training (none / occasional / some sustained / sustained / sustained and fulfilling)"	1
			No information – reported outcome "Increased likelihood of employment"[$]	1
		**Finances**		**3**
			No information – reported outcome "Financial savings"	1
			Single item - "Perception of income (high/comfortable/managing with treats/managing with occasional treats/essentials only/struggling)"	1
			Single item - "Current financial situation (significant financial problems/being reliant on others/unable to pay for essentials/managing essentials but nothing left over/comfortably managing and no financial concerns)"	1
		**Housing**		**4**
			No information – reported outcome "Access to housing"	1
			Single item – "Current housing (homeless / transitory / unsuitable / temporary / secure)"	1
			Single item – "Number of women accommodated in suitable refuge spaces"	1
			Number / percent of participants using supported accommodation resources (e.g., weeks spent in refuge / staffed accommodation / B&B, boarding house or hotel / homeless and living with friends or family)	1
				**1**
				5
		**Social ties/ relationships[$]**	Social Inclusion Scale[$]	1
			Inventory of interpersonal problems 32 (IIP-32) [$]	1
			Single item - "Current social and community support (no social contact /	1

For peer review only

Category	Domain	Subdomain	Measure	N
			negative social contacts / some positive contacts / majority positive contacts / all positive)" $	
			No information – reported outcome is "Improved relationship with family"$	1
			No information – reported outcome is "Improved social support"$	1
**Changing perpetrator behaviour**	**Changing perpetrator behaviour**	**Anger**		**1**
			The Novaco Anger Scale	1
		**Perpetrator needs**		**1**
			Distribution of needs	1
		**Attitudes to women / violence**		**20**
			Inventory of Beliefs About Wife Beating	1
			Questionnaire on Attitudes Consistent with Sexual Offending (QACSO)	3
			Socio-Sexual Knowledge and Attitudes Assessment – Revised (SSKAAT-R)	1
			The Intimate Partner Violence Responsibility Attribution Scale (IPVRAS)	1
			URICA-DV Readiness to change	1
			Propensity for Abusiveness Scale (PAS)	2
			Paulhus Deception Scale (PDS)	1
			Interpersonal Reactivity Index (IRI)	1
			Sexual Attitudes and Knowledge Scale	1
			Victim Empathy Scale-Adapted	1
			Sexual Offenders Self Appraisal Scale	1
			Treatment motivation Questionnaire	1
			Interpersonal Dependency Inventory	1
			Attitudes Toward Exhibitionism Questionnaire	1
			No information - "Attitudes to the offence"	1
			No information - "Attitudes towards women"	1
			No information - "Acceptance of responsibility"	1
		**Desirable responding**		**3**
			Balanced Inventory of Desirable Reporting	3
		**Locus of control / self-control**		**6**
			Multidimensional Locus of Control Scale	1
			Rotter's Locus of Control Scale	1
			Brief self-control scale (BSCS)	1
			Low self-control scale (LSCS)	1

For peer review only

Category	Domain	Subdomain	Measure	N
			Levenson Locus of Control	1
			The Nowicki-Strickland Locus of Control Scale	1
		**Reasons for violence**		**2**
			Number of reasons for violence given	1
			Number of participants reporting each of 7 potential reasons for abuse	1
**Victim-perpetrator relationship**	**Victim-perpetrator relationship**	**Victim-perpetrator relationship**		**12**
			The Communication Patterns Questionnaire-Short Form (CPQ-SF)	1
			Respectful communication	1
			Awareness of self and others	1
			Attitude to partner (self-partner correlation)	1
			Single item - "Relationship status"	1
			Single item - "Hopes for the relationship"	1
			Single item - "Living circumstances (with perpetrator / not living together / intermittently living together)	6
**Parenting**	**Parenting**	**Parenting**		**7**
			Parent Empowerment and Efficacy Measure (PEEM)	1
			Camberwell Assessment of Need for Mothers (Short version)	1
			Parenting Stress Index	2
			Parental Acceptance-Rejection Questionnaire (PARQ)	2
			The Parental Locus of Control Scale	1
**DSVA**	**Experience of DSVA**	**Impact of abuse**		**2**
			Number of participants experiencing each of 18 potential impacts of abuse	1
			Mean number of impacts of abuse	
		**Indicators of ongoing abuse**		**5**
			Additional MARACs/Reappearance at MARAC	3
			Other evidence of abuse (e.g. harassing texts)	1
			Cases reopened	1
		**Perceived safety**		**7**
			Safety Behaviour Checklist	1
			Single item - "Partner fearful (1= never to 4=often)"	1

For peer review only

Category	Domain	Subdomain	Measure	N
			Single item - "Feeling of safety (much safer / somewhat safer / no change / less safe)"	1
			Safety (6 items)	2
			Single item - "How safe do you feel? (not safe / a little unsafe / somewhat safe / very safe)	1
			No information - reported outcome is "increased safety"	1
			No information - reported outcome is "avoidance of forced marriage"	1
		**Revictimization**		**4**
			Average number of repeat victims per month	1
			Number / percent of repeat victims	1
			Number / percent of women reporting repeat victimisation (for IPA)	1
			Number / percent of women disclosing further incidents of violence to IDVA	1
		**Presence of abuse$**		**46**
			Severity of Abuse Grid - presence: Number / percent of people responding yes to the presence of abuse (reported for physical abuse, sexual abuse, harassment and stalking and jealous and controlling behaviour) $	10*
			Severity of Abuse Grid - cessation: Number / percent of people reporting no abuse$	11*
			Severity of Abuse Grid - reduction in presence: Number / percent of people reporting a reduction of abuse (reported for physical abuse, sexual abuse, harassment and stalking and jealous and controlling behaviour) $	1
			Severity of Abuse Grid - multiple types: Number / percent of people reporting multiple types of abuse$	7*
			Number / percent of participants experience concurrent domestic abuse as well as sexual abuse$	1
			Number / percent of participants who have been subjected to adult sex offences$	1
			Number / percent of men abusing each month$	1
			Space for Action Survey (% responding yes to 12 indicators) $	1
			Physical and Sexual Violence Survey (% responding yes to 7 indicators) $	1
			Harassment and other abusive acts survey (% responding yes to 6 indicators) $	1
			Fathering survey / using children against a partner (% responding yes to 5 indicators) $	2

For peer review only

Category	Domain	Subdomain	Measure	N
			Social media perpetration / victimisation survey$	1
			Composite Abuse Scale$	3
			Abusive Behaviour Inventory$	2
			Controlling Behaviour Inventory$	3
		**Number of abusive incidents or behaviours$**		**4**
			Mean number of abusive incidents per man$	1
			Mean number of abusive behaviours reported by participants$	1
			Number / percent of participants experiencing between 0 and 7 behaviours (reported for emotional, physical and sexual behaviours) $	1
			IMPACT monitoring toolkit (reported for emotional, physical, and sexual behaviours) $	1
		**Severity of abuse$**		**29**
			Severity of Abuse Grid - severity: Number / percent of participants reporting high / moderate / standard severity (reported for physical abuse, sexual abuse, harassment and stalking and jealous and controlling behaviour) $	8*,±
			Severity of Abuse Grid - reduction in severity: Number / percent of participants reporting a reduction of high / moderate / standard / unknown severity (reported for physical abuse, sexual abuse, harassment and stalking and jealous and controlling behaviour) $	1
			Severity of Abuse Grid – escalation in severity: Number / percent of participants reporting escalation in severity of abuse$	2
			Severity of Abuse Grid - severity: Number / percent of participants reporting any form of severe abuse$	2
			Severity of Abuse Grid - high severity: Number / percent of participants reporting severe abuse (reported for physical abuse, sexual abuse, harassment and stalking and jealous and controlling behaviour) $	1
			Severity of Abuse Grid - severity: Number / percent of participants reporting multiple types of abuse that are high severity$	6*
			Severity of Abuse Grid - severity: Number / percent of participants reporting at least one type of abuse that is high severity$	6*
			Cambridge Crime Harm Index$	3
		**Frequency of abuse$**		**14**

Category	Domain	Subdomain	Measure	N
			Severity of Abuse Grid - frequency: Number / percent of participants reporting high / moderate / standard / unknown frequency (reported for physical abuse, sexual abuse, harassment and stalking and jealous and controlling behaviour) $	5*
			Severity of Abuse Grid - frequency escalation: Number / percent of participants reporting an escalation in frequency$	6*
			Frequency of abusive behaviours (scale 1 = never -3 = often) $	1
			Frequency rating of past violence (none (1) -> frequent (10) $	1
			Frequency rating of current violence (none (1) -> frequent (10) $	1
		**Duration of abuse$**		**1**
			Number / percent of victims with incidents recorded over a period longer than 12 months$	1
		**Severity and frequency of abuse$**		**13**
			Severity of Abuse Grid - severity and frequency escalation: Number / percent of participants reporting any escalation in severity or frequency of abuse$	7*
			Severity of Abuse Grid - severity and frequency escalation: Number / percent of participants reporting at least one form of abuse that is high severity and escalating in frequency or severity$	6*
		**Risk$**		**18**
			Spousal Assault Risk Assessment$	2
			DASH risk (high/med/standard) $	2
			Drive-DASH (mean score) $	1
			DASH risk (number with 1-14 assessments who had a decrease in score) $	1
			Change in risk in perpetrators (measurement tool unclear) $	1
			Weighted change in risk in perpetrators (measurement tool unclear) $	1
			IDVA perception of change in risk (do not know / increased / limited or no reduction / moderate reduction / significant reduction) $	1
			IDVA rated reduction in risk (percentage reporting 'some reduction in risk') $	1
			IDVA perception of sustainability of change in risk (Do not know / short term / medium term / long term / risk is permanently eliminated) $	1
			Men's perception of risk of using violence again (never/not sure/possibly) $	1

For peer review only

Category	Domain	Subdomain	Measure	N
			Risk factors - Perpetrators' crimogenic behaviour and aggravating problems (frequency and percent of 7 factors) $	1
			Risk factors - Specific features of abuse (frequency and percent of 10 factors) $	1
			Risk factors - Victims' appraisals of threat (frequency and percent of 3 factors) $	1
			Risk factors - Factors relating to children (frequency and percent of 3 factors) $	1
			Risk factors - Factors relating to victim (frequency and percent of 4 factors) $	1
			Probability of none / standard / moderate / and high abuse (reported for physical abuse / sexual abuse / harassment and stalking / jealous and controlling behaviour) $	1
		**Police incidents$**		**39**
			Average / total number of police incidents$	1
			Average / total number of DV related police incidents$	2
			Average / total number of incidents recorded by the police per month$	1
			Average number of DV complaints per month$	1
			Average number of arrests made per month$	1
			Average number of victims refusing to make a complaint per month$	1
			Average number of mental health related DV complaints per month$	1
			Average number of alcohol related DV complaints per month$	1
			Average number of drug related DV complaints per month$	1
			Average number of concern for children reports per month$	1
			Number / percent of participants that committed DV related police incidents$	1
			Number / percent of participants that committed (unspecified) police incidents$	1
			Number / percent of participants with (unspecified) arrests$	1
			Number / percent of calls to domestic incidents$	1
			Number / percent of repeat calls to domestic incidents$	1
			Number / percent of repeat calls to domestic violence incidents (number with 2 incidents, 3 incidents, 4 incidents… 14 incidents) $	1
			Number / percent of DV incidents where the perpetrator was not present$	1
			Number / percent of DV incidents that were crimed$	1
			Number / percent of DV incidents that led to arrests$	2

For peer review only

Category	Domain	Subdomain	Measure	N
			Number / percent of incidents with an arrest where a statement is given$	1
			Number / percent of incidents with an arrest and a statement is given where it is then retracted$	1
			Number / percent of crimes where perpetrator was charged/summonsed$	1
			Number / percent of police incidents before and after a MARAC$	1
			Number / percent of high / medium / low severity police incidents before and after a MARAC$	1
			Number / percent of women experiencing no further post-MARAC incidents of violence or abuse$	1
			Number / percent of women experiencing no further post-MARAC police complaints or call outs$	2
			Number / percent of women experiencing no further post-MARAC police complaints$	2
			Number / percent of women experiencing no further post-MARAC police call outs$	2
			Number / percent of women experiencing either additional post-MARAC police complaints or call outs$	1
			Number / percent of women experiencing both additional post-MARAC police complaints and call outs$	1
			Percent change in total calls to domestic incidents$	1
			Percent change in total calls to domestic incidents (as proportion of all calls) $	1
			Frequency of police call outs (never / once / 2-5 times / 6-10 times) $	1
			Cost of police incidents$	1
	**Perpetration of DSVA**	**Reoffending**		**20**
			Number / percent with proven reoffending (DV, core violence or any offence)	1
			Number / percent who reoffended (police incidents)	1
			Number / percent who committed further sex offences	2
			Number / percent who reoffended (no further details)	1
			Number / percent with repeat prosecution for violence against partner	1
			Number / percent with reconviction for another sexual offence	1
			Number / percent with reconviction for another violence offence	1
			Number / percent with reconviction for any offence	2

For peer review only

Category	Domain	Subdomain	Measure	N
			Number / percent linked to another crime (suspect or charged)	1
			Number / percent linked to another DVA crime (suspect or charged)	1
			Frequency of re-arrests for DVA	1
			Prevalence of re-arrests for DVA	1
			Number / percent of crimes that that were DVA related	1
			Number of incidents of DV whilst on the programme	1
			Number of incidents of non-DV crime whilst on the programme	1
			Number / percent of partners reporting subsequent violence	1
			Number / percent of partners reporting 'frequent violence' at follow up	1
			Time to reoffend	1
		**Criminal justice outcomes**		**16**
			Average number of people charged per month	1
			Average number of cases proceeding through court per month	1
			Average number of days between arrest and case finalisation	1
			Number / percent of cases discontinued	2
			Number / percent of cases bound over / other / found not guilty or absolute discharge / convicted	1
			Number / percent of victims retracting	1
			Number / percent of trials that went ahead on scheduled date	1
			Number / percent of trials that received a conviction	1
			Number / percent of charges that received a conviction	1
			Number / percent of guilty and not guilty pleas	1
			Number / percent of defendants who had the following sentences: conditional discharge / financial penalty / custody / community punishment order / community rehabilitation order	1
			Number / percent of cases with the following convictions: custodial sentence / non-custodial sentence / conditional discharge	1
			Number / percent of evidence types in CPS files (victim statement / police statement / defendant interview / witness statement / medical statement / case exhibits / 999 tape / forensic)	1
			Improvements specific to the 'CPS Criteria for Success'	1

For peer review only

Category	Domain	Subdomain	Measure	N
			Average monetary penalties (fines and compensation)	1
		**Presence of abuse$**		**46**
			Severity of Abuse Grid - presence: Number / percent of people responding yes to the presence of abuse (reported for physical abuse, sexual abuse, harassment and stalking and jealous and controlling behaviour) $	10*
			Severity of Abuse Grid - cessation: Number / percent of people reporting no abuse$	11*
			Severity of Abuse Grid - reduction in presence: Number / percent of people reporting a reduction of abuse (reported for physical abuse, sexual abuse, harassment and stalking and jealous and controlling behaviour) $	1
			Severity of Abuse Grid - multiple types: Number / percent of people reporting multiple types of abuse$	7*
			Number / percent of participants experience concurrent domestic abuse as well as sexual abuse$	1
			Number / percent of participants who have been subjected to adult sex offences$	1
			Number / percent of men abusing each month$	1
			Space for Action Survey (% responding yes to 12 indicators) $	1
			Physical and Sexual Violence Survey (% responding yes to 7 indicators) $	1
			Harassment and other abusive acts survey (% responding yes to 6 indicators) $	1
			Fathering survey / using children against a partner (% responding yes to 5 indicators) $	2
			Social media perpetration / victimisation survey$	1
			Composite Abuse Scale$	3
			Abusive Behaviour Inventory$	2
			Controlling Behaviour Inventory$	3
		**Number of abusive incidents or behaviours$**		**4**
			Mean number of abusive incidents per man$	1
			Mean number of abusive behaviours reported by participants$	1
			Number / percent of participants experiencing between 0 and 7 behaviours (reported for emotional, physical and sexual behaviours) $	1
			IMPACT monitoring toolkit (reported for emotional, physical, and sexual behaviours) $	1
		**Severity of abuse$**		**29**

For peer review only

Category	Domain	Subdomain	Measure	N
			Severity of Abuse Grid - severity: Number / percent of participants reporting high / moderate / standard severity (reported for physical abuse, sexual abuse, harassment and stalking and jealous and controlling behaviour) [$]	8*,±
			Severity of Abuse Grid - reduction in severity: Number / percent of participants reporting a reduction of high / moderate / standard / unknown severity (reported for physical abuse, sexual abuse, harassment and stalking and jealous and controlling behaviour) [$]	1
			Severity of Abuse Grid – escalation in severity: Number / percent of participants reporting escalation in severity of abuse[$]	2
			Severity of Abuse Grid - severity: Number / percent of participants reporting any form of severe abuse[$]	2
			Severity of Abuse Grid - high severity: Number / percent of participants reporting severe abuse (reported for physical abuse, sexual abuse, harassment and stalking and jealous and controlling behaviour) [$]	1
			Severity of Abuse Grid - severity: Number / percent of participants reporting multiple types of abuse that are high severity[$]	6*
			Severity of Abuse Grid - severity: Number / percent of participants reporting at least one type of abuse that is high severity[$]	6*
			Cambridge Crime Harm Index[$]	3
		**Frequency of abuse[$]**		**14**
			Severity of Abuse Grid - frequency: Number / percent of participants reporting high / moderate / standard / unknown frequency (reported for physical abuse, sexual abuse, harassment and stalking and jealous and controlling behaviour) [$]	5*
			Severity of Abuse Grid - frequency escalation: Number / percent of participants reporting an escalation in frequency[$]	6*
			Frequency of abusive behaviours (scale 1 = never -3 = often) [$]	1
			Frequency rating of past violence (none (1) -> frequent (10) [$]	1
			Frequency rating of current violence (none (1) -> frequent (10) [$]	1
		**Duration of abuse[$]**		**1**
			Number / percent of victims with incidents recorded over a period longer than 12 months[$]	1
		**Severity and frequency of abuse[$]**		**13**
			Severity of Abuse Grid - severity and frequency escalation: Number / percent of participants reporting any escalation in severity or frequency of abuse[$]	7*
			Severity of Abuse Grid - severity and frequency escalation: Number / percent	6*

For peer review only

Category	Domain	Subdomain	Measure	N
			of participants reporting at least one form of abuse that is high severity and escalating in frequency or severity$	
		**Risk$**		**18**
			Spousal Assault Risk Assessment$	2
			DASH risk (high/med/standard) $	2
			Drive-DASH (mean score) $	1
			DASH risk (number with 1-14 assessments who had a decrease in score) $	1
			Change in risk in perpetrators (measurement tool unclear) $	1
			Weighted change in risk in perpetrators (measurement tool unclear) $	1
			IDVA perception of change in risk (do not know / increased / limited or no reduction / moderate reduction / significant reduction) $	1
			IDVA rated reduction in risk (percentage reporting 'some reduction in risk') $	1
			IDVA perception of sustainability of change in risk (Do not know / short term / medium term / long term / risk is permanently eliminated) $	1
			Men's perception of risk of using violence again (never/not sure/possibly) $	1
			Risk factors - Perpetrators' crimogenic behaviour and aggravating problems (frequency and percent of 7 factors) $	1
			Risk factors - Specific features of abuse (frequency and percent of 10 factors) $	1
			Risk factors - Victims' appraisals of threat (frequency and percent of 3 factors) $	1
			Risk factors - Factors relating to children (frequency and percent of 3 factors) $	1
			Risk factors - Factors relating to victim (frequency and percent of 4 factors) $	1
			Probability of none / standard / moderate / and high abuse (reported for physical abuse / sexual abuse / harassment and stalking / jealous and controlling behaviour) $	1
		**Police incidents$**		**39**
			Average / total number of police incidents$	1
			Average / total number of DV related police incidents$	2
			Average / total number of incidents recorded by the police per month$	1
			Average number of DV complaints per month$	1
			Average number of arrests made per month$	1

Category	Domain	Subdomain	Measure	N
			Average number of victims refusing to make a complaint per month$	1
			Average number of mental health related DV complaints per month$	1
			Average number of alcohol related DV complaints per month$	1
			Average number of drug related DV complaints per month$	1
			Average number of concern for children reports per month$	1
			Number / percent of participants that committed DV related police incidents$	1
			Number / percent of participants that committed (unspecified) police incidents$	1
			Number / percent of participants with (unspecified) arrests$	1
			Number / percent of calls to domestic incidents$	1
			Number / percent of repeat calls to domestic incidents$	1
			Number / percent of repeat calls to domestic violence incidents (number with 2 incidents, 3 incidents, 4 incidents… 14 incidents) $	1
			Number / percent of DV incidents where the perpetrator was not present$	1
			Number / percent of DV incidents that were crimed$	1
			Number / percent of DV incidents that led to arrests$	2
			Number / percent of incidents with an arrest where a statement is given$	1
			Number / percent of incidents with an arrest and a statement is given where it is then retracted$	1
			Number / percent of crimes where perpetrator was charged/summonsed$	1
			Number / percent of police incidents before and after a MARAC$	1
			Number / percent of high / medium / low severity police incidents before and after a MARAC$	1
			Number / percent of women experiencing no further post-MARAC incidents of violence or abuse$	1
			Number / percent of women experiencing no further post-MARAC police complaints or call outs$	2
			Number / percent of women experiencing no further post-MARAC police complaints$	2
			Number / percent of women experiencing no further post-MARAC police call outs$	2
			Number / percent of women experiencing either additional post-MARAC police complaints or call outs	1

For peer review only

Category	Domain	Subdomain	Measure	N
			Number / percent of women experiencing both additional post-MARAC police complaints and call outs	1
			Percent change in total calls to domestic incidents	1
			Percent change in total calls to domestic incidents (as proportion of all calls)	1
			Frequency of police call outs (never / once / 2-5 times / 6-10 times)	1
			Cost of police incidents	1

Note: Severity of Abuse Grid: there was a diverse approach to how it was used by different authors in the literature, , therefore we decided to split outcomes according to the various ways in which it was operationalised, rather than just reporting 'Severity of Abuse Grid' as an outcome.

* Note: Some studies do not explicitly specify using the Severity of Abuse Grid, but this has been presumed because the wording used when reporting outcomes matches exactly.

± Note: One study uses the outcome measure to assess perpetrators using abusive behaviours, rather than victim-survivor experiences of abuse.

$ Note: These outcomes and subdomains appear in more than one domain.

For peer review only

For peer review only

Frequency of outcomes by outcome domain, and the number of studies reporting one or more outcomes per outcome domains by intervention type, source of study, violence type and sector

		Outcome measure domain										
		Mental health	Physical health	Wellbeing	Behavioural	Empowerment	Socio-economic circumstances	Changing perpetrator beliefs and skills	Victim-perpetrator relationship	Parenting	Experience of DSVA	Perpetration of DSVA
Number of studies reporting at least one outcome		29	11	19	5	21	9	14	10	5	42	46
Number of total outcome measures		85	22	25	11	32	17	33	12	7	189	201
Number of total unique outcome measures		52	16	21	11	23	17	28	7	5	106	119
**Type of intervention**	**No. of studies**											
Perpetrator programme	27	2	1	2	1	1	1	14	4	3	14	21
Victim support:												
Psychological support	21	17	1	7	2	10	2	0	0	1	1	0
Advocacy	14	5	4	4	0	4	2	0	2	1	12	11
Multi-service	11	4	4	4	1	5	1	0	0	0	8	5
Housing	2	0	0	0	0	0	1	0	1	0	1	1
MARAC	3	0	0	0	0	0	0	0	0	0	3	2
Specialist domestic violence police teams	3	0	0	1	0	0	0	0	0	0	2	2
Specialist domestic violence court	1	0	0	0	0	0	0	0	0	0	0	1
Sexual violence service	1	1	1	1	1	1	1	0	0	0	1	1
Helpline	1	0	0	0	0	0	0	0	1	0	1	1
Outreach	1	0	0	0	0	0	0	0	1	0	1	1
Health	1	0	0	0	0	0	1	0	1	0	1	1
Other / Unclear	1	0	0	0	0	0	0	0	0	0	1	1

**Source**												
Peer reviewed	41	22	6	10	3	11	3	9	1	3	13	19
Grey literature	39	7	5	9	2	10	6	5	9	2	29	27
**Type of violence**												
Domestic violence and abuse	53	11	9	12	2	10	6	8	10	5	40	41
Sexual violence and abuse	15	7	2	4	2	5	1	6	0	0	1	4
Domestic and sexual violence and abuse	3	2	0	0	0	1	0	0	0	0	1	1
Childhood sexual abuse	9	9	0	3	1	5	2	0	0	0	0	0
**Sector**												
Third	39	12	7	14	3	12	7	5	9	4	27	23
Public	26	13	2	3	1	8	2	5	0	1	5	10
Mixed	12	3	2	1	1	0	0	2	1	0	9	11
Private	1	1	0	1	0	1	0	1	0	0	0	0
Unclear	2	0	0	0	0	0	0	1	0	0	1	2

Note: Sixteen studies reported an outcome that fits into multiple domains therefore some outcomes are represented by multiple domains. Additionally, 38 studies reported an outcome that was categorised as both the experience of DSVA and perpetration of DSVA, because the specific outcome could represent either domain depending on who is completing the outcome measure.

Note: Two studies have outcome domains that are represented in multiple intervention types (perpetrator programme, unclear/other and advocacy), because they report a perpetrator programme and an associated women's service, but it is unclear which intervention/service the outcomes were measured as part of.

Note: The number of studies for the different intervention/service types is more than the total number of studies (80) because 7 studies reported a perpetrator programme and a women's service.

For peer review only

Figure 4: Temporal trends in outcome domains reported in DVSA evaluations, 1991 to 2022

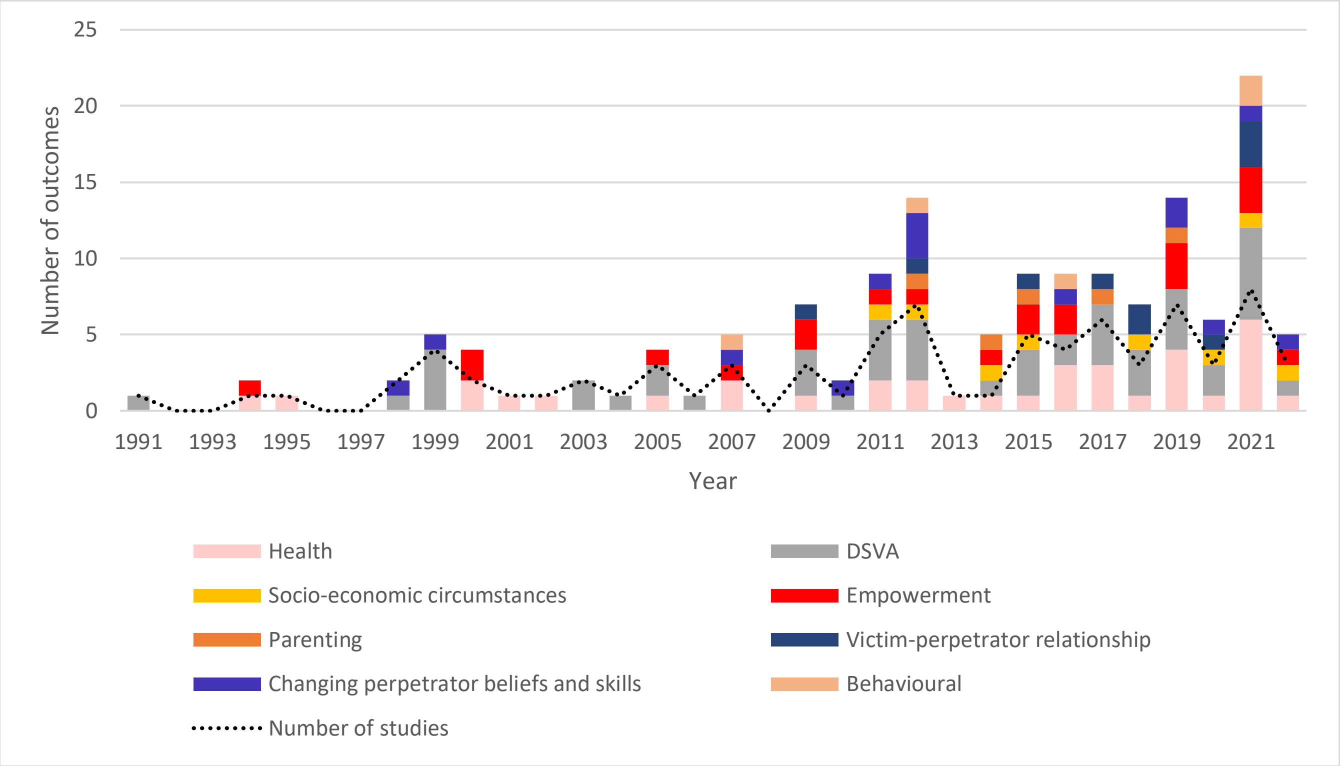

Note: For ease of interpretation, mental health, physical health and wellbeing domains have been combined into one health domain, and the experience of DSVA and perpetration of DSVA domains have been combined into a single 'DSVA' domain.
Note: Sixteen studies reported an outcome that crosses two or more domains therefore some outcomes are represented by multiple domains. Additionally, 38 studies reported an outcome that was categorised as both the experience of DSVA and perpetration of DSVA, because the specific outcome could represent either domain depending on who is completing the outcome measure.
